# Supplementary material for: Local energetic frustration conservation in protein families and superfamilies
Source: Nat Commun. 2023 Dec 16;14:8379. doi: 10.1038/s41467-023-43801-2 (PMC10725452; doi:10.1038/s41467-023-43801-2)
Supplement: Supplementary file 1 — Supplementary Information [file 41467_2023_43801_MOESM1_ESM.pdf]

Supplementary Note 1

Differentially conserved frustration networks in the human Ras superfamily

Comparing superfamilies that have a distribution of broader evolutionary distances among their members proves more challenging than for the globin case. The human RAS superfamily comprises a broad range of proteins that despite their sequence and functional diversification, share both a common structural framework and enzymatic activity related to the production of GDP by the hydrolysis of GTP[1]. This group of small GTPases play crucial roles in cellular signal transduction pathways, regulating processes such as cell growth, proliferation, and differentiation [2]. The activation of RAS signaling is mediated by guanine nucleotide exchange factors (GEFs), while GTPase-activating proteins (GAPs) facilitate the hydrolysis of GTP to GDP, thereby inactivating RAS [3]. These proteins exist in two conformational states: an active GTP-bound state capable of binding to effectors to transduce signals and an inactive GDP-bound state. There are 5 RAS subfamilies, i.e. RAS, RHO, RAB, ARF and RAN, that bind to different effectors. The conserved GTP-binding site consists of 5 motifs, named G1 to G5, that contain the core residues essential for the GTPase activity and its associated conformational changes. These motifs have been shown to harbor different SDPs linked to family-specific functionalities [1]. Mutations in RAS genes are commonly associated with various human cancers, including colorectal, lung, and pancreatic cancers, highlighting the importance of RAS signaling in oncogenesis [4]. Targeting RAS proteins has been an area of intense research for developing anticancer therapeutics [5]. The understanding of RAS biology and its implications in disease has been greatly advanced by studies utilizing molecular and cellular approaches, as well as animal models [6].

We retrieved all paralogs within the RAS superfamily in humans [1] and calculated their frustration conservation patterns (see Methods). The RAN family was omitted from our analysis as it contained only one sequence. As a consequence of the high energetic variability within each family, the frustration conservation analysis using the SRFI analysis shows little conservation for the G1-G5 motifs (Supplementary Fig 4) with only a few minimally frustrated and neutral positions being frustrationally conserved (FrstIC>0.5) across all families. Most of the minimally frustrated positions, i.e.: 14, 19, 20, 53, 56 and 81, are located in the G1 and G3 motifs. Within the G1 and G3 regions, there is relatively more energetic conservation compared to G2, G4, and G5. Additionally, in G4, N and K residues, which play a role in substrate specificity by contacting the oxygen of the purine [7], are maximally frustrated and conserved. Surprisingly, the T35 residue, present within a loop region in G2, crucial for sensing GTP and binding the Mg2+ ion [7], is conserved in terms of sequence in most members, but no conservation of energy is observed, or if conserved, it is neutral. This lack of frustration conservation is caused by the conformational diversity of the region across the different family members.

Inorder to gain resolution, we explored frustration conservation at the level of residue-residue contacts based on the mutational FI. This index was used instead of the configurational one as we expect functional signals to be more dependent on the amino acids identity than the conformational state of the protein. When mapped onto the reference structures for each RAS family, highly frustrated interactions are localized around the G1-G5 motifs whereas the rest of the fold is mainly enriched in minimally frustrated interactions (Supplementary Fig 5A). To enhance interpretability, we represented the interactions involving only residues that belong to the G1-G5 motifs or that are SDPs in each subfamily as graphs (Supplementary Fig 5B). Despite the large evolutionary distances, it is noticeable the highly connected network of frustrated interactions involving mainly G1 and G4 motifs, undetected when performing the SRFI. More in detail, highly frustrated interactions involving mainly Lys16 (G1), Asp57 (G3) and Lys117 (G4) are present in all four subfamilies. These interactions involve residues that interact with the GTP or the Mg2+ ion as well as some residues that interact with other protein partners (Supplementary Fig 6). RHO is the most divergent family as it has an extra helix next to G4,

after which there appears Pro138 (Pro124 in RAS numbering) that participates in many highly frustrated interactions with Lys117 and Lys118. These interactions within RHO are part of a different highly frustrated interaction network from the one containing Lys16 and Asp57.

Beyond similarities, there are energetic differences between the different subfamilies which seem to be related to SDPs (marked with asterisks in Supplementary Fig 4). As an example, SDP 83 (RAS numbering) shows family specific requirements; being neutral or without local frustration conservation at the single residue level but with conserved frustration levels in its interactions with other residues. This SDP is a highly conserved Asp with highly frustrated interactions (Supplementary Fig 5B) in ARF and RAB. In contrast, SDP 83 is a conserved Ser in RHO that establishes minimally frustrated interactions. The SDP identity is not conserved in RAS, having a mixture of both highly frustrated and minimally frustrated interactions (the latter with Val81, another SDP). Other SDPs, e.g. 20, 56 and 81 (RAS numbering) are minimally frustrated with variable identities within the hydrophobic and polar group of amino acids. The change in identity within the SDPs that interact with each other seems to be evolutionarily compensated in each subfamily, possibly finely tuning specific interactions with other residues.

In the case of non-SDP residues, such as the highly conserved residue G60, known to adopt different conformations in the active or inactive state of RAS [8] its behavior varies across subfamilies. In certain cases, this variability may be attributed to the ability of AlphaFold to capture different conformational states in the protein models, influencing the local structure for equivalent residues in homologous regions and therefore influencing their frustration values. Similarly, T35, which undergoes conformational changes depending on whether it binds with GDT or not [9], displays both minimally and maximally frustrated contacts across the ARF subfamily and only minimal frustration in the RHO subfamilies. Notably, in RAS and RAB, the contact energy is not conserved at all or, if conserved, it appears to be neutral. Despite expecting similar behavior to that observed in ARF and RHO, considering its mobile nature (as seen in the case of G60), we are limited in capturing differing conformations while analyzing a single protein. As a future endeavor, we could combine FrustraEvo will algorithms that are able to predict conformational ensembles for a given protein instead of just one structure [10].

The differential evolutionary conservation of frustration in the RAS superfamily adds a layer of information that is simply not possible to observe just using SDPs information and that complements existing literature. Moreover, it offers a local energetic perspective of how fully conserved residues in sequences differentially interact with other functional residues, e.g. those in the G1-G5 region, like residue G60 or T35.

Supplementary Note 2

Unsupervised Analysis of the SARSCov2 Proteome in the context of the entire Beta Coronaviruses phylogeny with FrustraEvo

We have used our pipeline to process 29 SARS-CoV-2 proteins or protein domains (see Methods) from which 22 of them passed our quality filters (more than 10 sequences and average pLDDT over aligned positions of the cluster ≥ 80; see Methods, Supplementary Fig 7). For each cluster we compared sequence and frustration conservation by calculating the mean SeqIC and FrustIC per position and per cluster. In **Fig. 4A** we observe a significant positive correlation between these values (r=0.69, pvalue=2.6e-14). Some protein families like cluster 5 in the C-terminal domain of the Nucleoprotein (N_CTerm) deviate from the correlation. These have a lower FrustIC than expected (Supplementary Fig 8). Close inspection of this case shows that this family has regions with low pLDDT scores (Supplementary Fig 9A) regardless of the median for the entire protein being pLDDT≥80, which is known to correlate with flexible regions [11]. Hence the conformation of the region and its frustration values can be heterogeneous and therefore yield lower FrustIC than expected given the sequence diversity in the family. At the same time, however, other factors such as the predicted amount of disordered residues (Supplementary Fig 9B) or the MSA phylogenetic diversity (Supplementary Fig 9C) can also influence the FrustIC and SeqIC relationship. On the other side of the spectrum, we have cases like cluster 2 in the non-structural protein (nsp) 13 (Supplementary Fig 8) for which the analysis of frustration conservation explains much more than expected from the overall correlation with sequence, because at some loci different amino acids can have similar frustration states, e.g, hydrophobic residues in the core of the structure (Supplementary Fig 10). Finally, when assessing full protein families we have found that some proteins, e.g. the C-terminal of the SARS-Unique Domain (SUD_Cterm) of nsp3 (3 subfamilies, FrustIC sd=0.042) or the Envelope (E) protein (2 subfamilies, FrustIC sd=0.046) are very homogeneous in terms of their average FrustIC values across subfamilies while others, e.g. N_Cterm (5 subfamilies, FrustIC sd=0.217), N_Nterm (6 subfamilies, FrustIC sd=0.20) or nsp8 (6 subfamilies, FrustIC sd=0.176), show a large amount of energetic variability across them. Once again, the quality of the models, the phylogenetic variability and flexibility of protein regions are factors that might influence these observations but no clear trend was identified (Supplementary Fig 9). Further information for all the families can be found in Supplementary Table 3 and Supplementary Table 4.

As we were interested in using this type of analysis to detect evolutionary constraints in SARS-CoV-2, we next focused on analyzing the Sarbecovirus subfamily that contains the proteins of this virus. In Fig. 4B we show the proportions for frustrationally conserved (FrustIC>0.5, either MIN, NEU or MAX frustrated) and non-conserved (FrustIC≤0.5) residues. This type of analysis allows us to rapidly sort proteins according to their frustration conservation level, which ultimately relates to there being different degrees of selective pressure on different proteins of the virus. The proportion of frustrationally conserved residues across proteins is heterogeneous (93.6% in the case of the E protein and up to 48.3% for the N_CTerm domain) reflecting the fact that some proteins are much more constrained by their family features than others. From those that are frustrationally conserved, the majority of residues belong to the neutral (mean = 0.35, sd =0.06) or minimally frustrated (mean=0.29, sd =0.06) classes, as expected. An interesting aspect to quantify is how much consistency there is between the Sarbecovirus family and the SARS-CoV-2 proteins. This would let us know to what extent the family restrictions are still present in the virus, whether the restrictions are of a different nature, i.e. stability vs. function, or whether there are novel constraints in the SARS-CoV-2 that were not present in the family. We found 332 residues conserved in the highly frustrated state within the proteome of the SARS-CoV-2 family (Supplementary Table 5) from which 301 residues were also conserved and highly frustrated in SARS-CoV-2 itself. This suggests that the functional signals, related to the presence of highly frustrated interactions in those regions, are coherent both at the family and at the SARS-CoV-2 levels. We found several residues where the frustration

conservation state differs between SARS-CoV-2 and its family. There are 62 residues that are conserved (FrustIC>0.5) in the neutral or minimally frustrated state at the family level but conserved in a highly frustrated state in SARS-CoV-2, suggesting recent gain of function events (Supplementary Table 6). While the majority of these residues are located in proteins that are well studied (Spike n=18, nsp5 n=6), it is noticeable that other less characterized ones contain many of these type of residues as well (nsp2 n=9, nsp3 domains n=9), defining interesting positions for their study (Supplementary Table6). In addition, there are 345 positions that are conserved and highly frustrated in SARS-CoV-2 but that are frustrationally conserved at the family level (Supplementary Table 5).

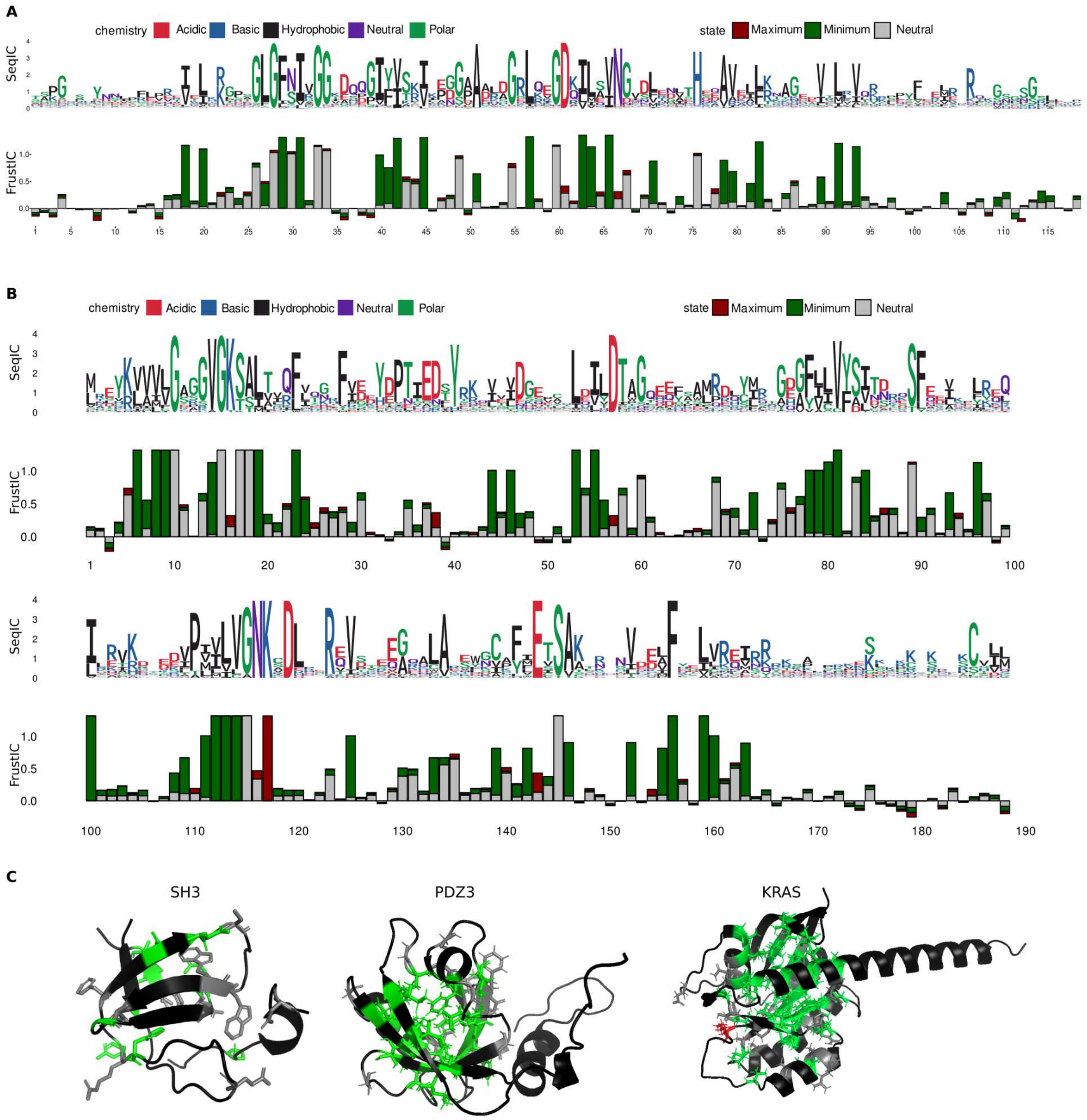

Supplementary Fig. 1. (A) Sequence and Frustration logo plots showing SeqIC and FrustIC values per MSA column respectively for PSD95-PDZ3 The numbering of the plot corresponds to the sequence of reference chain A from PDB 1BE9. Positions containing a gap in the sequences of reference are not considered in the plot. Protein PSD95-PDZ3 has been trimmed to match tested positions for mutation fitness in [14]. (B) Sequence and Frustration logo plots for KRAS calculated with Rojas et al. data. Numbering corresponds to the protein of reference P01116-2. (C) FrustIC results mapped to SH3, PDZ3 and KRAS protein models. Residues with FrustIC<=0.5 are shown in black. Residues with FrustIC>0.5 are coloured according to the frustration state that contributes more information to the overall FrustIC value.

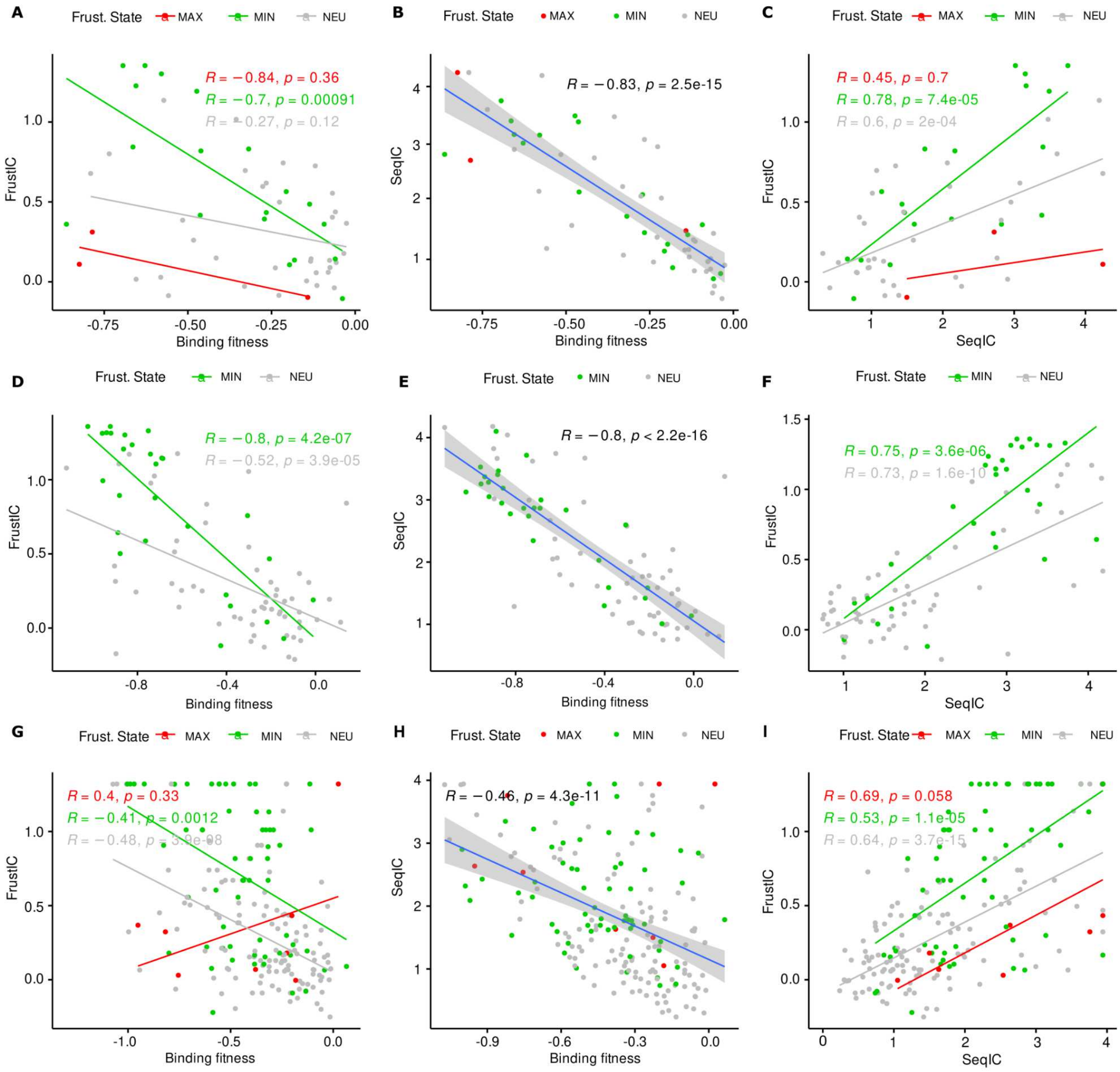

Supplementary Fig. 2. Pearson correlation between FrustIC or SeqIC and binding fitness and FrustIC vs SeqIC in SH3 domain (A, B, C), protein domain PDZ3 (D, E, F) and KRAS protein (G, H, I). P-value corresponds to a two-sided test. Error bands in the correlation plots correspond to a 95% confidence interval.

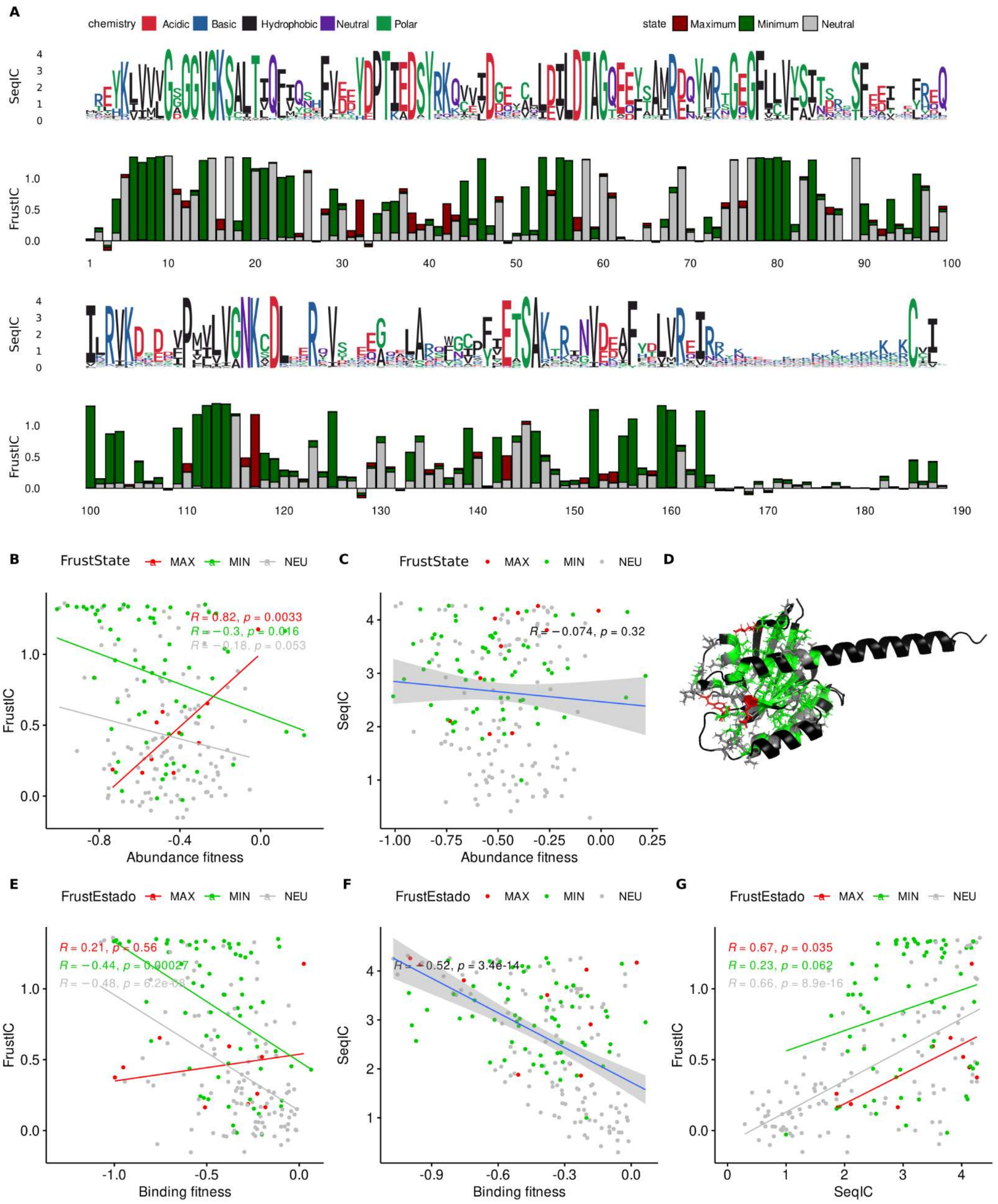

Supplementary Fig. 3. Results of KRAS with automatic retrieval of sequences of the protein family. (A) Sequence and Frustration logo plots for KRAS. Numbering corresponds to the protein of reference P01116-2. Pearson correlation between FrustIC or SeqIC and abundance fitness (B and C) or binding fitness (E and F) and FrustIC vs SeqIC (G). (D) FrustIC results mapped to KRAS proteins models. Residues with FrustIC<=0.5 are shown in black. Residues with FrustIC>0.5 are coloured according to the frustration state that contributes more information to the overall FrustIC value. P-value corresponds to a two-sided test. Error bands in the correlation plots correspond to a 95% confidence interval.

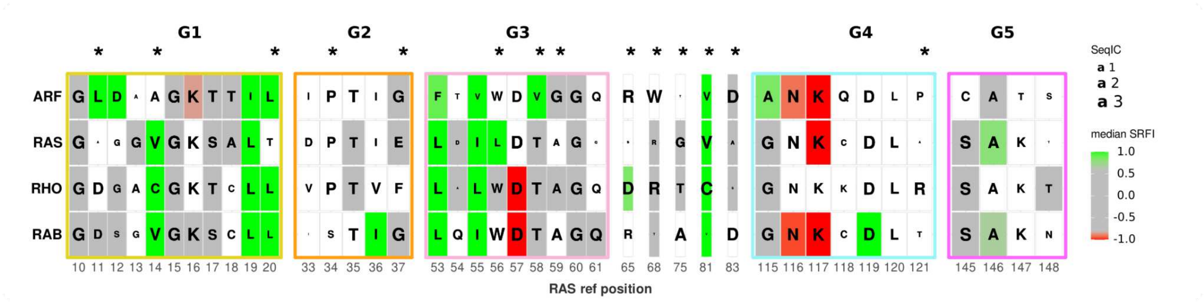

Supplementary Fig 4. Consensus multiple sequence frustration alignment (MSFA) comparing FrustIC and SeqIC results for the G1-G5 motifs and SDPs (marked with asterisks) in each of the subfamilies. Consensus amino acid identities are shown for each family. The size of the letter represents the SeqIC. The background color corresponds, in shades from green through gray to red, to the median single residue frustration index (SRFI) of that position across all structures in the family. White background means that FrustIC <= 0.5.

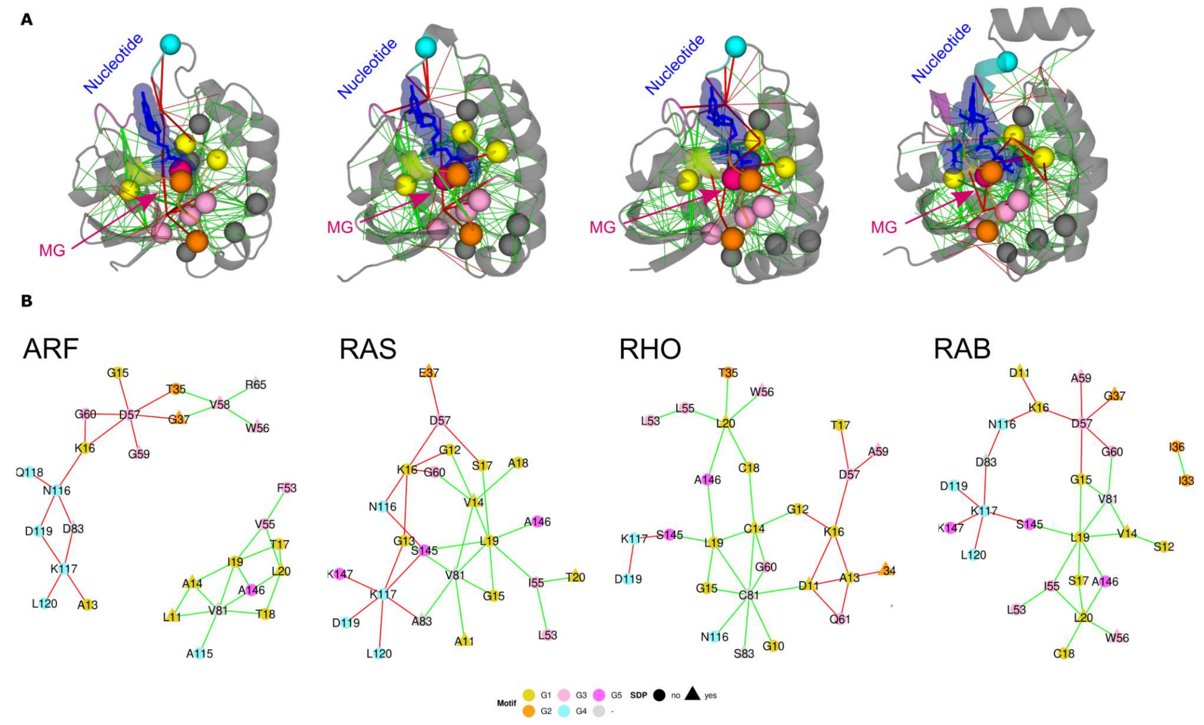

Supplementary Fig 5. Frustration conservation patterns unmask strongly conserved constraints across the Human RAS superfamily. A) Conserved highly frustrated (red lines) and minimally frustrated (green lines) interactions (FrustIC>0.5) according to the mutational FI. Cα of SDPs are in globular shape. G1 motif is in yellow, G2 orange, G3 light pink, G4 cyan and G5 in magenta. Structures shown correspond to PDBs 7MGE (ARF), 3TKL (RAB), 121P (RAS) and 6BCB (RHO). B) Networks representing conserved highly frustrated (red lines) or minimally frustrated (green lines) interactions (FrustIC>0.5) between residues within the G1-G5 motifs (circular shape) or SDPs (triangular shape) in at least 50% of the structures of each subfamily. Nodes with triangular shape correspond to SDPs outside the G1-G5 motifs.

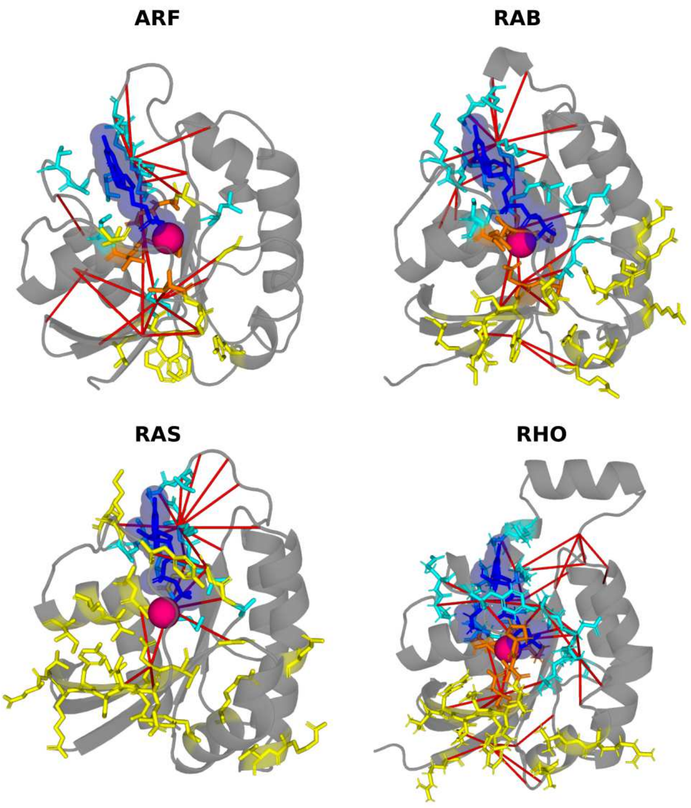

Supplementary Fig. 6. Highly frustrated contacts between residues of the ARF, RAB, RAS or RHO proteins (PDB codes 7MGE, 3TKL, 1XD2 and 6BCB respectively) and the GTP nucleotide (in dark blue and sticks) or the Mg ion (in magenta and spherical shape). Additionally, residues colored in blue or yellow and represented in sticks are residues that interact with the GTP and Mg ligands or a protein partner included in the PDB file respectively (inter-residue distance ≤ 5Å). When they are colored in orange means that they participate in both types of interfaces (protein and ligand).

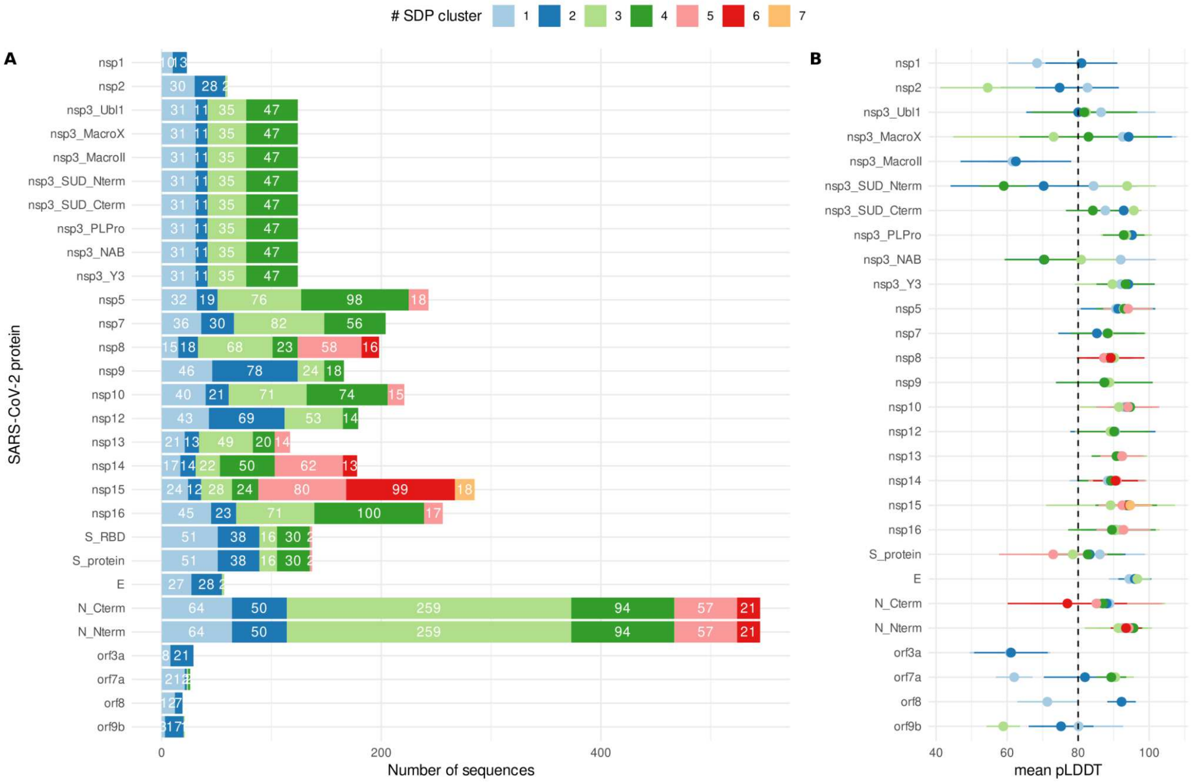

Supplementary Fig. 7. Coronavirus S3Det cluster and AlphaFold2 models quality. (A) Barplot depicting the distribution of sequences per S3Det cluster of all proteins or protein domains considered in the study (see Tables S2 and S3). Clusters with less than 10 sequences were not considered in our analyses. (B) Mean pLDDT score per S3Det cluster of each protein or protein domain. The dashed line at pLDDT=80 represents the minimum quality threshold for a cluster to be considered. Below that we considered that the models are of low quality and therefore removed from the analysis.

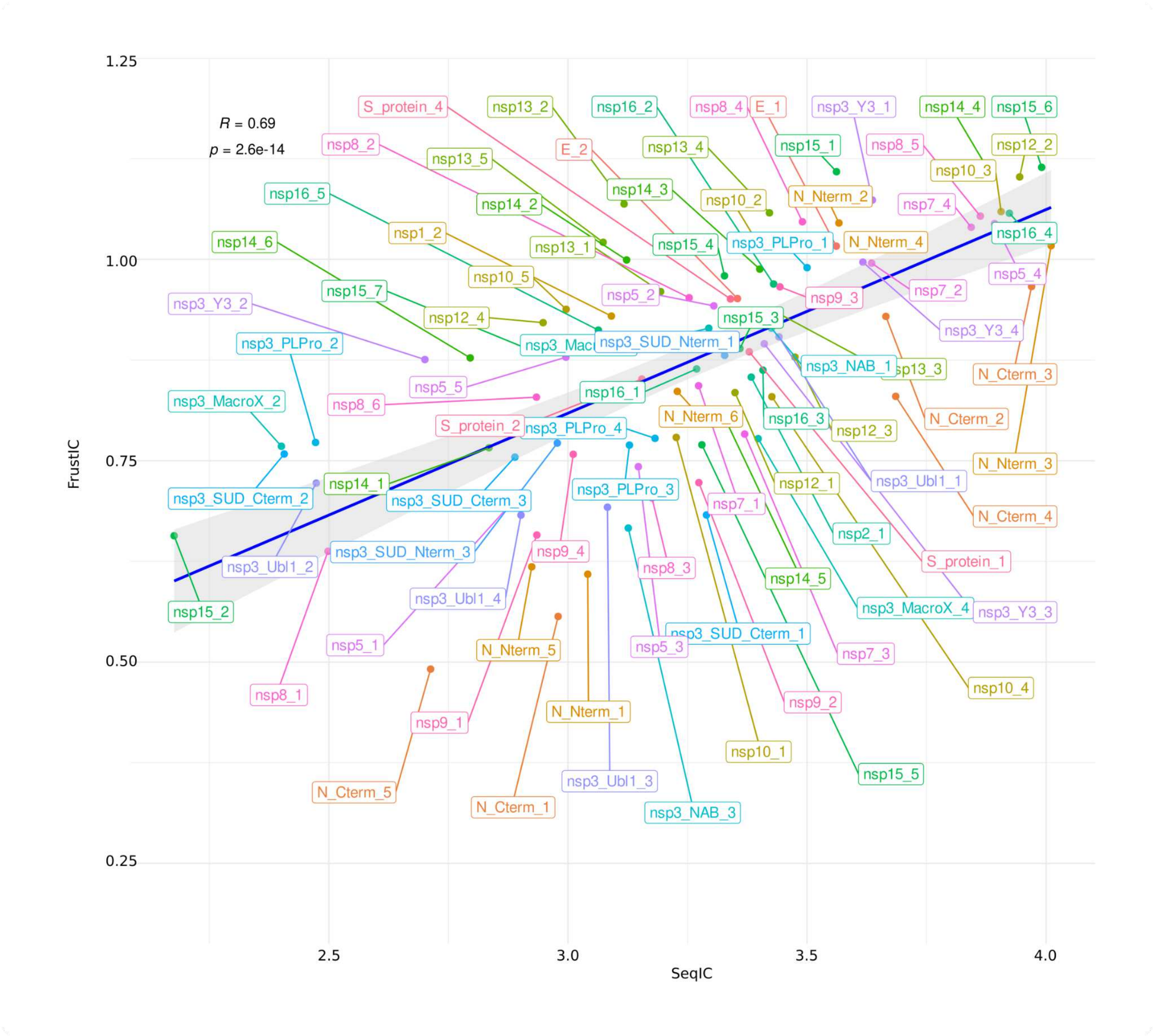

Supplementary Fig. 8. Same correlation plot as in Fig. 5 showing mean FrustIC vs mean SeqIC per S3Det cluster computed for Coronavirus proteins (see Methods) but with all the data points labelled by protein and S3Det cluster.

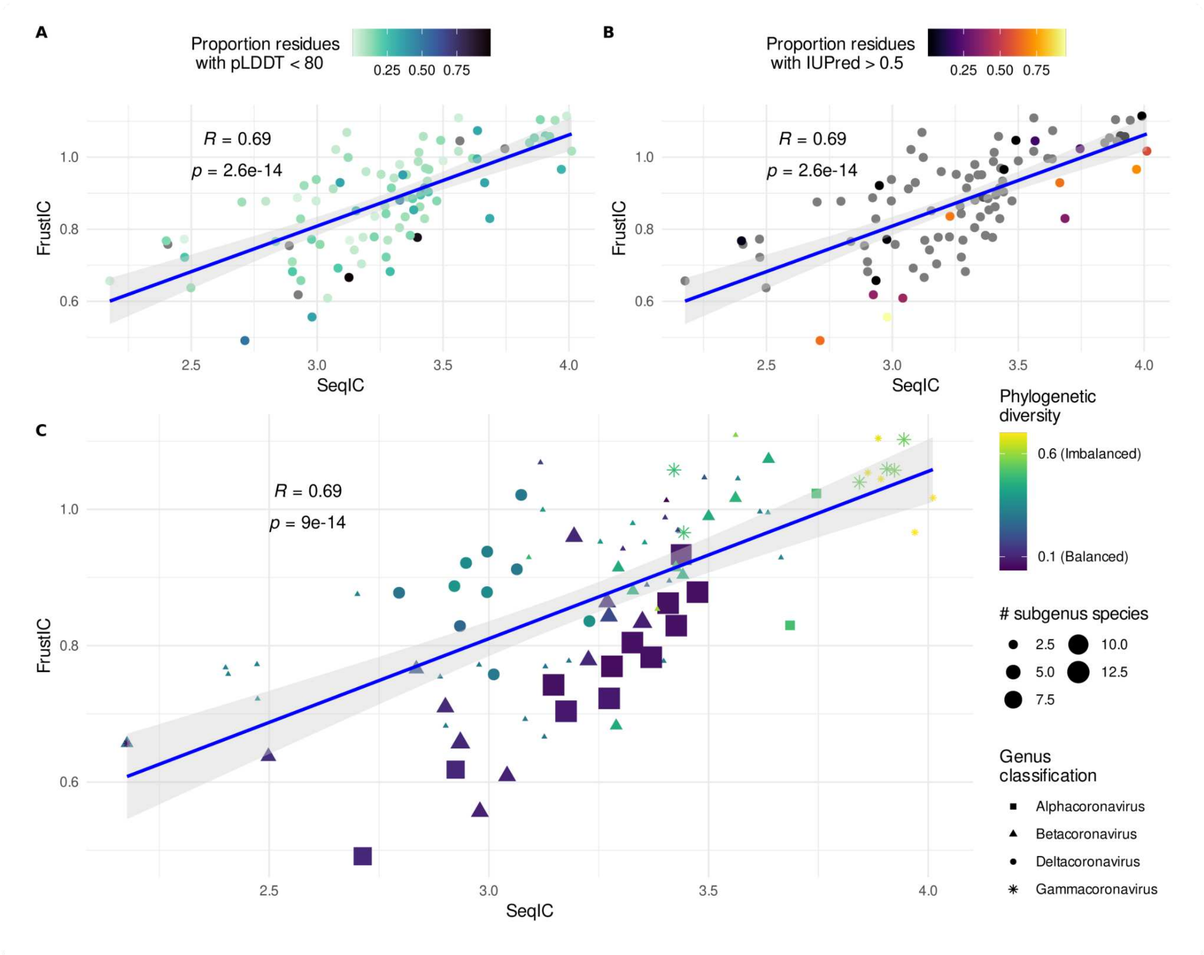

Supplementary Fig. 9. Factors affecting the expected correlation between SeqIC and FrustIC in Coronaviruses. (A) Quality of S3Det clusters models per protein represented by the mean proportion of residues with pLDDT<80 (low quality models). (B) Disorder tendency of S3Det clusters models represented by the mean proportion of residues with IUPred>0.5 (disordered). Grey dots indicate that no residues were found with IUPred> 0.5. (C) Phylogenetic balance represented by the diversity of the subgenus classification within each protein and S3Det cluster (see Methods). The size of the point represents the number of subgenus species represented by the considered sequences. The shape of the point refers to the corresponding genus classification (Alpha, Beta, Gamma or Deltacoronavirus) of all the sequences in each S3Det cluster.

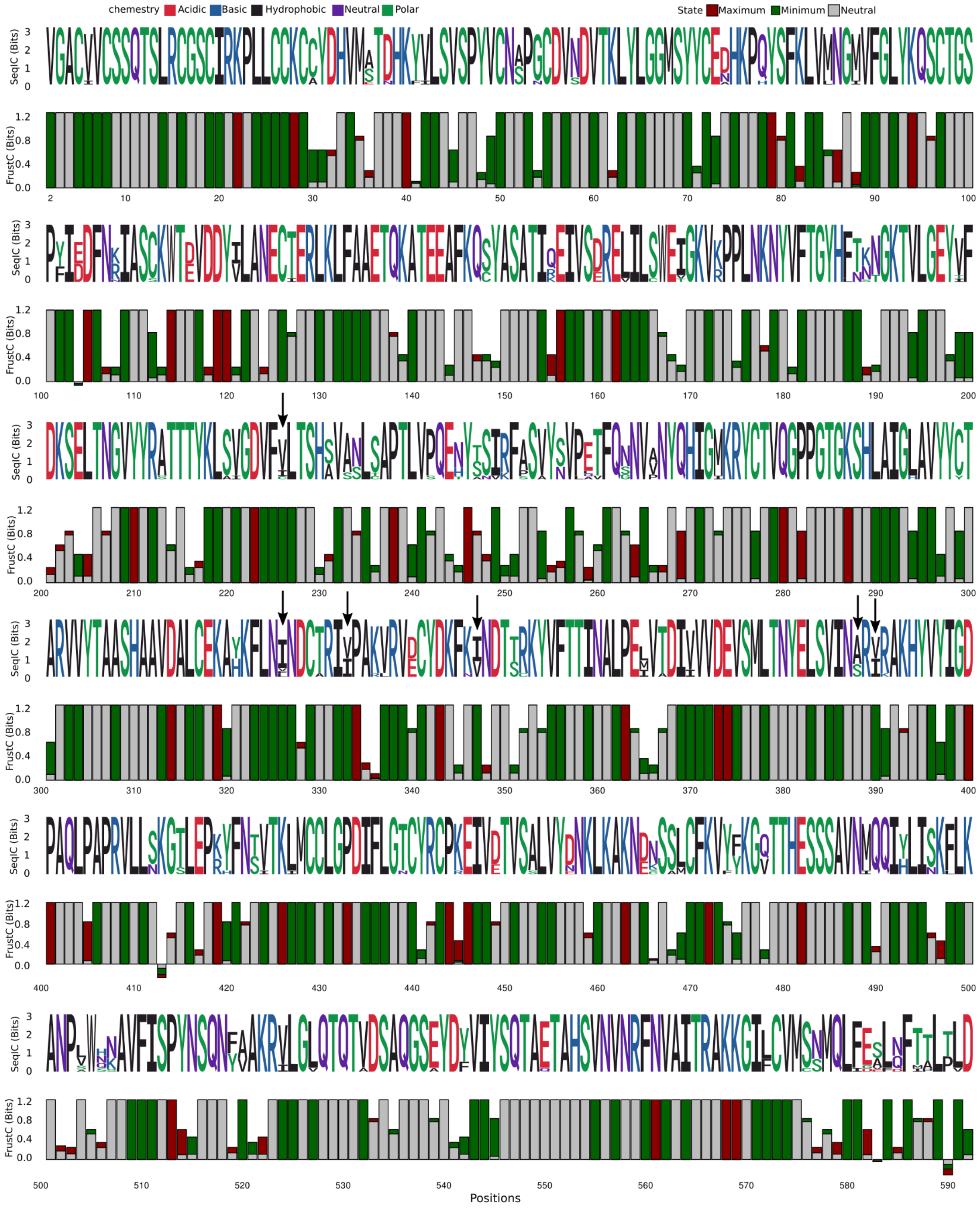

Supplementary Fig. 10. Sequence and Frustration logo plots showing SeqIC and FrustIC values per MSA column respectively for the cluster 2 of Coronavirus protein nsp13. The numbering of the plot corresponds to the sequence of reference AYR18613.1. Positions containing a gap in the sequences of reference are not considered in the plot. Arrows point to examples of hydrophobic residues with lower conservation of SeqIC compared to FrustIC in a minimally frustration state.

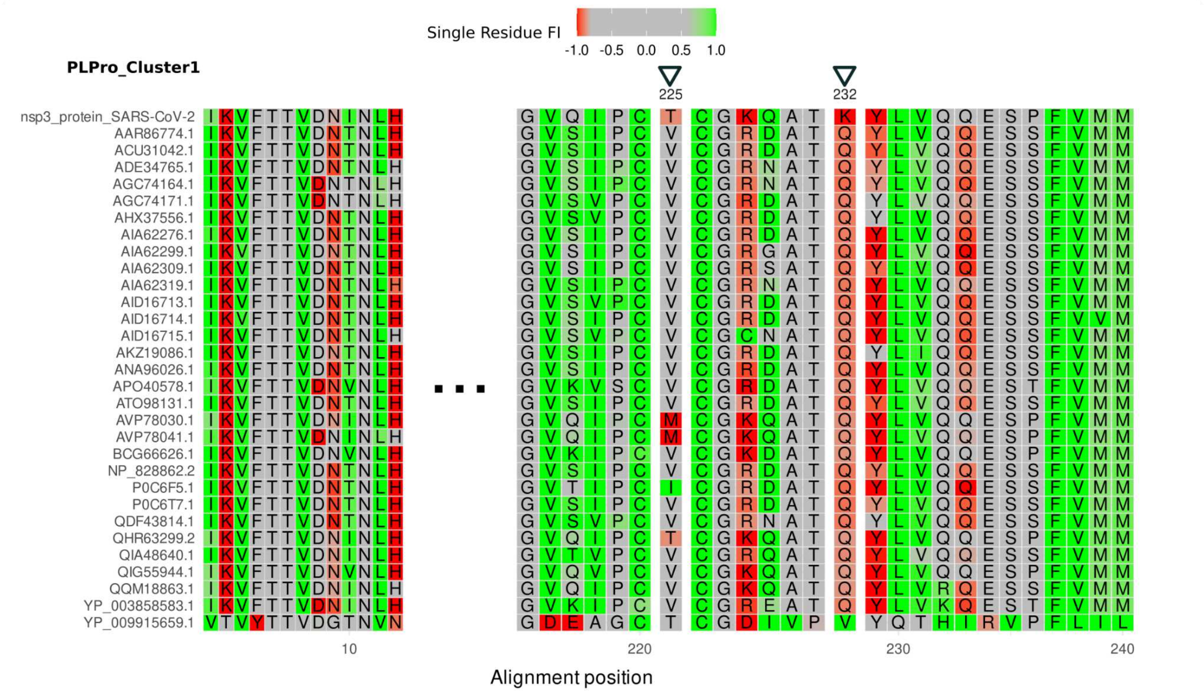

Supplementary Fig. 11. Selected positions of the multiple sequence frustration alignment of PLPro S3Det cluster1 (Sarbecovirus). Highlighted positions, marked in triangle, 221/225 and 228/232 (Alignment position / SARS-CoV-2 numbering) representing interesting examples of change in frustration in the SARS-CoV-2 sequence (first row) compared to the rest of sequences.

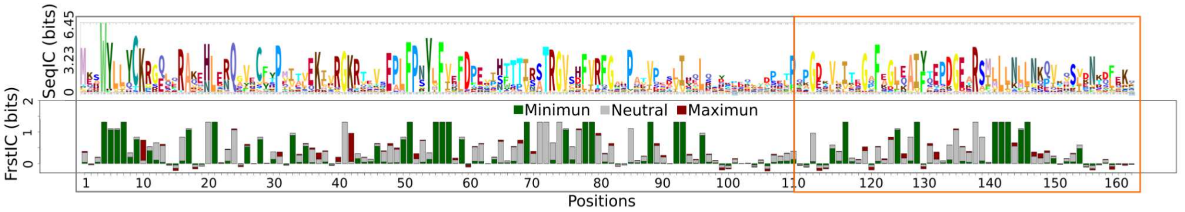

Supplementary Fig. 12. Conservation of local frustration and sequence identity for RfaH family. FrustIC based on the single-residue level frustration index. In green are represented the minimally frustrated; in red highly frustrated contacts; and in gray neutral. In gray box is the non-metamorphic region and in orange box the metamorphic region.

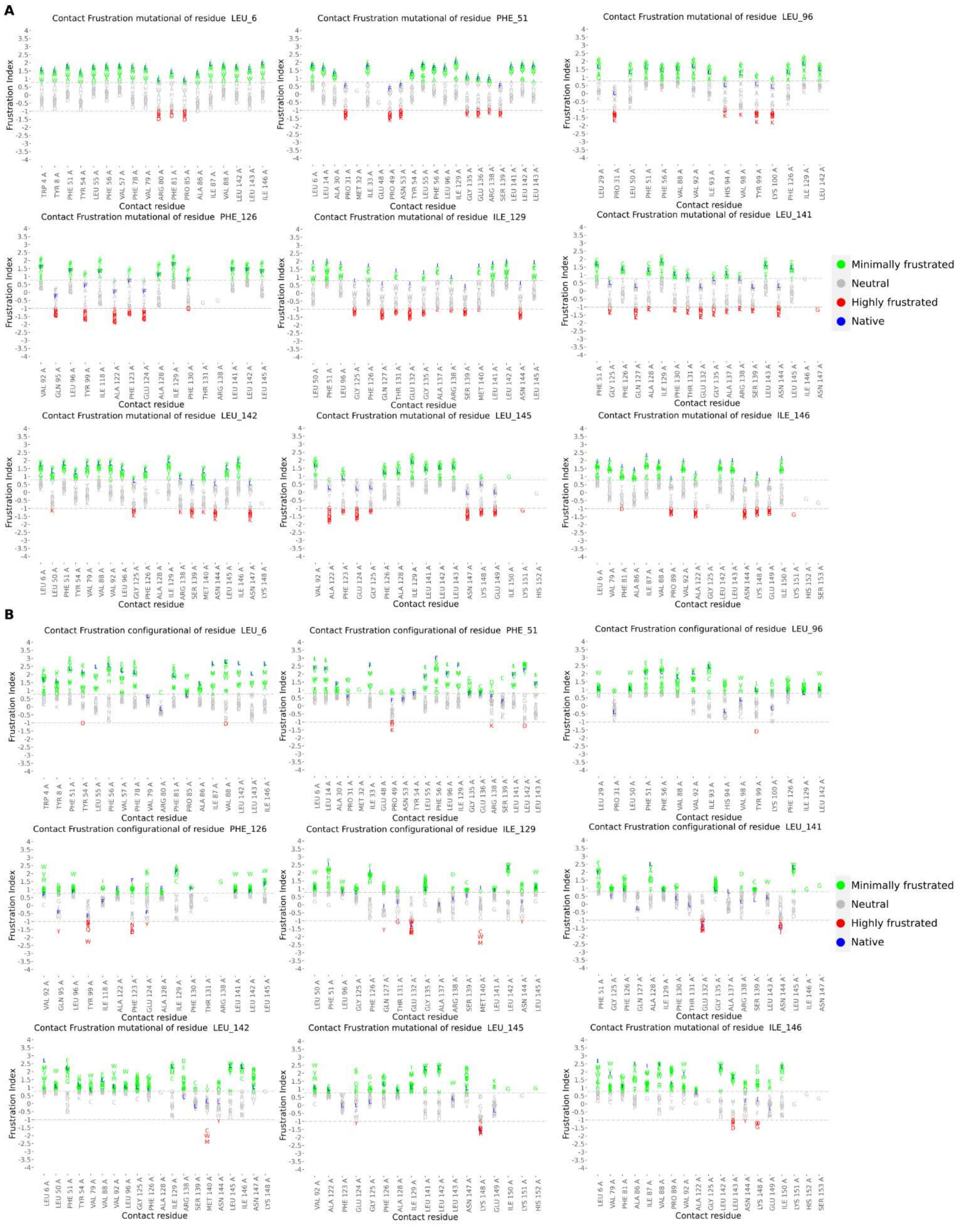

Supplementary Fig. 13. Contact frustration changes upon mutation, for mutational (A) and configurational (B) frustration index, of a specific residue for all canonical amino acids alternatives. X axis: all possible contacts that form the native protein and the mutants are shown. Y axis: frustration values, the canonical amino acids alternatives are represented in letters and coloured based to their frustration value. Native variant appears in blue.

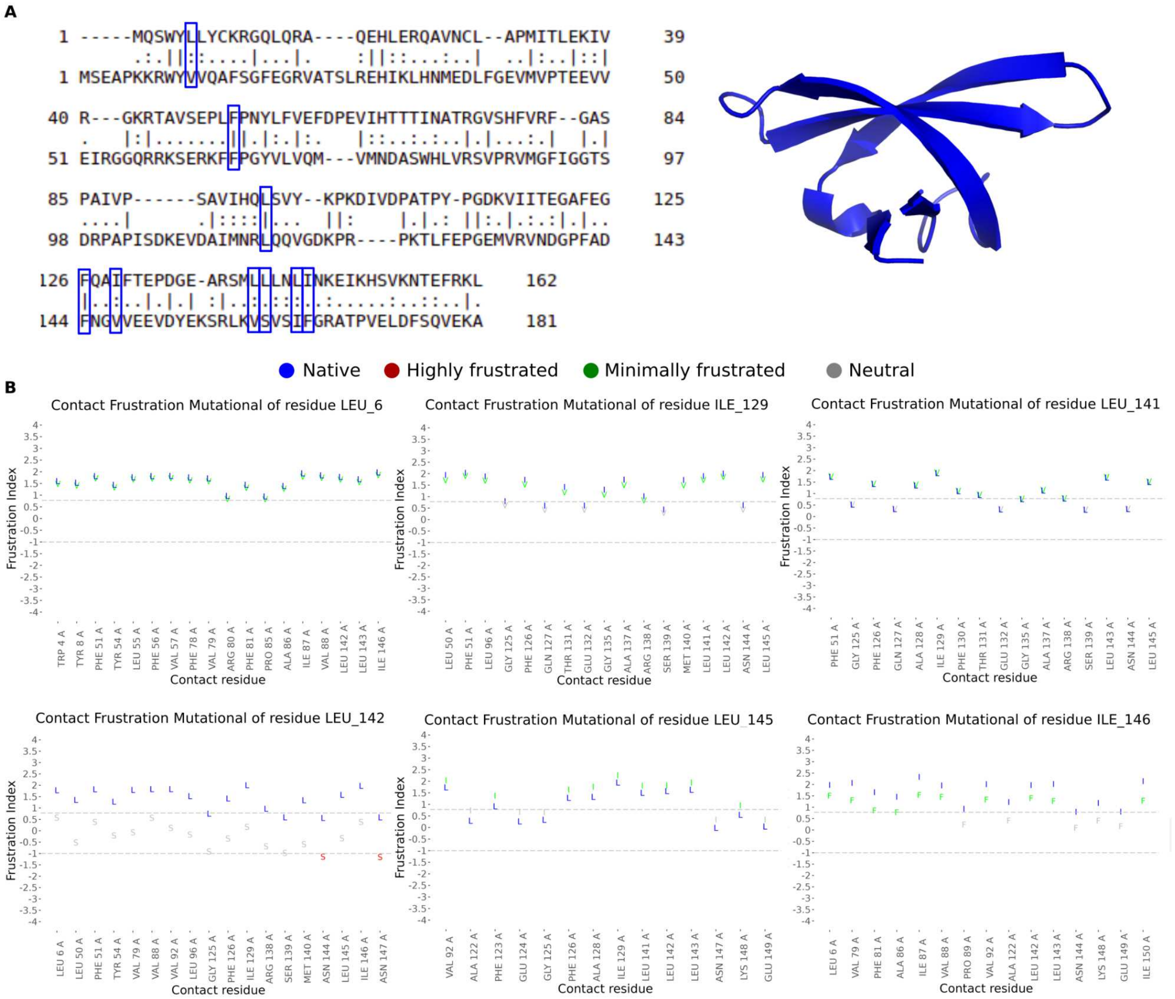

Supplementary Fig. 14. A) Sequence alignment between RFAH and NUSG, in the blue boxes are marked the 9 IR. B) Contact frustration changes for mutational index between native RFAH amino acids and NUSG equivalent positions. X axis: all possible contacts that form the native protein and the mutants are shown. Y axis: frustration values, the amino acids alternatives are represented in letters and coloured based to their frustration value. Native variant appears in blue.

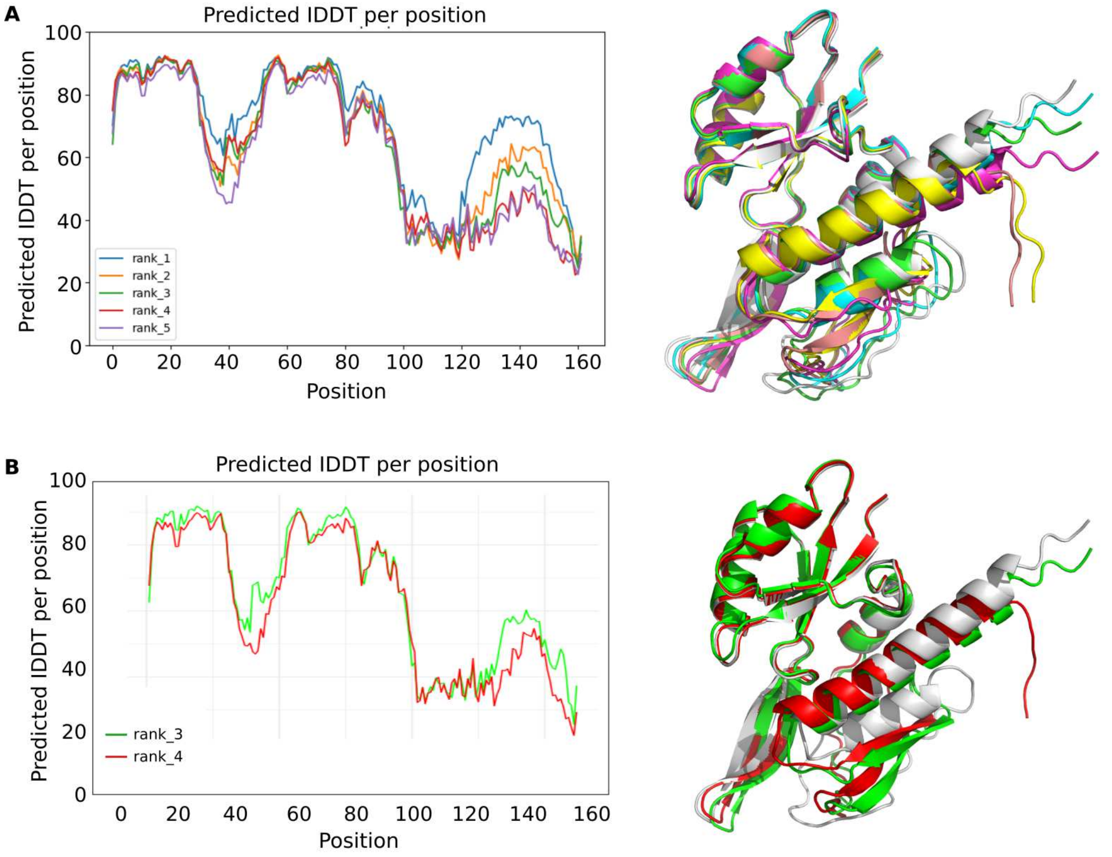

Supplementary Fig. 15. A) AlphaFold2 (AF2) models for RfaH containing 6 mutations with identities extracted from NusG. On the left a structural alignment between the 5 AF2 models of the NusG mutants and the native of RfaH from E. Coli is shown. In blue, orange, green, red and magenta are represented the NusG mutants and in white the RfaH from E. Coli. On the right the Predicted lDDT values per position for all NusG mutants of the AF2 models are shown. B) AF2 models for the RfaH sequence containing the L142S mutation. On the left are shown an structural alignment between 2 models of AF2 (Rank 3 and Rank 4) and the native of RfaH from E. Coli. In green and red the AF2 models and in white the native of RfaH from E. Coli. On the right the Predicted lDDT values per position for L142S mutants of the AF2 models are shown.

Supplementary Table 1. Frustration conservation state of relevant functional residues in hemoglobins.

Residue	FrustIC Score	Function
Lys7α	1.02	Forms a salt bridge with Asp74α (conserved and minimally frustrated) (Shaannan, 1983)
Glu27α	0.7	Forms a salt bridge with His112α (not energetically conserved) (Shaannan, 1983)
Glu30α	0.7	Forms a salt bridge with His50α (not energetically conserved) (Shaannan, 1983)
Lys40α	1.29	Lys40α is important for the protein-protein interactions in the α1-β2 pair, making a salt bridge with His146 in the β2 chain (His146β2), which in turns establishes an intra chain salt bridge with Asp94β2 (Imai et al. 1989). The salt bridges involving Lys40α and Asp94β2 play a key role in cooperative oxygen binding by hemoglobin.
Tyr42α	1.29	Participates in one of the strongest interactions in the α1-β2 interface, establishing a hydrogen bond with Asp99β2. Absence of this bond is actually observed in an abnormal hemoglobin, and shifts the allosteric equilibrium towards the oxy form, but it does not inhibit formation of the deoxy structure. In their absence the quaternary deoxy structure does not form and all cooperative effects are inhibited (Kavanaugh et al., 2005). Tyr42α is also one of the amino acids that interacts with the heme group in Human hemoglobin (Kavanaugh et al., 2005)..
Lys99α	0.84	Form salt bridge with Arg141α. Lys99α leads to anemias when mutated by amino acids that disrupt the protein-protein interactions with AHSP (Mollan et al. 2010).
Ser124α	1.02	When mutated by a Proline (HB Policoro) can lead to a disrupted interaction with AHSP due to conformation changes of the interacting helix in α-globin, leading to α-thalassemia (Bisconte et al. 2015).
Asp126α	0.57	Has been reported to play a role in the α1/α2 interface stabilization and has been found in Hb Sassari (Asp->His mutation) leading to a higher oxygen affinity possibly due to perturbations in the equilibrium between the two quaternary states (Sanna et al. 1994).
Lys127α	1.29	Mutation of the neighboring Lys127α can also lead to increased oxygen binding molecules as it happens with the naturally occurring variant Hb Waikato (Lys->Gln mutation) (Moore et al. 2021).
Tyr140α	0.51	Hb Rouen (increased oxygen affinity and decreased cooperativity, no described symptoms). Tyr140, is involved in the allosteric transition of Hb, which occurs when both α-heme groups become oxygenated tyr140 is expelled from the tyrosine pocket, leading to the rupture of 4 salt bridges that restrict the T-state associated with αArg141 and, therefore, the Hb α1/α2 interface. (Perutz, 1970).
Trp37β	0.59	Mutations in this residue have been shown to produce changes in the tertiary structure of hemoglobin, due to the disruption of intersubunit contacts associated with Trp37 that generate a change in the quaternary structure at the α1β2 interface (Kavanaugh, 1998)
Asn57β	1.29	It was reported as part of a hemoglobin variant known as HB G-Ferrara. It shows an increased of the oxygen affinity observed at acid pH values, may be related to an enhanced dissociation of the molecule into dimers (Giardina et al. 1978)
Glu101β	1.02	Interface with α-globin in human (Fermi 1975) (Shannan, 1983)
Asn108β	0.7	Interface with α-globin in human (Fermi 1975) (Shannan, 1983)
Tyr145β	0.68	It has been shown that this residue may be important in electron transfer pathways from reducing agents to the heme of Hb (Puscas, 2018).

Shaanan, B. (1983). Structure of human oxyhaemoglobin at 2· 1resolution. Journal of molecular biology, 171(1), 31-59.

Imai, K., Tsuneshige, A., Harano, T., & Harano, K. (1989). Structure-Function Relationships in Hemoglobin Kariya, Lys-40 (C5) α→ Glu, with High Oxygen Affinity: Functional role of the salt bridge between lys-40α and the β chain cooh terminus. Journal of Biological Chemistry, 264(19), 11174-11180.

Kavanaugh, J. S., Rogers, P. H., Arnone, A., Hui, H. L., Wierzba, A., DeYoung, A., ... & Friedman, J. M. (2005). Intersubunit interactions associated with Tyr42α stabilize the quaternary-T tetramer but are not major quaternary constraints in deoxyhemoglobin. Biochemistry, 44(10), 3806-3820.

Kavanaugh, J. S., Weydert, J. A., Rogers, P. H., & Arnone, A. (1998). High-resolution crystal structures of human hemoglobin with mutations at tryptophan 37β: structural basis for a high-affinity T-state. Biochemistry, 37(13), 4358-4373.

Mollan, T. L., Yu, X., Weiss, M. J., & Olson, J. S. (2010). The role of alpha-hemoglobin stabilizing protein in redox chemistry, denaturation, and hemoglobin assembly. Antioxidants & redox signaling, 12(2), 219-231.

Bisconte, M. G., Caldora, M., Musollino, G., Cardiero, G., Flagiello, A., La Porta, G., ... & Lacerra, G. (2015). α-Thalassemia associated with hb instability: a tale of two features. the case of Hb Rogliano or α1 Cod 108 (G15) Thr→ Asn and Hb Policoro or α2 Cod 124 (H7) Ser→ Pro. Plos one, 10(3), e0115738.

Sanna, M. T., Giardina, B., Scatena, R., Pellegrini, M., Olianas, A., Manca, L., ... & Corda, M. (1994). Functional alterations in adult and fetal hemoglobin Sassari Asp-alpha 126 (H9)–> His. The role of alpha 1 alpha 2 contact. Journal of Biological Chemistry, 269(28), 18338-18342.

Moore, J. A., Pullon, B. M., Wang, D., & Brennan, S. O. (2021). Hb Waikato [α127 (H10) Lys→ Gln; HBA1: c. 382A> C]: A Novel High Oxygen Affinity Variant. Hemoglobin, 45(1), 41-45.

Giardina, B., Brunori, M., Antonini, E., & Tentori, L. (1978). Properties of hemoglobin G. Ferrara (β57 (E1) Asn→ Lys). Biochimica et Biophysica Acta (BBA)-Protein Structure, 534(1), 1-6.

Fermi, G. (1975). Three-dimensional Fourier synthesis of human deoxyhaemoglobin at 2· 5 Å resolution: refinement of the atomic model. Journal of Molecular Biology, 97(2), 237-256.

Puscas, C., Radu, L., Carrascoza, F., Mot, A. C., Amariei, D., Lungu, O., ... & Silaghi-Dumitrescu, R. (2018). The high affinity of small-molecule antioxidants for hemoglobin. Free Radical Biology and Medicine, 124, 260-274.

Perutz, M. F. (1970). Stereochemistry of cooperative effects in haemoglobin: haem–haem interaction and the problem of allostery. Nature, 228(5273), 726-734.

Supplementary Table 2. Characteristics of the Coronavirus protein families considered in section 5. SARS-CoV-2 proteins that did not have an experimental PDB structure (in red) were discarded from the analysis.

Protein	SARS2 seq length	MSA length	MSA depth	# S3Det clusters	# SDPs	SARS-CoV-2 PDB
nsp1	180	185	24	2	4	7K7P
nsp2	638	699	61	3	51	7MSW
nsp3	1945	3426	124	4	48	7PKU, 7LGO, 6XA9, 7RKG, 6Z5T, 7THH
nsp4	500	625	328	6	5	-
nsp5	306	371	244	5	9	7CBT
nsp6	290	374	190	3	11	-
nsp7	83	89	205	4	3	7BV1
nsp8	198	232	198	6	1	7OZV
nsp9	113	118	166	4	1	7KRI
nsp10	139	153	221	5	1	7C2J
nsp11	13	-	-	-	-	-
nsp12	932	1131	195	2	27	7DTE
nsp13	601	809	127	2	2	7KRN
nsp14	527	591	179	6	10	7EIZ
nsp15	346	423	286	7	2	7N06
nsp16	298	497	257	5	6	7C2J
S	1273	1998	193	5	34	7A92
orf3a	275	287	30	2	8	7KJR
orf3b	57	-	-	-	-	-
E	75	105	58	3	3	7K3G
M	222	485	503	6	2	-
orf6	61	90	24	5	0	-
orf7a	121	137	26	4	0	7CI3
orf7b	43	55	15	3	0	-
N	419	780	547	6	3	7N01, 7ACT
orf8	121	135	19	2	9	7F5F
orf9b	97	98	21	3	4	7KDT
orf9c	73	73	12	2	0	-
orf10	38	38	3	0	0	-

Supplementary Table 3. Summary of AlphaFold2 modeling results for the Coronavirus protein families considered in section 5. Sequences containing 'X' characters or that are were too short failed to be modelled and are shown in red.

Protein or protein domain	# S3Det cluster	# Seqs	PDB_code	Trimmed ref seq length	Trimmed MSA length	Mean pLDDT	Not modelled	ID protein not modelled	Mean FrustIC	SD FrustIC	Mean SeqIC	SD SeqIC
nsp1	1	10				68.38	0	-				
nsp1	2	13	7k7p	116	121	80.93	0	-	0.930	0.383	3.091	0.401
nsp2	1	30				82.62	0	-	0.854	0.442	3.383	0.578
nsp2	2	28				74.74	0	-				
nsp2	3	2	7msw	501	541	54.45	0	-				
nsp3_Ubl1	1	31				85.13	0	-	0.904	0.419	3.441	0.494
nsp3_Ubl1	2	11				79.68	0	-				
nsp3_Ubl1	3	35				82.19	0	-	0.692	0.510	3.083	0.742
nsp3_Ubl1	4	47	7pku	110	125	81.38	0	-	0.681	0.497	2.903	0.808
nsp3_MacroX	1	31				94.79	0	-	0.914	0.422	3.295	0.659
nsp3_MacroX	2	11				92.92	0	-	0.768	0.484	2.401	0.770
nsp3_MacroX	3	35				78.35	1	AIZ74404.1				
nsp3_MacroX	4	47	6z5t	171	321	82.85	0	-	0.777	0.498	3.398	0.708
nsp3_MacroII	1	31				57.75	0	-				
nsp3_MacroII	2	11				64.11	0	-				
nsp3_MacroII	3	35				-	35	All				
nsp3_MacroII	4	47	model	127	163	-	47	All				
nsp3_SUD_Nterm	1	31				83.49	0	-	0.881	0.426	3.328	0.536
nsp3_SUD_Nterm	2	11				71.88	0	-				
nsp3_SUD_Nterm	3	35				91.66	0	-	0.768	0.475	2.966	0.805
nsp3_SUD_Nterm	4	47	model	263	307	58.17	0	-				
nsp3_SUD_Cterm	1	31				88.81	0	-	0.683	0.366	3.290	0.593
nsp3_SUD_Cterm	2	11				93.13	0	-	0.758	0.429	2.407	0.648
nsp3_SUD_Cterm	3	35				95.71	0	-	0.754	0.515	2.889	0.821
nsp3_SUD_Cterm	4	47	7thh	65	87	83.01	0	-	0.285	0.331	2.983	0.856
nsp3_PLPro	1	31				94.69	0	-	0.990	0.362	3.500	0.460
nsp3_PLPro	2	11				95.06	0	-	0.773	0.440	2.472	0.664
nsp3_PLPro	3	35				93.86	1	AIZ74404.1	0.769	0.476	3.129	0.754
nsp3_PLPro	4	47	6xa9	308	333	92.63	2	AWB14623.1, AFG25759.1	0.778	0.479	3.182	0.776
nsp3_NAB	1	31				88.57	3	ACU31042.1, AAR86774.1, ANA96026.1	0.915	0.422	3.426	0.450
nsp3_NAB	2	11				66.2	0	-				
nsp3_NAB	3	35				78.46	1	QLD98030.1	0.666	0.444	3.126	0.760
nsp3_NAB	4	47	7lgo	112	125	64.57	1	AFG25769.1	0.730	0.466	3.452	0.725
nsp3_Y3	1	31				90.3	0	-	1.074	0.339	3.637	0.320
nsp3_Y3	2	11	7rqg			92.94	0	-	0.876	0.406	2.701	0.460
nsp3_Y3	3	35				88.66	1	QLD98030.1	0.895	0.447	3.411	0.615
nsp3_Y3	4	47		101	104	92.92	0	-	0.997	0.431	3.617	0.537
nsp5	1	32				90.63	0	-	0.710	0.483	2.901	0.822
nsp5	2	19				91.25	0	-	0.942	0.430	3.305	0.502
nsp5	3	76				92.8	0	-	0.743	0.479	3.147	0.874
nsp5	4	98				93.04	0	-	1.044	0.366	3.892	0.450
nsp5	5	18	7cbt	304	347	94.06	0	-	0.878	0.412	2.996	0.623
nsp7	1	36				87.77	0	-	0.843	0.471	3.273	0.643
nsp7	2	30				84.84	1	QEG03810.1	0.995	0.387	3.635	0.349
nsp7	3	82				87.99	0	-	0.783	0.386	3.370	0.692
nsp7	4	56	7bv1	71	77	87.86	0	-	1.040	0.329	3.843	0.308
nsp8	1	15				88.88	0	-	0.638	0.443	2.498	0.649
nsp8	2	18				89.33	0	-	0.952	0.402	3.254	0.496
nsp8	3	68				90.22	0	-	0.704	0.455	3.176	0.755
nsp8	4	23				87.99	0	-	1.047	0.362	3.490	0.423
nsp8	5	58				86.96	0	-	1.054	0.340	3.862	0.376
nsp8	6	16	7ozv	186	220	88.18	0	-	0.829	0.416	2.934	0.572
nsp9	1	46				88.27	0	-	0.658	0.478	2.935	0.795
nsp9	2	78				87.36	0	-	0.723	0.438	3.274	0.806
nsp9	3	24				88.81	0	-	0.966	0.352	3.443	0.308
nsp9	4	18	7kri	112	117	87.41	0	-	0.758	0.428	3.011	0.640
nsp10	1	40				90.44	0	-	0.779	0.489	3.227	0.764
nsp10	2	21				90.84	0	-	1.013	0.376	3.404	0.518
nsp10	3	71				88.43	0	-	1.059	0.331	3.906	0.327
nsp10	4	74				91.7	0	-	0.829	0.458	3.426	0.773
nsp10	5	15	7c2j	121	129	90	0	-	0.938	0.404	2.996	0.603
nsp12	1	43				90.18	1	QLD97932.1	0.835	0.475	3.349	0.750
nsp12	2	69				89.76	0	-	1.102	0.310	3.945	0.292
nsp12	3	53				89.02	0	-	0.878	0.447	3.472	0.720
nsp12	4	14	7dte	925	1007	90.32	0	-	0.921	0.424	2.947	0.616
nsp13	1	21				92.31	0	-	0.960	0.436	3.194	0.648
nsp13	2	13				91.93	0	-	1.069	0.341	3.117	0.340
nsp13	3	49				91.43	0	-	0.934	0.432	3.438	0.715
nsp13	4	20				90.74	0	-	1.058	0.319	3.421	0.304
nsp13	5	14	7krn	591	604	92.3	0	-	1.021	0.356	3.074	0.427
nsp14	1	17				88.31	0	-	0.766	0.477	2.835	0.691
nsp14	2	14				89.2	0	-	0.999	0.393	3.123	0.456
nsp14	3	22				90.27	0	-	0.988	0.404	3.401	0.482
nsp14	4	50				89.24	0	-	1.103	0.308	3.886	0.279
nsp14	5	62				90.56	1	QEG03729.1	0.805	0.465	3.327	0.820
nsp14	6	13	7eiz	521	554	90.32	0	-	0.878	0.419	2.796	0.602
nsp15	1	24				94.64	0	-	1.109	0.320	3.562	0.376
nsp15	2	12				93.94	0	-	0.657	0.464	2.176	0.688
nsp15	3	28				89.14	0	-	0.889	0.465	3.360	0.620
nsp15	4	24				93.53	0	-	0.980	0.424	3.327	0.606
nsp15	5	80				92.45	1	QEG03729.1	0.770	0.470	3.280	0.816
nsp15	6	99				94.4	0	-	1.114	0.320	3.991	0.333
nsp15	7	18	7n06	344	421	94.64	0	-	0.887	0.411	2.922	0.658
nsp16	1	45				91.56	0	-	0.864	0.471	3.269	0.778
nsp16	2	23				90.18	0	-	0.969	0.422	3.430	0.474
nsp16	3	71				90.89	0	-	0.862	0.465	3.408	0.738
nsp16	4	100				88.57	0	-	1.058	0.372	3.924	0.427
nsp16	5	17	7c2j	296	319	92.64	0	-	0.912	0.406	3.063	0.546
S_protein	1	51				86.12	2	camel_Abu_Dhabi_B101, hCoV-OC43_USA_TCNP_00204_2017	0.885	0.444	3.380	0.737
S_protein	2	35				83.26	2	camel_UAE_415915_W4_2015, BtCoV_KW2E-F93_Nyc_spec_GHA_2010	0.850	0.461	3.153	0.784
S_protein	3	16				78.48	1	Bat-CoV_CMR705-P13				
S_protein	4	29				82.71	0	-	0.951	0.436	3.340	0.744
S_protein	5	2	7k3g	1148	1539	72.97	0	-				

E	1	27				95.17	0	-	1.016	0.275	3.561	0.262
E	2	28				95.74	0	-	0.951	0.402	3.355	0.574
E	3	2	7n0i	30	30	96.48	0	-	0.467	0.384	2.000	0.000
N_Nterm	1	64				92.54	0	-	0.557	0.398	2.980	0.773
N_Nterm	2	50				95.12	0	-	0.929	0.431	3.665	0.608
N_Nterm	3	259				91.31	0	-	0.966	0.375	3.970	0.386
N_Nterm	4	94				95.6	0	-	0.830	0.405	3.685	0.570
N_Nterm	5	57				94.23	0	-	0.492	0.431	2.708	0.802
N_Nterm	6	21	7act	117	185	93.48	0	-	0.567	0.422	2.980	0.680
N_Cterm	1	64				87.35	0	-	0.609	0.489	3.041	0.840
N_Cterm	2	50				84.73	0	-	1.036	0.385	3.560	0.674
N_Cterm	3	259				84.36	1	ADP06474.1	1.017	0.338	4.011	0.391
N_Cterm	4	94				84.39	0	-	1.024	0.369	3.745	0.506
N_Cterm	5	57				83.58	0	-	0.613	0.471	2.925	0.803
N_Cterm	6	21	7a92	124	157	74.06	0	-				
orf3a	1	8				60.8	0	-				
orf3a	2	21	7kjr	198	204	59.93	0	-				
orf7a	1	21				63.16	0	-				
orf7a	2	2				83.54	0	-	0.422	0.415	2.000	0.000
orf7a	3	1				90.94	0	-	-0.082	0.000	2.000	0.000
orf7a	4	2	7ci3	68	74	90	0	-	0.422	0.415	2.000	0.000
orf8	1	12				71.57	0	-				
orf8	2	7	7f5f	103	112	91.98	0	-	0.763	0.486	2.012	0.495
orf9b	1	3				80.12	0	-				
orf9b	2	17				75.18	0	-				
orf9b	3	1	7kdt	37	37	58.92	0	-				
	Total number of models						4395					

Supplementary Table 4. Frustration states of SARS-CoV-2 proteins close orthologs (same S3Det cluster), computed with FrustraEvo, and SARS-CoV-2 individual frustration score, computed with FrustratometeR. FrustIC represents the conservation of frustration across the cluster and SRFI is the single residue frustration index of the SARS-CoV-2 sequence. The first column containin amino acids letters corresponds to the consensus amino acid based on the MSA of the cluster while 'SARS2 AA' represents the amino acid appearing in the SARS-CoV-2 protein.

Protein	SARS2 containing cluster	Protein position	MSA consensus AA	FrustIC	Frustraevo state	SARS2 AA	SRFI	Frustratometer state
nsp2	1	1	R	0.395	MIN	R	0.607	NEU
nsp2	1	2	Y	0.391	NEU	Y	-0.327	NEU
nsp2	1	3	V	**1.313**	MIN	V	1.36	MIN
nsp2	1	4	D	0.316	MAX	D	-1.097	MAX
nsp2	1	5	N	**1.313**	MAX	N	-1.071	MAX
nsp2	1	6	N	**1.102**	NEU	N	-0.89	NEU
nsp2	1	7	F	**0.960**	MIN	F	1.235	MIN
nsp2	1	8	C	**1.313**	MIN	C	2.602	MIN
nsp2	1	9	G	**1.313**	NEU	G	0.015	NEU
nsp2	1	10	P	**1.313**	NEU	P	-0.541	NEU
nsp2	1	11	D	0.476	NEU	D	-1.25	MAX
nsp2	1	12	G	**1.313**	NEU	G	-0.082	NEU
nsp2	1	13	Y	**1.102**	MAX	Y	-0.892	NEU
nsp2	1	14	P	**1.102**	MAX	P	-1.176	MAX
nsp2	1	15	L	**1.313**	MIN	L	0.774	NEU
nsp2	1	16	E	**0.529**	NEU	E	-0.603	NEU
nsp2	1	17	C	**1.313**	MIN	C	2.89	MIN
nsp2	1	18	I	**1.313**	MIN	I	1.885	MIN
nsp2	1	19	K	**0.960**	NEU	K	-0.721	NEU
nsp2	1	20	D	**1.102**	NEU	D	-0.518	NEU
nsp2	1	21	L	**0.663**	MIN	L	0.949	MIN
nsp2	1	22	L	**0.960**	MIN	L	0.908	MIN
nsp2	1	23	A	**1.313**	NEU	A	0.44	NEU
nsp2	1	24	R	**0.960**	MIN	R	0.701	NEU
nsp2	1	25	A	**0.591**	NEU	A	0.538	NEU
nsp2	1	26	G	**1.313**	NEU	G	-0.81	NEU
nsp2	1	27	K	0.316	NEU	K	-0.976	NEU
nsp2	1	28	S	**1.313**	NEU	A	0.425	NEU
nsp2	1	29	M	-0.060	MAX	S	0.59	NEU
nsp2	1	30	C	**0.844**	MIN	C	1.875	MIN
nsp2	1	31	T	**0.638**	MIN	T	1.268	MIN
nsp2	1	32	L	**0.591**	NEU	L	0.604	NEU
nsp2	1	33	S	**1.102**	NEU	S	-0.168	NEU
nsp2	1	34	E	0.432	NEU	E	-0.653	NEU
nsp2	1	35	Q	**0.663**	NEU	Q	-1.009	MAX
nsp2	1	36	L	**1.313**	MIN	L	0.838	MIN
nsp2	1	37	D	**1.102**	NEU	D	0.179	NEU
nsp2	1	38	F	0.316	MAX	F	-0.41	NEU
nsp2	1	39	I	**1.313**	MIN	I	1.255	MIN

nsp2	1	40	E	**0.663**	NEU	D	0.289	NEU
nsp2	1	41	S	**1.313**	NEU	T	-0.663	NEU
nsp2	1	42	K	0.326	MAX	K	-1.188	MAX
nsp2	1	43	R	0.326	NEU	R	1.297	MIN
nsp2	1	44	G	**1.313**	NEU	G	-0.462	NEU
nsp2	1	45	V	-0.170	MAX	V	-1.087	MAX
nsp2	1	46	Y	**1.102**	MAX	Y	-2.324	MAX
nsp2	1	47	C	**0.746**	MIN	C	1.704	MIN
nsp2	1	48	C	**1.313**	MIN	C	2.088	MIN
nsp2	1	49	R	0.313	MIN	R	0.589	NEU
nsp2	1	50	E	**0.663**	MAX	E	-1.459	MAX
nsp2	1	51	H	**1.313**	NEU	H	-0.45	NEU
nsp2	1	52	E	0.331	NEU	E	-0.591	NEU
nsp2	1	53	H	**1.313**	NEU	H	-0.161	NEU
nsp2	1	54	E	**0.663**	NEU	E	-0.961	NEU
nsp2	1	55	I	**1.313**	MIN	I	1.027	MIN
nsp2	1	56	A	**1.102**	MIN	A	0.601	NEU
nsp2	1	57	W	**1.313**	MIN	W	0.833	MIN
nsp2	1	58	F	0.222	MIN	Y	0.665	NEU
nsp2	1	59	T	**1.313**	NEU	T	-0.251	NEU
nsp2	1	60	E	**0.752**	NEU	E	-0.64	NEU
nsp2	1	61	R	**0.844**	NEU	R	-0.265	NEU
nsp2	1	62	S	**0.960**	NEU	S	-0.49	NEU
nsp2	1	63	D	**0.844**	NEU	E	-0.791	NEU
nsp2	1	64	K	**0.663**	NEU	K	-0.703	NEU
nsp2	1	65	S	**1.313**	NEU	S	-0.483	NEU
nsp2	1	66	Y	**0.844**	MIN	Y	0.635	NEU
nsp2	1	67	E	**1.313**	NEU	E	-0.682	NEU
nsp2	1	68	H	**0.844**	MIN	L	1.358	MIN
nsp2	1	69	Q	0.432	MAX	Q	-1.066	MAX
nsp2	1	70	T	**1.102**	NEU	T	-0.396	NEU
nsp2	1	71	P	**0.529**	NEU	P	-1.029	MAX
nsp2	1	72	F	**1.313**	MIN	F	0.769	NEU
nsp2	1	73	E	0.326	NEU	E	0.396	NEU
nsp2	1	74	I	**1.313**	MIN	I	1.164	MIN
nsp2	1	75	K	**0.591**	NEU	K	0.408	NEU
nsp2	1	76	S	0.476	NEU	L	1.293	MIN
nsp2	1	77	A	**1.313**	MIN	A	0.8	MIN
nsp2	1	78	K	0.365	MAX	K	-1.236	MAX
nsp2	1	79	K	**1.102**	NEU	K	-0.82	NEU
nsp2	1	80	F	**1.313**	MIN	F	1.086	MIN
nsp2	1	81	D	0.313	MIN	D	0.929	MIN
nsp2	1	82	T	**1.313**	NEU	T	-0.353	NEU
nsp2	1	83	F	**0.844**	MIN	F	1.066	MIN
nsp2	1	84	K	**0.663**	MIN	N	0.735	NEU
nsp2	1	85	G	**0.844**	NEU	G	-0.788	NEU

nsp2	1	86	E	**0.529**	NEU	E	-0.116	NEU
nsp2	1	87	C	**1.313**	MIN	C	1.276	MIN
nsp2	1	88	P	**1.313**	NEU	P	-0.177	NEU
nsp2	1	89	K	-0.120	NEU	N	-0.95	NEU
nsp2	1	90	F	**1.313**	MIN	F	1.11	MIN
nsp2	1	91	V	**1.313**	MIN	V	1.3	MIN
nsp2	1	92	F	**1.313**	MIN	F	1.303	MIN
nsp2	1	93	P	**1.313**	MAX	P	-1.238	MAX
nsp2	1	94	L	**1.313**	MIN	L	1.104	MIN
nsp2	1	95	N	**1.102**	NEU	N	-0.801	NEU
nsp2	1	96	S	**1.313**	NEU	S	-0.676	NEU
nsp2	1	97	K	0.395	NEU	I	1.505	MIN
nsp2	1	98	V	**1.313**	MIN	I	1.213	MIN
nsp2	1	99	K	**0.752**	NEU	K	-0.989	NEU
nsp2	1	100	V	**0.529**	MIN	T	-0.409	NEU
nsp2	1	101	I	**1.313**	MIN	I	1.302	MIN
nsp2	1	102	Q	0.313	NEU	Q	0.062	NEU
nsp2	1	103	P	**1.102**	NEU	P	-0.509	NEU
nsp2	1	104	R	**0.591**	NEU	R	0.21	NEU
nsp2	1	105	V	**0.960**	NEU	V	-0.775	NEU
nsp2	1	106	E	**1.102**	MIN	E	1.779	MIN
nsp2	1	107	K	**1.102**	NEU	K	-0.156	NEU
nsp2	1	108	K	**0.663**	MIN	K	0.24	NEU
nsp2	1	109	K	**1.102**	MIN	K	1.707	MIN
nsp2	1	110	T	0.395	NEU	L	0.593	NEU
nsp2	1	111	E	**1.313**	MIN	D	1.343	MIN
nsp2	1	112	G	**1.313**	NEU	G	-0.022	NEU
nsp2	1	113	F	**1.313**	MIN	F	1.053	MIN
nsp2	1	114	M	**1.313**	MIN	M	0.876	MIN
nsp2	1	115	G	**0.844**	NEU	G	0.582	NEU
nsp2	1	116	R	**0.960**	NEU	R	-0.291	NEU
nsp2	1	117	I	**1.313**	MIN	I	1.676	MIN
nsp2	1	118	R	**1.313**	NEU	R	-0.116	NEU
nsp2	1	119	S	**1.313**	NEU	S	-0.177	NEU
nsp2	1	120	V	**1.313**	MIN	V	1.791	MIN
nsp2	1	121	Y	**1.313**	NEU	Y	-0.665	NEU
nsp2	1	122	P	0.326	NEU	P	0.554	NEU
nsp2	1	123	V	**1.313**	MIN	V	1.553	MIN
nsp2	1	124	A	**1.102**	MAX	A	-1.055	MAX
nsp2	1	125	S	**0.960**	NEU	S	-0.317	NEU
nsp2	1	126	P	**1.313**	NEU	P	-0.408	NEU
nsp2	1	127	Q	0.299	NEU	N	-0.492	NEU
nsp2	1	128	E	**0.746**	NEU	E	-0.393	NEU
nsp2	1	129	C	**1.313**	MIN	C	2.353	MIN
nsp2	1	130	N	**0.960**	NEU	N	-0.582	NEU
nsp2	1	131	D	0.299	NEU	Q	0.499	NEU

nsp2	1	132	M	**0.746**	MIN	M	0.919	MIN
nsp2	1	133	H	**0.663**	NEU	C	2.073	MIN
nsp2	1	134	L	**1.313**	MIN	L	1.088	MIN
nsp2	1	135	S	**1.313**	NEU	S	-0.49	NEU
nsp2	1	136	T	**0.746**	NEU	T	0.077	NEU
nsp2	1	137	L	**0.960**	MIN	L	1.049	MIN
nsp2	1	138	M	-0.162	MIN	M	-0.572	NEU
nsp2	1	139	K	**0.844**	MIN	K	1.055	MIN
nsp2	1	140	C	**1.313**	MIN	C	2.44	MIN
nsp2	1	141	N	0.395	NEU	D	2.281	MIN
nsp2	1	142	H	**1.313**	MIN	H	1.162	MIN
nsp2	1	143	C	**0.591**	MIN	C	1.108	MIN
nsp2	1	144	D	0.313	MIN	G	-0.772	NEU
nsp2	1	145	E	0.395	MAX	E	-1.341	MAX
nsp2	1	146	V	0.204	MIN	T	0.428	NEU
nsp2	1	147	S	**1.313**	NEU	S	0.042	NEU
nsp2	1	148	W	0.017	MIN	W	0.541	NEU
nsp2	1	149	Q	0.238	NEU	Q	-0.236	NEU
nsp2	1	150	T	**1.313**	NEU	T	-0.339	NEU
nsp2	1	151	C	0.432	MIN	G	-0.695	NEU
nsp2	1	152	D	0.432	NEU	D	-1.264	MAX
nsp2	1	153	F	**1.313**	MIN	F	1.178	MIN
nsp2	1	154	L	0.476	MIN	V	1.659	MIN
nsp2	1	155	K	**1.102**	NEU	K	-0.716	NEU
nsp2	1	156	A	**1.313**	NEU	A	-0.244	NEU
nsp2	1	157	T	**1.313**	MIN	T	1.028	MIN
nsp2	1	158	C	**0.960**	MIN	C	1.015	MIN
nsp2	1	159	E	0.395	MAX	E	-0.467	NEU
nsp2	1	160	Q	-0.237	MIN	F	0.677	NEU
nsp2	1	161	C	**0.960**	MIN	C	0.48	NEU
nsp2	1	162	G	**0.960**	NEU	G	-1.13	MAX
nsp2	1	163	T	0.432	NEU	T	-0.999	NEU
nsp2	1	164	E	0.432	MIN	E	1.044	MIN
nsp2	1	165	N	**1.102**	NEU	N	-0.671	NEU
nsp2	1	166	L	0.313	MIN	L	0.032	NEU
nsp2	1	167	V	**0.591**	MIN	T	-0.157	NEU
nsp2	1	168	C	-0.105	MIN	K	-1.096	MAX
nsp2	1	169	E	0.071	NEU	E	0.938	MIN
nsp2	1	170	G	**1.102**	NEU	G	-0.056	NEU
nsp2	1	171	P	**0.663**	NEU	A	0.594	NEU
nsp2	1	172	T	**1.313**	NEU	T	-0.289	NEU
nsp2	1	173	T	**1.313**	NEU	T	-0.219	NEU
nsp2	1	174	C	**1.313**	MIN	C	1.621	MIN
nsp2	1	175	G	**1.313**	NEU	G	-0.254	NEU
nsp2	1	176	Y	0.476	NEU	Y	-0.173	NEU
nsp2	1	177	L	0.326	NEU	L	0.248	NEU

nsp2	1	178	P	0.395	NEU	P	-0.623	NEU
nsp2	1	179	T	0.154	NEU	Q	-0.06	NEU
nsp2	1	180	N	**0.960**	NEU	N	-0.93	NEU
nsp2	1	181	A	**0.844**	NEU	A	0.292	NEU
nsp2	1	182	V	**1.313**	MIN	V	1.175	MIN
nsp2	1	183	V	**1.102**	MIN	V	1.29	MIN
nsp2	1	184	K	**1.313**	MAX	K	-1.497	MAX
nsp2	1	185	M	0.342	MIN	I	1.196	MIN
nsp2	1	186	P	**0.591**	NEU	Y	-3.026	MAX
nsp2	1	187	C	**1.102**	MIN	C	1.897	MIN
nsp2	1	188	P	**0.529**	NEU	P	-0.908	NEU
nsp2	1	189	A	-0.012	NEU	A	-0.946	NEU
nsp2	1	190	C	**1.102**	MIN	C	1.351	MIN
nsp2	1	191	Q	**0.893**	NEU	H	-0.413	NEU
nsp2	1	192	D	**0.529**	MAX	N	-0.887	NEU
nsp2	1	193	P	**0.746**	MIN	S	-0.55	NEU
nsp2	1	194	E	0.407	NEU	E	-0.229	NEU
nsp2	1	195	I	0.136	NEU	V	0.278	NEU
nsp2	1	196	G	**1.313**	NEU	G	-0.668	NEU
nsp2	1	197	P	**0.663**	NEU	P	1.106	MIN
nsp2	1	198	E	**0.960**	MAX	E	-1.553	MAX
nsp2	1	199	H	**1.102**	NEU	H	-0.709	NEU
nsp2	1	200	S	**1.313**	NEU	S	-0.492	NEU
nsp2	1	201	V	0.136	NEU	L	0.695	NEU
nsp2	1	202	A	**1.313**	NEU	A	-0.227	NEU
nsp2	1	203	D	**0.529**	NEU	E	-1.559	MAX
nsp2	1	204	Y	**0.960**	NEU	Y	-0.307	NEU
nsp2	1	205	H	**1.313**	NEU	H	-0.653	NEU
nsp2	1	206	N	**1.313**	NEU	N	-0.637	NEU
nsp2	1	207	H	0.114	MIN	E	-1.758	MAX
nsp2	1	208	S	**1.313**	NEU	S	-0.355	NEU
nsp2	1	209	N	0.391	NEU	G	-0.353	NEU
nsp2	1	210	I	**1.313**	MIN	L	1.312	MIN
nsp2	1	211	E	**0.591**	MIN	K	-0.123	NEU
nsp2	1	212	T	**1.313**	NEU	T	-0.2	NEU
nsp2	1	213	R	**0.543**	MIN	I	1.521	MIN
nsp2	1	214	L	**1.313**	NEU	L	-0.182	NEU
nsp2	1	215	R	0.342	NEU	R	-0.432	NEU
nsp2	1	216	K	-0.257	MIN	K	0.083	NEU
nsp2	1	217	G	**1.313**	NEU	G	-0.003	NEU
nsp2	1	218	G	**1.313**	NEU	G	-0.82	NEU
nsp2	1	219	R	**1.313**	NEU	R	-0.774	NEU
nsp2	1	220	T	**0.844**	NEU	T	-0.084	NEU
nsp2	1	221	K	-0.106	MAX	I	1.304	MIN
nsp2	1	222	C	0.432	MIN	A	0.168	NEU
nsp2	1	223	F	**1.102**	MIN	F	1.372	MIN

nsp2	1	224	G	**1.313**	NEU	G	-0.243	NEU
nsp2	1	225	G	**1.313**	NEU	G	-0.355	NEU
nsp2	1	226	C	**1.313**	MIN	C	1.405	MIN
nsp2	1	227	V	**1.313**	MIN	V	1.219	MIN
nsp2	1	228	F	**1.313**	MIN	F	1.383	MIN
nsp2	1	229	A	**1.313**	NEU	S	-0.663	NEU
nsp2	1	230	Y	0.476	MAX	Y	-0.713	NEU
nsp2	1	231	V	**1.313**	MIN	V	1.542	MIN
nsp2	1	232	G	**1.313**	NEU	G	-0.342	NEU
nsp2	1	233	C	**1.313**	MIN	C	1.306	MIN
nsp2	1	234	Y	**0.893**	NEU	H	0.018	NEU
nsp2	1	235	N	**1.313**	NEU	N	-0.247	NEU
nsp2	1	236	K	**0.746**	MAX	K	-0.98	NEU
nsp2	1	237	R	0.476	NEU	C	1.647	MIN
nsp2	1	238	A	**1.313**	NEU	A	0.126	NEU
nsp2	1	239	Y	**0.746**	NEU	Y	0.446	NEU
nsp2	1	240	W	**0.844**	NEU	W	0.568	NEU
nsp2	1	241	V	**1.313**	MIN	V	1.317	MIN
nsp2	1	242	P	**1.313**	NEU	P	-0.882	NEU
nsp2	1	243	R	**1.313**	NEU	R	-0.551	NEU
nsp2	1	244	A	**1.313**	NEU	A	0.387	NEU
nsp2	1	245	S	**1.313**	NEU	S	-0.418	NEU
nsp2	1	246	A	**1.313**	NEU	A	0.274	NEU
nsp2	1	247	N	**1.313**	NEU	N	-0.697	NEU
nsp2	1	248	I	**1.313**	MIN	I	1.014	MIN
nsp2	1	249	G	**0.663**	NEU	G	-0.957	NEU
nsp2	1	250	A	0.476	NEU	C	2.014	MIN
nsp2	1	251	N	**1.313**	NEU	N	-0.711	NEU
nsp2	1	252	H	**1.313**	NEU	H	-0.413	NEU
nsp2	1	253	T	**1.313**	NEU	T	-0.406	NEU
nsp2	1	254	G	**1.313**	NEU	G	-0.342	NEU
nsp2	1	255	I	**1.313**	MIN	V	0.962	MIN
nsp2	1	256	T	0.395	NEU	V	1.176	MIN
nsp2	1	257	G	**1.102**	NEU	G	-0.016	NEU
nsp2	1	258	D	0.061	MIN	E	-1.542	MAX
nsp2	1	259	N	**1.313**	NEU	G	-0.479	NEU
nsp2	1	260	V	0.476	MIN	S	-0.611	NEU
nsp2	1	261	E	**0.529**	MAX	E	-2.346	MAX
nsp2	1	262	T	**0.591**	MIN	G	0.065	NEU
nsp2	1	263	L	**0.844**	MIN	L	0.882	MIN
nsp2	1	264	N	**0.529**	MAX	N	-0.925	NEU
nsp2	1	265	E	**0.960**	MAX	D	-1.772	MAX
nsp2	1	266	D	**0.529**	MAX	N	-0.775	NEU
nsp2	1	267	L	**1.313**	MIN	L	0.873	MIN
nsp2	1	268	L	**0.591**	MIN	L	0.565	NEU
nsp2	1	269	E	0.365	NEU	E	-0.475	NEU

nsp2	1	270	I	**1.313**	MIN	I	1.529	MIN
nsp2	1	271	L	**1.313**	MIN	L	1.049	MIN
nsp2	1	272	N	0.238	NEU	Q	-0.231	NEU
nsp2	1	273	R	0.071	NEU	K	-1.518	MAX
nsp2	1	274	E	-0.146	NEU	E	0.425	NEU
nsp2	1	275	R	-0.091	NEU	K	-1.622	MAX
nsp2	1	276	V	**1.313**	MIN	V	1.652	MIN
nsp2	1	277	N	**0.529**	NEU	N	0.561	NEU
nsp2	1	278	I	**1.313**	MIN	I	1.405	MIN
nsp2	1	279	N	**1.102**	NEU	N	-0.682	NEU
nsp2	1	280	I	**1.313**	MIN	I	2.087	MIN
nsp2	1	281	V	**0.529**	MIN	V	0.645	NEU
nsp2	1	282	G	**1.102**	NEU	G	0.051	NEU
nsp2	1	283	D	**0.591**	NEU	D	0.535	NEU
nsp2	1	284	F	0.395	NEU	F	0.246	NEU
nsp2	1	285	Q	-0.055	MAX	K	1.068	MIN
nsp2	1	286	L	**1.313**	MIN	L	1.205	MIN
nsp2	1	287	N	**0.529**	NEU	N	-0.988	NEU
nsp2	1	288	E	**0.746**	NEU	E	-0.951	NEU
nsp2	1	289	E	0.432	MAX	E	-1.139	MAX
nsp2	1	290	V	**1.313**	MIN	I	1.649	MIN
nsp2	1	291	A	0.326	MIN	A	0.417	NEU
nsp2	1	292	I	**1.313**	MIN	I	1.263	MIN
nsp2	1	293	I	**1.313**	MIN	I	1.534	MIN
nsp2	1	294	L	**1.313**	MIN	L	1.205	MIN
nsp2	1	295	A	0.432	NEU	A	0.544	NEU
nsp2	1	296	S	**1.313**	NEU	S	-0.69	NEU
nsp2	1	297	F	**1.313**	MIN	F	0.884	MIN
nsp2	1	298	S	**1.313**	NEU	S	-0.732	NEU
nsp2	1	299	A	**0.844**	NEU	A	0.385	NEU
nsp2	1	300	S	**1.313**	NEU	S	-0.264	NEU
nsp2	1	301	T	**1.102**	NEU	T	-0.361	NEU
nsp2	1	302	S	**0.960**	NEU	S	-0.757	NEU
nsp2	1	303	A	0.395	NEU	A	0.678	NEU
nsp2	1	304	F	**0.960**	MIN	F	1.06	MIN
nsp2	1	305	I	**1.313**	MIN	V	1.097	MIN
nsp2	1	306	D	**1.313**	NEU	E	-0.534	NEU
nsp2	1	307	T	**1.313**	NEU	T	-0.235	NEU
nsp2	1	308	V	**1.313**	MIN	V	1.229	MIN
nsp2	1	309	K	0.365	NEU	K	-0.744	NEU
nsp2	1	310	G	**1.313**	NEU	G	-0.1	NEU
nsp2	1	311	L	**1.313**	MIN	L	1.092	MIN
nsp2	1	312	D	**0.529**	MIN	D	-0.385	NEU
nsp2	1	313	Y	0.331	NEU	Y	0.78	MIN
nsp2	1	314	K	**0.960**	MAX	K	-1.745	MAX
nsp2	1	315	S	**0.960**	NEU	A	0.749	NEU

nsp2	1	316	F	**1.313**	MIN	F	0.76	NEU
nsp2	1	317	K	**1.313**	NEU	K	-0.771	NEU
nsp2	1	318	A	0.154	NEU	Q	-1.226	MAX
nsp2	1	319	I	**1.313**	MIN	I	2.039	MIN
nsp2	1	320	V	**1.313**	MIN	V	1.346	MIN
nsp2	1	321	E	**1.313**	NEU	E	-0.725	NEU
nsp2	1	322	S	**1.313**	NEU	S	-0.697	NEU
nsp2	1	323	C	**1.313**	MIN	C	1.787	MIN
nsp2	1	324	G	**0.960**	NEU	G	-1.004	MAX
nsp2	1	325	N	0.316	MAX	N	-1.207	MAX
nsp2	1	326	Y	0.326	NEU	F	0.433	NEU
nsp2	1	327	K	-0.186	NEU	K	-0.467	NEU
nsp2	1	328	V	**1.313**	MIN	V	1.307	MIN
nsp2	1	329	T	**1.313**	NEU	T	0.394	NEU
nsp2	1	330	K	**0.844**	NEU	K	-0.779	NEU
nsp2	1	331	G	**1.313**	NEU	G	-0.223	NEU
nsp2	1	332	K	**1.102**	NEU	K	0.401	NEU
nsp2	1	333	P	**0.543**	NEU	A	-0.129	NEU
nsp2	1	334	V	0.143	NEU	K	-0.634	NEU
nsp2	1	335	K	0.202	MIN	K	0.94	MIN
nsp2	1	336	G	**0.638**	NEU	G	-0.111	NEU
nsp2	1	337	A	**0.591**	NEU	A	-0.696	NEU
nsp2	1	338	W	0.313	MIN	W	0.453	NEU
nsp2	1	339	N	**1.313**	NEU	N	-0.744	NEU
nsp2	1	340	I	**1.313**	MIN	I	1.373	MIN
nsp2	1	341	G	**0.591**	NEU	G	-1.163	MAX
nsp2	1	342	Q	**0.960**	NEU	E	-0.765	NEU
nsp2	1	343	Q	0.342	NEU	Q	0.564	NEU
nsp2	1	344	K	0.040	MIN	K	1.71	MIN
nsp2	1	345	S	**1.313**	NEU	S	-0.2	NEU
nsp2	1	346	I	**0.746**	MIN	I	2.254	MIN
nsp2	1	347	L	**1.102**	MIN	L	0.799	MIN
nsp2	1	348	T	**1.102**	NEU	S	-0.434	NEU
nsp2	1	349	P	0.476	NEU	P	-1.138	MAX
nsp2	1	350	L	**1.313**	MIN	L	1.127	MIN
nsp2	1	351	C	-0.106	MIN	Y	-0.926	NEU
nsp2	1	352	G	**0.960**	NEU	A	0.369	NEU
nsp2	1	353	F	**1.102**	MIN	F	0.939	MIN
nsp2	1	354	P	**0.501**	NEU	A	0.632	NEU
nsp2	1	355	S	**1.313**	NEU	S	-0.478	NEU
nsp2	1	356	Q	0.432	NEU	E	-0.868	NEU
nsp2	1	357	A	**0.844**	NEU	A	0.1	NEU
nsp2	1	358	A	0.476	NEU	A	0.471	NEU
nsp2	1	359	G	**0.960**	NEU	R	-0.402	NEU
nsp2	1	360	V	**1.313**	MIN	V	1.194	MIN
nsp2	1	361	I	**1.313**	MIN	V	1.124	MIN

nsp2	1	362	R	**1.102**	NEU	R	-0.797	NEU
nsp2	1	363	S	**1.102**	NEU	S	-0.456	NEU
nsp2	1	364	I	**1.313**	MIN	I	1.251	MIN
nsp2	1	365	F	0.395	MIN	F	0.367	NEU
nsp2	1	366	S	**1.313**	NEU	S	-0.659	NEU
nsp2	1	367	R	**1.313**	NEU	R	-0.449	NEU
nsp2	1	368	T	**0.893**	NEU	T	-0.922	NEU
nsp2	1	369	L	**1.313**	MIN	L	1.206	MIN
nsp2	1	370	D	0.101	NEU	E	-0.725	NEU
nsp2	1	371	A	0.154	NEU	T	-0.161	NEU
nsp2	1	372	A	**0.638**	NEU	A	-0.387	NEU
nsp2	1	373	N	**0.529**	NEU	Q	0.762	NEU
nsp2	1	374	H	**0.960**	NEU	N	-1.199	MAX
nsp2	1	375	S	0.316	NEU	S	-0.329	NEU
nsp2	1	376	I	**1.313**	MIN	V	1.021	MIN
nsp2	1	377	P	0.202	NEU	R	1.234	MIN
nsp2	1	378	D	**0.529**	NEU	V	0.811	MIN
nsp2	1	379	L	**1.313**	MIN	L	1.423	MIN
nsp2	1	380	Q	0.326	MAX	Q	-0.901	NEU
nsp2	1	381	R	-0.144	NEU	K	-1.288	MAX
nsp2	1	382	A	**0.746**	MIN	A	0.774	NEU
nsp2	1	383	A	0.432	MIN	A	0.544	NEU
nsp2	1	384	V	**0.960**	MIN	I	1.682	MIN
nsp2	1	385	T	0.476	NEU	T	0.567	NEU
nsp2	1	386	I	**0.960**	MIN	I	1.866	MIN
nsp2	1	387	L	**1.313**	MIN	L	1.337	MIN
nsp2	1	388	D	**0.529**	MAX	D	-1.21	MAX
nsp2	1	389	G	0.476	NEU	G	-1.255	MAX
nsp2	1	390	I	**1.313**	MIN	I	1.477	MIN
nsp2	1	391	S	**1.102**	NEU	S	-0.83	NEU
nsp2	1	392	E	**0.529**	NEU	Q	-0.351	NEU
nsp2	1	393	Q	**0.543**	NEU	Y	0.498	NEU
nsp2	1	394	S	**1.313**	NEU	S	-0.793	NEU
nsp2	1	395	L	**0.960**	MIN	L	0.753	NEU
nsp2	1	396	R	0.432	MIN	R	0.09	NEU
nsp2	1	397	L	**1.313**	MIN	L	1.309	MIN
nsp2	1	398	V	**1.313**	MIN	I	1.298	MIN
nsp2	1	399	D	0.365	NEU	D	-0.954	NEU
nsp2	1	400	A	0.342	MIN	A	0.546	NEU
nsp2	1	401	M	**1.313**	MIN	M	0.672	NEU
nsp2	1	402	V	**0.591**	MIN	M	0.79	MIN
nsp2	1	403	Y	0.156	NEU	F	0.326	NEU
nsp2	1	404	T	**1.313**	NEU	T	-0.326	NEU
nsp2	1	405	S	**1.313**	NEU	S	-0.708	NEU
nsp2	1	406	D	0.299	NEU	D	-0.152	NEU
nsp2	1	407	L	**0.960**	MIN	L	1.22	MIN

nsp2	1	408	L	0.461	MIN	A	-1.354	MAX
nsp2	1	409	T	**1.102**	NEU	T	-0.203	NEU
nsp2	1	410	N	**1.102**	NEU	N	-0.518	NEU
nsp2	1	411	S	**0.529**	NEU	N	-1.104	MAX
nsp2	1	412	V	**1.313**	MIN	L	1.468	MIN
nsp2	1	413	I	**1.313**	MIN	V	1.058	MIN
nsp2	1	414	V	**1.313**	MIN	V	1.577	MIN
nsp2	1	415	M	**0.529**	MIN	M	0.478	NEU
nsp2	1	416	A	0.326	MIN	A	0.581	NEU
nsp2	1	417	Y	0.316	MIN	Y	0.616	NEU
nsp2	1	418	V	**1.313**	MIN	I	1.341	MIN
nsp2	1	419	T	**1.313**	NEU	T	-0.275	NEU
nsp2	1	420	G	**1.313**	NEU	G	-0.774	NEU
nsp2	1	421	G	**1.313**	NEU	G	-0.689	NEU
nsp2	1	422	L	**1.313**	MIN	V	1.244	MIN
nsp2	1	423	V	**1.313**	MIN	V	1.162	MIN
nsp2	1	424	Q	**0.591**	NEU	Q	-0.883	NEU
nsp2	1	425	Q	0.154	NEU	L	1.533	MIN
nsp2	1	426	T	**0.529**	NEU	T	-0.39	NEU
nsp2	1	427	S	**0.752**	NEU	S	-0.481	NEU
nsp2	1	428	Q	**0.752**	NEU	Q	-0.814	NEU
nsp2	1	429	W	0.391	MIN	W	0.863	MIN
nsp2	1	430	L	**1.313**	MIN	L	1.345	MIN
nsp2	1	431	S	**1.102**	NEU	T	-0.043	NEU
nsp2	1	432	N	**0.960**	NEU	N	-0.71	NEU
nsp2	1	433	L	**1.102**	MIN	I	2.048	MIN
nsp2	1	434	L	**0.529**	MIN	F	0.46	NEU
nsp2	1	435	G	**0.893**	NEU	G	-0.759	NEU
nsp2	1	436	T	**0.960**	NEU	T	-0.303	NEU
nsp2	1	437	T	**0.591**	MIN	V	1.532	MIN
nsp2	1	438	V	-0.106	MIN	Y	-1.097	MAX
nsp2	1	439	E	0.365	MIN	E	0.388	NEU
nsp2	1	440	K	0.331	MAX	K	-1.436	MAX
nsp2	1	441	L	**1.102**	MIN	L	1.329	MIN
nsp2	1	442	K	0.476	NEU	K	-0.579	NEU
nsp2	1	443	P	**0.960**	NEU	P	-0.882	NEU
nsp2	1	444	V	**1.313**	MIN	V	1.166	MIN
nsp2	1	445	F	**1.313**	MIN	L	1.176	MIN
nsp2	1	446	A	0.391	NEU	D	-1.467	MAX
nsp2	1	447	W	0.326	MIN	W	0.64	NEU
nsp2	1	448	I	**1.313**	MIN	L	1.196	MIN
nsp2	1	449	E	**0.844**	NEU	E	-0.517	NEU
nsp2	1	450	A	-0.091	NEU	E	-1.108	MAX
nsp2	1	451	K	**0.960**	NEU	K	-0.123	NEU
nsp2	1	452	L	**1.313**	MIN	F	0.936	MIN
nsp2	1	453	S	0.281	NEU	K	1.368	MIN

nsp2	1	454	A	**0.844**	NEU	E	-0.685	NEU
nsp2	1	455	G	**1.313**	NEU	G	-0.736	NEU
nsp2	1	456	V	**1.313**	MIN	V	1.399	MIN
nsp2	1	457	E	**0.960**	NEU	E	-0.575	NEU
nsp2	1	458	F	**0.746**	MIN	F	0.587	NEU
nsp2	1	459	L	**1.313**	MIN	L	1.366	MIN
nsp2	1	460	K	**0.529**	NEU	R	0.659	NEU
nsp2	1	461	D	0.476	MAX	D	-1.443	MAX
nsp2	1	462	A	0.365	MIN	G	-0.612	NEU
nsp2	1	463	W	**0.529**	MIN	W	0.637	NEU
nsp2	1	464	E	**1.102**	NEU	E	-0.959	NEU
nsp2	1	465	I	**1.313**	MIN	I	1.292	MIN
nsp2	1	466	L	**1.313**	MIN	V	0.983	MIN
nsp2	1	467	K	0.316	MAX	K	-0.795	NEU
nsp2	1	468	F	**0.960**	MIN	F	1.279	MIN
nsp2	1	469	L	**1.313**	MIN	I	1.313	MIN
nsp2	1	470	I	0.476	MIN	S	-0.567	NEU
nsp2	1	471	T	**0.844**	NEU	T	-0.723	NEU
nsp2	1	472	G	**0.529**	NEU	C	1.721	MIN
nsp2	1	473	V	0.432	MIN	A	0.511	NEU
nsp2	1	474	F	**0.746**	MIN	C	1.315	MIN
nsp2	1	475	D	0.461	MAX	E	-1.094	MAX
nsp2	1	476	I	**1.313**	MIN	I	2.116	MIN
nsp2	1	477	V	0.342	NEU	V	1.254	MIN
nsp2	1	478	K	**1.313**	NEU	G	-0.56	NEU
nsp2	1	479	G	**0.960**	NEU	G	-0.519	NEU
nsp2	1	480	Q	0.476	NEU	Q	-0.944	NEU
nsp2	1	481	I	**1.313**	MIN	I	1.124	MIN
nsp2	1	482	Q	-0.193	MAX	V	0.913	MIN
nsp2	1	483	V	0.476	MIN	T	-0.509	NEU
nsp2	1	484	A	0.202	NEU	C	0.676	NEU
nsp2	1	485	S	0.461	NEU	A	0.914	MIN
nsp2	1	486	D	**0.844**	NEU	K	0.467	NEU
nsp2	1	487	N	0.114	MIN	E	0.496	NEU
nsp2	1	488	I	**1.313**	MIN	I	1.434	MIN
nsp2	1	489	K	**0.844**	NEU	K	0.357	NEU
nsp2	1	490	E	0.461	NEU	E	-0.955	NEU
nsp2	1	491	C	0.432	MIN	S	-0.454	NEU
nsp2	1	492	V	**1.313**	MIN	V	0.964	MIN
nsp2	1	493	K	**1.102**	NEU	Q	-0.837	NEU
nsp2	1	494	T	0.204	NEU	T	0.081	NEU
nsp2	1	495	F	**1.313**	MIN	F	0.755	NEU
nsp2	1	496	I	**1.102**	MIN	F	0.681	NEU
nsp2	1	497	D	-0.237	MIN	K	-2.616	MAX
nsp2	1	498	V	**0.960**	MIN	L	1.387	MIN
nsp2	1	499	V	**1.313**	MIN	V	1.058	MIN

nsp2	1	500	N	0.365	MAX	N	-1.041	MAX
nsp2	1	501	K	0.365	NEU	K	-1.669	MAX
nsp2	1	502	A	0.476	MIN	F	1.238	MIN
nsp3_Ubl1	1	1	A	**0.767**	NEU	A	-0.882	NEU
nsp3_Ubl1	1	2	P	0.037	MIN	P	0.305	NEU
nsp3_Ubl1	1	3	T	0.376	NEU	T	-0.425	NEU
nsp3_Ubl1	1	4	K	-0.147	MAX	K	1.508	MIN
nsp3_Ubl1	1	5	V	0.376	NEU	V	1.172	MIN
nsp3_Ubl1	1	6	T	**0.856**	NEU	T	0.631	NEU
nsp3_Ubl1	1	7	F	**1.109**	NEU	F	0.288	NEU
nsp3_Ubl1	1	8	G	**1.314**	NEU	G	-0.139	NEU
nsp3_Ubl1	1	9	E	**1.109**	NEU	D	-0.817	NEU
nsp3_Ubl1	1	10	D	**1.314**	MAX	D	-1.395	MAX
nsp3_Ubl1	1	11	T	0.333	NEU	T	0.152	NEU
nsp3_Ubl1	1	12	V	0.376	NEU	V	0.552	NEU
nsp3_Ubl1	1	13	L	**0.767**	MIN	I	1.129	MIN
nsp3_Ubl1	1	14	E	0.315	MAX	E	-1.244	MAX
nsp3_Ubl1	1	15	V	**1.314**	MIN	V	1.407	MIN
nsp3_Ubl1	1	16	Q	**0.655**	NEU	Q	-0.817	NEU
nsp3_Ubl1	1	17	G	0.491	MAX	G	-1.244	MAX
nsp3_Ubl1	1	18	Y	**0.905**	MAX	Y	-1.137	MAX
nsp3_Ubl1	1	19	K	0.299	MAX	K	-1.32	MAX
nsp3_Ubl1	1	20	N	0.445	NEU	S	0.063	NEU
nsp3_Ubl1	1	21	V	**1.314**	MIN	V	1.13	MIN
nsp3_Ubl1	1	22	K	0.321	NEU	N	-0.372	NEU
nsp3_Ubl1	1	23	I	**1.314**	MIN	I	1.121	MIN
nsp3_Ubl1	1	24	T	**1.314**	NEU	T	0.117	NEU
nsp3_Ubl1	1	25	F	**1.109**	MIN	F	0.64	NEU
nsp3_Ubl1	1	26	E	**1.109**	MAX	E	-1.129	MAX
nsp3_Ubl1	1	27	L	**1.314**	MIN	L	1.049	MIN
nsp3_Ubl1	1	28	D	**1.314**	NEU	D	-0.777	NEU
nsp3_Ubl1	1	29	E	**1.109**	NEU	E	0.47	NEU
nsp3_Ubl1	1	30	R	**0.969**	MIN	R	1.086	MIN
nsp3_Ubl1	1	31	V	**1.314**	MIN	I	1.909	MIN
nsp3_Ubl1	1	32	D	**1.109**	MAX	D	-1.495	MAX
nsp3_Ubl1	1	33	K	**1.109**	MIN	K	1.296	MIN
nsp3_Ubl1	1	34	V	**1.314**	MIN	V	1.627	MIN
nsp3_Ubl1	1	35	L	**1.314**	MIN	L	1.565	MIN
nsp3_Ubl1	1	36	N	**0.544**	NEU	N	-0.89	NEU
nsp3_Ubl1	1	37	E	**1.109**	NEU	E	-0.365	NEU
nsp3_Ubl1	1	38	K	**0.969**	MAX	K	-1.189	MAX
nsp3_Ubl1	1	39	C	**1.109**	MIN	C	1.18	MIN
nsp3_Ubl1	1	40	S	**0.767**	NEU	S	0.116	NEU
nsp3_Ubl1	1	41	V	0.333	MIN	A	-0.958	NEU
nsp3_Ubl1	1	42	Y	**1.109**	NEU	Y	0.415	NEU
nsp3_Ubl1	1	43	T	0.352	NEU	T	-0.848	NEU

nsp3_Ubl1	1	44	V	**1.314**	MIN	V	1.306	MIN
nsp3_Ubl1	1	45	E	0.491	MAX	E	-1.404	MAX
nsp3_Ubl1	1	46	S	0.256	NEU	L	1.438	MIN
nsp3_Ubl1	1	47	G	**0.760**	NEU	G	-1.128	MAX
nsp3_Ubl1	1	48	T	**1.109**	NEU	T	0.23	NEU
nsp3_Ubl1	1	49	E	0.169	MAX	E	-1.972	MAX
nsp3_Ubl1	1	50	V	**1.314**	MIN	V	1.144	MIN
nsp3_Ubl1	1	51	T	**0.856**	NEU	N	-0.581	NEU
nsp3_Ubl1	1	52	E	0.445	NEU	E	-1.156	MAX
nsp3_Ubl1	1	53	F	**1.314**	MIN	F	0.981	MIN
nsp3_Ubl1	1	54	A	0.376	NEU	A	0.654	NEU
nsp3_Ubl1	1	55	C	**0.969**	MIN	C	1.659	MIN
nsp3_Ubl1	1	56	V	**1.314**	MIN	V	1.4	MIN
nsp3_Ubl1	1	57	V	**1.314**	MIN	V	1.306	MIN
nsp3_Ubl1	1	58	A	**1.314**	NEU	A	0.294	NEU
nsp3_Ubl1	1	59	E	**0.856**	NEU	D	-0.99	NEU
nsp3_Ubl1	1	60	A	0.491	MIN	A	0.553	NEU
nsp3_Ubl1	1	61	V	**1.314**	MIN	V	1.523	MIN
nsp3_Ubl1	1	62	V	**1.314**	MIN	I	0.973	MIN
nsp3_Ubl1	1	63	K	**1.314**	NEU	K	-0.694	NEU
nsp3_Ubl1	1	64	T	**1.314**	NEU	T	-0.472	NEU
nsp3_Ubl1	1	65	L	**1.314**	MIN	L	1.116	MIN
nsp3_Ubl1	1	66	Q	0.445	MAX	Q	-1.148	MAX
nsp3_Ubl1	1	67	P	0.299	MIN	P	0.757	NEU
nsp3_Ubl1	1	68	V	**1.314**	MIN	V	1.464	MIN
nsp3_Ubl1	1	69	S	**1.314**	NEU	S	-0.767	NEU
nsp3_Ubl1	1	70	D	0.376	NEU	E	0.651	NEU
nsp3_Ubl1	1	71	L	**1.109**	MIN	L	0.94	MIN
nsp3_Ubl1	1	72	L	**1.314**	MIN	L	1.229	MIN
nsp3_Ubl1	1	73	T	**0.760**	MIN	T	1.068	MIN
nsp3_Ubl1	1	74	N	0.445	NEU	P	0.35	NEU
nsp3_Ubl1	1	75	M	**1.314**	MIN	L	1.127	MIN
nsp3_Ubl1	1	76	G	**1.314**	NEU	G	0.236	NEU
nsp3_Ubl1	1	77	I	**1.314**	MIN	I	1.671	MIN
nsp3_Ubl1	1	78	D	0.333	NEU	D	-1.435	MAX
nsp3_Ubl1	1	79	L	**1.314**	MIN	L	1.179	MIN
nsp3_Ubl1	1	80	D	**1.109**	NEU	D	-0.59	NEU
nsp3_Ubl1	1	81	E	0.376	NEU	E	-1.267	MAX
nsp3_Ubl1	1	82	W	**1.314**	MIN	W	0.754	NEU
nsp3_Ubl1	1	83	S	**1.109**	NEU	S	-0.779	NEU
nsp3_Ubl1	1	84	V	0.350	MIN	M	-1.602	MAX
nsp3_Ubl1	1	85	A	**1.314**	NEU	A	0.309	NEU
nsp3_Ubl1	1	86	T	**0.905**	NEU	T	-0.64	NEU
nsp3_Ubl1	1	87	F	0.407	MIN	Y	0.424	NEU
nsp3_Ubl1	1	88	Y	0.376	MIN	Y	0.022	NEU
nsp3_Ubl1	1	89	L	**1.314**	MIN	L	1.285	MIN

nsp3_Ubl1	1	90	F	**1.314**	MIN	F	1.241	MIN
nsp3_Ubl1	1	91	D	**0.856**	NEU	D	-0.766	NEU
nsp3_Ubl1	1	92	D	0.445	NEU	E	-1.355	MAX
nsp3_Ubl1	1	93	A	0.333	NEU	S	1.283	MIN
nsp3_Ubl1	1	94	G	**1.314**	NEU	G	0.29	NEU
nsp3_Ubl1	1	95	E	**1.314**	MAX	E	-1.099	MAX
nsp3_Ubl1	1	96	E	-0.028	MAX	F	1.022	MIN
nsp3_Ubl1	1	97	K	**0.856**	NEU	K	-0.411	NEU
nsp3_Ubl1	1	98	L	**1.314**	MIN	L	1.306	MIN
nsp3_Ubl1	1	99	S	0.315	NEU	A	-0.018	NEU
nsp3_Ubl1	1	100	S	**1.314**	NEU	S	-0.732	NEU
nsp3_Ubl1	1	101	R	**0.677**	MIN	H	0.302	NEU
nsp3_Ubl1	1	102	M	**1.314**	MIN	M	0.831	MIN
nsp3_Ubl1	1	103	Y	**1.109**	NEU	Y	0.512	NEU
nsp3_Ubl1	1	104	C	**1.314**	MIN	C	1.23	MIN
nsp3_Ubl1	1	105	S	**1.314**	NEU	S	-0.58	NEU
nsp3_Ubl1	1	106	F	**1.109**	MIN	F	0.837	MIN
nsp3_Ubl1	1	107	Y	**1.109**	MAX	Y	-3.043	MAX
nsp3_Ubl1	1	108	P	**0.969**	MIN	P	1.279	MIN
nsp3_Ubl1	1	109	P	0.445	NEU	P	0.764	NEU
nsp3_Ubl1	1	110	D	**1.311**	NEU	D	0.189	NEU
nsp3_Ubl1	1	111	E	**0.856**	MIN	E	0.765	NEU
nsp3_Macro›	1	1	E	-0.147	MIN	E	2.33	MIN
nsp3_Macro›	1	2	V	**0.561**	NEU	V	-0.859	NEU
nsp3_Macro›	1	3	N	0.133	MAX	N	-1.063	MAX
nsp3_Macro›	1	4	Q	**0.856**	NEU	S	-0.423	NEU
nsp3_Macro›	1	5	F	0.315	MIN	F	0.642	NEU
nsp3_Macro›	1	6	T	**0.856**	NEU	S	-0.011	NEU
nsp3_Macro›	1	7	G	**0.856**	NEU	G	0.248	NEU
nsp3_Macro›	1	8	Y	0.352	MIN	Y	0.522	NEU
nsp3_Macro›	1	9	L	**0.856**	MIN	L	1.4	MIN
nsp3_Macro›	1	10	K	**1.314**	NEU	K	-0.37	NEU
nsp3_Macro›	1	11	L	**1.314**	MIN	L	1.257	MIN
nsp3_Macro›	1	12	T	**1.314**	NEU	T	-0.283	NEU
nsp3_Macro›	1	13	D	**0.760**	MIN	D	0.87	MIN
nsp3_Macro›	1	14	N	0.321	NEU	N	-0.83	NEU
nsp3_Macro›	1	15	V	**1.314**	MIN	V	1.148	MIN
nsp3_Macro›	1	16	A	0.428	NEU	Y	-0.568	NEU
nsp3_Macro›	1	17	I	**1.314**	MIN	I	1.525	MIN
nsp3_Macro›	1	18	K	**1.109**	MAX	K	-1.228	MAX
nsp3_Macro›	1	19	C	**0.544**	MIN	N	-1.012	MAX
nsp3_Macro›	1	20	V	0.445	MIN	A	-0.079	NEU
nsp3_Macro›	1	21	D	0.321	NEU	D	-1.774	MAX
nsp3_Macro›	1	22	I	**1.109**	MIN	I	1.483	MIN
nsp3_Macro›	1	23	V	**1.314**	MIN	V	1.427	MIN
nsp3_Macro›	1	24	K	-0.034	MAX	E	0.854	MIN

nsp3_Macro)	1	25	E	**0.856**	NEU	E	-0.572	NEU
nsp3_Macro)	1	26	A	**0.760**	MIN	A	0.628	NEU
nsp3_Macro)	1	27	Q	0.491	NEU	K	1.197	MIN
nsp3_Macro)	1	28	S	**0.544**	NEU	K	2.203	MIN
nsp3_Macro)	1	29	A	0.352	MIN	V	1.321	MIN
nsp3_Macro)	1	30	K	-0.004	NEU	K	0.169	NEU
nsp3_Macro)	1	31	P	**0.606**	NEU	P	-0.817	NEU
nsp3_Macro)	1	32	T	0.350	NEU	T	-0.07	NEU
nsp3_Macro)	1	33	V	**1.314**	MIN	V	1.317	MIN
nsp3_Macro)	1	34	I	**1.314**	MIN	V	1.105	MIN
nsp3_Macro)	1	35	V	**1.314**	MIN	V	1.217	MIN
nsp3_Macro)	1	36	N	**0.969**	NEU	N	-0.944	NEU
nsp3_Macro)	1	37	A	**1.314**	NEU	A	0.328	NEU
nsp3_Macro)	1	38	A	**0.856**	NEU	A	0.574	NEU
nsp3_Macro)	1	39	N	**1.314**	NEU	N	-0.908	NEU
nsp3_Macro)	1	40	I	**0.856**	MIN	V	1.231	MIN
nsp3_Macro)	1	41	H	0.410	NEU	Y	1.067	MIN
nsp3_Macro)	1	42	L	**1.314**	MIN	L	1.228	MIN
nsp3_Macro)	1	43	K	**1.314**	NEU	K	0.168	NEU
nsp3_Macro)	1	44	H	**1.109**	NEU	H	-0.785	NEU
nsp3_Macro)	1	45	G	**1.109**	NEU	G	-0.339	NEU
nsp3_Macro)	1	46	G	**1.109**	NEU	G	-0.801	NEU
nsp3_Macro)	1	47	G	**1.314**	NEU	G	-0.242	NEU
nsp3_Macro)	1	48	V	**1.314**	MIN	V	1.393	MIN
nsp3_Macro)	1	49	A	**1.314**	NEU	A	0.4	NEU
nsp3_Macro)	1	50	G	**1.314**	NEU	G	0.102	NEU
nsp3_Macro)	1	51	A	**1.314**	NEU	A	0.449	NEU
nsp3_Macro)	1	52	L	**1.314**	MIN	L	1.167	MIN
nsp3_Macro)	1	53	N	**1.314**	NEU	N	-0.503	NEU
nsp3_Macro)	1	54	K	**1.314**	NEU	K	0.047	NEU
nsp3_Macro)	1	55	A	**1.314**	NEU	A	0.093	NEU
nsp3_Macro)	1	56	T	**1.109**	NEU	T	-0.034	NEU
nsp3_Macro)	1	57	N	**1.314**	NEU	N	0.122	NEU
nsp3_Macro)	1	58	G	**1.314**	NEU	N	-0.324	NEU
nsp3_Macro)	1	59	A	0.376	MAX	A	-0.618	NEU
nsp3_Macro)	1	60	M	**1.109**	NEU	M	-0.103	NEU
nsp3_Macro)	1	61	Q	**0.969**	NEU	Q	-0.243	NEU
nsp3_Macro)	1	62	K	0.144	NEU	V	0.301	NEU
nsp3_Macro)	1	63	E	**1.109**	MAX	E	-1.812	MAX
nsp3_Macro)	1	64	S	0.220	MAX	S	-0.789	NEU
nsp3_Macro)	1	65	D	-0.215	MIN	D	-1.163	MAX
nsp3_Macro)	1	66	D	0.074	NEU	D	-1.354	MAX
nsp3_Macro)	1	67	Y	**0.760**	NEU	Y	0.41	NEU
nsp3_Macro)	1	68	I	**1.314**	MIN	I	2.14	MIN
nsp3_Macro)	1	69	K	0.407	MIN	A	-0.219	NEU
nsp3_Macro)	1	70	L	0.035	MIN	T	-0.615	NEU

nsp3_Macro›	1	71	N	**1.109**	NEU	N	-0.629	NEU
nsp3_Macro›	1	72	G	**1.314**	NEU	G	-0.214	NEU
nsp3_Macro›	1	73	P	**0.969**	NEU	P	-0.291	NEU
nsp3_Macro›	1	74	L	**1.314**	MIN	L	1.232	MIN
nsp3_Macro›	1	75	T	0.428	NEU	K	-1.378	MAX
nsp3_Macro›	1	76	V	**1.314**	MIN	V	1.145	MIN
nsp3_Macro›	1	77	G	**1.314**	NEU	G	-0.365	NEU
nsp3_Macro›	1	78	G	**1.314**	NEU	G	-0.448	NEU
nsp3_Macro›	1	79	S	**1.314**	NEU	S	-0.638	NEU
nsp3_Macro›	1	80	C	**1.109**	MIN	C	1.566	MIN
nsp3_Macro›	1	81	L	**0.760**	MIN	V	0.66	NEU
nsp3_Macro›	1	82	L	**1.314**	MIN	L	1.332	MIN
nsp3_Macro›	1	83	S	**1.314**	NEU	S	-0.365	NEU
nsp3_Macro›	1	84	G	**1.314**	NEU	G	-0.86	NEU
nsp3_Macro›	1	85	H	**1.314**	NEU	H	-0.577	NEU
nsp3_Macro›	1	86	N	**0.969**	NEU	N	0.225	NEU
nsp3_Macro›	1	87	L	**1.314**	MIN	L	1.247	MIN
nsp3_Macro›	1	88	A	**1.109**	NEU	A	0.351	NEU
nsp3_Macro›	1	89	K	0.315	MAX	K	-0.266	NEU
nsp3_Macro›	1	90	K	**0.677**	MAX	H	-0.329	NEU
nsp3_Macro›	1	91	C	**1.314**	MIN	C	1.068	MIN
nsp3_Macro›	1	92	L	**1.314**	MIN	L	1.057	MIN
nsp3_Macro›	1	93	H	**1.314**	NEU	H	-0.003	NEU
nsp3_Macro›	1	94	V	**1.314**	MIN	V	1.256	MIN
nsp3_Macro›	1	95	V	**1.314**	MIN	V	1.085	MIN
nsp3_Macro›	1	96	G	**1.314**	NEU	G	-0.279	NEU
nsp3_Macro›	1	97	P	0.407	NEU	P	-0.861	NEU
nsp3_Macro›	1	98	N	**1.314**	NEU	N	-0.626	NEU
nsp3_Macro›	1	99	L	**0.969**	MIN	V	1.018	MIN
nsp3_Macro›	1	100	N	**0.544**	NEU	N	-0.12	NEU
nsp3_Macro›	1	101	A	**1.314**	NEU	K	0.419	NEU
nsp3_Macro›	1	102	G	**0.856**	NEU	G	-0.986	NEU
nsp3_Macro›	1	103	E	**0.856**	MAX	E	-0.969	NEU
nsp3_Macro›	1	104	D	**0.856**	MAX	D	-1.241	MAX
nsp3_Macro›	1	105	I	**1.109**	MIN	I	1.71	MIN
nsp3_Macro›	1	106	Q	0.412	NEU	Q	-0.214	NEU
nsp3_Macro›	1	107	L	**0.677**	MIN	L	0.403	NEU
nsp3_Macro›	1	108	L	**1.314**	MIN	L	0.914	MIN
nsp3_Macro›	1	109	K	**0.655**	NEU	K	-0.68	NEU
nsp3_Macro›	1	110	A	**0.969**	NEU	S	-0.587	NEU
nsp3_Macro›	1	111	A	**1.314**	MIN	A	0.798	MIN
nsp3_Macro›	1	112	Y	**1.314**	MIN	Y	0.896	MIN
nsp3_Macro›	1	113	E	0.480	NEU	E	0.786	MIN
nsp3_Macro›	1	114	N	**0.969**	NEU	N	-0.841	NEU
nsp3_Macro›	1	115	F	**1.314**	MIN	F	1.273	MIN
nsp3_Macro›	1	116	N	0.321	NEU	N	-0.895	NEU

nsp3_Macro>	1	117	S	**1.314**	NEU	Q	-0.331	NEU
nsp3_Macro>	1	118	Q	**1.109**	NEU	H	-0.179	NEU
nsp3_Macro>	1	119	D	0.191	MAX	E	-0.432	NEU
nsp3_Macro>	1	120	V	0.350	MIN	V	1.221	MIN
nsp3_Macro>	1	121	L	**1.314**	MIN	L	1.259	MIN
nsp3_Macro>	1	122	L	**1.314**	MIN	L	1.34	MIN
nsp3_Macro>	1	123	A	**1.314**	NEU	A	0.296	NEU
nsp3_Macro>	1	124	P	**1.109**	MAX	P	-1.037	MAX
nsp3_Macro>	1	125	L	**1.314**	MIN	L	1.125	MIN
nsp3_Macro>	1	126	L	**1.314**	MIN	L	0.972	MIN
nsp3_Macro>	1	127	S	**1.314**	NEU	S	-0.539	NEU
nsp3_Macro>	1	128	A	**1.314**	NEU	A	-0.172	NEU
nsp3_Macro>	1	129	G	**1.314**	NEU	G	-0.415	NEU
nsp3_Macro>	1	130	I	**1.109**	MIN	I	1.739	MIN
nsp3_Macro>	1	131	F	**1.314**	MIN	F	0.947	MIN
nsp3_Macro>	1	132	G	**1.109**	NEU	G	0.433	NEU
nsp3_Macro>	1	133	A	0.491	NEU	A	0.708	NEU
nsp3_Macro>	1	134	K	**1.109**	NEU	D	-0.689	NEU
nsp3_Macro>	1	135	P	**1.314**	NEU	P	-0.686	NEU
nsp3_Macro>	1	136	L	**1.109**	MIN	I	1.627	MIN
nsp3_Macro>	1	137	Q	**0.655**	NEU	H	-0.598	NEU
nsp3_Macro>	1	138	S	**1.314**	NEU	S	-0.667	NEU
nsp3_Macro>	1	139	L	**1.314**	MIN	L	1.236	MIN
nsp3_Macro>	1	140	K	0.428	NEU	R	0.635	NEU
nsp3_Macro>	1	141	V	**0.856**	MIN	V	1.296	MIN
nsp3_Macro>	1	142	C	**1.314**	MIN	C	1.211	MIN
nsp3_Macro>	1	143	V	**1.109**	MIN	V	1.286	MIN
nsp3_Macro>	1	144	E	**0.767**	NEU	D	0.092	NEU
nsp3_Macro>	1	145	T	0.350	NEU	T	-0.347	NEU
nsp3_Macro>	1	146	V	**1.314**	MIN	V	1.115	MIN
nsp3_Macro>	1	147	R	**0.655**	MIN	R	1.529	MIN
nsp3_Macro>	1	148	T	**0.905**	NEU	T	-0.229	NEU
nsp3_Macro>	1	149	Q	**0.856**	NEU	N	-0.911	NEU
nsp3_Macro>	1	150	V	**1.314**	MIN	V	1.217	MIN
nsp3_Macro>	1	151	Y	**0.606**	MIN	Y	0.808	MIN
nsp3_Macro>	1	152	I	**1.314**	MIN	L	1.453	MIN
nsp3_Macro>	1	153	A	**0.544**	NEU	A	0.283	NEU
nsp3_Macro>	1	154	V	**1.314**	MIN	V	1.366	MIN
nsp3_Macro>	1	155	N	0.191	NEU	F	-0.662	NEU
nsp3_Macro>	1	156	D	0.407	MAX	D	0.279	NEU
nsp3_Macro>	1	157	K	0.220	NEU	K	-1.184	MAX
nsp3_Macro>	1	158	A	0.299	MIN	N	1.307	MIN
nsp3_Macro>	1	159	L	**1.109**	MIN	L	1.377	MIN
nsp3_Macro>	1	160	Y	**0.561**	MAX	Y	-1.116	MAX
nsp3_Macro>	1	161	D	0.350	NEU	D	-0.083	NEU
nsp3_Macro>	1	162	Q	**0.561**	MAX	K	-1.548	MAX

nsp3_Macro)	1	163	V	**1.314**	MIN	L	1.225	MIN
nsp3_Macro)	1	164	V	**0.969**	MIN	V	1.33	MIN
nsp3_Macro)	1	165	M	0.376	NEU	S	-0.522	NEU
nsp3_Macro)	1	166	D	0.321	NEU	S	-0.682	NEU
nsp3_Macro)	1	167	Y	0.445	NEU	F	0.696	NEU
nsp3_Macro)	1	168	L	**1.102**	MIN	L	0.99	MIN
nsp3_Macro)	1	169	D	**1.313**	NEU	E	0.315	NEU
nsp3_Macro)	1	170	L	**0.606**	NEU	M	0.548	NEU
nsp3_Macro)	1	171	K	0.376	MIN	K	-0.886	NEU
nsp3_Macro)	1	172	P	**0.969**	NEU	S	0.025	NEU
nsp3_SUD_N	1	1	K	**0.844**	NEU	K	0.362	NEU
nsp3_SUD_N	1	2	I	**0.844**	MIN	I	0.603	NEU
nsp3_SUD_N	1	3	K	**1.102**	MAX	K	-1.564	MAX
nsp3_SUD_N	1	4	A	**0.638**	NEU	A	0.497	NEU
nsp3_SUD_N	1	5	C	**0.663**	MIN	C	1.531	MIN
nsp3_SUD_N	1	6	I	**1.313**	MIN	V	1.387	MIN
nsp3_SUD_N	1	7	E	**0.844**	MAX	E	-1.049	MAX
nsp3_SUD_N	1	8	E	**1.102**	NEU	E	-0.727	NEU
nsp3_SUD_N	1	9	V	**1.313**	MIN	V	1.372	MIN
nsp3_SUD_N	1	10	T	**1.313**	NEU	T	-0.114	NEU
nsp3_SUD_N	1	11	T	**1.102**	NEU	T	-0.233	NEU
nsp3_SUD_N	1	12	T	**0.969**	MIN	T	1.155	MIN
nsp3_SUD_N	1	13	L	**1.109**	MIN	L	1.527	MIN
nsp3_SUD_N	1	14	E	**0.905**	MIN	E	1.038	MIN
nsp3_SUD_N	1	15	E	**1.314**	NEU	E	0.367	NEU
nsp3_SUD_N	1	16	T	**0.856**	NEU	T	-0.168	NEU
nsp3_SUD_N	1	17	K	0.100	MIN	K	0.739	NEU
nsp3_SUD_N	1	18	F	**0.905**	NEU	F	-0.751	NEU
nsp3_SUD_N	1	19	L	**0.760**	MIN	L	0.71	NEU
nsp3_SUD_N	1	20	T	**1.109**	NEU	T	-0.512	NEU
nsp3_SUD_N	1	21	N	**1.109**	NEU	E	-0.671	NEU
nsp3_SUD_N	1	22	K	**0.969**	NEU	N	-0.834	NEU
nsp3_SUD_N	1	23	L	**1.109**	MIN	L	1.307	MIN
nsp3_SUD_N	1	24	L	**1.314**	MIN	L	1.435	MIN
nsp3_SUD_N	1	25	L	**1.109**	MIN	L	1.241	MIN
nsp3_SUD_N	1	26	F	**1.314**	MIN	Y	0.923	MIN
nsp3_SUD_N	1	27	A	0.321	NEU	I	1.269	MIN
nsp3_SUD_N	1	28	D	**1.314**	NEU	D	-0.194	NEU
nsp3_SUD_N	1	29	I	**1.109**	MIN	I	1.444	MIN
nsp3_SUD_N	1	30	N	**0.760**	NEU	N	0.721	NEU
nsp3_SUD_N	1	31	G	**1.109**	NEU	G	-0.611	NEU
nsp3_SUD_N	1	32	K	**1.109**	NEU	N	-0.705	NEU
nsp3_SUD_N	1	33	L	**0.969**	MIN	L	1.347	MIN
nsp3_SUD_N	1	34	Y	0.191	NEU	H	-0.4	NEU
nsp3_SUD_N	1	35	P	-0.124	NEU	P	-0.94	NEU
nsp3_SUD_N	1	36	D	0.352	MAX	D	-0.991	NEU

nsp3_SUD_N	1	37	S	**1.314**	NEU	S	-0.676	NEU
nsp3_SUD_N	1	38	Q	0.333	MAX	A	-0.633	NEU
nsp3_SUD_N	1	39	N	0.376	NEU	T	-0.222	NEU
nsp3_SUD_N	1	40	M	**1.314**	MIN	L	1.529	MIN
nsp3_SUD_N	1	41	L	**1.314**	MIN	V	1.269	MIN
nsp3_SUD_N	1	42	R	0.123	MIN	S	-0.51	NEU
nsp3_SUD_N	1	43	G	**1.314**	NEU	D	-0.261	NEU
nsp3_SUD_N	1	44	E	-0.195	NEU	I	1.737	MIN
nsp3_SUD_N	1	45	D	**0.767**	NEU	D	-0.753	NEU
nsp3_SUD_N	1	46	M	0.315	NEU	I	1.57	MIN
nsp3_SUD_N	1	47	S	0.191	MIN	T	0.452	NEU
nsp3_SUD_N	1	48	F	-0.147	NEU	F	-1.594	MAX
nsp3_SUD_N	1	49	L	**1.313**	MIN	L	1.368	MIN
nsp3_SUD_N	1	50	E	**0.767**	NEU	K	0.142	NEU
nsp3_SUD_N	1	51	K	0.154	MAX	K	-0.966	NEU
nsp3_SUD_N	1	52	D	0.085	NEU	D	1.355	MIN
nsp3_SUD_N	1	53	A	0.179	NEU	A	0.889	MIN
nsp3_SUD_N	1	54	P	**0.561**	NEU	P	0.018	NEU
nsp3_SUD_N	1	55	Y	**0.905**	NEU	Y	-0.348	NEU
nsp3_SUD_N	1	56	I	**1.109**	MIN	I	1.659	MIN
nsp3_SUD_N	1	57	V	**1.314**	MIN	V	1.249	MIN
nsp3_SUD_N	1	58	G	**1.314**	NEU	G	-0.469	NEU
nsp3_SUD_N	1	59	D	**1.109**	MAX	D	-1.144	MAX
nsp3_SUD_N	1	60	V	**1.314**	MIN	V	1.475	MIN
nsp3_SUD_N	1	61	I	**1.109**	MIN	V	1.403	MIN
nsp3_SUD_N	1	62	T	**0.655**	NEU	Q	-0.923	NEU
nsp3_SUD_N	1	63	S	0.191	NEU	E	-1.239	MAX
nsp3_SUD_N	1	64	G	**0.856**	NEU	G	-0.839	NEU
nsp3_SUD_N	1	65	D	**0.760**	NEU	V	0.911	MIN
nsp3_SUD_N	1	66	I	**1.314**	MIN	L	1.041	MIN
nsp3_SUD_N	1	67	T	**1.314**	NEU	T	-0.374	NEU
nsp3_SUD_N	1	68	C	**0.677**	MIN	A	0.459	NEU
nsp3_SUD_N	1	69	V	**1.314**	MIN	V	1.151	MIN
nsp3_SUD_N	1	70	V	**1.314**	MIN	V	1.355	MIN
nsp3_SUD_N	1	71	I	**1.314**	MIN	I	1.402	MIN
nsp3_SUD_N	1	72	P	**0.677**	NEU	P	-1.067	MAX
nsp3_SUD_N	1	73	A	**0.905**	NEU	T	-0.292	NEU
nsp3_SUD_N	1	74	K	-0.070	NEU	K	-1.273	MAX
nsp3_SUD_N	1	75	K	-0.124	NEU	K	-0.896	NEU
nsp3_SUD_N	1	76	A	0.041	MAX	A	-0.658	NEU
nsp3_SUD_N	1	77	G	**0.969**	NEU	G	0.722	NEU
nsp3_SUD_N	1	78	G	**1.314**	NEU	G	-0.663	NEU
nsp3_SUD_N	1	79	T	**0.856**	NEU	T	-0.021	NEU
nsp3_SUD_N	1	80	T	**0.856**	NEU	T	0.087	NEU
nsp3_SUD_N	1	81	E	0.333	NEU	E	-0.468	NEU
nsp3_SUD_N	1	82	M	**0.760**	MIN	M	0.651	NEU

nsp3_SUD_N	1	83	L	**1.314**	MIN	L	1.028	MIN
nsp3_SUD_N	1	84	A	**0.960**	NEU	A	0.681	NEU
nsp3_SUD_N	1	85	K	**0.856**	NEU	K	-0.826	NEU
nsp3_SUD_N	1	86	A	**1.109**	MIN	A	0.942	MIN
nsp3_SUD_N	1	87	L	**1.314**	MIN	L	1.269	MIN
nsp3_SUD_N	1	88	K	0.445	MAX	R	-0.32	NEU
nsp3_SUD_N	1	89	K	0.376	NEU	K	-1.115	MAX
nsp3_SUD_N	1	90	V	**1.314**	MIN	V	1.278	MIN
nsp3_SUD_N	1	91	P	0.480	NEU	P	-0.392	NEU
nsp3_SUD_N	1	92	V	0.256	MIN	T	-0.558	NEU
nsp3_SUD_N	1	93	D	-0.028	NEU	D	-1.013	MAX
nsp3_SUD_N	1	94	E	**0.561**	NEU	N	-0.888	NEU
nsp3_SUD_N	1	95	Y	0.376	MIN	Y	0.334	NEU
nsp3_SUD_N	1	96	I	**1.109**	MIN	I	1.525	MIN
nsp3_SUD_N	1	97	T	**1.314**	NEU	T	-0.44	NEU
nsp3_SUD_N	1	98	T	**1.109**	NEU	T	-0.259	NEU
nsp3_SUD_N	1	99	Y	0.233	MAX	Y	0.614	NEU
nsp3_SUD_N	1	100	P	0.333	NEU	P	-0.904	NEU
nsp3_SUD_N	1	101	G	**1.109**	NEU	G	-0.195	NEU
nsp3_SUD_N	1	102	Q	**1.109**	NEU	Q	-1.126	MAX
nsp3_SUD_N	1	103	G	0.352	MIN	G	0.549	NEU
nsp3_SUD_N	1	104	C	0.220	MIN	L	1.166	MIN
nsp3_SUD_N	1	105	A	**0.655**	NEU	N	-0.795	NEU
nsp3_SUD_N	1	106	G	0.491	NEU	G	-0.583	NEU
nsp3_SUD_N	1	107	Y	-0.044	MAX	Y	-0.895	NEU
nsp3_SUD_N	1	108	T	0.491	MIN	T	0.837	MIN
nsp3_SUD_N	1	109	L	**0.856**	MIN	V	0.918	MIN
nsp3_SUD_N	1	110	E	0.333	MIN	E	1.544	MIN
nsp3_SUD_N	1	111	E	0.445	NEU	E	-0.118	NEU
nsp3_SUD_N	1	112	A	**0.677**	NEU	A	0.417	NEU
nsp3_SUD_N	1	113	K	**1.109**	NEU	K	-0.784	NEU
nsp3_SUD_N	1	114	T	**0.856**	MIN	T	1.414	MIN
nsp3_SUD_N	1	115	A	**0.969**	MIN	V	1.47	MIN
nsp3_SUD_N	1	116	L	**1.109**	MIN	L	1.45	MIN
nsp3_SUD_N	1	117	K	**0.606**	MAX	K	-1.322	MAX
nsp3_SUD_N	1	118	K	0.315	MAX	K	-1.244	MAX
nsp3_SUD_N	1	119	C	**0.856**	MIN	C	1.774	MIN
nsp3_SUD_N	1	120	K	0.144	MAX	K	-0.727	NEU
nsp3_SUD_N	1	121	S	**1.314**	NEU	S	-0.539	NEU
nsp3_SUD_N	1	122	A	**0.638**	MIN	A	0.733	NEU
nsp3_SUD_N	1	123	F	**1.314**	MIN	F	1.225	MIN
nsp3_SUD_N	1	124	Y	0.376	NEU	Y	0.731	NEU
nsp3_SUD_N	1	125	V	**1.314**	MIN	I	1.372	MIN
nsp3_SUD_N	1	126	L	**1.314**	MIN	L	1.409	MIN
nsp3_SUD_N	1	127	P	**0.969**	NEU	P	-0.859	NEU
nsp3_SUD_N	1	128	S	**1.314**	NEU	S	-0.54	NEU

nsp3_SUD_N	1	129	E	0.220	NEU	I	1.215	MIN
nsp3_SUD_N	1	130	T	0.154	NEU	I	-0.467	NEU
nsp3_SUD_N	1	131	P	0.094	NEU	S	0.255	NEU
nsp3_SUD_N	1	132	N	0.491	NEU	N	-0.882	NEU
nsp3_SUD_N	1	133	A	-0.226	NEU	E	-1.249	MAX
nsp3_SUD_N	1	134	K	0.133	MIN	K	1.598	MIN
nsp3_SUD_N	1	135	E	0.154	MAX	Q	-0.979	NEU
nsp3_SUD_N	1	136	E	0.281	NEU	E	-0.431	NEU
nsp3_SUD_N	1	137	I	0.445	MIN	I	1.356	MIN
nsp3_SUD_N	1	138	L	**0.856**	MIN	L	1.446	MIN
nsp3_SUD_N	1	139	G	**1.109**	NEU	G	-0.622	NEU
nsp3_SUD_N	1	140	T	**1.314**	NEU	T	-0.235	NEU
nsp3_SUD_N	1	141	V	**1.314**	MIN	V	1.698	MIN
nsp3_SUD_N	1	142	S	**0.969**	NEU	S	-0.535	NEU
nsp3_SUD_N	1	143	W	**1.314**	NEU	W	0.173	NEU
nsp3_SUD_N	1	144	N	**1.314**	NEU	N	-0.686	NEU
nsp3_SUD_N	1	145	L	**1.314**	MIN	L	1.236	MIN
nsp3_SUD_N	1	146	R	**1.109**	NEU	R	-0.368	NEU
nsp3_SUD_N	1	147	E	**0.969**	MAX	E	-1.376	MAX
nsp3_SUD_N	1	148	M	**1.109**	NEU	M	0.212	NEU
nsp3_SUD_N	1	149	L	**1.314**	MIN	L	1.398	MIN
nsp3_SUD_N	1	150	A	**1.109**	NEU	A	-0.452	NEU
nsp3_SUD_N	1	151	H	**1.314**	NEU	H	-0.383	NEU
nsp3_SUD_N	1	152	A	**1.314**	NEU	A	0.338	NEU
nsp3_SUD_N	1	153	E	**0.677**	NEU	E	0.543	NEU
nsp3_SUD_N	1	154	E	0.376	NEU	E	0.411	NEU
nsp3_SUD_N	1	155	T	0.352	NEU	T	-0.599	NEU
nsp3_SUD_N	1	156	R	**1.314**	MIN	R	1.761	MIN
nsp3_SUD_N	1	157	K	0.169	NEU	K	-0.056	NEU
nsp3_SUD_N	1	158	L	**1.314**	MIN	L	1.235	MIN
nsp3_SUD_N	1	159	M	**1.314**	MIN	M	0.745	NEU
nsp3_SUD_N	1	160	P	**0.631**	NEU	P	-0.953	NEU
nsp3_SUD_N	1	161	I	**1.314**	MIN	V	1.448	MIN
nsp3_SUD_N	1	162	C	**0.760**	MIN	C	0.674	NEU
nsp3_SUD_N	1	163	M	**1.314**	MIN	V	1.312	MIN
nsp3_SUD_N	1	164	D	**0.760**	NEU	E	0.504	NEU
nsp3_SUD_N	1	165	V	0.445	MIN	T	-0.266	NEU
nsp3_SUD_N	1	166	R	0.445	NEU	K	-1.027	MAX
nsp3_SUD_N	1	167	A	0.407	MIN	A	-0.039	NEU
nsp3_SUD_N	1	168	I	**1.109**	MIN	I	1.988	MIN
nsp3_SUD_N	1	169	M	**0.856**	MIN	V	1.468	MIN
nsp3_SUD_N	1	170	A	**1.109**	NEU	S	-0.591	NEU
nsp3_SUD_N	1	171	T	**1.314**	NEU	T	-0.093	NEU
nsp3_SUD_N	1	172	I	**1.314**	MIN	I	1.702	MIN
nsp3_SUD_N	1	173	Q	**0.655**	MAX	Q	-1.146	MAX
nsp3_SUD_N	1	174	R	**0.677**	NEU	R	-0.916	NEU

nsp3_SUD_N	1	175	K	0.256	MAX	K	-0.569	NEU	
nsp3_SUD_N	1	176	Y	**0.969**	NEU	Y	-0.905	NEU	
nsp3_SUD_N	1	177	K	**1.109**	NEU	K	0.461	NEU	
nsp3_SUD_N	1	178	G	**1.109**	NEU	G	0.238	NEU	
nsp3_SUD_N	1	179	I	**1.109**	MIN	I	2.186	MIN	
nsp3_SUD_N	1	180	K	**0.677**	MAX	K	-1.244	MAX	
nsp3_SUD_N	1	181	I	**1.109**	MIN	I	1.898	MIN	
nsp3_SUD_N	1	182	Q	**0.606**	NEU	Q	-0.752	NEU	
nsp3_SUD_N	1	183	E	**1.109**	MAX	E	-1.075	MAX	
nsp3_SUD_N	1	184	G	**1.314**	NEU	G	-0.097	NEU	
nsp3_SUD_N	1	185	I	**1.109**	MIN	V	0.976	MIN	
nsp3_SUD_N	1	186	V	**1.314**	MIN	V	1.505	MIN	
nsp3_SUD_N	1	187	D	**1.109**	MIN	D	0.736	NEU	
nsp3_SUD_N	1	188	Y	**1.313**	NEU	Y	-0.583	NEU	
nsp3_SUD_N	1	189	G	**0.969**	NEU	G	-0.257	NEU	
nsp3_SUD_N	1	190	V	0.491	MIN	A	-0.139	NEU	
nsp3_SUD_N	1	191	R	**1.314**	NEU	R	0.169	NEU	
nsp3_SUD_N	1	192	F	**1.314**	MIN	F	0.76	NEU	
nsp3_SUD_N	1	193	F	0.352	NEU	Y	-0.067	NEU	
nsp3_SUD_N	1	194	F	**1.314**	MIN	F	1.148	MIN	
nsp3_SUD_N	1	195	Y	0.333	NEU	Y	-0.53	NEU	
nsp3_SUD_N	1	196	T	**1.314**	NEU	T	0.297	NEU	
nsp3_SUD_N	1	197	S	**1.314**	NEU	S	-0.098	NEU	
nsp3_SUD_N	1	198	K	0.333	NEU	K	0.906	MIN	
nsp3_SUD_N	1	199	E	**1.314**	NEU	T	-0.28	NEU	
nsp3_SUD_N	1	200	P	0.376	NEU	T	0.088	NEU	
nsp3_SUD_N	1	201	V	**1.314**	MIN	V	1.074	MIN	
nsp3_SUD_N	1	202	A	**0.544**	NEU	A	0.676	NEU	
nsp3_SUD_N	1	203	S	**1.109**	NEU	S	0.007	NEU	
nsp3_SUD_N	1	204	I	**1.314**	MIN	L	1.253	MIN	
nsp3_SUD_N	1	205	I	**1.314**	MIN	I	1.245	MIN	
nsp3_SUD_N	1	206	T	0.491	NEU	N	1.795	MIN	
nsp3_SUD_N	1	207	K	0.445	MAX	T	-0.136	NEU	
nsp3_SUD_N	1	208	L	**1.314**	MIN	L	1.515	MIN	
nsp3_SUD_N	1	209	N	**0.760**	NEU	N	-0.667	NEU	
nsp3_SUD_N	1	210	S	**0.760**	NEU	D	0.026	NEU	
nsp3_SUD_N	1	211	L	**1.314**	MIN	L	1.427	MIN	
nsp3_SUD_N	1	212	N	0.321	MIN	N	-0.262	NEU	
nsp3_SUD_N	1	213	E	**1.109**	NEU	E	-0.573	NEU	
nsp3_SUD_N	1	214	P	0.376	MAX	T	-0.402	NEU	
nsp3_SUD_N	1	215	L	**1.314**	MIN	L	1.439	MIN	
nsp3_SUD_N	1	216	V	**1.314**	MIN	V	1.17	MIN	
nsp3_SUD_N	1	217	T	**1.314**	NEU	T	-0.323	NEU	
nsp3_SUD_N	1	218	M	**1.314**	NEU	M	0.002	NEU	
nsp3_SUD_N	1	219	P	0.491	NEU	P	-1.001	MAX	
nsp3_SUD_N	1	220	I	**1.314**	MIN	L	1.405	MIN	

nsp3_SUD_N	1	221	G	**1.314**	NEU	G	-0.537	NEU
nsp3_SUD_N	1	222	Y	**1.109**	NEU	Y	-0.195	NEU
nsp3_SUD_N	1	223	V	**1.109**	MIN	V	1.046	MIN
nsp3_SUD_N	1	224	T	**1.314**	NEU	T	-0.137	NEU
nsp3_SUD_N	1	225	H	**1.314**	NEU	H	-0.239	NEU
nsp3_SUD_N	1	226	G	**1.109**	NEU	G	-0.14	NEU
nsp3_SUD_N	1	227	F	**1.314**	MIN	L	1.53	MIN
nsp3_SUD_N	1	228	N	**0.677**	MAX	N	-1.151	MAX
nsp3_SUD_N	1	229	L	**1.314**	MIN	L	1.297	MIN
nsp3_SUD_N	1	230	E	**0.969**	MAX	E	-1.122	MAX
nsp3_SUD_N	1	231	E	**0.856**	MAX	E	-1.146	MAX
nsp3_SUD_N	1	232	A	**1.314**	NEU	A	0.285	NEU
nsp3_SUD_N	1	233	A	**0.606**	NEU	A	0.398	NEU
nsp3_SUD_N	1	234	R	**1.314**	NEU	R	-0.284	NEU
nsp3_SUD_N	1	235	C	**0.856**	MIN	Y	0.575	NEU
nsp3_SUD_N	1	236	M	**1.314**	MIN	M	0.868	MIN
nsp3_SUD_N	1	237	R	**0.606**	NEU	R	-0.932	NEU
nsp3_SUD_N	1	238	S	**1.314**	NEU	S	-0.983	NEU
nsp3_SUD_N	1	239	L	**1.314**	MIN	L	1.615	MIN
nsp3_SUD_N	1	240	K	**1.314**	NEU	K	-0.648	NEU
nsp3_SUD_N	1	241	A	**1.314**	MIN	V	1.101	MIN
nsp3_SUD_N	1	242	P	0.244	NEU	P	-1.202	MAX
nsp3_SUD_N	1	243	A	0.321	MIN	A	0.802	MIN
nsp3_SUD_N	1	244	V	**0.544**	MIN	T	-0.128	NEU
nsp3_SUD_N	1	245	V	**1.109**	MIN	V	1.268	MIN
nsp3_SUD_N	1	246	S	**1.109**	NEU	S	-0.743	NEU
nsp3_SUD_N	1	247	V	**1.314**	MIN	V	1.572	MIN
nsp3_SUD_N	1	248	S	**1.314**	NEU	S	-0.608	NEU
nsp3_SUD_N	1	249	S	**1.314**	NEU	S	-0.135	NEU
nsp3_SUD_N	1	250	P	**0.969**	NEU	P	-0.895	NEU
nsp3_SUD_N	1	251	D	**1.314**	NEU	D	-0.707	NEU
nsp3_SUD_N	1	252	A	**0.969**	NEU	A	0.163	NEU
nsp3_SUD_N	1	253	V	**1.109**	MIN	V	1.554	MIN
nsp3_SUD_N	1	254	T	**0.767**	NEU	T	-0.384	NEU
nsp3_SUD_N	1	255	T	**1.314**	NEU	A	0.313	NEU
nsp3_SUD_N	1	256	Y	**1.109**	NEU	Y	0.465	NEU
nsp3_SUD_N	1	257	N	**1.109**	MAX	N	-1.261	MAX
nsp3_SUD_N	1	258	G	**1.109**	NEU	G	-0.288	NEU
nsp3_SUD_N	1	259	Y	**1.109**	MAX	Y	-2.537	MAX
nsp3_SUD_N	1	260	L	**1.109**	MIN	L	1.023	MIN
nsp3_SUD_N	1	261	T	**0.969**	NEU	T	-0.311	NEU
nsp3_SUD_N	1	262	S	**1.109**	NEU	S	-0.736	NEU
nsp3_SUD_N	1	263	S	**1.314**	NEU	S	-0.07	NEU
nsp3_SUD_N	1	264	S	**1.314**	NEU	S	0.335	NEU
nsp3_SUD_C	1	1	T	0.321	MIN	T	1.567	MIN
nsp3_SUD_C	1	2	P	0.223	NEU	P	-0.569	NEU

nsp3_SUD_C	1	3	E	0.376	MAX	E	-1.165	MAX
nsp3_SUD_C	1	4	E	0.315	NEU	E	-0.884	NEU
nsp3_SUD_C	1	5	H	0.321	NEU	H	0.568	NEU
nsp3_SUD_C	1	6	F	0.407	NEU	F	-0.313	NEU
nsp3_SUD_C	1	7	I	**0.606**	MIN	I	2.013	MIN
nsp3_SUD_C	1	8	E	0.299	MAX	E	-1.853	MAX
nsp3_SUD_C	1	9	T	**1.314**	NEU	T	-0.207	NEU
nsp3_SUD_C	1	10	V	**0.969**	MIN	I	1.739	MIN
nsp3_SUD_C	1	11	S	**0.969**	NEU	S	-0.607	NEU
nsp3_SUD_C	1	12	L	0.491	MIN	L	0.985	MIN
nsp3_SUD_C	1	13	A	-0.155	MIN	A	1.014	MIN
nsp3_SUD_C	1	14	G	**1.314**	NEU	G	-0.377	NEU
nsp3_SUD_C	1	15	S	**0.760**	NEU	S	-0.665	NEU
nsp3_SUD_C	1	16	Y	0.333	MAX	Y	0.001	NEU
nsp3_SUD_C	1	17	R	**0.905**	NEU	K	-0.309	NEU
nsp3_SUD_C	1	18	D	0.191	MIN	D	0.783	MIN
nsp3_SUD_C	1	19	W	0.223	NEU	W	0.451	NEU
nsp3_SUD_C	1	20	S	**0.905**	NEU	S	0.137	NEU
nsp3_SUD_C	1	21	Y	0.054	NEU	Y	-0.564	NEU
nsp3_SUD_C	1	22	S	**1.109**	NEU	S	-0.118	NEU
nsp3_SUD_C	1	23	G	**0.655**	NEU	G	-0.305	NEU
nsp3_SUD_C	1	24	Q	**0.767**	NEU	Q	-0.413	NEU
nsp3_SUD_C	1	25	R	0.256	MIN	S	-0.739	NEU
nsp3_SUD_C	1	26	T	**0.856**	NEU	T	0.457	NEU
nsp3_SUD_C	1	27	E	0.376	MIN	Q	0.553	NEU
nsp3_SUD_C	1	28	L	**0.760**	MIN	L	0.564	NEU
nsp3_SUD_C	1	29	G	**0.561**	NEU	G	0.088	NEU
nsp3_SUD_C	1	30	V	**1.109**	MIN	I	2.151	MIN
nsp3_SUD_C	1	31	E	0.352	NEU	E	-0.77	NEU
nsp3_SUD_C	1	32	F	0.445	MIN	F	0.787	MIN
nsp3_SUD_C	1	33	L	**1.109**	MIN	L	1.539	MIN
nsp3_SUD_C	1	34	K	**0.677**	MAX	K	-0.863	NEU
nsp3_SUD_C	1	35	R	**0.760**	NEU	R	0.603	NEU
nsp3_SUD_C	1	36	G	**1.109**	NEU	G	0.066	NEU
nsp3_SUD_C	1	37	D	**1.314**	MIN	D	1.415	MIN
nsp3_SUD_C	1	38	K	**1.109**	MAX	K	-1.908	MAX
nsp3_SUD_C	1	39	I	0.480	MIN	S	-0.103	NEU
nsp3_SUD_C	1	40	V	**1.109**	MIN	V	1.997	MIN
nsp3_SUD_C	1	41	Y	0.352	MAX	Y	-0.171	NEU
nsp3_SUD_C	1	42	H	**0.905**	NEU	Y	-0.273	NEU
nsp3_SUD_C	1	43	L	**0.969**	NEU	T	0.224	NEU
nsp3_SUD_C	1	44	E	0.256	NEU	S	0.262	NEU
nsp3_SUD_C	1	45	S	0.480	NEU	N	0.596	NEU
nsp3_SUD_C	1	46	P	**0.752**	NEU	P	-0.096	NEU
nsp3_SUD_C	1	47	I	**0.844**	MIN	T	0.639	NEU
nsp3_SUD_C	1	48	E	0.445	NEU	T	0.086	NEU

nsp3_SUD_C	1	49	F	**0.544**	MIN	F	0.997	MIN
nsp3_SUD_C	1	50	H	**0.969**	NEU	H	-0.032	NEU
nsp3_SUD_C	1	51	L	**0.969**	MIN	L	1.467	MIN
nsp3_SUD_C	1	52	D	0.491	NEU	D	0.47	NEU
nsp3_SUD_C	1	53	G	**1.109**	NEU	G	-0.412	NEU
nsp3_SUD_C	1	54	E	**0.856**	MAX	E	-1.628	MAX
nsp3_SUD_C	1	55	V	**0.969**	MIN	V	1.04	MIN
nsp3_SUD_C	1	56	L	**0.969**	MIN	I	1.786	MIN
nsp3_SUD_C	1	57	P	-0.226	NEU	T	0.979	MIN
nsp3_SUD_C	1	58	L	**0.655**	MIN	F	0.454	NEU
nsp3_SUD_C	1	59	D	**0.856**	MIN	D	0.592	NEU
nsp3_SUD_C	1	60	K	**0.760**	MAX	N	-1.139	MAX
nsp3_SUD_C	1	61	L	**1.109**	MIN	L	1.533	MIN
nsp3_SUD_C	1	62	K	0.376	MAX	K	-0.928	NEU
nsp3_SUD_C	1	63	S	0.407	NEU	T	0.766	NEU
nsp3_SUD_C	1	64	L	**0.760**	MIN	L	0.959	MIN
nsp3_SUD_C	1	65	L	**1.109**	MIN	L	1.545	MIN
nsp3_SUD_C	1	66	S	**1.313**	NEU	S	0.086	NEU
nsp3_PLPro	1	1	I	**1.314**	MIN	I	0.83	MIN
nsp3_PLPro	1	2	K	**1.109**	MAX	K	-1.119	MAX
nsp3_PLPro	1	3	V	**1.314**	MIN	V	1.02	MIN
nsp3_PLPro	1	4	F	**1.109**	NEU	F	0.052	NEU
nsp3_PLPro	1	5	T	**1.314**	NEU	T	-0.479	NEU
nsp3_PLPro	1	6	T	**1.314**	NEU	T	-0.082	NEU
nsp3_PLPro	1	7	V	**1.109**	MIN	V	1.927	MIN
nsp3_PLPro	1	8	D	0.480	NEU	D	-0.65	NEU
nsp3_PLPro	1	9	N	**1.314**	NEU	N	-0.828	NEU
nsp3_PLPro	1	10	T	**0.856**	MIN	I	0.975	MIN
nsp3_PLPro	1	11	N	**1.109**	NEU	N	-0.256	NEU
nsp3_PLPro	1	12	L	**1.314**	MIN	L	1.153	MIN
nsp3_PLPro	1	13	H	0.491	MAX	H	-1.08	MAX
nsp3_PLPro	1	14	T	**1.314**	NEU	T	-0.584	NEU
nsp3_PLPro	1	15	Q	**0.856**	NEU	Q	-0.952	NEU
nsp3_PLPro	1	16	I	**1.314**	MIN	V	1.356	MIN
nsp3_PLPro	1	17	V	**1.314**	MIN	V	1.694	MIN
nsp3_PLPro	1	18	D	0.352	MIN	D	0.942	MIN
nsp3_PLPro	1	19	M	0.074	NEU	M	-1.577	MAX
nsp3_PLPro	1	20	S	**1.109**	MIN	S	0.828	MIN
nsp3_PLPro	1	21	M	**1.109**	MAX	M	-1.53	MAX
nsp3_PLPro	1	22	T	**1.109**	NEU	T	-0.252	NEU
nsp3_PLPro	1	23	Y	**0.856**	MIN	Y	0.713	NEU
nsp3_PLPro	1	24	G	**1.314**	NEU	G	-0.396	NEU
nsp3_PLPro	1	25	Q	**1.314**	NEU	Q	0.262	NEU
nsp3_PLPro	1	26	Q	**0.856**	NEU	Q	-0.763	NEU
nsp3_PLPro	1	27	F	**1.314**	MIN	F	0.757	NEU
nsp3_PLPro	1	28	G	**1.314**	NEU	G	-0.685	NEU

nsp3_PLPro	1	29	P	**1.314**	NEU	P	0.364	NEU
nsp3_PLPro	1	30	T	**0.856**	NEU	T	-0.142	NEU
nsp3_PLPro	1	31	Y	**0.856**	NEU	Y	-0.125	NEU
nsp3_PLPro	1	32	L	**1.314**	MIN	L	1.165	MIN
nsp3_PLPro	1	33	D	**0.969**	NEU	D	-0.778	NEU
nsp3_PLPro	1	34	G	**1.314**	NEU	G	-0.362	NEU
nsp3_PLPro	1	35	A	0.144	MIN	A	0.606	NEU
nsp3_PLPro	1	36	D	**0.905**	NEU	D	0.352	NEU
nsp3_PLPro	1	37	V	**1.314**	MIN	V	1.415	MIN
nsp3_PLPro	1	38	T	**1.314**	NEU	T	-0.072	NEU
nsp3_PLPro	1	39	K	**1.314**	NEU	K	0.387	NEU
nsp3_PLPro	1	40	I	**1.109**	MIN	I	1.656	MIN
nsp3_PLPro	1	41	K	**1.314**	NEU	K	0.251	NEU
nsp3_PLPro	1	42	P	**0.544**	NEU	P	-0.467	NEU
nsp3_PLPro	1	43	H	0.352	NEU	H	0.41	NEU
nsp3_PLPro	1	44	V	-0.084	MIN	N	-1.48	MAX
nsp3_PLPro	1	45	N	**0.969**	NEU	S	-0.026	NEU
nsp3_PLPro	1	46	H	**1.314**	NEU	H	-0.336	NEU
nsp3_PLPro	1	47	E	**1.109**	MIN	E	1.626	MIN
nsp3_PLPro	1	48	G	**0.969**	NEU	G	-0.474	NEU
nsp3_PLPro	1	49	K	0.445	MAX	K	-1.091	MAX
nsp3_PLPro	1	50	T	**1.109**	NEU	T	-0.135	NEU
nsp3_PLPro	1	51	F	**1.314**	MIN	F	1.289	MIN
nsp3_PLPro	1	52	F	**0.905**	MIN	Y	0.878	MIN
nsp3_PLPro	1	53	V	**1.314**	MIN	V	1.104	MIN
nsp3_PLPro	1	54	L	**1.109**	MIN	L	1.14	MIN
nsp3_PLPro	1	55	P	**0.677**	MAX	P	-1.435	MAX
nsp3_PLPro	1	56	S	0.445	NEU	N	0.06	NEU
nsp3_PLPro	1	57	D	0.445	NEU	D	-0.896	NEU
nsp3_PLPro	1	58	D	**0.969**	MIN	D	1.785	MIN
nsp3_PLPro	1	59	T	**0.760**	MIN	T	0.921	MIN
nsp3_PLPro	1	60	L	**1.314**	MIN	L	1.058	MIN
nsp3_PLPro	1	61	R	**1.109**	MIN	R	1.593	MIN
nsp3_PLPro	1	62	S	**0.677**	NEU	V	1.714	MIN
nsp3_PLPro	1	63	E	0.445	MAX	E	-0.962	NEU
nsp3_PLPro	1	64	A	**0.969**	NEU	A	0.5	NEU
nsp3_PLPro	1	65	F	**0.856**	NEU	F	-0.155	NEU
nsp3_PLPro	1	66	E	**1.109**	NEU	E	-0.886	NEU
nsp3_PLPro	1	67	Y	**0.631**	MAX	Y	-1.651	MAX
nsp3_PLPro	1	68	Y	**0.969**	NEU	Y	-0.765	NEU
nsp3_PLPro	1	69	H	**1.109**	NEU	H	-0.085	NEU
nsp3_PLPro	1	70	T	**1.109**	NEU	T	-0.633	NEU
nsp3_PLPro	1	71	L	**0.606**	NEU	T	-0.339	NEU
nsp3_PLPro	1	72	D	**1.314**	NEU	D	0.014	NEU
nsp3_PLPro	1	73	E	**0.856**	NEU	P	0.019	NEU
nsp3_PLPro	1	74	S	**1.109**	MIN	S	1.542	MIN

nsp3_PLPro	1	75	F	1.109	MIN	F	1.038	MIN
nsp3_PLPro	1	76	L	1.314	MIN	L	1.524	MIN
nsp3_PLPro	1	77	G	1.314	NEU	G	0.159	NEU
nsp3_PLPro	1	78	R	1.314	NEU	R	-0.011	NEU
nsp3_PLPro	1	79	Y	1.314	NEU	Y	0.05	NEU
nsp3_PLPro	1	80	M	0.767	MIN	M	0.851	MIN
nsp3_PLPro	1	81	S	1.314	NEU	S	-0.469	NEU
nsp3_PLPro	1	82	A	1.314	NEU	A	0.458	NEU
nsp3_PLPro	1	83	L	1.314	MIN	L	1.397	MIN
nsp3_PLPro	1	84	N	1.314	NEU	N	-0.334	NEU
nsp3_PLPro	1	85	H	1.314	NEU	H	-0.398	NEU
nsp3_PLPro	1	86	T	1.109	NEU	T	-0.305	NEU
nsp3_PLPro	1	87	K	1.109	NEU	K	-0.886	NEU
nsp3_PLPro	1	88	K	0.760	MIN	K	1.084	MIN
nsp3_PLPro	1	89	W	0.491	NEU	W	-1.088	MAX
nsp3_PLPro	1	90	K	1.109	MAX	K	-1.15	MAX
nsp3_PLPro	1	91	F	0.445	MIN	Y	-1.22	MAX
nsp3_PLPro	1	92	P	1.109	MAX	P	-1.635	MAX
nsp3_PLPro	1	93	Q	1.314	MAX	Q	-1.338	MAX
nsp3_PLPro	1	94	V	1.314	MIN	V	1.226	MIN
nsp3_PLPro	1	95	G	0.544	NEU	N	-1.074	MAX
nsp3_PLPro	1	96	G	1.314	NEU	G	-0.64	NEU
nsp3_PLPro	1	97	L	1.314	MIN	L	1.288	MIN
nsp3_PLPro	1	98	T	1.109	NEU	T	-0.267	NEU
nsp3_PLPro	1	99	S	0.969	NEU	S	-0.691	NEU
nsp3_PLPro	1	100	I	1.314	MIN	I	2.035	MIN
nsp3_PLPro	1	101	K	1.109	MAX	K	-1.319	MAX
nsp3_PLPro	1	102	W	1.314	MAX	W	-1.646	MAX
nsp3_PLPro	1	103	A	0.905	NEU	A	-0.857	NEU
nsp3_PLPro	1	104	D	1.314	NEU	D	0.148	NEU
nsp3_PLPro	1	105	N	0.856	MAX	N	-1.024	MAX
nsp3_PLPro	1	106	N	1.109	NEU	N	-0.79	NEU
nsp3_PLPro	1	107	C	1.314	MIN	C	2.011	MIN
nsp3_PLPro	1	108	Y	0.445	MIN	Y	0.506	NEU
nsp3_PLPro	1	109	L	1.314	MIN	L	1.538	MIN
nsp3_PLPro	1	110	S	1.314	NEU	A	0.454	NEU
nsp3_PLPro	1	111	S	1.314	NEU	T	-0.264	NEU
nsp3_PLPro	1	112	V	0.606	MIN	A	0.558	NEU
nsp3_PLPro	1	113	L	1.314	MIN	L	1.362	MIN
nsp3_PLPro	1	114	L	1.314	MIN	L	1.366	MIN
nsp3_PLPro	1	115	A	1.109	NEU	T	-0.02	NEU
nsp3_PLPro	1	116	L	1.314	MIN	L	1.392	MIN
nsp3_PLPro	1	117	Q	0.352	MAX	Q	-0.859	NEU
nsp3_PLPro	1	118	Q	1.109	NEU	Q	-0.759	NEU
nsp3_PLPro	1	119	I	1.314	MIN	I	1.588	MIN
nsp3_PLPro	1	120	E	1.109	MIN	E	1.422	MIN

nsp3_PLPro	1	121	V	**1.314**	MIN	L	1.368	MIN
nsp3_PLPro	1	122	K	0.407	MIN	K	0.266	NEU
nsp3_PLPro	1	123	F	**1.109**	MIN	F	1.058	MIN
nsp3_PLPro	1	124	N	0.333	NEU	N	-0.021	NEU
nsp3_PLPro	1	125	A	**1.314**	NEU	P	-0.804	NEU
nsp3_PLPro	1	126	P	**1.314**	NEU	P	-0.307	NEU
nsp3_PLPro	1	127	A	0.445	NEU	A	-0.131	NEU
nsp3_PLPro	1	128	L	**1.314**	MIN	L	1.407	MIN
nsp3_PLPro	1	129	Q	**0.606**	MAX	Q	-1.01	MAX
nsp3_PLPro	1	130	E	**0.677**	NEU	D	-0.817	NEU
nsp3_PLPro	1	131	A	**1.314**	MIN	A	0.737	NEU
nsp3_PLPro	1	132	Y	**1.314**	NEU	Y	-0.938	NEU
nsp3_PLPro	1	133	Y	**1.109**	MAX	Y	-2.306	MAX
nsp3_PLPro	1	134	R	**1.314**	NEU	R	0.036	NEU
nsp3_PLPro	1	135	A	**1.314**	NEU	A	0.51	NEU
nsp3_PLPro	1	136	R	**1.314**	NEU	R	-0.024	NEU
nsp3_PLPro	1	137	A	**1.109**	NEU	A	0.254	NEU
nsp3_PLPro	1	138	G	**1.314**	NEU	G	-0.253	NEU
nsp3_PLPro	1	139	D	**1.109**	NEU	E	-0.736	NEU
nsp3_PLPro	1	140	A	**0.856**	MIN	A	0.587	NEU
nsp3_PLPro	1	141	A	**1.314**	NEU	A	0.189	NEU
nsp3_PLPro	1	142	N	**0.969**	NEU	N	-0.863	NEU
nsp3_PLPro	1	143	F	**1.109**	MIN	F	0.674	NEU
nsp3_PLPro	1	144	C	**1.314**	MIN	C	1.382	MIN
nsp3_PLPro	1	145	A	**1.109**	NEU	A	0.433	NEU
nsp3_PLPro	1	146	L	**1.314**	MIN	L	1.364	MIN
nsp3_PLPro	1	147	I	**1.109**	MIN	I	1.444	MIN
nsp3_PLPro	1	148	L	**1.314**	MIN	L	1.732	MIN
nsp3_PLPro	1	149	A	**0.677**	NEU	A	0.569	NEU
nsp3_PLPro	1	150	Y	**0.969**	MAX	Y	-1.764	MAX
nsp3_PLPro	1	151	S	0.256	NEU	C	0.585	NEU
nsp3_PLPro	1	152	N	**0.767**	NEU	N	-0.065	NEU
nsp3_PLPro	1	153	K	**1.314**	NEU	K	-0.832	NEU
nsp3_PLPro	1	154	T	**0.655**	NEU	T	0.247	NEU
nsp3_PLPro	1	155	V	**0.969**	MIN	V	1.18	MIN
nsp3_PLPro	1	156	G	**1.314**	NEU	G	-0.317	NEU
nsp3_PLPro	1	157	E	0.350	NEU	E	-1.145	MAX
nsp3_PLPro	1	158	L	**1.314**	NEU	L	0.126	NEU
nsp3_PLPro	1	159	G	0.376	MAX	G	-1.288	MAX
nsp3_PLPro	1	160	D	**1.314**	NEU	D	-0.627	NEU
nsp3_PLPro	1	161	V	**1.109**	MIN	V	1.305	MIN
nsp3_PLPro	1	162	R	**0.544**	MIN	R	0.941	MIN
nsp3_PLPro	1	163	E	-0.004	NEU	E	-0.757	NEU
nsp3_PLPro	1	164	T	**1.314**	NEU	T	-0.427	NEU
nsp3_PLPro	1	165	M	**0.677**	NEU	M	0.54	NEU
nsp3_PLPro	1	166	T	**0.561**	NEU	S	-0.694	NEU

nsp3_PLPro	1	167	H	**1.314**	NEU	Y	-0.653	NEU
nsp3_PLPro	1	168	L	**1.314**	MIN	L	1.534	MIN
nsp3_PLPro	1	169	L	**1.314**	MIN	F	1.333	MIN
nsp3_PLPro	1	170	Q	**1.109**	MAX	Q	-1.171	MAX
nsp3_PLPro	1	171	H	**1.314**	NEU	H	0.253	NEU
nsp3_PLPro	1	172	A	0.445	MIN	A	0.755	NEU
nsp3_PLPro	1	173	N	**1.314**	MIN	N	1.715	MIN
nsp3_PLPro	1	174	L	**1.314**	MIN	L	1.378	MIN
nsp3_PLPro	1	175	E	-0.177	NEU	D	2.217	MIN
nsp3_PLPro	1	176	S	**1.109**	NEU	S	0.429	NEU
nsp3_PLPro	1	177	A	0.299	NEU	C	2.423	MIN
nsp3_PLPro	1	178	K	**0.969**	NEU	K	-0.571	NEU
nsp3_PLPro	1	179	R	**0.760**	NEU	R	0.555	NEU
nsp3_PLPro	1	180	V	**0.544**	MIN	V	0.53	NEU
nsp3_PLPro	1	181	L	**1.314**	MIN	L	1.214	MIN
nsp3_PLPro	1	182	N	0.365	MAX	N	-0.872	NEU
nsp3_PLPro	1	183	V	**1.314**	MIN	V	0.872	MIN
nsp3_PLPro	1	184	V	**1.109**	MIN	V	0.551	NEU
nsp3_PLPro	1	185	C	**1.314**	MIN	C	1.929	MIN
nsp3_PLPro	1	186	K	**0.969**	MIN	K	0.796	MIN
nsp3_PLPro	1	187	H	0.350	NEU	T	-1.035	MAX
nsp3_PLPro	1	188	C	**1.109**	MIN	C	1.405	MIN
nsp3_PLPro	1	189	G	**1.314**	NEU	G	-0.535	NEU
nsp3_PLPro	1	190	Q	**1.109**	NEU	Q	0.23	NEU
nsp3_PLPro	1	191	K	**1.314**	NEU	Q	0.09	NEU
nsp3_PLPro	1	192	T	**0.760**	NEU	Q	-0.722	NEU
nsp3_PLPro	1	193	T	**1.314**	NEU	T	-0.026	NEU
nsp3_PLPro	1	194	T	0.491	MIN	T	0.46	NEU
nsp3_PLPro	1	195	L	**1.109**	MIN	L	1.568	MIN
nsp3_PLPro	1	196	T	**0.677**	NEU	K	0.143	NEU
nsp3_PLPro	1	197	G	**1.314**	NEU	G	-0.444	NEU
nsp3_PLPro	1	198	V	**1.314**	MIN	V	1.313	MIN
nsp3_PLPro	1	199	E	0.333	NEU	E	-0.587	NEU
nsp3_PLPro	1	200	A	**1.314**	NEU	A	0.238	NEU
nsp3_PLPro	1	201	V	**1.314**	MIN	V	1.247	MIN
nsp3_PLPro	1	202	M	0.299	NEU	M	-0.964	NEU
nsp3_PLPro	1	203	Y	**0.856**	NEU	Y	0.038	NEU
nsp3_PLPro	1	204	M	**0.969**	MAX	M	-1.395	MAX
nsp3_PLPro	1	205	G	**1.109**	NEU	G	-0.086	NEU
nsp3_PLPro	1	206	T	**1.314**	NEU	T	-0.243	NEU
nsp3_PLPro	1	207	L	**1.109**	MIN	L	1.201	MIN
nsp3_PLPro	1	208	S	**1.314**	NEU	S	-0.393	NEU
nsp3_PLPro	1	209	Y	**0.856**	NEU	Y	0.451	NEU
nsp3_PLPro	1	210	D	**0.544**	MIN	E	1.226	MIN
nsp3_PLPro	1	211	N	0.352	NEU	Q	-0.843	NEU
nsp3_PLPro	1	212	L	**1.314**	MIN	F	0.987	MIN

nsp3_PLPro	1	213	K	**1.109**	NEU	K	-0.235	NEU
nsp3_PLPro	1	214	T	**0.969**	NEU	K	-0.366	NEU
nsp3_PLPro	1	215	G	**1.314**	NEU	G	-0.461	NEU
nsp3_PLPro	1	216	V	**1.109**	MIN	V	1.589	MIN
nsp3_PLPro	1	217	S	**0.561**	MIN	Q	0.411	NEU
nsp3_PLPro	1	218	I	**1.109**	MIN	I	2.553	MIN
nsp3_PLPro	1	219	P	0.445	NEU	P	0.423	NEU
nsp3_PLPro	1	220	C	**1.314**	MIN	C	1.407	MIN
nsp3_PLPro	1	221	V	**0.767**	NEU	T	-0.88	NEU
nsp3_PLPro	1	222	C	**1.314**	MIN	C	1.415	MIN
nsp3_PLPro	1	223	G	**1.314**	NEU	G	-0.233	NEU
nsp3_PLPro	1	224	R	0.256	NEU	K	-1.29	MAX
nsp3_PLPro	1	225	D	**0.969**	MIN	Q	1.171	MIN
nsp3_PLPro	1	226	A	**1.109**	NEU	A	0.144	NEU
nsp3_PLPro	1	227	T	**1.314**	NEU	T	0.102	NEU
nsp3_PLPro	1	228	Q	**0.905**	NEU	K	-1.325	MAX
nsp3_PLPro	1	229	Y	0.321	MAX	Y	-1.132	MAX
nsp3_PLPro	1	230	L	**1.109**	MIN	L	1.247	MIN
nsp3_PLPro	1	231	V	**1.109**	MIN	V	1.273	MIN
nsp3_PLPro	1	232	Q	**0.856**	NEU	Q	0.25	NEU
nsp3_PLPro	1	233	Q	**0.905**	NEU	Q	-0.474	NEU
nsp3_PLPro	1	234	E	**1.109**	NEU	E	0.172	NEU
nsp3_PLPro	1	235	S	**1.109**	NEU	S	-0.593	NEU
nsp3_PLPro	1	236	S	**1.314**	NEU	P	-0.438	NEU
nsp3_PLPro	1	237	F	**1.314**	MIN	F	1.243	MIN
nsp3_PLPro	1	238	V	**1.314**	MIN	V	1.385	MIN
nsp3_PLPro	1	239	M	**1.314**	MIN	M	0.846	MIN
nsp3_PLPro	1	240	M	**1.314**	MIN	M	0.88	MIN
nsp3_PLPro	1	241	S	**1.314**	NEU	S	-0.579	NEU
nsp3_PLPro	1	242	A	**0.561**	MIN	A	0.57	NEU
nsp3_PLPro	1	243	P	**1.109**	NEU	P	-0.627	NEU
nsp3_PLPro	1	244	P	**1.109**	NEU	P	-0.769	NEU
nsp3_PLPro	1	245	A	**1.109**	NEU	A	0.096	NEU
nsp3_PLPro	1	246	E	**0.544**	NEU	Q	-0.668	NEU
nsp3_PLPro	1	247	Y	**1.109**	NEU	Y	-0.316	NEU
nsp3_PLPro	1	248	K	0.333	NEU	E	1.712	MIN
nsp3_PLPro	1	249	L	**1.109**	MIN	L	1.543	MIN
nsp3_PLPro	1	250	Q	**0.631**	NEU	K	0.134	NEU
nsp3_PLPro	1	251	Q	**1.314**	NEU	H	0.229	NEU
nsp3_PLPro	1	252	G	**1.109**	NEU	G	-0.357	NEU
nsp3_PLPro	1	253	T	**0.606**	NEU	T	-1.152	MAX
nsp3_PLPro	1	254	F	**1.314**	MIN	F	1.349	MIN
nsp3_PLPro	1	255	L	**1.109**	MIN	T	-0.381	NEU
nsp3_PLPro	1	256	C	**1.314**	MIN	C	1.226	MIN
nsp3_PLPro	1	257	A	**0.606**	NEU	A	0.679	NEU
nsp3_PLPro	1	258	N	**1.314**	NEU	S	-0.582	NEU

nsp3_PLPro	1	259	E	**0.856**	MAX	E	-1.103	MAX
nsp3_PLPro	1	260	Y	**0.631**	NEU	Y	-0.675	NEU
nsp3_PLPro	1	261	T	**1.109**	NEU	T	-0.061	NEU
nsp3_PLPro	1	262	G	**0.856**	MIN	G	0.635	NEU
nsp3_PLPro	1	263	N	**0.905**	NEU	N	-0.204	NEU
nsp3_PLPro	1	264	Y	0.491	NEU	Y	-1.079	MAX
nsp3_PLPro	1	265	Q	**0.969**	NEU	Q	-0.325	NEU
nsp3_PLPro	1	266	C	**1.109**	MIN	C	1.806	MIN
nsp3_PLPro	1	267	G	**1.314**	NEU	G	0.015	NEU
nsp3_PLPro	1	268	H	**1.314**	NEU	H	0.465	NEU
nsp3_PLPro	1	269	Y	**0.677**	NEU	Y	-0.833	NEU
nsp3_PLPro	1	270	T	**1.109**	NEU	K	-0.375	NEU
nsp3_PLPro	1	271	H	**1.314**	NEU	H	-0.597	NEU
nsp3_PLPro	1	272	I	**1.314**	MIN	I	1.497	MIN
nsp3_PLPro	1	273	T	**0.969**	NEU	T	-0.233	NEU
nsp3_PLPro	1	274	A	0.349	NEU	S	-0.5	NEU
nsp3_PLPro	1	275	K	0.352	NEU	K	-1.028	MAX
nsp3_PLPro	1	276	E	0.037	MIN	E	-0.073	NEU
nsp3_PLPro	1	277	T	**0.969**	NEU	T	0.248	NEU
nsp3_PLPro	1	278	L	**1.314**	MIN	L	0.832	MIN
nsp3_PLPro	1	279	Y	**1.314**	NEU	Y	-0.74	NEU
nsp3_PLPro	1	280	R	0.174	NEU	C	0.915	MIN
nsp3_PLPro	1	281	I	**1.314**	MIN	I	1.43	MIN
nsp3_PLPro	1	282	D	0.321	MIN	D	-0.227	NEU
nsp3_PLPro	1	283	G	**1.314**	NEU	G	-0.373	NEU
nsp3_PLPro	1	284	A	**1.314**	NEU	A	0.196	NEU
nsp3_PLPro	1	285	H	0.220	NEU	L	1.079	MIN
nsp3_PLPro	1	286	L	**1.314**	MIN	L	0.981	MIN
nsp3_PLPro	1	287	T	**1.314**	NEU	T	-0.28	NEU
nsp3_PLPro	1	288	K	0.407	MAX	K	0.09	NEU
nsp3_PLPro	1	289	M	0.491	MIN	S	-0.531	NEU
nsp3_PLPro	1	290	S	**0.677**	NEU	S	-0.096	NEU
nsp3_PLPro	1	291	E	**1.109**	MIN	E	1.257	MIN
nsp3_PLPro	1	292	Y	**0.767**	NEU	Y	-0.314	NEU
nsp3_PLPro	1	293	K	0.256	NEU	K	0.745	NEU
nsp3_PLPro	1	294	G	**1.314**	NEU	G	-0.364	NEU
nsp3_PLPro	1	295	P	**0.767**	MAX	P	-1.496	MAX
nsp3_PLPro	1	296	V	**1.314**	MIN	I	1.225	MIN
nsp3_PLPro	1	297	T	**1.314**	NEU	T	-0.438	NEU
nsp3_PLPro	1	298	D	0.350	MAX	D	-0.972	NEU
nsp3_PLPro	1	299	V	**1.109**	MIN	V	1.324	MIN
nsp3_PLPro	1	300	F	**1.314**	MIN	F	1.383	MIN
nsp3_PLPro	1	301	Y	**1.314**	MIN	Y	0.702	NEU
nsp3_PLPro	1	302	K	**1.109**	NEU	K	-0.776	NEU
nsp3_PLPro	1	303	E	**1.109**	MAX	E	-1.209	MAX
nsp3_PLPro	1	304	T	**0.969**	NEU	N	-0.123	NEU

nsp3_PLPro	1	305	S	**1.109**	NEU	S	0.492	NEU
nsp3_PLPro	1	306	Y	**1.109**	MAX	Y	-2.668	MAX
nsp3_PLPro	1	307	T	0.220	NEU	T	0.342	NEU
nsp3_PLPro	1	308	T	**1.314**	NEU	T	0.022	NEU
nsp3_PLPro	1	309	T	0.352	MIN	T	0.888	MIN
nsp3_NAB	1	1	Y	**1.309**	MAX	F	-1.332	MAX
nsp3_NAB	1	2	T	**1.087**	NEU	T	0.065	NEU
nsp3_NAB	1	3	E	0.346	NEU	E	-1.118	MAX
nsp3_NAB	1	4	Q	**1.087**	NEU	Q	-0.459	NEU
nsp3_NAB	1	5	P	**1.309**	NEU	P	0.157	NEU
nsp3_NAB	1	6	I	**1.309**	MIN	I	1.638	MIN
nsp3_NAB	1	7	D	**0.602**	NEU	D	-0.864	NEU
nsp3_NAB	1	8	L	**1.309**	MIN	L	1.483	MIN
nsp3_NAB	1	9	V	**0.938**	MIN	V	0.837	MIN
nsp3_NAB	1	10	P	0.369	NEU	P	-0.95	NEU
nsp3_NAB	1	11	T	**1.309**	NEU	N	-0.75	NEU
nsp3_NAB	1	12	Q	**1.309**	NEU	Q	-0.489	NEU
nsp3_NAB	1	13	P	**1.309**	NEU	P	-0.376	NEU
nsp3_NAB	1	14	L	0.446	NEU	Y	-2.936	MAX
nsp3_NAB	1	15	P	**0.938**	NEU	P	0.453	NEU
nsp3_NAB	1	16	N	0.313	NEU	N	-0.762	NEU
nsp3_NAB	1	17	A	0.170	MAX	A	-1.134	MAX
nsp3_NAB	1	18	S	**1.309**	NEU	S	-0.341	NEU
nsp3_NAB	1	19	F	0.363	MIN	F	0.566	NEU
nsp3_NAB	1	20	D	**0.718**	NEU	D	-0.766	NEU
nsp3_NAB	1	21	N	**0.938**	NEU	N	-0.438	NEU
nsp3_NAB	1	22	F	**1.309**	MIN	F	1.232	MIN
nsp3_NAB	1	23	K	**1.087**	NEU	K	-0.682	NEU
nsp3_NAB	1	24	L	**1.309**	MIN	F	0.891	MIN
nsp3_NAB	1	25	T	**0.560**	MIN	V	1.813	MIN
nsp3_NAB	1	26	C	**1.087**	MIN	C	1.452	MIN
nsp3_NAB	1	27	S	**1.309**	NEU	D	-0.799	NEU
nsp3_NAB	1	28	N	**0.938**	NEU	N	-0.454	NEU
nsp3_NAB	1	29	T	0.170	NEU	I	2.187	MIN
nsp3_NAB	1	30	K	**1.087**	MIN	K	1.388	MIN
nsp3_NAB	1	31	F	**1.309**	MIN	F	1.493	MIN
nsp3_NAB	1	32	A	**1.309**	MIN	A	1.075	MIN
nsp3_NAB	1	33	D	0.131	MIN	D	-0.299	NEU
nsp3_NAB	1	34	D	**0.818**	NEU	D	-0.889	NEU
nsp3_NAB	1	35	L	**1.309**	MIN	L	1.25	MIN
nsp3_NAB	1	36	N	**1.309**	NEU	N	-0.857	NEU
nsp3_NAB	1	37	Q	**0.938**	NEU	Q	-0.567	NEU
nsp3_NAB	1	38	M	**0.867**	MIN	L	1.13	MIN
nsp3_NAB	1	39	T	**0.938**	NEU	T	0.03	NEU
nsp3_NAB	1	40	G	**1.087**	NEU	G	-0.457	NEU
nsp3_NAB	1	41	F	-0.029	NEU	Y	-2.077	MAX

nsp3_NAB	1	42	K	0.404	MIN	K	0.989	MIN
nsp3_NAB	1	43	K	**0.560**	MAX	K	-0.841	NEU
nsp3_NAB	1	44	P	0.111	MIN	P	0.847	MIN
nsp3_NAB	1	45	A	**1.087**	NEU	A	0.446	NEU
nsp3_NAB	1	46	S	0.404	NEU	S	0.813	MIN
nsp3_NAB	1	47	R	0.404	MIN	R	0.603	NEU
nsp3_NAB	1	48	E	**1.087**	NEU	E	-0.13	NEU
nsp3_NAB	1	49	L	**1.309**	MIN	L	1.111	MIN
nsp3_NAB	1	50	S	**0.632**	NEU	K	-0.28	NEU
nsp3_NAB	1	51	V	**1.309**	MIN	V	1.465	MIN
nsp3_NAB	1	52	T	**1.309**	NEU	T	-0.547	NEU
nsp3_NAB	1	53	F	**1.309**	MIN	F	0.944	MIN
nsp3_NAB	1	54	F	**1.087**	MIN	F	0.894	MIN
nsp3_NAB	1	55	P	**1.309**	NEU	P	-0.698	NEU
nsp3_NAB	1	56	D	0.446	MAX	D	-0.943	NEU
nsp3_NAB	1	57	L	**1.309**	MIN	L	1.159	MIN
nsp3_NAB	1	58	N	**0.720**	NEU	N	0.405	NEU
nsp3_NAB	1	59	G	**1.309**	NEU	G	-0.875	NEU
nsp3_NAB	1	60	D	0.124	NEU	D	0.513	NEU
nsp3_NAB	1	61	V	**1.309**	MIN	V	1.347	MIN
nsp3_NAB	1	62	V	**1.309**	MIN	V	1.232	MIN
nsp3_NAB	1	63	A	**1.309**	NEU	A	0.372	NEU
nsp3_NAB	1	64	I	**1.309**	MIN	I	1.4	MIN
nsp3_NAB	1	65	D	**1.309**	NEU	D	-0.118	NEU
nsp3_NAB	1	66	Y	**1.309**	MIN	Y	0.755	NEU
nsp3_NAB	1	67	R	**0.718**	NEU	K	-0.335	NEU
nsp3_NAB	1	68	H	**1.087**	NEU	H	-0.117	NEU
nsp3_NAB	1	69	Y	0.421	MAX	Y	-1.072	MAX
nsp3_NAB	1	70	S	**0.632**	NEU	T	0.264	NEU
nsp3_NAB	1	71	A	0.343	MIN	P	-0.738	NEU
nsp3_NAB	1	72	S	**1.087**	NEU	S	-0.308	NEU
nsp3_NAB	1	73	F	**1.309**	MIN	F	1.098	MIN
nsp3_NAB	1	74	K	**0.938**	NEU	K	-0.686	NEU
nsp3_NAB	1	75	K	**0.632**	NEU	K	-1.03	MAX
nsp3_NAB	1	76	G	**1.309**	NEU	G	0.066	NEU
nsp3_NAB	1	77	A	**0.560**	NEU	A	0.532	NEU
nsp3_NAB	1	78	K	**1.309**	MAX	K	-1.39	MAX
nsp3_NAB	1	79	L	**1.309**	MIN	L	1.03	MIN
nsp3_NAB	1	80	L	**0.938**	MIN	L	1.166	MIN
nsp3_NAB	1	81	H	**1.087**	MIN	H	0.614	NEU
nsp3_NAB	1	82	K	0.313	NEU	K	-0.95	NEU
nsp3_NAB	1	83	P	**1.087**	NEU	P	-0.843	NEU
nsp3_NAB	1	84	I	**1.309**	MIN	I	1.205	MIN
nsp3_NAB	1	85	V	**1.309**	MIN	V	1.192	MIN
nsp3_NAB	1	86	W	**1.309**	MIN	W	0.86	MIN
nsp3_NAB	1	87	H	**1.309**	NEU	H	0.121	NEU

nsp3_NAB	1	88	I	**1.087**	MIN	V	1.354	MIN
nsp3_NAB	1	89	N	**0.938**	NEU	N	-0.084	NEU
nsp3_NAB	1	90	Q	0.343	MAX	N	-0.622	NEU
nsp3_NAB	1	91	T	0.242	NEU	A	0.422	NEU
nsp3_NAB	1	92	T	**0.938**	NEU	T	-0.004	NEU
nsp3_NAB	1	93	N	0.498	NEU	N	-0.744	NEU
nsp3_NAB	1	94	K	0.324	MIN	K	0.792	MIN
nsp3_NAB	1	95	T	**1.309**	NEU	A	0.002	NEU
nsp3_NAB	1	96	T	**1.087**	NEU	T	-0.053	NEU
nsp3_NAB	1	97	Y	-0.023	MAX	Y	-2.579	MAX
nsp3_NAB	1	98	K	**1.087**	NEU	K	-0.364	NEU
nsp3_NAB	1	99	P	**1.087**	NEU	P	-0.276	NEU
nsp3_NAB	1	100	N	0.404	NEU	N	-0.954	NEU
nsp3_NAB	1	101	T	**0.632**	NEU	T	-0.254	NEU
nsp3_NAB	1	102	W	0.498	NEU	W	-1.204	MAX
nsp3_NAB	1	103	C	**1.309**	MIN	C	1.909	MIN
nsp3_NAB	1	104	L	**1.309**	MIN	I	1.208	MIN
nsp3_NAB	1	105	R	**1.309**	NEU	R	-0.568	NEU
nsp3_NAB	1	106	C	**1.309**	MIN	C	1.86	MIN
nsp3_NAB	1	107	L	**1.309**	MIN	L	1.076	MIN
nsp3_NAB	1	108	W	**0.818**	MIN	W	0.921	MIN
nsp3_NAB	1	109	S	**1.309**	NEU	S	0.483	NEU
nsp3_NAB	1	110	T	**1.087**	NEU	T	-0.496	NEU
nsp3_NAB	1	111	K	-0.247	MAX	K	-1.14	MAX
nsp3_NAB	1	112	P	**1.087**	MIN	P	1.788	MIN
nsp3_NAB	1	113	V	0.242	MAX	V	-1.105	MAX
nsp3_Y3	1	1	H	**0.544**	MIN	Q	-0.033	NEU
nsp3_Y3	1	2	S	0.191	MAX	S	-1.35	MAX
nsp3_Y3	1	3	D	0.349	NEU	D	-1.26	MAX
nsp3_Y3	1	4	L	**0.969**	MIN	I	1.048	MIN
nsp3_Y3	1	5	E	**0.760**	MAX	E	-1.155	MAX
nsp3_Y3	1	6	V	**1.314**	MIN	V	1.109	MIN
nsp3_Y3	1	7	T	**1.314**	NEU	T	-0.253	NEU
nsp3_Y3	1	8	G	**1.314**	NEU	G	-0.863	NEU
nsp3_Y3	1	9	D	**1.109**	MAX	D	-1.83	MAX
nsp3_Y3	1	10	S	**1.314**	NEU	S	-0.749	NEU
nsp3_Y3	1	11	C	**1.109**	MIN	C	1.06	MIN
nsp3_Y3	1	12	N	**1.109**	NEU	N	-0.876	NEU
nsp3_Y3	1	13	N	**1.109**	NEU	N	-0.661	NEU
nsp3_Y3	1	14	F	0.491	MIN	Y	0.306	NEU
nsp3_Y3	1	15	M	**1.314**	MIN	M	1.099	MIN
nsp3_Y3	1	16	L	**1.314**	MIN	L	1.485	MIN
nsp3_Y3	1	17	T	**1.314**	NEU	T	0.019	NEU
nsp3_Y3	1	18	Y	**1.314**	MAX	Y	-3.679	MAX
nsp3_Y3	1	19	N	**1.314**	MAX	N	-1.082	MAX
nsp3_Y3	1	20	K	0.315	NEU	K	0.025	NEU

nsp3_Y3	1	21	V	**1.314**	MIN	V	0.892	MIN
nsp3_Y3	1	22	E	**1.314**	MIN	E	2.329	MIN
nsp3_Y3	1	23	N	0.376	NEU	N	0.621	NEU
nsp3_Y3	1	24	M	**1.314**	NEU	M	-0.103	NEU
nsp3_Y3	1	25	T	**1.109**	MIN	T	0.877	MIN
nsp3_Y3	1	26	P	**1.314**	NEU	P	-0.623	NEU
nsp3_Y3	1	27	R	**0.544**	NEU	R	-0.998	NEU
nsp3_Y3	1	28	D	**1.314**	MAX	D	-1.2	MAX
nsp3_Y3	1	29	L	**1.314**	MIN	L	1.37	MIN
nsp3_Y3	1	30	G	**1.314**	NEU	G	-0.483	NEU
nsp3_Y3	1	31	A	**1.109**	NEU	A	0.13	NEU
nsp3_Y3	1	32	C	**1.109**	MIN	C	1.623	MIN
nsp3_Y3	1	33	I	**1.314**	MIN	I	1.834	MIN
nsp3_Y3	1	34	D	0.407	NEU	D	-1.037	MAX
nsp3_Y3	1	35	C	**1.314**	MIN	C	1.535	MIN
nsp3_Y3	1	36	N	**1.314**	NEU	S	-0.549	NEU
nsp3_Y3	1	37	A	**1.314**	NEU	A	0.145	NEU
nsp3_Y3	1	38	R	**1.314**	NEU	R	-0.58	NEU
nsp3_Y3	1	39	H	**1.314**	NEU	H	-0.037	NEU
nsp3_Y3	1	40	I	**1.314**	MIN	I	1.91	MIN
nsp3_Y3	1	41	N	**1.314**	NEU	N	-0.745	NEU
nsp3_Y3	1	42	A	0.491	NEU	A	0.443	NEU
nsp3_Y3	1	43	Q	**0.905**	NEU	Q	0.114	NEU
nsp3_Y3	1	44	V	**1.314**	MIN	V	1.295	MIN
nsp3_Y3	1	45	A	**1.314**	NEU	A	0.175	NEU
nsp3_Y3	1	46	K	**0.969**	NEU	K	-0.035	NEU
nsp3_Y3	1	47	S	**1.109**	NEU	S	-0.708	NEU
nsp3_Y3	1	48	H	**1.314**	NEU	H	-0.342	NEU
nsp3_Y3	1	49	N	0.169	NEU	N	0.755	NEU
nsp3_Y3	1	50	V	**1.109**	MIN	I	1.913	MIN
nsp3_Y3	1	51	S	**1.314**	NEU	A	-0.402	NEU
nsp3_Y3	1	52	L	**1.314**	MIN	L	1.457	MIN
nsp3_Y3	1	53	I	**1.314**	MIN	I	0.894	MIN
nsp3_Y3	1	54	W	0.407	MIN	W	0.524	NEU
nsp3_Y3	1	55	N	**1.314**	NEU	N	-0.231	NEU
nsp3_Y3	1	56	V	**1.314**	MIN	V	1.415	MIN
nsp3_Y3	1	57	K	**1.109**	MAX	K	-1.292	MAX
nsp3_Y3	1	58	D	**1.314**	NEU	D	-0.935	NEU
nsp3_Y3	1	59	Y	0.491	NEU	F	0.904	MIN
nsp3_Y3	1	60	M	**0.856**	NEU	M	0.38	NEU
nsp3_Y3	1	61	S	**1.109**	NEU	S	-0.089	NEU
nsp3_Y3	1	62	L	**0.969**	MIN	L	0.862	MIN
nsp3_Y3	1	63	S	**1.314**	NEU	S	-0.06	NEU
nsp3_Y3	1	64	E	**1.109**	MIN	E	1.138	MIN
nsp3_Y3	1	65	Q	**1.314**	NEU	Q	0.18	NEU
nsp3_Y3	1	66	L	**1.314**	MIN	L	1.109	MIN

nsp3_Y3	1	67	R	**1.314**	NEU	R	-0.142	NEU
nsp3_Y3	1	68	K	**0.969**	MAX	K	-1.672	MAX
nsp3_Y3	1	69	Q	**1.314**	MAX	Q	-1.044	MAX
nsp3_Y3	1	70	I	**1.314**	MIN	I	1.622	MIN
nsp3_Y3	1	71	R	**0.969**	MAX	R	-1.149	MAX
nsp3_Y3	1	72	S	**1.314**	NEU	S	-0.285	NEU
nsp3_Y3	1	73	A	**1.314**	NEU	A	0.331	NEU
nsp3_Y3	1	74	A	0.407	NEU	A	0.48	NEU
nsp3_Y3	1	75	K	**0.905**	MAX	K	-1.947	MAX
nsp3_Y3	1	76	K	**1.109**	MAX	K	-1.145	MAX
nsp3_Y3	1	77	N	**0.969**	MAX	N	-1.024	MAX
nsp3_Y3	1	78	N	**1.109**	MIN	N	0.597	NEU
nsp3_Y3	1	79	I	**1.314**	MIN	L	1.186	MIN
nsp3_Y3	1	80	P	**1.314**	NEU	P	-0.662	NEU
nsp3_Y3	1	81	F	**1.314**	MIN	F	0.88	MIN
nsp3_Y3	1	82	R	0.407	NEU	K	-1.285	MAX
nsp3_Y3	1	83	L	**1.314**	MIN	L	1.291	MIN
nsp3_Y3	1	84	T	**1.314**	NEU	T	-0.369	NEU
nsp3_Y3	1	85	C	**1.109**	NEU	C	-0.476	NEU
nsp3_Y3	1	86	A	0.491	NEU	A	0.538	NEU
nsp3_Y3	1	87	T	**1.109**	NEU	T	-0.216	NEU
nsp3_Y3	1	88	T	**1.109**	NEU	T	-0.057	NEU
nsp3_Y3	1	89	R	**1.109**	NEU	R	-0.092	NEU
nsp3_Y3	1	90	Q	**0.544**	NEU	Q	-1.122	MAX
nsp3_Y3	1	91	V	0.445	MAX	V	-0.963	NEU
nsp3_Y3	1	92	V	**1.109**	MIN	V	1.409	MIN
nsp3_Y3	1	93	N	0.299	NEU	N	-1.12	MAX
nsp3_Y3	1	94	V	**1.314**	MIN	V	1.396	MIN
nsp3_Y3	1	95	I	**1.314**	MIN	V	0.888	MIN
nsp3_Y3	1	96	T	**1.314**	NEU	T	-0.091	NEU
nsp3_Y3	1	97	T	**1.314**	NEU	T	-0.055	NEU
nsp3_Y3	1	98	K	**1.314**	MAX	K	-1.414	MAX
nsp3_Y3	1	99	I	**1.314**	MIN	I	1.34	MIN
nsp3_Y3	1	100	S	**1.314**	NEU	A	-0.777	NEU
nsp3_Y3	1	101	L	**1.314**	MIN	L	1.024	MIN
nsp3_Y3	1	102	K	**0.856**	MIN	K	0.857	MIN
nsp5	1	1	S	**0.772**	NEU	S	-0.174	NEU
nsp5	1	2	G	0.341	MAX	G	-0.989	NEU
nsp5	1	3	L	**1.316**	MIN	F	1.17	MIN
nsp5	1	4	V	0.319	NEU	R	0.752	NEU
nsp5	1	5	K	-0.006	NEU	K	-1.734	MAX
nsp5	1	6	M	0.341	NEU	M	0.561	NEU
nsp5	1	7	A	**0.781**	NEU	A	-1.104	MAX
nsp5	1	8	H	0.498	NEU	F	0.78	MIN
nsp5	1	9	P	0.341	NEU	P	-1.017	MAX
nsp5	1	10	S	**1.316**	NEU	S	-0.618	NEU

nsp5	1	11	G	**1.316**	NEU	G	-0.783	NEU
nsp5	1	12	A	**0.578**	NEU	K	-0.541	NEU
nsp5	1	13	V	**1.316**	MIN	V	1.1	MIN
nsp5	1	14	E	**1.316**	MAX	E	-1.127	MAX
nsp5	1	15	P	**0.671**	NEU	G	-0.582	NEU
nsp5	1	16	C	**1.316**	MIN	C	1.493	MIN
nsp5	1	17	M	**1.316**	MIN	M	0.792	MIN
nsp5	1	18	V	**1.316**	MIN	V	1.185	MIN
nsp5	1	19	Q	**0.867**	NEU	Q	-0.557	NEU
nsp5	1	20	V	**1.316**	MIN	V	1.104	MIN
nsp5	1	21	T	**0.781**	NEU	T	0.148	NEU
nsp5	1	22	C	**1.115**	MIN	C	0.944	MIN
nsp5	1	23	G	**1.316**	NEU	G	-0.865	NEU
nsp5	1	24	S	0.316	MIN	T	0.96	MIN
nsp5	1	25	M	**0.867**	NEU	T	-0.385	NEU
nsp5	1	26	T	**1.316**	NEU	T	-0.001	NEU
nsp5	1	27	L	**1.316**	MIN	L	0.721	NEU
nsp5	1	28	N	**0.691**	NEU	N	-0.811	NEU
nsp5	1	29	G	**1.316**	NEU	G	-0.387	NEU
nsp5	1	30	L	**1.316**	MIN	L	1.2	MIN
nsp5	1	31	W	**0.979**	NEU	W	0.334	NEU
nsp5	1	32	L	**1.316**	MIN	L	1.196	MIN
nsp5	1	33	D	**0.772**	MAX	D	-1.276	MAX
nsp5	1	34	N	**0.979**	MAX	D	-1.071	MAX
nsp5	1	35	Y	0.341	MIN	V	1.279	MIN
nsp5	1	36	V	**1.316**	MIN	V	1.163	MIN
nsp5	1	37	Y	**0.867**	MIN	Y	0.671	NEU
nsp5	1	38	C	**1.316**	MIN	C	1.392	MIN
nsp5	1	39	P	**1.115**	MAX	P	-1.083	MAX
nsp5	1	40	R	**1.316**	NEU	R	-0.453	NEU
nsp5	1	41	H	**1.316**	NEU	H	-0.069	NEU
nsp5	1	42	V	**1.316**	MIN	V	1.201	MIN
nsp5	1	43	M	0.200	MIN	I	0.956	MIN
nsp5	1	44	C	**1.314**	MIN	C	2.102	MIN
nsp5	1	45	P	0.352	NEU	T	0.914	MIN
nsp5	1	46	A	0.407	NEU	S	-0.638	NEU
nsp5	1	47	D	0.302	NEU	E	-0.186	NEU
nsp5	1	48	Q	-0.006	NEU	D	-0.864	NEU
nsp5	1	49	L	0.369	MIN	M	-0.834	NEU
nsp5	1	50	L	-0.050	NEU	L	-0.343	NEU
nsp5	1	51	D	0.194	MIN	N	0.727	NEU
nsp5	1	52	P	0.319	MAX	P	-1.128	MAX
nsp5	1	53	N	**0.505**	NEU	N	-0.73	NEU
nsp5	1	54	Y	0.319	NEU	Y	0.212	NEU
nsp5	1	55	D	0.361	MAX	E	-1.505	MAX
nsp5	1	56	A	**0.558**	NEU	D	-0.291	NEU

nsp5	1	57	L	0.388	MIN	L	0.822	MIN
nsp5	1	58	L	**0.558**	MIN	L	1.45	MIN
nsp5	1	59	I	0.236	NEU	I	-0.354	NEU
nsp5	1	60	S	**1.115**	NEU	R	0.357	NEU
nsp5	1	61	M	0.118	MIN	K	-1.424	MAX
nsp5	1	62	T	**0.772**	NEU	S	-0.06	NEU
nsp5	1	63	N	0.361	MAX	N	-1.037	MAX
nsp5	1	64	H	**0.505**	NEU	H	0.207	NEU
nsp5	1	65	S	**0.781**	NEU	N	-0.833	NEU
nsp5	1	66	F	**1.316**	MIN	F	1.383	MIN
nsp5	1	67	H	0.327	MIN	L	0.017	NEU
nsp5	1	68	V	**1.316**	MIN	V	1.197	MIN
nsp5	1	69	V	0.498	NEU	Q	-0.802	NEU
nsp5	1	70	G	0.180	NEU	A	0.316	NEU
nsp5	1	71	A	**1.109**	NEU	G	-0.681	NEU
nsp5	1	72	P	0.361	NEU	N	0.639	NEU
nsp5	1	73	A	0.317	NEU	V	1.433	MIN
nsp5	1	74	N	0.025	NEU	Q	-0.426	NEU
nsp5	1	75	L	**1.316**	MIN	L	1.057	MIN
nsp5	1	76	R	**1.115**	NEU	R	0.093	NEU
nsp5	1	77	V	**1.316**	MIN	V	1.216	MIN
nsp5	1	78	I	0.319	MIN	I	-0.194	NEU
nsp5	1	79	G	**0.979**	NEU	G	-0.785	NEU
nsp5	1	80	H	**1.316**	NEU	H	-0.221	NEU
nsp5	1	81	S	0.302	NEU	S	0.169	NEU
nsp5	1	82	M	**1.115**	MIN	M	1.111	MIN
nsp5	1	83	Q	-0.193	NEU	Q	-1.208	MAX
nsp5	1	84	G	**0.867**	NEU	N	-0.813	NEU
nsp5	1	85	T	**0.558**	NEU	C	1.591	MIN
nsp5	1	86	L	**1.316**	MIN	V	0.86	MIN
nsp5	1	87	L	**1.316**	MIN	L	1.327	MIN
nsp5	1	88	K	0.361	MAX	K	-1.374	MAX
nsp5	1	89	L	**1.316**	MIN	L	1.066	MIN
nsp5	1	90	T	**1.115**	NEU	K	-0.607	NEU
nsp5	1	91	V	**1.316**	MIN	V	1.328	MIN
nsp5	1	92	D	**0.671**	NEU	D	0.255	NEU
nsp5	1	93	S	0.147	NEU	T	-0.431	NEU
nsp5	1	94	A	-0.090	NEU	A	-0.987	NEU
nsp5	1	95	N	0.361	MAX	N	-1.016	MAX
nsp5	1	96	P	0.236	NEU	P	-0.215	NEU
nsp5	1	97	K	0.236	NEU	K	-0.666	NEU
nsp5	1	98	T	**1.316**	NEU	T	-0.455	NEU
nsp5	1	99	P	**1.316**	NEU	P	-0.462	NEU
nsp5	1	100	A	0.302	NEU	K	0.061	NEU
nsp5	1	101	Y	**0.867**	MIN	Y	0.604	NEU
nsp5	1	102	T	**0.558**	NEU	K	-1.425	MAX

nsp5	1	103	F	**0.772**	MIN	F	0.672	NEU
nsp5	1	104	T	0.327	NEU	V	1.289	MIN
nsp5	1	105	T	0.302	NEU	R	1.286	MIN
nsp5	1	106	V	**0.979**	MIN	I	1.293	MIN
nsp5	1	107	K	0.369	MAX	Q	-1.019	MAX
nsp5	1	108	P	0.097	NEU	P	-0.93	NEU
nsp5	1	109	G	**1.316**	NEU	G	-0.342	NEU
nsp5	1	110	Q	0.097	NEU	Q	-0.831	NEU
nsp5	1	111	S	**0.916**	NEU	T	-0.056	NEU
nsp5	1	112	F	**1.316**	MIN	F	1.368	MIN
nsp5	1	113	S	**1.316**	NEU	S	-0.58	NEU
nsp5	1	114	V	**1.316**	MIN	V	1.248	MIN
nsp5	1	115	L	**1.316**	MIN	L	0.884	MIN
nsp5	1	116	A	**1.115**	NEU	A	0.375	NEU
nsp5	1	117	C	**0.979**	MIN	C	2.024	MIN
nsp5	1	118	Y	**0.505**	MAX	Y	-2.864	MAX
nsp5	1	119	N	**0.558**	NEU	N	0.295	NEU
nsp5	1	120	G	**1.316**	NEU	G	-0.131	NEU
nsp5	1	121	R	0.317	NEU	S	0.119	NEU
nsp5	1	122	P	0.153	MAX	P	-0.835	NEU
nsp5	1	123	T	**0.772**	NEU	S	-0.566	NEU
nsp5	1	124	G	**1.316**	NEU	G	-0.457	NEU
nsp5	1	125	V	0.090	MIN	V	1.43	MIN
nsp5	1	126	Y	**0.620**	NEU	Y	-0.349	NEU
nsp5	1	127	T	**1.115**	NEU	Q	-0.792	NEU
nsp5	1	128	V	**1.316**	MIN	C	1.888	MIN
nsp5	1	129	V	0.044	NEU	A	0.491	NEU
nsp5	1	130	M	**1.115**	MIN	M	1.028	MIN
nsp5	1	131	R	0.388	MIN	R	-0.522	NEU
nsp5	1	132	P	0.361	MAX	P	-1.333	MAX
nsp5	1	133	N	0.273	NEU	N	-0.755	NEU
nsp5	1	134	G	0.097	NEU	F	1.383	MIN
nsp5	1	135	T	**0.979**	NEU	T	-0.362	NEU
nsp5	1	136	I	**1.316**	MIN	I	1.167	MIN
nsp5	1	137	K	0.057	NEU	K	-1.103	MAX
nsp5	1	138	G	**1.316**	NEU	G	-0.521	NEU
nsp5	1	139	S	**1.316**	NEU	S	0.234	NEU
nsp5	1	140	F	**1.316**	MIN	F	1.274	MIN
nsp5	1	141	L	**0.578**	MIN	L	0.734	NEU
nsp5	1	142	C	**0.558**	MIN	N	-0.679	NEU
nsp5	1	143	G	**1.316**	NEU	G	-0.5	NEU
nsp5	1	144	S	**1.316**	NEU	S	-0.542	NEU
nsp5	1	145	C	**1.316**	MIN	C	2.409	MIN
nsp5	1	146	G	**1.316**	NEU	G	-0.291	NEU
nsp5	1	147	S	**1.316**	NEU	S	-0.615	NEU
nsp5	1	148	V	**0.505**	MIN	V	1.182	MIN

nsp5	1	149	G	**1.316**	NEU	G	-0.478	NEU
nsp5	1	150	Y	0.361	MIN	F	1.199	MIN
nsp5	1	151	T	-0.032	NEU	N	-0.839	NEU
nsp5	1	152	K	-0.251	NEU	I	1.711	MIN
nsp5	1	153	E	0.044	MAX	D	-0.13	NEU
nsp5	1	154	G	**0.558**	NEU	Y	-1.047	MAX
nsp5	1	155	N	**0.505**	NEU	D	1.644	MIN
nsp5	1	156	V	-0.006	MIN	C	1.823	MIN
nsp5	1	157	I	**1.316**	MIN	V	1.064	MIN
nsp5	1	158	N	0.459	MAX	S	-0.429	NEU
nsp5	1	159	F	**1.316**	MIN	F	1.228	MIN
nsp5	1	160	C	**1.316**	MIN	C	1.78	MIN
nsp5	1	161	Y	**1.316**	NEU	Y	0.517	NEU
nsp5	1	162	M	**0.647**	MIN	M	1.198	MIN
nsp5	1	163	H	**1.316**	NEU	H	-0.368	NEU
nsp5	1	164	Q	0.316	NEU	H	-0.092	NEU
nsp5	1	165	M	0.273	NEU	M	0.281	NEU
nsp5	1	166	E	**1.115**	MAX	E	-1.844	MAX
nsp5	1	167	L	0.316	MIN	L	1.246	MIN
nsp5	1	168	P	-0.259	NEU	P	0.775	NEU
nsp5	1	169	N	0.388	NEU	T	-0.264	NEU
nsp5	1	170	G	0.317	MAX	G	-1.091	MAX
nsp5	1	171	T	**0.505**	NEU	V	1.816	MIN
nsp5	1	172	H	**1.316**	NEU	H	-0.447	NEU
nsp5	1	173	T	**1.115**	NEU	A	0.407	NEU
nsp5	1	174	G	**1.316**	NEU	G	-0.331	NEU
nsp5	1	175	S	**0.979**	NEU	T	-0.479	NEU
nsp5	1	176	D	0.327	NEU	D	-1.232	MAX
nsp5	1	177	F	**1.316**	MIN	L	1.16	MIN
nsp5	1	178	D	-0.020	NEU	E	-1.747	MAX
nsp5	1	179	G	**1.316**	NEU	G	-0.273	NEU
nsp5	1	180	N	0.361	NEU	N	-0.133	NEU
nsp5	1	181	F	0.236	NEU	F	1.271	MIN
nsp5	1	182	Y	**0.979**	MAX	Y	-2.777	MAX
nsp5	1	183	G	**1.316**	NEU	G	-0.726	NEU
nsp5	1	184	P	-0.020	NEU	P	0.669	NEU
nsp5	1	185	F	0.341	NEU	F	1.48	MIN
nsp5	1	186	E	0.041	MIN	V	0.815	MIN
nsp5	1	187	D	0.341	NEU	D	-0.858	NEU
nsp5	1	188	R	**0.867**	NEU	R	0.205	NEU
nsp5	1	189	Q	0.459	MIN	Q	0.668	NEU
nsp5	1	190	V	0.341	MIN	T	0.776	NEU
nsp5	1	191	H	0.044	MAX	A	-1.588	MAX
nsp5	1	192	Q	**0.979**	NEU	Q	-0.82	NEU
nsp5	1	193	A	-0.063	NEU	A	-1.444	MAX
nsp5	1	194	A	**0.505**	MIN	A	0.848	MIN

nsp5	1	195	L	-0.104	NEU	G	0.208	NEU
nsp5	1	196	T	0.388	MIN	T	0.073	NEU
nsp5	1	197	D	0.206	MIN	D	0.558	NEU
nsp5	1	198	K	-0.104	MAX	T	-0.043	NEU
nsp5	1	199	T	0.316	MIN	T	-0.121	NEU
nsp5	1	200	C	**1.115**	MIN	I	1.153	MIN
nsp5	1	201	T	**1.316**	NEU	T	-0.198	NEU
nsp5	1	202	V	**0.620**	MIN	V	1.464	MIN
nsp5	1	203	N	0.459	MAX	N	-1.043	MAX
nsp5	1	204	V	**1.316**	MIN	V	1.26	MIN
nsp5	1	205	V	**1.316**	MIN	L	1.25	MIN
nsp5	1	206	A	**0.772**	NEU	A	0.307	NEU
nsp5	1	207	W	**1.316**	NEU	W	0.215	NEU
nsp5	1	208	L	**1.316**	MIN	L	1.43	MIN
nsp5	1	209	Y	**1.316**	NEU	Y	-0.272	NEU
nsp5	1	210	A	0.459	NEU	A	0.155	NEU
nsp5	1	211	A	**1.115**	NEU	A	0.418	NEU
nsp5	1	212	V	**1.316**	MIN	V	1.418	MIN
nsp5	1	213	L	**1.314**	MIN	I	1.526	MIN
nsp5	1	214	N	**0.760**	NEU	N	-0.9	NEU
nsp5	1	215	G	**1.314**	NEU	G	-0.595	NEU
nsp5	1	216	C	-0.090	MIN	D	-0.883	NEU
nsp5	1	217	N	-0.001	MAX	R	-0.944	NEU
nsp5	1	218	W	0.147	MIN	W	-0.007	NEU
nsp5	1	219	F	0.316	MIN	F	1.028	MIN
nsp5	1	220	V	**1.115**	MIN	L	0.535	NEU
nsp5	1	221	K	0.118	NEU	N	-1.074	MAX
nsp5	1	222	P	0.153	NEU	R	1.376	MIN
nsp5	1	223	N	**0.781**	NEU	F	-0.691	NEU
nsp5	1	224	R	**0.916**	NEU	T	-0.167	NEU
nsp5	1	225	T	0.420	NEU	T	-0.497	NEU
nsp5	1	226	S	**0.772**	NEU	T	-0.36	NEU
nsp5	1	227	V	0.255	MIN	L	0.889	MIN
nsp5	1	228	A	**0.781**	NEU	N	0.087	NEU
nsp5	1	229	D	**0.781**	NEU	D	-0.796	NEU
nsp5	1	230	F	**0.867**	MIN	F	0.717	NEU
nsp5	1	231	N	0.361	MAX	N	-0.998	NEU
nsp5	1	232	E	0.025	MAX	L	0.032	NEU
nsp5	1	233	W	0.153	NEU	V	1.214	MIN
nsp5	1	234	A	**1.115**	NEU	A	0.163	NEU
nsp5	1	235	M	-0.152	MIN	M	0.106	NEU
nsp5	1	236	K	**0.505**	NEU	K	-1.19	MAX
nsp5	1	237	N	**0.916**	NEU	Y	-0.699	NEU
nsp5	1	238	Q	**0.620**	NEU	N	-0.166	NEU
nsp5	1	239	F	0.342	MIN	Y	-0.952	NEU
nsp5	1	240	T	**0.620**	NEU	E	-1.061	MAX

nsp5	1	241	E	0.361	MAX	P	-1.113	MAX
nsp5	1	242	F	**1.316**	MIN	L	1.199	MIN
nsp5	1	243	T	**0.558**	MIN	T	-0.199	NEU
nsp5	1	244	G	**0.558**	NEU	Q	-1.059	MAX
nsp5	1	245	T	0.429	NEU	D	-1.142	MAX
nsp5	1	246	-	**1.265**	NEU	H	-0.194	NEU
nsp5	1	247	-	**1.265**	MIN	V	1.367	MIN
nsp5	1	248	Q	0.498	NEU	D	0.085	NEU
nsp5	1	249	A	0.319	NEU	I	1.625	MIN
nsp5	1	250	L	**1.316**	MIN	L	1.032	MIN
nsp5	1	251	D	0.388	MAX	G	-0.603	NEU
nsp5	1	252	P	0.062	NEU	P	-0.779	NEU
nsp5	1	253	L	**1.316**	MIN	L	1.3	MIN
nsp5	1	254	A	0.420	MIN	S	-0.718	NEU
nsp5	1	255	A	0.153	MIN	A	-0.482	NEU
nsp5	1	256	K	0.459	MAX	Q	-0.978	NEU
nsp5	1	257	T	**1.316**	NEU	T	-0.387	NEU
nsp5	1	258	G	**0.620**	NEU	G	-0.778	NEU
nsp5	1	259	V	**1.316**	MIN	I	1.628	MIN
nsp5	1	260	S	**0.578**	NEU	A	0.47	NEU
nsp5	1	261	V	**1.115**	MIN	V	1.281	MIN
nsp5	1	262	E	0.498	MAX	L	1.384	MIN
nsp5	1	263	Q	0.327	NEU	D	-0.698	NEU
nsp5	1	264	M	**1.115**	MIN	M	0.675	NEU
nsp5	1	265	L	**1.316**	MIN	C	0.91	MIN
nsp5	1	266	Y	0.244	NEU	A	-0.001	NEU
nsp5	1	267	A	0.319	NEU	S	-0.793	NEU
nsp5	1	268	L	**1.115**	MIN	L	1.291	MIN
nsp5	1	269	K	**1.316**	MAX	K	-1.537	MAX
nsp5	1	270	Q	-0.211	NEU	E	-2.078	MAX
nsp5	1	271	-	**1.265**	MIN	L	1.43	MIN
nsp5	1	272	L	**1.316**	MIN	L	1.558	MIN
nsp5	1	273	S	0.369	NEU	Q	-1.611	MAX
nsp5	1	274	N	0.153	NEU	N	-0.011	NEU
nsp5	1	275	G	0.327	NEU	G	-1.507	MAX
nsp5	1	276	F	**0.979**	MIN	M	0.792	MIN
nsp5	1	277	Q	-0.090	NEU	N	1.107	MIN
nsp5	1	278	G	0.327	MAX	G	-0.515	NEU
nsp5	1	279	K	0.341	NEU	R	-0.026	NEU
nsp5	1	280	T	0.429	NEU	T	0.021	NEU
nsp5	1	281	I	**1.316**	MIN	I	1.457	MIN
nsp5	1	282	L	**1.316**	MIN	L	0.951	MIN
nsp5	1	283	G	**0.578**	MAX	G	-0.647	NEU
nsp5	1	284	S	**0.671**	NEU	S	-0.59	NEU
nsp5	1	285	T	0.498	NEU	A	-0.295	NEU
nsp5	1	286	M	0.182	NEU	L	1.108	MIN

nsp5	1	287	L	**1.316**	MIN	L	1.555	MIN
nsp5	1	288	E	**1.316**	MAX	E	-1.235	MAX
nsp5	1	289	D	0.420	MAX	D	-1.236	MAX
nsp5	1	290	E	0.429	NEU	E	-1.351	MAX
nsp5	1	291	F	0.327	MIN	F	1.18	MIN
nsp5	1	292	T	**1.316**	NEU	T	-0.52	NEU
nsp5	1	293	P	**0.772**	MAX	P	-1.084	MAX
nsp5	1	294	E	-0.178	MAX	F	1.241	MIN
nsp5	1	295	D	**0.691**	NEU	D	-0.348	NEU
nsp5	1	296	V	**1.316**	MIN	V	1.077	MIN
nsp5	1	297	N	-0.178	MAX	V	1.288	MIN
nsp5	1	298	M	0.498	NEU	R	0.144	NEU
nsp5	1	299	Q	**0.772**	MAX	Q	-0.987	NEU
nsp5	1	300	M	**0.691**	MIN	C	1.163	MIN
nsp5	1	301	M	**0.867**	NEU	S	-0.487	NEU
nsp5	1	302	G	**1.316**	NEU	G	-0.425	NEU
nsp5	1	303	V	**0.772**	MIN	V	0.302	NEU
nsp5	1	304	V	0.147	MIN	T	-0.063	NEU
nsp5	1	305	M	-0.099	NEU	F	-0.075	NEU
nsp7	1	1	K	0.442	NEU	K	-0.319	NEU
nsp7	1	2	L	0.403	MIN	M	-0.851	NEU
nsp7	1	3	T	**0.610**	NEU	S	-0.283	NEU
nsp7	1	4	D	0.403	NEU	D	0.304	NEU
nsp7	1	5	L	**1.321**	MIN	V	1.137	MIN
nsp7	1	6	K	**1.138**	MAX	K	-1.143	MAX
nsp7	1	7	C	**1.138**	MIN	C	1.802	MIN
nsp7	1	8	T	**1.321**	NEU	T	-0.254	NEU
nsp7	1	9	S	**1.321**	NEU	S	-0.822	NEU
nsp7	1	10	V	**1.321**	MIN	V	1.272	MIN
nsp7	1	11	V	**1.321**	MIN	V	1.174	MIN
nsp7	1	12	L	**1.321**	MIN	L	0.92	MIN
nsp7	1	13	L	**1.321**	MIN	L	0.951	MIN
nsp7	1	14	S	**1.321**	NEU	S	-0.748	NEU
nsp7	1	15	V	**1.321**	MIN	V	1.325	MIN
nsp7	1	16	L	**1.138**	MIN	L	1.087	MIN
nsp7	1	17	Q	**1.011**	MAX	Q	-1.308	MAX
nsp7	1	18	Q	0.341	NEU	Q	-1.028	MAX
nsp7	1	19	L	**1.321**	MIN	L	1.098	MIN
nsp7	1	20	H	0.433	MIN	R	1.192	MIN
nsp7	1	21	L	**1.321**	MIN	V	1.255	MIN
nsp7	1	22	E	-0.094	MAX	E	-0.915	NEU
nsp7	1	23	A	**0.907**	NEU	S	-0.265	NEU
nsp7	1	24	N	0.323	NEU	S	-0.991	NEU
nsp7	1	25	S	**1.321**	NEU	S	-0.818	NEU
nsp7	1	26	K	**0.557**	MAX	K	-1.622	MAX
nsp7	1	27	A	0.377	NEU	L	1.251	MIN

nsp7	1	28	W	**1.138**	NEU	W	-0.553	NEU
nsp7	1	29	A	**0.740**	NEU	A	0.617	NEU
nsp7	1	30	H	**0.610**	NEU	Q	-0.882	NEU
nsp7	1	31	C	**1.321**	MIN	C	1.119	MIN
nsp7	1	32	V	**1.321**	MIN	V	0.856	MIN
nsp7	1	33	K	-0.049	NEU	Q	0.419	NEU
nsp7	1	34	L	**1.011**	MIN	L	1.091	MIN
nsp7	1	35	H	**1.321**	NEU	H	-0.106	NEU
nsp7	1	36	N	**1.321**	MAX	N	-1.172	MAX
nsp7	1	37	D	0.330	NEU	D	-1.427	MAX
nsp7	1	38	I	**1.321**	MIN	I	1.36	MIN
nsp7	1	39	L	**0.907**	NEU	L	-0.565	NEU
nsp7	1	40	A	-0.210	NEU	L	-1.1	MAX
nsp7	1	41	A	**0.907**	NEU	A	-0.274	NEU
nsp7	1	42	T	0.433	NEU	K	1.012	MIN
nsp7	1	43	D	**0.610**	MAX	D	-1.507	MAX
nsp7	1	44	P	0.377	MAX	T	-0.364	NEU
nsp7	1	45	T	**1.321**	NEU	T	-0.34	NEU
nsp7	1	46	E	0.180	MAX	E	-0.354	NEU
nsp7	1	47	A	**0.510**	NEU	A	0.14	NEU
nsp7	1	48	F	0.468	NEU	F	0.203	NEU
nsp7	1	49	E	**1.138**	MAX	E	-1.624	MAX
nsp7	1	50	K	0.341	MAX	K	-0.981	NEU
nsp7	1	51	F	**1.321**	MIN	M	0.714	NEU
nsp7	1	52	V	**1.011**	MIN	V	0.878	MIN
nsp7	1	53	S	0.377	NEU	S	-0.695	NEU
nsp7	1	54	L	**1.321**	MIN	L	1.186	MIN
nsp7	1	55	L	**1.321**	MIN	L	1.067	MIN
nsp7	1	56	A	**1.138**	NEU	S	-0.664	NEU
nsp7	1	57	T	0.377	NEU	V	1.299	MIN
nsp7	1	58	L	**1.321**	MIN	L	1.054	MIN
nsp7	1	59	M	0.330	MIN	L	0.827	MIN
nsp7	1	60	S	**1.321**	NEU	S	-0.303	NEU
nsp7	1	61	F	**1.138**	MIN	M	1.016	MIN
nsp7	1	62	S	0.280	NEU	Q	-1.115	MAX
nsp7	1	63	G	**1.138**	NEU	G	-0.487	NEU
nsp7	1	64	N	**0.671**	NEU	A	0.099	NEU
nsp7	1	65	V	**1.321**	MIN	V	1.232	MIN
nsp7	1	66	D	-0.064	MAX	D	-0.531	NEU
nsp7	1	67	L	**1.321**	MIN	I	1.009	MIN
nsp7	1	68	E	0.212	MAX	N	0.055	NEU
nsp7	1	69	A	0.376	NEU	K	0.45	NEU
nsp7	1	70	L	**1.321**	MIN	L	0.993	MIN
nsp7	1	71	A	**0.610**	MIN	C	2.491	MIN
nsp7	1	72	S	0.194	NEU	E	2.043	MIN
nsp8	1	1	F	0.294	MIN	F	1.311	MIN

nsp8	1	2	S	**0.565**	NEU	S	-0.555	NEU
nsp8	1	3	N	-0.008	NEU	S	1.005	MIN
nsp8	1	4	L	**0.911**	MIN	L	1.278	MIN
nsp8	1	5	P	**1.265**	NEU	P	-0.302	NEU
nsp8	1	6	S	**1.265**	NEU	S	-0.74	NEU
nsp8	1	7	Y	**0.698**	NEU	Y	0.054	NEU
nsp8	1	8	V	**0.911**	MIN	A	0.545	NEU
nsp8	1	9	A	0.359	NEU	A	0.502	NEU
nsp8	1	10	Y	**0.911**	NEU	F	0.375	NEU
nsp8	1	11	E	**0.698**	NEU	A	0.345	NEU
nsp8	1	12	N	**0.565**	NEU	T	0.268	NEU
nsp8	1	13	A	**0.911**	NEU	A	0.524	NEU
nsp8	1	14	Q	-0.022	NEU	Q	-0.845	NEU
nsp8	1	15	K	-0.008	NEU	E	-1.252	MAX
nsp8	1	16	A	**0.565**	MIN	A	0.688	NEU
nsp8	1	17	Y	0.359	NEU	Y	-0.353	NEU
nsp8	1	18	D	0.268	NEU	E	-1.369	MAX
nsp8	1	19	Q	-0.008	NEU	Q	-1.202	MAX
nsp8	1	20	A	**1.265**	MIN	A	0.795	MIN
nsp8	1	21	V	0.294	MIN	V	0.454	NEU
nsp8	1	22	A	0.428	NEU	A	-0.785	NEU
nsp8	1	23	N	0.359	NEU	N	-0.526	NEU
nsp8	1	24	G	**0.911**	NEU	G	-0.635	NEU
nsp8	1	25	D	0.268	NEU	D	-1.041	MAX
nsp8	1	26	S	**1.265**	NEU	S	-0.695	NEU
nsp8	1	27	E	-0.135	NEU	E	-0.29	NEU
nsp8	1	28	S	**1.265**	NEU	V	-0.298	NEU
nsp8	1	29	V	0.268	MIN	V	0.865	MIN
nsp8	1	30	L	**1.265**	MIN	L	1.12	MIN
nsp8	1	31	K	**0.543**	MAX	K	-1.474	MAX
nsp8	1	32	A	0.268	NEU	K	-1.822	MAX
nsp8	1	33	L	**0.911**	MIN	L	1.349	MIN
nsp8	1	34	K	**1.265**	MAX	K	-1.405	MAX
nsp8	1	35	K	**1.265**	MAX	K	-2.147	MAX
nsp8	1	36	A	-0.008	MIN	S	-0.706	NEU
nsp8	1	37	M	0.268	MIN	L	1.219	MIN
nsp8	1	38	N	0.346	NEU	N	0.602	NEU
nsp8	1	39	V	**0.543**	MIN	V	0.299	NEU
nsp8	1	40	A	0.294	NEU	A	0.648	NEU
nsp8	1	41	K	**0.911**	NEU	K	-0.713	NEU
nsp8	1	42	S	**0.698**	NEU	S	0.235	NEU
nsp8	1	43	V	0.268	NEU	E	-0.781	NEU
nsp8	1	44	L	**0.911**	MIN	F	1.152	MIN
nsp8	1	45	D	**0.911**	NEU	D	-0.592	NEU
nsp8	1	46	R	-0.008	NEU	R	-0.255	NEU
nsp8	1	47	D	**0.911**	MAX	D	-1.526	MAX

nsp8	1	48	A	**0.698**	MIN	A	0.782	MIN
nsp8	1	49	A	**1.265**	NEU	A	-0.864	NEU
nsp8	1	50	M	**0.543**	NEU	M	0.355	NEU
nsp8	1	51	Q	-0.301	NEU	Q	-1.029	MAX
nsp8	1	52	R	**0.911**	NEU	R	-0.748	NEU
nsp8	1	53	K	**0.698**	MAX	K	-1.175	MAX
nsp8	1	54	L	0.428	MIN	L	1.489	MIN
nsp8	1	55	E	0.346	NEU	E	0.433	NEU
nsp8	1	56	R	0.268	MAX	K	-1.229	MAX
nsp8	1	57	M	0.294	NEU	M	0.513	NEU
nsp8	1	58	A	0.294	MIN	A	0.933	MIN
nsp8	1	59	D	**0.698**	NEU	D	-0.677	NEU
nsp8	1	60	Q	-0.008	NEU	Q	-0.608	NEU
nsp8	1	61	A	0.346	MIN	A	0.693	NEU
nsp8	1	62	M	**0.698**	NEU	M	0.388	NEU
nsp8	1	63	T	0.346	NEU	T	0.596	NEU
nsp8	1	64	Q	0.034	NEU	Q	-0.896	NEU
nsp8	1	65	M	**0.543**	MIN	M	0.567	NEU
nsp8	1	66	Y	0.268	MAX	Y	-2.64	MAX
nsp8	1	67	K	**1.265**	MAX	K	-1.732	MAX
nsp8	1	68	Q	**0.698**	MAX	Q	-1.194	MAX
nsp8	1	69	A	**0.911**	NEU	A	0.142	NEU
nsp8	1	70	R	**1.265**	MIN	R	1.675	MIN
nsp8	1	71	A	**1.265**	NEU	S	-0.521	NEU
nsp8	1	72	E	0.346	MIN	E	0.56	NEU
nsp8	1	73	D	**1.265**	MIN	D	1.039	MIN
nsp8	1	74	K	0.428	MAX	K	-1.391	MAX
nsp8	1	75	R	-0.073	MIN	R	1.364	MIN
nsp8	1	76	A	**0.543**	NEU	A	0.028	NEU
nsp8	1	77	K	0.106	NEU	K	-0.594	NEU
nsp8	1	78	V	**0.911**	MIN	V	1.163	MIN
nsp8	1	79	T	0.268	NEU	T	0.164	NEU
nsp8	1	80	S	**1.265**	NEU	S	-0.75	NEU
nsp8	1	81	A	**0.543**	MIN	A	0.828	MIN
nsp8	1	82	M	**0.911**	MIN	M	0.604	NEU
nsp8	1	83	Q	**1.265**	NEU	Q	-0.805	NEU
nsp8	1	84	T	**0.911**	NEU	T	0.148	NEU
nsp8	1	85	M	**0.698**	NEU	M	0.161	NEU
nsp8	1	86	L	**1.265**	MIN	L	1.473	MIN
nsp8	1	87	F	**0.911**	MIN	F	0.729	NEU
nsp8	1	88	N	**1.265**	NEU	T	0.326	NEU
nsp8	1	89	M	0.294	MIN	M	0.324	NEU
nsp8	1	90	L	**1.265**	MIN	L	1.127	MIN
nsp8	1	91	R	**0.543**	NEU	R	0.515	NEU
nsp8	1	92	R	-0.022	MIN	K	0.329	NEU
nsp8	1	93	L	**1.265**	MIN	L	1.243	MIN

nsp8	1	94	D	**0.911**	NEU	D	-0.717	NEU
nsp8	1	95	N	**1.265**	NEU	N	-0.197	NEU
nsp8	1	96	D	0.034	MIN	D	0.593	NEU
nsp8	1	97	A	-0.008	NEU	A	0.142	NEU
nsp8	1	98	L	**1.265**	MIN	L	0.975	MIN
nsp8	1	99	N	**1.265**	NEU	N	0.548	NEU
nsp8	1	100	N	0.428	MIN	N	1.554	MIN
nsp8	1	101	I	**1.265**	MIN	I	1.486	MIN
nsp8	1	102	I	**0.911**	MIN	I	1.315	MIN
nsp8	1	103	N	0.212	MIN	N	1.19	MIN
nsp8	1	104	N	0.346	NEU	N	-0.409	NEU
nsp8	1	105	A	**0.911**	MIN	A	0.758	NEU
nsp8	1	106	R	-0.008	MIN	R	1.167	MIN
nsp8	1	107	N	0.359	NEU	D	-0.703	NEU
nsp8	1	108	G	0.294	NEU	G	-1.007	MAX
nsp8	1	109	C	**0.543**	NEU	C	0.185	NEU
nsp8	1	110	V	**1.265**	MIN	V	1.278	MIN
nsp8	1	111	P	0.268	NEU	P	-1.129	MAX
nsp8	1	112	L	**1.265**	MIN	L	0.847	MIN
nsp8	1	113	N	**1.265**	NEU	N	-0.466	NEU
nsp8	1	114	I	**0.911**	MIN	I	1.244	MIN
nsp8	1	115	I	**1.265**	MIN	I	1.406	MIN
nsp8	1	116	P	**0.543**	NEU	P	-0.46	NEU
nsp8	1	117	L	0.294	MIN	L	1.169	MIN
nsp8	1	118	T	**0.911**	NEU	T	-0.457	NEU
nsp8	1	119	A	**0.911**	NEU	T	-0.313	NEU
nsp8	1	120	A	**0.698**	NEU	A	-0.288	NEU
nsp8	1	121	N	-0.022	MIN	A	0.606	NEU
nsp8	1	122	K	0.428	MAX	K	-0.987	NEU
nsp8	1	123	L	**1.265**	MIN	L	1.527	MIN
nsp8	1	124	M	**0.543**	MIN	M	0.822	MIN
nsp8	1	125	V	**1.265**	MIN	V	1.253	MIN
nsp8	1	126	V	**1.265**	MIN	V	1.667	MIN
nsp8	1	127	V	**1.265**	MIN	I	1.318	MIN
nsp8	1	128	P	**1.265**	NEU	P	-0.399	NEU
nsp8	1	129	D	0.346	MAX	D	-0.842	NEU
nsp8	1	130	Y	-0.135	NEU	Y	-0.738	NEU
nsp8	1	131	S	0.346	MIN	N	1.305	MIN
nsp8	1	132	V	0.294	MIN	T	-0.073	NEU
nsp8	1	133	Y	0.428	NEU	Y	-0.03	NEU
nsp8	1	134	K	0.294	NEU	K	1.114	MIN
nsp8	1	135	N	0.023	NEU	N	-0.166	NEU
nsp8	1	136	T	**0.698**	NEU	T	-0.421	NEU
nsp8	1	137	C	**1.265**	MIN	C	1.078	MIN
nsp8	1	138	T	-0.008	NEU	D	-0.127	NEU
nsp8	1	139	G	0.268	NEU	G	-0.052	NEU

nsp8	1	140	P	0.346	MAX	T	-0.193	NEU
nsp8	1	141	T	0.428	NEU	T	-0.116	NEU
nsp8	1	142	L	**1.265**	MIN	F	0.877	MIN
nsp8	1	143	T	**0.911**	NEU	T	-0.202	NEU
nsp8	1	144	Y	**0.565**	NEU	Y	-0.472	NEU
nsp8	1	145	A	**0.565**	NEU	A	-0.221	NEU
nsp8	1	146	G	0.106	NEU	S	0.918	MIN
nsp8	1	147	A	**0.911**	NEU	A	0.289	NEU
nsp8	1	148	L	0.346	MIN	L	0.83	MIN
nsp8	1	149	W	0.268	MIN	W	0.546	NEU
nsp8	1	150	D	**0.543**	MAX	E	-0.912	NEU
nsp8	1	151	V	**1.265**	MIN	I	1.917	MIN
nsp8	1	152	Q	0.294	NEU	Q	-0.6	NEU
nsp8	1	153	Q	**0.543**	NEU	Q	-0.969	NEU
nsp8	1	154	V	**1.265**	MIN	V	1.423	MIN
nsp8	1	155	V	0.428	MIN	V	1.398	MIN
nsp8	1	156	D	**0.698**	MAX	D	-1.37	MAX
nsp8	1	157	A	**1.265**	NEU	A	-0.176	NEU
nsp8	1	158	D	**0.543**	NEU	D	0.269	NEU
nsp8	1	159	G	**0.911**	NEU	S	-0.676	NEU
nsp8	1	160	K	**0.565**	NEU	K	-0.412	NEU
nsp8	1	161	I	-0.008	NEU	I	1.785	MIN
nsp8	1	162	V	**1.265**	MIN	V	1.316	MIN
nsp8	1	163	N	-0.022	NEU	Q	-1.34	MAX
nsp8	1	164	L	0.212	MIN	L	0.841	MIN
nsp8	1	165	S	**0.698**	NEU	S	-0.217	NEU
nsp8	1	166	D	0.268	NEU	E	-1.923	MAX
nsp8	1	167	I	**1.265**	MIN	I	2.308	MIN
nsp8	1	168	T	0.294	MIN	S	1.133	MIN
nsp8	1	169	R	0.273	NEU	M	-0.256	NEU
nsp8	1	170	D	0.034	NEU	D	0.735	NEU
nsp8	1	171	N	**0.543**	NEU	N	-0.308	NEU
nsp8	1	172	S	**1.258**	NEU	S	-0.609	NEU
nsp8	1	173	P	0.060	NEU	P	0.601	NEU
nsp8	1	174	N	**0.666**	MIN	N	0.991	MIN
nsp8	1	175	L	**0.887**	MIN	L	1.449	MIN
nsp8	1	176	A	0.034	MAX	A	-1.176	MAX
nsp8	1	177	W	**0.543**	NEU	W	0.662	NEU
nsp8	1	178	P	**0.543**	NEU	P	-0.975	NEU
nsp8	1	179	L	**1.265**	MIN	L	1.333	MIN
nsp8	1	180	V	**0.698**	MIN	I	1.31	MIN
nsp8	1	181	V	**1.265**	MIN	V	1.326	MIN
nsp8	1	182	T	**1.265**	NEU	T	-0.087	NEU
nsp8	1	183	A	**1.265**	MIN	A	0.761	NEU
nsp8	1	184	L	0.268	MIN	L	1.487	MIN
nsp8	1	185	R	**0.911**	NEU	R	-0.794	NEU

nsp8	1	186	A	**0.508**	NEU	A	0.005	NEU
nsp8	1	187	N	0.060	NEU	N	-0.845	NEU
nsp9	1	1	N	**1.330**	NEU	N	-0.418	NEU
nsp9	1	2	N	0.335	NEU	N	-1.064	MAX
nsp9	1	3	E	-0.203	NEU	E	-0.678	NEU
nsp9	1	4	L	**0.771**	NEU	L	-0.841	NEU
nsp9	1	5	M	0.008	NEU	S	-0.496	NEU
nsp9	1	6	P	0.115	NEU	P	-0.774	NEU
nsp9	1	7	Q	**0.574**	NEU	V	0.605	NEU
nsp9	1	8	G	0.219	NEU	A	-1.379	MAX
nsp9	1	9	L	**1.330**	MIN	L	1.517	MIN
nsp9	1	10	K	0.219	NEU	R	0.828	MIN
nsp9	1	11	T	**0.982**	NEU	Q	-0.953	NEU
nsp9	1	12	M	-0.153	MIN	M	0.498	NEU
nsp9	1	13	V	0.056	NEU	S	-0.411	NEU
nsp9	1	14	V	**1.330**	MIN	C	1.109	MIN
nsp9	1	15	N	**0.771**	NEU	A	-0.152	NEU
nsp9	1	16	A	0.331	MIN	A	0.733	NEU
nsp9	1	17	G	**1.330**	NEU	G	-0.327	NEU
nsp9	1	18	P	-0.203	NEU	T	-0.038	NEU
nsp9	1	19	D	0.005	NEU	T	-0.525	NEU
nsp9	1	20	Q	0.056	NEU	Q	-1.046	MAX
nsp9	1	21	T	-0.011	NEU	T	0.618	NEU
nsp9	1	22	-	0.169	MIN	A	-0.707	NEU
nsp9	1	23	C	**0.982**	MIN	C	0.783	MIN
nsp9	1	24	N	**0.581**	MIN	T	0.131	NEU
nsp9	1	25	T	0.056	NEU	D	0.026	NEU
nsp9	1	26	-	**1.258**	NEU	D	-0.495	NEU
nsp9	1	27	P	**0.624**	NEU	N	-0.603	NEU
nsp9	1	28	T	**0.771**	NEU	A	0.05	NEU
nsp9	1	29	L	0.443	MIN	L	1.123	MIN
nsp9	1	30	A	0.364	NEU	A	0.397	NEU
nsp9	1	31	Y	0.443	NEU	Y	0.531	NEU
nsp9	1	32	Y	0.364	NEU	Y	0.194	NEU
nsp9	1	33	N	**1.179**	NEU	N	-0.802	NEU
nsp9	1	34	P	0.363	NEU	T	0.158	NEU
nsp9	1	35	V	0.342	MIN	T	-0.255	NEU
nsp9	1	36	Q	0.189	MIN	K	1.573	MIN
nsp9	1	37	G	0.419	NEU	G	0.202	NEU
nsp9	1	38	G	0.260	NEU	G	0.478	NEU
nsp9	1	39	R	0.352	NEU	R	0.429	NEU
nsp9	1	40	M	0.331	MIN	F	1.49	MIN
nsp9	1	41	V	**1.330**	MIN	V	1.844	MIN
nsp9	1	42	M	**1.179**	MIN	L	1.324	MIN
nsp9	1	43	A	**1.330**	NEU	A	0.121	NEU
nsp9	1	44	I	**1.330**	MIN	L	1.552	MIN

nsp9	1	45	L	**1.330**	MIN	L	1.307	MIN
nsp9	1	46	S	**0.771**	NEU	S	-0.815	NEU
nsp9	1	47	D	**0.502**	NEU	D	0.942	MIN
nsp9	1	48	N	-0.004	MIN	L	1.018	MIN
nsp9	1	49	D	**0.663**	MAX	Q	-0.844	NEU
nsp9	1	50	G	0.467	NEU	D	-0.499	NEU
nsp9	1	51	L	**1.330**	MIN	L	1.103	MIN
nsp9	1	52	K	-0.041	MAX	K	-1.748	MAX
nsp9	1	53	Y	0.342	NEU	W	0.089	NEU
nsp9	1	54	A	0.397	MIN	A	0.599	NEU
nsp9	1	55	K	0.471	MAX	R	-0.07	NEU
nsp9	1	56	V	**1.330**	MIN	F	0.972	MIN
nsp9	1	57	E	0.330	MIN	P	0.708	NEU
nsp9	1	58	K	0.229	NEU	K	-1.758	MAX
nsp9	1	59	S	**0.574**	MIN	S	0.715	NEU
nsp9	1	60	D	0.116	NEU	D	2.477	MIN
nsp9	1	61	G	-0.179	MIN	G	2.005	MIN
nsp9	1	62	-	**1.250**	MIN	T	0.716	NEU
nsp9	1	63	G	0.364	NEU	G	1.497	MIN
nsp9	1	64	F	0.335	MIN	T	0.119	NEU
nsp9	1	65	V	**1.330**	MIN	I	1.646	MIN
nsp9	1	66	V	0.028	NEU	Y	-1.385	MAX
nsp9	1	67	L	**0.834**	MIN	T	-0.175	NEU
nsp9	1	68	E	**0.834**	NEU	E	-0.477	NEU
nsp9	1	69	L	**1.179**	MIN	L	1.319	MIN
nsp9	1	70	Q	**0.833**	NEU	E	-0.808	NEU
nsp9	1	71	P	**1.330**	NEU	P	-0.814	NEU
nsp9	1	72	P	**0.834**	MAX	P	-1.013	MAX
nsp9	1	73	C	**1.330**	MIN	C	2.089	MIN
nsp9	1	74	K	0.364	NEU	R	-0.412	NEU
nsp9	1	75	F	0.419	NEU	F	0.695	NEU
nsp9	1	76	L	0.364	MIN	V	0.572	NEU
nsp9	1	77	I	**0.771**	MIN	T	0.119	NEU
nsp9	1	78	A	-0.097	MAX	D	1.653	MIN
nsp9	1	79	G	0.331	MIN	T	0.194	NEU
nsp9	1	80	P	0.077	MIN	P	1.254	MIN
nsp9	1	81	K	0.471	MIN	K	0.118	NEU
nsp9	1	82	G	**1.179**	NEU	G	0.252	NEU
nsp9	1	83	P	0.208	NEU	P	-1.693	MAX
nsp9	1	84	K	0.342	NEU	K	-0.164	NEU
nsp9	1	85	I	**1.179**	MIN	V	0.997	MIN
nsp9	1	86	K	0.342	MAX	K	-1.326	MAX
nsp9	1	87	Y	0.443	NEU	Y	0.802	MIN
nsp9	1	88	L	**1.179**	MIN	L	1.526	MIN
nsp9	1	89	Y	**0.903**	MIN	Y	0.551	NEU
nsp9	1	90	F	**1.330**	MIN	F	1.348	MIN

nsp9	1	91	V	**0.714**	MIN	I	1.312	MIN
nsp9	1	92	K	**0.568**	MAX	K	-1.055	MAX
nsp9	1	93	G	**1.330**	NEU	G	-0.562	NEU
nsp9	1	94	L	**1.330**	MIN	L	1.294	MIN
nsp9	1	95	N	**0.714**	NEU	N	0.117	NEU
nsp9	1	96	N	**0.834**	NEU	N	1.008	MIN
nsp9	1	97	L	**0.714**	NEU	L	-0.777	NEU
nsp9	1	98	H	**0.663**	NEU	N	-0.819	NEU
nsp9	1	99	R	**1.330**	NEU	R	-0.536	NEU
nsp9	1	100	G	**1.330**	NEU	G	-0.63	NEU
nsp9	1	101	Q	-0.130	NEU	M	0.742	NEU
nsp9	1	102	V	**1.330**	MIN	V	1.199	MIN
nsp9	1	103	L	**1.330**	MIN	L	1.395	MIN
nsp9	1	104	G	**1.179**	NEU	G	-0.215	NEU
nsp9	1	105	T	**1.330**	NEU	S	-0.804	NEU
nsp9	1	106	I	**1.330**	MIN	L	1.296	MIN
nsp9	1	107	A	**0.834**	NEU	A	0.231	NEU
nsp9	1	108	A	**0.922**	NEU	A	-0.072	NEU
nsp9	1	109	T	**0.771**	NEU	T	0.078	NEU
nsp9	1	110	V	**0.903**	MIN	V	1.379	MIN
nsp9	1	111	R	0.397	MIN	R	1.566	MIN
nsp9	1	112	L	**0.771**	MIN	L	1.277	MIN
nsp9	1	113	Q	**1.072**	NEU	Q	-0.736	NEU
nsp10	1	1	A	0.020	NEU	A	0.676	NEU
nsp10	1	2	F	0.476	NEU	F	0.699	NEU
nsp10	1	3	A	**0.691**	NEU	A	0.657	NEU
nsp10	1	4	V	**0.715**	NEU	V	0.627	NEU
nsp10	1	5	D	**0.856**	NEU	D	-0.754	NEU
nsp10	1	6	P	0.137	MAX	A	0.728	NEU
nsp10	1	7	A	0.126	NEU	A	0.634	NEU
nsp10	1	8	K	0.341	NEU	K	-1.146	MAX
nsp10	1	9	A	**1.325**	NEU	A	0.125	NEU
nsp10	1	10	Y	**1.039**	NEU	Y	-0.368	NEU
nsp10	1	11	K	-0.177	NEU	K	-1.161	MAX
nsp10	1	12	D	**0.656**	NEU	D	-0.464	NEU
nsp10	1	13	Y	-0.124	MAX	Y	-1.115	MAX
nsp10	1	14	L	0.325	MIN	L	-0.184	NEU
nsp10	1	15	N	0.355	NEU	A	0.049	NEU
nsp10	1	16	S	**1.156**	NEU	S	0.08	NEU
nsp10	1	17	G	**1.039**	NEU	G	-0.415	NEU
nsp10	1	18	G	**1.325**	NEU	G	-0.173	NEU
nsp10	1	19	A	-0.036	NEU	Q	-1.261	MAX
nsp10	1	20	P	**1.039**	NEU	P	-0.563	NEU
nsp10	1	21	I	**1.325**	MIN	I	1.66	MIN
nsp10	1	22	T	**0.872**	NEU	T	-0.063	NEU
nsp10	1	23	N	**1.325**	NEU	N	-0.15	NEU

nsp10	1	24	C	**1.325**	MIN	C	1.77	MIN
nsp10	1	25	V	**1.325**	MIN	V	0.786	MIN
nsp10	1	26	K	**1.156**	NEU	K	-0.301	NEU
nsp10	1	27	M	**1.156**	MIN	M	0.736	NEU
nsp10	1	28	L	**1.156**	NEU	L	-0.618	NEU
nsp10	1	29	T	0.354	NEU	C	2.427	MIN
nsp10	1	30	P	**0.872**	NEU	T	0.226	NEU
nsp10	1	31	H	-0.215	NEU	H	0.544	NEU
nsp10	1	32	T	**0.656**	NEU	T	0.516	NEU
nsp10	1	33	G	**1.156**	NEU	G	0.146	NEU
nsp10	1	34	T	0.415	NEU	T	0.683	NEU
nsp10	1	35	G	**1.325**	NEU	G	0.157	NEU
nsp10	1	36	I	0.415	MIN	Q	-0.526	NEU
nsp10	1	37	A	**1.325**	NEU	A	0.281	NEU
nsp10	1	38	I	**1.325**	MIN	I	0.94	MIN
nsp10	1	39	T	**0.656**	NEU	T	0.651	NEU
nsp10	1	40	V	0.203	MIN	V	1.48	MIN
nsp10	1	41	K	-0.030	NEU	T	1.033	MIN
nsp10	1	42	P	**0.941**	NEU	P	-0.396	NEU
nsp10	1	43	E	0.349	NEU	E	-0.916	NEU
nsp10	1	44	A	**1.156**	NEU	A	-0.483	NEU
nsp10	1	45	N	0.354	MIN	N	0.733	NEU
nsp10	1	46	A	-0.205	NEU	M	-0.556	NEU
nsp10	1	47	D	0.396	NEU	D	-0.652	NEU
nsp10	1	48	Q	**1.325**	NEU	Q	-0.904	NEU
nsp10	1	49	E	**0.781**	NEU	E	-0.862	NEU
nsp10	1	50	S	**1.325**	NEU	S	-0.542	NEU
nsp10	1	51	F	**1.325**	MIN	F	0.975	MIN
nsp10	1	52	G	**1.325**	NEU	G	-0.133	NEU
nsp10	1	53	G	**1.325**	NEU	G	-0.006	NEU
nsp10	1	54	A	**1.156**	NEU	A	0.347	NEU
nsp10	1	55	S	**1.325**	NEU	S	-0.334	NEU
nsp10	1	56	C	**1.325**	MIN	C	1.184	MIN
nsp10	1	57	C	**1.325**	MIN	C	1.783	MIN
nsp10	1	58	L	**1.325**	MIN	L	1.329	MIN
nsp10	1	59	Y	0.261	MAX	Y	-0.483	NEU
nsp10	1	60	C	**1.325**	MIN	C	1.163	MIN
nsp10	1	61	R	**1.156**	NEU	R	-0.598	NEU
nsp10	1	62	C	**1.325**	MIN	C	1.3	MIN
nsp10	1	63	H	**1.325**	NEU	H	-0.261	NEU
nsp10	1	64	I	**1.325**	MIN	I	2.531	MIN
nsp10	1	65	E	-0.114	NEU	D	0.702	NEU
nsp10	1	66	H	**0.781**	NEU	H	0.144	NEU
nsp10	1	67	P	0.288	MIN	P	0.404	NEU
nsp10	1	68	D	0.444	NEU	N	-0.232	NEU
nsp10	1	69	V	-0.118	MIN	P	1.113	MIN

nsp10	1	70	S	0.288	NEU	K	0.198	NEU
nsp10	1	71	G	**0.856**	NEU	G	-0.368	NEU
nsp10	1	72	V	**0.556**	NEU	F	-1.308	MAX
nsp10	1	73	C	**1.325**	MIN	C	1.779	MIN
nsp10	1	74	K	0.341	MIN	D	0.941	MIN
nsp10	1	75	Y	**0.715**	MIN	L	1.095	MIN
nsp10	1	76	K	**0.656**	MAX	K	-1.804	MAX
nsp10	1	77	G	**1.325**	NEU	G	-0.641	NEU
nsp10	1	78	K	**0.656**	MAX	K	-1.469	MAX
nsp10	1	79	F	**1.039**	MIN	Y	0.563	NEU
nsp10	1	80	V	**1.325**	MIN	V	0.985	MIN
nsp10	1	81	Q	**1.325**	NEU	Q	-0.835	NEU
nsp10	1	82	I	**1.325**	MIN	I	0.88	MIN
nsp10	1	83	P	**0.715**	NEU	P	-0.942	NEU
nsp10	1	84	T	0.476	NEU	T	0.423	NEU
nsp10	1	85	Q	0.184	NEU	T	-0.417	NEU
nsp10	1	86	C	0.468	MIN	C	1.791	MIN
nsp10	1	87	V	0.341	NEU	A	0.163	NEU
nsp10	1	88	N	**0.691**	NEU	N	0.515	NEU
nsp10	1	89	D	**0.556**	MAX	D	-1.11	MAX
nsp10	1	90	P	**1.325**	NEU	P	-0.629	NEU
nsp10	1	91	V	**1.325**	MIN	V	1.221	MIN
nsp10	1	92	G	**1.325**	NEU	G	-0.487	NEU
nsp10	1	93	F	**1.325**	MIN	F	1.331	MIN
nsp10	1	94	C	**0.514**	MIN	T	-0.511	NEU
nsp10	1	95	L	**1.325**	MIN	L	1.13	MIN
nsp10	1	96	R	0.007	NEU	K	0.17	NEU
nsp10	1	97	N	**0.656**	MAX	N	-1.152	MAX
nsp10	1	98	T	0.325	MIN	T	-0.114	NEU
nsp10	1	99	V	**0.856**	NEU	V	0.402	NEU
nsp10	1	100	C	**1.325**	MIN	C	1.338	MIN
nsp10	1	101	N	**0.941**	NEU	T	-0.615	NEU
nsp10	1	102	V	0.415	MIN	V	1.116	MIN
nsp10	1	103	C	**1.325**	MIN	C	1.083	MIN
nsp10	1	104	Q	**0.514**	NEU	G	-0.583	NEU
nsp10	1	105	M	0.415	NEU	M	0.304	NEU
nsp10	1	106	W	**0.856**	MIN	W	0.805	MIN
nsp10	1	107	K	-0.012	MAX	K	-1.258	MAX
nsp10	1	108	G	**1.325**	NEU	G	0.069	NEU
nsp10	1	109	Y	0.370	NEU	Y	-0.835	NEU
nsp10	1	110	G	**1.325**	NEU	G	-0.198	NEU
nsp10	1	111	C	**1.325**	MIN	C	1.348	MIN
nsp10	1	112	N	**0.781**	NEU	S	-0.771	NEU
nsp10	1	113	C	**1.325**	MIN	C	1.457	MIN
nsp10	1	114	D	-0.042	NEU	D	1.659	MIN
nsp10	1	115	S	0.370	NEU	Q	1.077	MIN

nsp10	1	116	L	**1.032**	MIN	L	0.895	MIN
nsp10	1	117	R	0.012	NEU	R	1.633	MIN
nsp10	1	118	E	0.347	NEU	E	0.077	NEU
nsp10	1	119	P	0.199	NEU	P	0.564	NEU
nsp10	1	120	M	**0.764**	NEU	M	0.069	NEU
nsp10	1	121	L	**0.705**	NEU	L	-0.876	NEU
nsp10	1	122	Q	**1.032**	NEU	Q	0.796	MIN
nsp12	1	1	-	0.121	MIN	A	0.553	NEU
nsp12	1	2	T	0.368	NEU	Q	-0.874	NEU
nsp12	1	3	N	**0.539**	MIN	S	0.456	NEU
nsp12	1	4	F	**1.032**	MIN	F	1.182	MIN
nsp12	1	5	L	**1.152**	MIN	L	1.397	MIN
nsp12	1	6	N	**0.856**	NEU	N	-0.549	NEU
nsp12	1	7	R	**1.039**	NEU	R	-0.172	NEU
nsp12	1	8	V	**1.156**	MIN	V	1.097	MIN
nsp12	1	9	R	**0.781**	NEU	C	2.173	MIN
nsp12	1	10	G	**1.039**	NEU	G	-0.202	NEU
nsp12	1	11	T	**0.618**	NEU	V	-0.451	NEU
nsp12	1	12	S	**0.514**	NEU	S	-0.175	NEU
nsp12	1	13	V	0.494	NEU	A	-1.027	MAX
nsp12	1	14	A	0.182	MIN	A	0.267	NEU
nsp12	1	15	R	**1.325**	MIN	R	2.494	MIN
nsp12	1	16	L	**1.325**	MIN	L	1.403	MIN
nsp12	1	17	V	-0.114	MIN	T	-0.109	NEU
nsp12	1	18	P	**1.325**	NEU	P	-0.277	NEU
nsp12	1	19	C	**0.618**	MIN	C	0.212	NEU
nsp12	1	20	A	**0.656**	NEU	G	0.621	NEU
nsp12	1	21	S	0.332	NEU	T	-0.795	NEU
nsp12	1	22	G	**0.715**	NEU	G	0.16	NEU
nsp12	1	23	L	**0.603**	MIN	T	-0.581	NEU
nsp12	1	24	S	-0.003	NEU	S	0.651	NEU
nsp12	1	25	T	**0.656**	NEU	T	-0.666	NEU
nsp12	1	26	D	**1.325**	MAX	D	-1.349	MAX
nsp12	1	27	V	**1.325**	MIN	V	1.532	MIN
nsp12	1	28	V	**1.325**	NEU	V	-0.749	NEU
nsp12	1	29	L	0.096	MIN	Y	-0.842	NEU
nsp12	1	30	R	**1.325**	NEU	R	-0.684	NEU
nsp12	1	31	A	**1.325**	NEU	A	0.076	NEU
nsp12	1	32	F	0.415	MIN	F	0.991	MIN
nsp12	1	33	D	**1.325**	MAX	D	-1.206	MAX
nsp12	1	34	I	**1.325**	MIN	I	1.426	MIN
nsp12	1	35	C	-0.083	MIN	Y	-2.325	MAX
nsp12	1	36	N	0.332	MAX	N	-1.634	MAX
nsp12	1	37	A	**0.577**	NEU	D	0	NEU
nsp12	1	38	K	**0.781**	MIN	K	0.827	MIN
nsp12	1	39	V	0.327	NEU	V	1.003	MIN

nsp12	1	40	A	**1.325**	NEU	A	-0.602	NEU
nsp12	1	41	G	**1.325**	NEU	G	-0.397	NEU
nsp12	1	42	I	**1.325**	MIN	F	0.785	MIN
nsp12	1	43	G	**1.325**	NEU	A	0.206	NEU
nsp12	1	44	L	0.106	MIN	K	-1.407	MAX
nsp12	1	45	H	-0.219	MAX	F	0.319	NEU
nsp12	1	46	Y	-0.191	MIN	L	1.196	MIN
nsp12	1	47	K	**1.325**	MAX	K	-1.77	MAX
nsp12	1	48	T	-0.126	NEU	T	-0.012	NEU
nsp12	1	49	N	**0.941**	NEU	N	0.18	NEU
nsp12	1	50	C	0.476	MIN	C	1.406	MIN
nsp12	1	51	C	**1.325**	MIN	C	2.261	MIN
nsp12	1	52	R	**1.325**	NEU	R	-0.531	NEU
nsp12	1	53	F	**1.156**	MIN	F	1.354	MIN
nsp12	1	54	Q	-0.233	MAX	Q	-0.842	NEU
nsp12	1	55	E	0.355	NEU	E	-1.035	MAX
nsp12	1	56	L	0.476	MIN	K	0.786	MIN
nsp12	1	57	D	0.055	NEU	D	-0.074	NEU
nsp12	1	58	E	**0.577**	MIN	E	1.311	MIN
nsp12	1	59	D	-0.248	MAX	D	1.059	MIN
nsp12	1	60	G	**1.156**	NEU	D	1.614	MIN
nsp12	1	61	N	-0.036	NEU	N	0.823	MIN
nsp12	1	62	K	0.319	NEU	L	-0.357	NEU
nsp12	1	63	L	**1.156**	NEU	I	-0.8	NEU
nsp12	1	64	D	**1.156**	NEU	D	-0.571	NEU
nsp12	1	65	S	**1.039**	NEU	S	-0.781	NEU
nsp12	1	66	Y	**1.039**	MIN	Y	0.949	MIN
nsp12	1	67	F	**1.325**	MIN	F	1.037	MIN
nsp12	1	68	V	**1.325**	MIN	V	1.313	MIN
nsp12	1	69	V	**1.325**	MIN	V	1.204	MIN
nsp12	1	70	K	**0.514**	MAX	K	-1.654	MAX
nsp12	1	71	R	**1.325**	NEU	R	-0.558	NEU
nsp12	1	72	H	0.341	NEU	H	1.328	MIN
nsp12	1	73	T	**0.603**	NEU	T	0.069	NEU
nsp12	1	74	L	**1.039**	NEU	F	-0.336	NEU
nsp12	1	75	E	0.442	NEU	S	0.605	NEU
nsp12	1	76	N	**0.656**	NEU	N	-0.085	NEU
nsp12	1	77	Y	**0.941**	MAX	Y	-2.568	MAX
nsp12	1	78	N	-0.030	NEU	Q	0.661	NEU
nsp12	1	79	L	0.137	NEU	H	1.117	MIN
nsp12	1	80	E	**0.656**	MAX	E	-1.389	MAX
nsp12	1	81	K	0.216	NEU	E	-1.451	MAX
nsp12	1	82	E	0.441	NEU	T	0.349	NEU
nsp12	1	83	C	**1.156**	MIN	I	2.032	MIN
nsp12	1	84	Y	**0.872**	NEU	Y	0.094	NEU
nsp12	1	85	E	0.288	MIN	N	-0.834	NEU

nsp12	1	86	L	0.415	MIN	L	1.307	MIN
nsp12	1	87	L	**0.856**	MIN	L	1.396	MIN
nsp12	1	88	K	**0.552**	NEU	K	-1.192	MAX
nsp12	1	89	D	**0.514**	NEU	D	0.189	NEU
nsp12	1	90	C	**0.872**	MIN	C	1.173	MIN
nsp12	1	91	G	0.055	MIN	P	1.165	MIN
nsp12	1	92	V	0.332	MIN	A	0.104	NEU
nsp12	1	93	V	**1.325**	MIN	V	0.964	MIN
nsp12	1	94	A	**0.715**	MIN	A	0.791	MIN
nsp12	1	95	E	**0.514**	NEU	K	-0.709	NEU
nsp12	1	96	H	**1.325**	NEU	H	-0.283	NEU
nsp12	1	97	D	**1.325**	NEU	D	-0.43	NEU
nsp12	1	98	F	**0.856**	NEU	F	-0.542	NEU
nsp12	1	99	F	**1.325**	MIN	F	1.758	MIN
nsp12	1	100	T	-0.057	NEU	K	-1.944	MAX
nsp12	1	101	F	**1.325**	MIN	F	1.453	MIN
nsp12	1	102	D	-0.068	MIN	R	1.522	MIN
nsp12	1	103	V	**1.039**	MIN	I	2.194	MIN
nsp12	1	104	D	-0.101	NEU	D	0.981	MIN
nsp12	1	105	G	**0.514**	NEU	G	-0.484	NEU
nsp12	1	106	S	-0.169	MIN	D	-1.452	MAX
nsp12	1	107	M	0.114	NEU	M	0.629	NEU
nsp12	1	108	V	0.328	MIN	V	0.833	MIN
nsp12	1	109	P	**1.164**	NEU	P	-0.099	NEU
nsp12	1	110	H	**1.327**	NEU	H	0.143	NEU
nsp12	1	111	I	**0.735**	MIN	I	0.581	NEU
nsp12	1	112	V	0.386	MIN	S	-0.468	NEU
nsp12	1	113	R	**1.164**	NEU	R	-0.694	NEU
nsp12	1	114	Q	0.463	NEU	Q	-0.965	NEU
nsp12	1	115	R	0.211	NEU	R	0.766	NEU
nsp12	1	116	L	**1.050**	MIN	L	1.124	MIN
nsp12	1	117	T	**1.327**	NEU	T	-0.303	NEU
nsp12	1	118	K	0.353	MIN	K	0.917	MIN
nsp12	1	119	Y	0.434	NEU	Y	0.015	NEU
nsp12	1	120	T	**1.327**	NEU	T	-0.187	NEU
nsp12	1	121	M	0.386	MIN	M	0.836	MIN
nsp12	1	122	A	0.327	MIN	A	0.468	NEU
nsp12	1	123	D	**0.535**	MAX	D	-1.066	MAX
nsp12	1	124	L	**1.327**	MIN	L	1.567	MIN
nsp12	1	125	V	**1.327**	MIN	V	1.155	MIN
nsp12	1	126	Y	**1.164**	NEU	Y	-0.168	NEU
nsp12	1	127	A	0.408	NEU	A	-0.19	NEU
nsp12	1	128	L	**1.327**	MIN	L	1.326	MIN
nsp12	1	129	R	**1.327**	NEU	R	-0.489	NEU
nsp12	1	130	H	**1.327**	NEU	H	-0.157	NEU
nsp12	1	131	F	0.353	NEU	F	0.574	NEU

nsp12	1	132	D	0.497	NEU	D	0.388	NEU
nsp12	1	133	R	0.030	NEU	E	-0.732	NEU
nsp12	1	134	N	**0.677**	MIN	G	0.356	NEU
nsp12	1	135	N	0.327	MIN	N	0.23	NEU
nsp12	1	136	C	**1.326**	MIN	C	1.9	MIN
nsp12	1	137	E	0.368	MIN	D	0.946	MIN
nsp12	1	138	T	0.209	MIN	T	-0.048	NEU
nsp12	1	139	L	**1.327**	MIN	L	1.397	MIN
nsp12	1	140	K	0.027	MAX	K	-0.857	NEU
nsp12	1	141	E	**0.578**	NEU	E	-0.567	NEU
nsp12	1	142	I	**1.327**	MIN	I	2.054	MIN
nsp12	1	143	L	**1.327**	MIN	L	1.485	MIN
nsp12	1	144	V	0.386	MIN	V	0.345	NEU
nsp12	1	145	L	0.239	NEU	T	0.423	NEU
nsp12	1	146	Y	**0.735**	NEU	Y	0.417	NEU
nsp12	1	147	G	0.158	NEU	N	0.25	NEU
nsp12	1	148	C	0.408	MIN	C	1.852	MIN
nsp12	1	149	C	**1.327**	MIN	C	2.059	MIN
nsp12	1	150	D	0.361	MAX	D	-1.287	MAX
nsp12	1	151	E	-0.083	NEU	D	-0.587	NEU
nsp12	1	152	S	0.386	MIN	D	1.264	MIN
nsp12	1	153	Y	0.423	MAX	Y	-2.179	MAX
nsp12	1	154	F	0.463	MIN	F	0.194	NEU
nsp12	1	155	D	**1.050**	NEU	N	0.088	NEU
nsp12	1	156	K	0.192	NEU	K	0.905	MIN
nsp12	1	157	K	**0.677**	MIN	K	1.014	MIN
nsp12	1	158	D	0.408	NEU	D	0.18	NEU
nsp12	1	159	W	0.386	NEU	W	-0.879	NEU
nsp12	1	160	Y	0.161	MAX	Y	-1.199	MAX
nsp12	1	161	D	**1.327**	NEU	D	-0.407	NEU
nsp12	1	162	F	**0.535**	MIN	F	1.102	MIN
nsp12	1	163	V	**1.050**	MIN	V	1.098	MIN
nsp12	1	164	E	**0.714**	NEU	E	0.149	NEU
nsp12	1	165	N	**1.327**	NEU	N	-0.637	NEU
nsp12	1	166	P	**1.164**	NEU	P	-0.489	NEU
nsp12	1	167	D	0.434	NEU	D	0.69	NEU
nsp12	1	168	I	**1.327**	MIN	I	1.898	MIN
nsp12	1	169	I	**1.327**	MIN	L	1.225	MIN
nsp12	1	170	N	0.408	MIN	R	0.773	NEU
nsp12	1	171	V	**1.327**	MIN	V	1.257	MIN
nsp12	1	172	Y	**1.050**	NEU	Y	0.22	NEU
nsp12	1	173	H	**0.535**	MAX	A	0.306	NEU
nsp12	1	174	K	**0.800**	NEU	N	-0.294	NEU
nsp12	1	175	L	**1.327**	MIN	L	1.395	MIN
nsp12	1	176	G	**1.327**	NEU	G	-0.788	NEU
nsp12	1	177	E	0.126	NEU	E	-0.958	NEU

nsp12	1	178	I	0.341	NEU	R	-0.095	NEU
nsp12	1	179	V	**1.327**	MIN	V	1.297	MIN
nsp12	1	180	R	0.386	NEU	R	-0.443	NEU
nsp12	1	181	R	0.386	NEU	Q	-1.157	MAX
nsp12	1	182	A	**1.327**	NEU	A	-0.142	NEU
nsp12	1	183	L	**1.050**	MIN	L	1.128	MIN
nsp12	1	184	L	**0.955**	MIN	L	1.039	MIN
nsp12	1	185	N	**0.577**	NEU	K	-1.317	MAX
nsp12	1	186	T	**0.873**	NEU	T	-0.463	NEU
nsp12	1	187	V	0.353	MIN	V	1.633	MIN
nsp12	1	188	K	0.126	NEU	Q	-0.837	NEU
nsp12	1	189	F	**0.677**	MIN	F	0.746	NEU
nsp12	1	190	A	0.463	MIN	C	0.959	MIN
nsp12	1	191	D	0.304	NEU	D	0.746	NEU
nsp12	1	192	A	**0.890**	NEU	A	0.348	NEU
nsp12	1	193	M	**0.624**	MIN	M	0.701	NEU
nsp12	1	194	V	**0.677**	MIN	R	-0.465	NEU
nsp12	1	195	E	0.328	NEU	N	-0.459	NEU
nsp12	1	196	A	0.199	MIN	A	0.719	NEU
nsp12	1	197	G	**1.327**	NEU	G	-0.004	NEU
nsp12	1	198	L	**1.327**	MIN	I	1.383	MIN
nsp12	1	199	V	**1.327**	MIN	V	1.184	MIN
nsp12	1	200	G	**1.327**	NEU	G	-0.451	NEU
nsp12	1	201	V	**1.327**	MIN	V	1.719	MIN
nsp12	1	202	L	**1.327**	MIN	L	0.972	MIN
nsp12	1	203	T	**1.327**	NEU	T	-0.364	NEU
nsp12	1	204	L	**1.327**	MIN	L	1.175	MIN
nsp12	1	205	D	**1.164**	MAX	D	-1.331	MAX
nsp12	1	206	N	**1.050**	NEU	N	-0.958	NEU
nsp12	1	207	Q	0.341	NEU	Q	-1.013	MAX
nsp12	1	208	D	0.368	NEU	D	-0.961	NEU
nsp12	1	209	L	**1.327**	MIN	L	1.362	MIN
nsp12	1	210	N	-0.072	MIN	N	1.043	MIN
nsp12	1	211	G	**1.327**	NEU	G	-0.481	NEU
nsp12	1	212	K	**0.535**	NEU	N	-0.051	NEU
nsp12	1	213	W	**0.604**	NEU	W	0.463	NEU
nsp12	1	214	Y	**0.714**	MAX	Y	-2.02	MAX
nsp12	1	215	D	0.497	NEU	D	-0.868	NEU
nsp12	1	216	F	0.328	MIN	F	0.494	NEU
nsp12	1	217	G	**1.327**	NEU	G	-0.504	NEU
nsp12	1	218	D	**1.164**	NEU	D	0.165	NEU
nsp12	1	219	F	**1.327**	MIN	F	1.297	MIN
nsp12	1	220	V	**1.327**	MIN	I	2.184	MIN
nsp12	1	221	I	-0.080	MIN	Q	-0.648	NEU
nsp12	1	222	T	**0.873**	NEU	T	-0.369	NEU
nsp12	1	223	A	0.034	NEU	T	0.521	NEU

nsp12	1	224	P	0.333	MAX	P	-0.458	NEU
nsp12	1	225	G	0.497	NEU	G	0.701	NEU
nsp12	1	226	C	**0.577**	NEU	S	-0.121	NEU
nsp12	1	227	G	**1.327**	NEU	G	-0.041	NEU
nsp12	1	228	V	**1.327**	MIN	V	1.673	MIN
nsp12	1	229	A	0.126	NEU	P	-1.118	MAX
nsp12	1	230	V	**1.327**	MIN	V	1.388	MIN
nsp12	1	231	V	0.341	MIN	V	1.488	MIN
nsp12	1	232	D	**0.735**	MAX	D	-0.63	NEU
nsp12	1	233	S	**1.327**	NEU	S	-0.577	NEU
nsp12	1	234	Y	0.333	MIN	Y	0.666	NEU
nsp12	1	235	Y	**1.327**	NEU	Y	-0.426	NEU
nsp12	1	236	S	**1.327**	NEU	S	-0.288	NEU
nsp12	1	237	Y	0.368	NEU	L	1.11	MIN
nsp12	1	238	L	0.333	NEU	L	1.344	MIN
nsp12	1	239	M	**1.327**	MIN	M	0.951	MIN
nsp12	1	240	P	**1.327**	MAX	P	-1.051	MAX
nsp12	1	241	M	**1.164**	MIN	I	1.312	MIN
nsp12	1	242	L	**0.535**	MIN	L	1.298	MIN
nsp12	1	243	T	**1.327**	NEU	T	-0.293	NEU
nsp12	1	244	M	0.408	MIN	L	1.246	MIN
nsp12	1	245	T	0.386	NEU	T	-0.325	NEU
nsp12	1	246	H	**0.873**	NEU	R	-0.84	NEU
nsp12	1	247	A	0.368	NEU	A	-0.425	NEU
nsp12	1	248	L	**1.327**	MIN	L	1.53	MIN
nsp12	1	249	A	0.192	MAX	T	-0.146	NEU
nsp12	1	250	A	**1.003**	NEU	A	-0.316	NEU
nsp12	1	251	E	**1.164**	NEU	E	0.06	NEU
nsp12	1	252	L	0.100	MIN	S	-0.666	NEU
nsp12	1	253	H	0.080	NEU	H	0.952	MIN
nsp12	1	254	-	0.120	MIN	V	1.292	MIN
nsp12	1	255	D	**1.308**	MIN	D	1.775	MIN
nsp12	1	256	-	-0.179	NEU	T	0.796	MIN
nsp12	1	257	D	-0.148	NEU	D	-0.932	NEU
nsp12	1	258	L	**0.735**	MIN	L	1.116	MIN
nsp12	1	259	N	0.100	NEU	T	0.301	NEU
nsp12	1	260	K	-0.087	NEU	K	-0.415	NEU
nsp12	1	261	P	**0.735**	NEU	P	0.061	NEU
nsp12	1	262	Y	0.088	NEU	Y	-2.69	MAX
nsp12	1	263	R	0.353	NEU	I	1.529	MIN
nsp12	1	264	E	0.027	MIN	K	0.482	NEU
nsp12	1	265	W	**0.677**	MIN	W	0.972	MIN
nsp12	1	266	D	0.192	MAX	D	-0.708	NEU
nsp12	1	267	L	0.463	NEU	L	0.454	NEU
nsp12	1	268	V	0.103	NEU	L	0.583	NEU
nsp12	1	269	Q	0.423	NEU	K	0.286	NEU

nsp12	1	270	Y	**0.800**	MIN	Y	0.639	NEU
nsp12	1	271	D	**0.535**	MAX	D	-0.895	NEU
nsp12	1	272	F	0.136	MIN	F	1.001	MIN
nsp12	1	273	T	0.497	NEU	T	0.595	NEU
nsp12	1	274	D	**0.796**	NEU	E	0.981	MIN
nsp12	1	275	Y	0.368	MAX	E	-0.248	NEU
nsp12	1	276	K	**0.624**	MAX	R	-0.477	NEU
nsp12	1	277	L	**0.535**	MIN	L	0.72	NEU
nsp12	1	278	E	0.345	NEU	K	1.59	MIN
nsp12	1	279	L	**1.327**	MIN	L	1.246	MIN
nsp12	1	280	F	**0.955**	MIN	F	0.955	MIN
nsp12	1	281	N	0.353	NEU	D	-0.23	NEU
nsp12	1	282	K	0.341	NEU	R	-1.049	MAX
nsp12	1	283	Y	**1.164**	MAX	Y	-1.384	MAX
nsp12	1	284	F	**0.535**	MIN	F	0.598	NEU
nsp12	1	285	K	0.463	NEU	K	-0.623	NEU
nsp12	1	286	H	**0.873**	NEU	Y	0.778	NEU
nsp12	1	287	W	**1.327**	MAX	W	-1.458	MAX
nsp12	1	288	D	0.276	NEU	D	-0.014	NEU
nsp12	1	289	M	0.209	NEU	Q	-0.709	NEU
nsp12	1	290	T	0.463	NEU	T	-0.576	NEU
nsp12	1	291	Y	**1.164**	MAX	Y	-1.918	MAX
nsp12	1	292	H	**0.955**	MIN	H	0.704	NEU
nsp12	1	293	P	**0.955**	NEU	P	-0.017	NEU
nsp12	1	294	N	0.497	NEU	N	-0.907	NEU
nsp12	1	295	C	0.386	MIN	C	1.672	MIN
nsp12	1	296	V	**0.955**	MIN	V	1.407	MIN
nsp12	1	297	N	**0.577**	NEU	N	0.516	NEU
nsp12	1	298	C	**1.327**	MIN	C	1.609	MIN
nsp12	1	299	A	-0.043	NEU	L	0.133	NEU
nsp12	1	300	D	**1.050**	NEU	D	-0.114	NEU
nsp12	1	301	D	**1.327**	NEU	D	-0.428	NEU
nsp12	1	302	R	**0.873**	NEU	R	0.131	NEU
nsp12	1	303	C	**1.327**	MIN	C	2.307	MIN
nsp12	1	304	I	**1.164**	MIN	I	2.124	MIN
nsp12	1	305	L	**1.327**	MIN	L	1.195	MIN
nsp12	1	306	H	**1.327**	NEU	H	-0.595	NEU
nsp12	1	307	C	**1.327**	MIN	C	1.608	MIN
nsp12	1	308	A	**1.164**	MIN	A	0.652	NEU
nsp12	1	309	N	**1.327**	MAX	N	-1.149	MAX
nsp12	1	310	F	**1.327**	MIN	F	1.37	MIN
nsp12	1	311	N	0.368	MAX	N	-0.835	NEU
nsp12	1	312	I	**1.327**	MIN	V	0.943	MIN
nsp12	1	313	L	**1.327**	MIN	L	1.264	MIN
nsp12	1	314	F	**1.164**	MIN	F	1.191	MIN
nsp12	1	315	S	**0.873**	NEU	S	-0.424	NEU

nsp12	1	316	M	**0.677**	MIN	T	-0.335	NEU
nsp12	1	317	V	**0.535**	MIN	V	1.113	MIN
nsp12	1	318	L	0.463	MIN	F	0.094	NEU
nsp12	1	319	P	**1.003**	NEU	P	-0.328	NEU
nsp12	1	320	N	-0.080	NEU	P	0.827	MIN
nsp12	1	321	T	**1.327**	NEU	T	-0.138	NEU
nsp12	1	322	C	0.327	MIN	S	-0.319	NEU
nsp12	1	323	F	0.333	NEU	F	0.975	MIN
nsp12	1	324	G	**1.327**	NEU	G	-0.314	NEU
nsp12	1	325	P	**0.955**	NEU	P	-0.896	NEU
nsp12	1	326	L	**1.327**	MIN	L	0.838	MIN
nsp12	1	327	V	**1.327**	MIN	V	1.802	MIN
nsp12	1	328	R	**0.577**	NEU	R	-0.787	NEU
nsp12	1	329	K	0.408	MAX	K	-1.649	MAX
nsp12	1	330	I	**1.327**	MIN	I	2.061	MIN
nsp12	1	331	F	0.463	NEU	F	0.401	NEU
nsp12	1	332	V	**1.327**	MIN	V	1.496	MIN
nsp12	1	333	D	**0.577**	NEU	D	0.085	NEU
nsp12	1	334	G	**1.327**	NEU	G	-0.037	NEU
nsp12	1	335	V	**1.327**	MIN	V	0.684	NEU
nsp12	1	336	P	**0.873**	NEU	P	-0.668	NEU
nsp12	1	337	F	**1.164**	MIN	F	0.709	NEU
nsp12	1	338	V	**1.327**	MIN	V	1.657	MIN
nsp12	1	339	V	**1.327**	MIN	V	1.175	MIN
nsp12	1	340	S	**1.327**	NEU	S	-0.571	NEU
nsp12	1	341	I	0.386	MIN	T	-0.334	NEU
nsp12	1	342	G	**1.327**	NEU	G	-0.586	NEU
nsp12	1	343	Y	**0.955**	MIN	Y	0.714	NEU
nsp12	1	344	H	**1.327**	NEU	H	-0.142	NEU
nsp12	1	345	Y	0.333	MIN	F	1.141	MIN
nsp12	1	346	K	0.386	MAX	R	-0.567	NEU
nsp12	1	347	E	**1.327**	MAX	E	-1.416	MAX
nsp12	1	348	L	**1.327**	MIN	L	1.215	MIN
nsp12	1	349	G	**1.327**	NEU	G	-0.369	NEU
nsp12	1	350	V	**1.327**	MIN	V	1.177	MIN
nsp12	1	351	V	**1.327**	MIN	V	1.142	MIN
nsp12	1	352	M	0.463	NEU	H	-0.558	NEU
nsp12	1	353	N	**1.050**	NEU	N	-0.955	NEU
nsp12	1	354	M	0.199	MAX	Q	0.265	NEU
nsp12	1	355	D	**1.327**	MIN	D	1.764	MIN
nsp12	1	356	V	**0.624**	MIN	V	0.238	NEU
nsp12	1	357	D	**0.714**	NEU	N	-0.605	NEU
nsp12	1	358	T	-0.024	NEU	L	0.896	MIN
nsp12	1	359	H	0.328	MIN	H	0.762	NEU
nsp12	1	360	R	0.222	NEU	S	-0.469	NEU
nsp12	1	361	Y	0.114	MAX	S	0.585	NEU

nsp12	1	362	R	**1.327**	MIN	R	2.058	MIN
nsp12	1	363	L	**1.327**	MIN	L	1.137	MIN
nsp12	1	364	S	**1.164**	NEU	S	-0.179	NEU
nsp12	1	365	L	**0.735**	MIN	F	0.329	NEU
nsp12	1	366	K	**0.577**	NEU	K	-1.319	MAX
nsp12	1	367	E	**1.327**	NEU	E	-0.855	NEU
nsp12	1	368	L	**1.327**	MIN	L	1.268	MIN
nsp12	1	369	L	0.497	MIN	L	1.572	MIN
nsp12	1	370	V	**0.624**	MIN	V	1.381	MIN
nsp12	1	371	Y	**1.327**	NEU	Y	0.391	NEU
nsp12	1	372	A	0.497	NEU	A	0.269	NEU
nsp12	1	373	A	**0.624**	NEU	A	0.448	NEU
nsp12	1	374	D	**1.327**	MAX	D	-1.116	MAX
nsp12	1	375	P	**0.873**	NEU	P	-0.982	NEU
nsp12	1	376	A	0.497	MIN	A	0.739	NEU
nsp12	1	377	M	**1.327**	MIN	M	0.847	MIN
nsp12	1	378	H	**1.164**	NEU	H	-0.495	NEU
nsp12	1	379	V	**0.677**	MIN	A	0.514	NEU
nsp12	1	380	A	**1.164**	NEU	A	0.241	NEU
nsp12	1	381	S	**1.327**	NEU	S	-0.705	NEU
nsp12	1	382	A	**0.535**	NEU	G	-0.72	NEU
nsp12	1	383	N	**1.164**	NEU	N	-0.501	NEU
nsp12	1	384	A	0.423	NEU	L	1.288	MIN
nsp12	1	385	L	**1.164**	MIN	L	1.342	MIN
nsp12	1	386	L	0.239	NEU	L	-0.568	NEU
nsp12	1	387	D	-0.087	MAX	D	-1.079	MAX
nsp12	1	388	L	**0.955**	MIN	K	1.198	MIN
nsp12	1	389	R	0.382	MIN	R	1.562	MIN
nsp12	1	390	T	**1.327**	NEU	T	-0.479	NEU
nsp12	1	391	C	0.027	NEU	T	0.166	NEU
nsp12	1	392	C	**1.164**	MIN	C	1.554	MIN
nsp12	1	393	F	**0.677**	MIN	F	0.419	NEU
nsp12	1	394	S	**1.327**	NEU	S	-0.779	NEU
nsp12	1	395	V	**1.327**	MIN	V	1.673	MIN
nsp12	1	396	A	0.353	NEU	A	0.865	MIN
nsp12	1	397	A	0.434	NEU	A	0.529	NEU
nsp12	1	398	L	**1.050**	MIN	L	0.461	NEU
nsp12	1	399	T	**1.050**	NEU	T	0.283	NEU
nsp12	1	400	T	0.201	NEU	N	-0.127	NEU
nsp12	1	401	G	0.380	NEU	N	-0.519	NEU
nsp12	1	402	V	**0.800**	MIN	V	1.042	MIN
nsp12	1	403	T	-0.249	MIN	A	-1.015	MAX
nsp12	1	404	F	0.327	MIN	F	1.057	MIN
nsp12	1	405	Q	**0.735**	NEU	Q	-0.919	NEU
nsp12	1	406	T	0.184	NEU	T	0.17	NEU
nsp12	1	407	V	**0.800**	MIN	V	1.368	MIN

nsp12	1	408	K	0.434	MIN	K	0.611	NEU
nsp12	1	409	P	0.434	NEU	P	-0.684	NEU
nsp12	1	410	G	**1.327**	NEU	G	-0.359	NEU
nsp12	1	411	N	**0.535**	NEU	N	-0.966	NEU
nsp12	1	412	F	**1.327**	MIN	F	1.455	MIN
nsp12	1	413	N	**1.327**	NEU	N	-0.54	NEU
nsp12	1	414	Q	0.497	NEU	K	-1.03	MAX
nsp12	1	415	D	**1.327**	NEU	D	-0.589	NEU
nsp12	1	416	F	**1.327**	MIN	F	1.218	MIN
nsp12	1	417	Y	**1.327**	NEU	Y	-0.145	NEU
nsp12	1	418	D	0.368	MAX	D	-1.043	MAX
nsp12	1	419	F	**0.577**	NEU	F	-0.339	NEU
nsp12	1	420	A	0.368	MIN	A	0.366	NEU
nsp12	1	421	V	**1.164**	MIN	V	0.825	MIN
nsp12	1	422	S	**0.677**	NEU	S	-0.169	NEU
nsp12	1	423	K	0.318	MAX	K	-1.133	MAX
nsp12	1	424	G	**1.050**	NEU	G	-0.615	NEU
nsp12	1	425	F	**1.050**	MIN	F	0.656	NEU
nsp12	1	426	F	**1.164**	MIN	F	1.009	MIN
nsp12	1	427	K	**1.327**	MAX	K	-1.78	MAX
nsp12	1	428	E	**1.327**	NEU	E	-0.83	NEU
nsp12	1	429	G	**1.327**	NEU	G	-0.157	NEU
nsp12	1	430	S	**1.327**	NEU	S	-0.945	NEU
nsp12	1	431	S	0.497	MIN	S	0.87	MIN
nsp12	1	432	V	**1.327**	MIN	V	1.254	MIN
nsp12	1	433	D	0.368	NEU	E	-0.03	NEU
nsp12	1	434	L	**1.327**	MIN	L	0.757	NEU
nsp12	1	435	K	**0.778**	NEU	K	-0.174	NEU
nsp12	1	436	H	**1.327**	NEU	H	-0.517	NEU
nsp12	1	437	F	**1.327**	MIN	F	1.423	MIN
nsp12	1	438	F	**1.327**	MIN	F	1.3	MIN
nsp12	1	439	F	-0.166	NEU	F	0.274	NEU
nsp12	1	440	A	**0.800**	NEU	A	-0.91	NEU
nsp12	1	441	Q	**1.327**	NEU	Q	-0.72	NEU
nsp12	1	442	D	**0.624**	NEU	D	-0.966	NEU
nsp12	1	443	G	**1.164**	NEU	G	-0.715	NEU
nsp12	1	444	N	**1.164**	NEU	N	-0.172	NEU
nsp12	1	445	A	**1.327**	NEU	A	0.265	NEU
nsp12	1	446	A	**0.955**	MIN	A	0.781	MIN
nsp12	1	447	I	**0.873**	MIN	I	0.913	MIN
nsp12	1	448	T	**1.327**	NEU	S	-0.127	NEU
nsp12	1	449	D	**1.327**	NEU	D	-0.468	NEU
nsp12	1	450	Y	**1.164**	MIN	Y	0.81	MIN
nsp12	1	451	N	**1.164**	NEU	D	-0.401	NEU
nsp12	1	452	Y	**0.677**	MAX	Y	0.12	NEU
nsp12	1	453	Y	**1.050**	MIN	Y	1.471	MIN

nsp12	1	454	R	0.126	NEU	R	-0.423	NEU
nsp12	1	455	Y	0.184	NEU	Y	0.432	NEU
nsp12	1	456	N	**0.955**	NEU	N	-0.895	NEU
nsp12	1	457	L	**0.535**	NEU	L	0.24	NEU
nsp12	1	458	P	**1.327**	MAX	P	-1.196	MAX
nsp12	1	459	T	**1.327**	NEU	T	-0.183	NEU
nsp12	1	460	M	**1.327**	MIN	M	0.894	MIN
nsp12	1	461	C	**1.327**	MIN	C	1.441	MIN
nsp12	1	462	D	**0.735**	MAX	D	-0.979	NEU
nsp12	1	463	I	**1.327**	MIN	I	0.963	MIN
nsp12	1	464	K	0.497	MAX	R	0.207	NEU
nsp12	1	465	Q	**0.624**	NEU	Q	-0.849	NEU
nsp12	1	466	L	**0.624**	MIN	L	1.308	MIN
nsp12	1	467	L	**1.327**	MIN	L	0.992	MIN
nsp12	1	468	F	0.386	MIN	F	1.231	MIN
nsp12	1	469	V	**0.535**	MIN	V	1.285	MIN
nsp12	1	470	L	**1.327**	MIN	V	1.382	MIN
nsp12	1	471	E	0.386	NEU	E	-1.16	MAX
nsp12	1	472	V	**1.327**	MIN	V	1.239	MIN
nsp12	1	473	V	**1.327**	MIN	V	1.262	MIN
nsp12	1	474	D	0.463	MAX	D	-1.202	MAX
nsp12	1	475	K	**1.003**	NEU	K	-0.519	NEU
nsp12	1	476	Y	**1.164**	NEU	Y	0.044	NEU
nsp12	1	477	F	**0.735**	MIN	F	0.865	MIN
nsp12	1	478	E	0.368	MIN	D	0.351	NEU
nsp12	1	479	I	**1.327**	MIN	C	1.593	MIN
nsp12	1	480	Y	**1.327**	MIN	Y	0.779	NEU
nsp12	1	481	D	**0.873**	MIN	D	0.746	NEU
nsp12	1	482	G	**1.327**	NEU	G	-0.219	NEU
nsp12	1	483	G	**1.327**	NEU	G	-0.541	NEU
nsp12	1	484	C	**1.327**	MIN	C	1.725	MIN
nsp12	1	485	I	**1.327**	MIN	I	0.866	MIN
nsp12	1	486	N	-0.009	NEU	N	0.611	NEU
nsp12	1	487	A	**1.327**	NEU	A	-0.276	NEU
nsp12	1	488	S	**1.050**	NEU	N	-0.374	NEU
nsp12	1	489	Q	0.408	MAX	Q	-1.06	MAX
nsp12	1	490	V	**1.327**	MIN	V	1.378	MIN
nsp12	1	491	I	**1.164**	NEU	I	0.131	NEU
nsp12	1	492	V	**1.327**	MIN	V	1.192	MIN
nsp12	1	493	N	-0.051	NEU	N	-0.365	NEU
nsp12	1	494	N	**1.164**	MIN	N	0.886	MIN
nsp12	1	495	Y	0.341	MAX	L	1.533	MIN
nsp12	1	496	D	**1.327**	MIN	D	2.369	MIN
nsp12	1	497	K	0.497	MAX	K	-1.32	MAX
nsp12	1	498	S	**1.327**	NEU	S	-0.014	NEU
nsp12	1	499	A	**1.327**	NEU	A	-0.063	NEU

nsp12	1	500	G	**1.327**	NEU	G	-0.066	NEU
nsp12	1	501	Y	0.333	MIN	F	1.045	MIN
nsp12	1	502	P	**1.327**	NEU	P	-0.971	NEU
nsp12	1	503	F	**1.327**	MIN	F	1.288	MIN
nsp12	1	504	N	**1.327**	NEU	N	-0.588	NEU
nsp12	1	505	K	**0.873**	MAX	K	-1.644	MAX
nsp12	1	506	F	**0.677**	MIN	W	-2.508	MAX
nsp12	1	507	G	**1.327**	NEU	G	-0.357	NEU
nsp12	1	508	K	**1.327**	MAX	K	-1.633	MAX
nsp12	1	509	A	**1.327**	NEU	A	0.391	NEU
nsp12	1	510	R	**1.327**	MIN	R	2.132	MIN
nsp12	1	511	L	**1.327**	MIN	L	1.217	MIN
nsp12	1	512	Y	0.328	MAX	Y	-1.223	MAX
nsp12	1	513	Y	**1.327**	NEU	Y	-0.177	NEU
nsp12	1	514	E	**1.327**	NEU	D	-0.392	NEU
nsp12	1	515	S	**1.327**	NEU	S	-0.865	NEU
nsp12	1	516	L	0.161	MIN	M	0.248	NEU
nsp12	1	517	S	**0.955**	NEU	S	0.493	NEU
nsp12	1	518	Y	0.327	NEU	Y	-2.366	MAX
nsp12	1	519	E	**1.164**	NEU	E	-0.831	NEU
nsp12	1	520	E	**1.164**	MAX	D	-1.104	MAX
nsp12	1	521	Q	**1.327**	NEU	Q	-0.848	NEU
nsp12	1	522	D	**1.327**	MAX	D	-1.734	MAX
nsp12	1	523	E	0.353	NEU	A	0.378	NEU
nsp12	1	524	L	**1.164**	MIN	L	1.297	MIN
nsp12	1	525	F	0.141	NEU	F	0.652	NEU
nsp12	1	526	A	**0.955**	NEU	A	0.386	NEU
nsp12	1	527	Y	**0.604**	MAX	Y	-2.1	MAX
nsp12	1	528	T	**1.327**	NEU	T	-0.347	NEU
nsp12	1	529	K	0.333	NEU	K	-0.643	NEU
nsp12	1	530	R	**1.327**	MIN	R	1.013	MIN
nsp12	1	531	N	**0.577**	MAX	N	-1.061	MAX
nsp12	1	532	V	**1.327**	MIN	V	1.101	MIN
nsp12	1	533	L	**1.164**	MIN	I	0.534	NEU
nsp12	1	534	P	0.341	MAX	P	-0.895	NEU
nsp12	1	535	T	**1.327**	NEU	T	-0.161	NEU
nsp12	1	536	I	**1.164**	MIN	I	1.047	MIN
nsp12	1	537	T	**1.327**	NEU	T	-0.11	NEU
nsp12	1	538	Q	**1.327**	NEU	Q	-0.753	NEU
nsp12	1	539	M	**1.327**	MIN	M	0.788	MIN
nsp12	1	540	N	**0.873**	NEU	N	-0.926	NEU
nsp12	1	541	L	**1.327**	MIN	L	1.362	MIN
nsp12	1	542	K	**0.735**	NEU	K	-0.092	NEU
nsp12	1	543	Y	0.365	MAX	Y	-2.148	MAX
nsp12	1	544	A	**1.327**	NEU	A	-0.61	NEU
nsp12	1	545	I	**1.327**	MIN	I	0.994	MIN

nsp12	1	546	S	**1.327**	NEU	S	-0.736	NEU
nsp12	1	547	A	**0.955**	MAX	A	-1.242	MAX
nsp12	1	548	K	**1.327**	MAX	K	-2.122	MAX
nsp12	1	549	N	**1.327**	NEU	N	-0.254	NEU
nsp12	1	550	R	**0.955**	NEU	R	0.28	NEU
nsp12	1	551	A	**1.164**	NEU	A	-0.461	NEU
nsp12	1	552	R	**1.327**	NEU	R	-0.074	NEU
nsp12	1	553	T	**1.327**	NEU	T	-0.471	NEU
nsp12	1	554	V	0.341	NEU	V	0.279	NEU
nsp12	1	555	A	**0.955**	MIN	A	0.706	NEU
nsp12	1	556	G	**1.327**	NEU	G	-0.102	NEU
nsp12	1	557	V	**1.327**	MIN	V	1.348	MIN
nsp12	1	558	S	**1.327**	NEU	S	-0.5	NEU
nsp12	1	559	I	**1.327**	MIN	I	1.415	MIN
nsp12	1	560	L	**1.327**	MIN	C	1.543	MIN
nsp12	1	561	S	**1.327**	NEU	S	-0.617	NEU
nsp12	1	562	T	**1.327**	NEU	T	-0.302	NEU
nsp12	1	563	M	0.386	NEU	M	0.161	NEU
nsp12	1	564	T	**1.327**	NEU	T	-0.217	NEU
nsp12	1	565	N	**1.327**	NEU	N	-0.791	NEU
nsp12	1	566	R	**1.327**	NEU	R	-0.797	NEU
nsp12	1	567	Q	**0.800**	NEU	Q	-0.908	NEU
nsp12	1	568	F	-0.225	NEU	F	1.076	MIN
nsp12	1	569	H	**1.327**	NEU	H	0.015	NEU
nsp12	1	570	Q	0.386	NEU	Q	-0.904	NEU
nsp12	1	571	K	**1.327**	MAX	K	-1.983	MAX
nsp12	1	572	M	**1.327**	MIN	L	0.998	MIN
nsp12	1	573	L	**1.327**	MIN	L	1.561	MIN
nsp12	1	574	K	**1.164**	MAX	K	-1.028	MAX
nsp12	1	575	S	**1.327**	NEU	S	-0.461	NEU
nsp12	1	576	I	**1.327**	MIN	I	1.686	MIN
nsp12	1	577	A	**0.800**	MIN	A	0.777	NEU
nsp12	1	578	A	0.497	MAX	A	-1.149	MAX
nsp12	1	579	T	**1.327**	NEU	T	-0.484	NEU
nsp12	1	580	R	0.327	MIN	R	0.676	NEU
nsp12	1	581	G	**0.873**	NEU	G	-0.73	NEU
nsp12	1	582	A	0.341	NEU	A	0.403	NEU
nsp12	1	583	T	**1.327**	NEU	T	-0.557	NEU
nsp12	1	584	V	**1.327**	MIN	V	1.233	MIN
nsp12	1	585	V	**1.327**	MIN	V	1.237	MIN
nsp12	1	586	I	**1.327**	MIN	I	1.479	MIN
nsp12	1	587	G	**1.164**	NEU	G	-0.595	NEU
nsp12	1	588	T	**1.327**	NEU	T	-0.307	NEU
nsp12	1	589	T	**1.327**	NEU	S	0.275	NEU
nsp12	1	590	K	**1.327**	NEU	K	0.278	NEU
nsp12	1	591	F	**0.873**	MIN	F	0.815	MIN

nsp12	1	592	Y	**1.164**	MAX	Y	-1.528	MAX
nsp12	1	593	G	**1.327**	NEU	G	-0.306	NEU
nsp12	1	594	G	**1.327**	NEU	G	-0.387	NEU
nsp12	1	595	W	**1.327**	NEU	W	-0.636	NEU
nsp12	1	596	D	**0.577**	NEU	H	-0.243	NEU
nsp12	1	597	D	0.353	MIN	N	-0.214	NEU
nsp12	1	598	M	0.353	MIN	M	0.62	NEU
nsp12	1	599	L	**1.327**	MIN	L	1.486	MIN
nsp12	1	600	R	-0.105	NEU	K	-1.576	MAX
nsp12	1	601	T	**1.327**	NEU	T	-0.204	NEU
nsp12	1	602	L	**1.327**	MIN	V	1.128	MIN
nsp12	1	603	Y	0.333	MIN	Y	0.444	NEU
nsp12	1	604	K	0.353	MAX	S	0.019	NEU
nsp12	1	605	D	**0.535**	MIN	D	1.389	MIN
nsp12	1	606	V	**1.327**	MIN	V	1.101	MIN
nsp12	1	607	D	0.353	MIN	E	0.451	NEU
nsp12	1	608	N	0.382	NEU	N	-0.357	NEU
nsp12	1	609	P	**1.327**	NEU	P	-0.316	NEU
nsp12	1	610	H	0.063	NEU	H	-0.284	NEU
nsp12	1	611	L	**1.327**	MIN	L	1.076	MIN
nsp12	1	612	M	**1.327**	MIN	M	0.904	MIN
nsp12	1	613	G	**1.327**	NEU	G	-0.482	NEU
nsp12	1	614	W	0.442	NEU	W	-0.178	NEU
nsp12	1	615	D	0.027	NEU	D	-1.707	MAX
nsp12	1	616	Y	0.470	MAX	Y	0.864	MIN
nsp12	1	617	P	**0.873**	NEU	P	-0.203	NEU
nsp12	1	618	K	**0.735**	NEU	K	-0.371	NEU
nsp12	1	619	C	**1.327**	MIN	C	1.531	MIN
nsp12	1	620	D	**1.327**	NEU	D	-0.275	NEU
nsp12	1	621	R	**1.164**	NEU	R	-0.505	NEU
nsp12	1	622	A	**0.955**	NEU	A	0.461	NEU
nsp12	1	623	M	0.368	NEU	M	0.52	NEU
nsp12	1	624	P	0.333	NEU	P	-1.004	MAX
nsp12	1	625	N	**0.577**	MAX	N	-1.034	MAX
nsp12	1	626	M	**0.577**	MIN	M	0.554	NEU
nsp12	1	627	L	**1.327**	MIN	L	1.072	MIN
nsp12	1	628	R	**1.327**	NEU	R	-0.747	NEU
nsp12	1	629	I	**1.327**	MIN	I	1.939	MIN
nsp12	1	630	F	**1.327**	MIN	M	0.832	MIN
nsp12	1	631	A	**1.327**	NEU	A	0.388	NEU
nsp12	1	632	S	**0.577**	NEU	S	-0.925	NEU
nsp12	1	633	L	**1.327**	MIN	L	1.314	MIN
nsp12	1	634	V	**1.327**	MIN	V	1.217	MIN
nsp12	1	635	L	**1.164**	MIN	L	1.035	MIN
nsp12	1	636	A	**1.327**	NEU	A	0.274	NEU
nsp12	1	637	R	**1.050**	MIN	R	1.263	MIN

nsp12	1	638	K	0.463	NEU	K	-1.231	MAX
nsp12	1	639	H	**1.327**	NEU	H	0.115	NEU
nsp12	1	640	S	0.368	NEU	T	-0.66	NEU
nsp12	1	641	T	**0.677**	NEU	T	-0.079	NEU
nsp12	1	642	C	**1.327**	MIN	C	1.291	MIN
nsp12	1	643	C	**1.327**	MIN	C	1.348	MIN
nsp12	1	644	S	0.497	NEU	S	0.14	NEU
nsp12	1	645	Q	0.313	NEU	L	0.421	NEU
nsp12	1	646	S	**1.327**	NEU	S	-0.737	NEU
nsp12	1	647	D	0.105	NEU	H	-0.762	NEU
nsp12	1	648	R	**1.327**	NEU	R	-0.631	NEU
nsp12	1	649	F	**1.164**	MIN	F	1.241	MIN
nsp12	1	650	Y	-0.001	MIN	Y	1.016	MIN
nsp12	1	651	R	**0.577**	MIN	R	0.149	NEU
nsp12	1	652	L	**1.327**	MIN	L	1.068	MIN
nsp12	1	653	A	**1.327**	MIN	A	0.691	NEU
nsp12	1	654	N	0.386	NEU	N	-1.008	MAX
nsp12	1	655	E	**1.050**	MAX	E	-1.211	MAX
nsp12	1	656	C	**1.327**	MIN	C	1.879	MIN
nsp12	1	657	A	**1.327**	NEU	A	0.401	NEU
nsp12	1	658	Q	**1.327**	NEU	Q	-0.887	NEU
nsp12	1	659	V	0.368	MIN	V	0.672	NEU
nsp12	1	660	L	**1.050**	NEU	L	0.336	NEU
nsp12	1	661	S	**1.327**	NEU	S	-0.398	NEU
nsp12	1	662	E	**0.800**	NEU	E	-0.865	NEU
nsp12	1	663	I	0.368	MIN	M	0.704	NEU
nsp12	1	664	V	**1.327**	MIN	V	1.567	MIN
nsp12	1	665	L	**1.164**	MIN	M	1.006	MIN
nsp12	1	666	C	**1.164**	MIN	C	1.61	MIN
nsp12	1	667	G	**1.327**	NEU	G	-0.416	NEU
nsp12	1	668	G	**1.327**	NEU	G	-0.466	NEU
nsp12	1	669	G	0.386	NEU	S	-0.558	NEU
nsp12	1	670	Y	0.368	MIN	L	1.314	MIN
nsp12	1	671	Y	0.368	NEU	Y	0.569	NEU
nsp12	1	672	V	**1.327**	MIN	V	1.487	MIN
nsp12	1	673	K	**1.327**	MAX	K	-1.289	MAX
nsp12	1	674	P	0.386	MAX	P	-1.133	MAX
nsp12	1	675	G	**1.327**	NEU	G	-0.168	NEU
nsp12	1	676	G	**1.164**	NEU	G	-0.216	NEU
nsp12	1	677	T	**1.327**	NEU	T	-0.147	NEU
nsp12	1	678	S	**1.164**	NEU	S	-0.643	NEU
nsp12	1	679	S	**1.327**	NEU	S	-0.254	NEU
nsp12	1	680	G	**1.327**	NEU	G	-0.521	NEU
nsp12	1	681	D	**1.164**	MAX	D	-1.269	MAX
nsp12	1	682	A	**1.003**	NEU	A	0.08	NEU
nsp12	1	683	T	**1.327**	NEU	T	-0.18	NEU

nsp12	1	684	T	**1.164**	NEU	T	-0.179	NEU
nsp12	1	685	A	**0.890**	NEU	A	0.12	NEU
nsp12	1	686	Y	**0.800**	MIN	Y	0.685	NEU
nsp12	1	687	A	**1.164**	NEU	A	0.259	NEU
nsp12	1	688	N	**1.327**	NEU	N	-0.635	NEU
nsp12	1	689	S	**1.164**	NEU	S	-0.774	NEU
nsp12	1	690	V	**1.164**	MIN	V	1.596	MIN
nsp12	1	691	F	**1.327**	MIN	F	1.273	MIN
nsp12	1	692	N	**1.164**	NEU	N	-0.721	NEU
nsp12	1	693	I	**1.327**	MIN	I	1.243	MIN
nsp12	1	694	C	**1.327**	MIN	C	1.048	MIN
nsp12	1	695	Q	**1.045**	NEU	Q	-0.781	NEU
nsp12	1	696	A	**1.327**	NEU	A	0.39	NEU
nsp12	1	697	V	**0.577**	MIN	V	1.414	MIN
nsp12	1	698	T	**1.327**	NEU	T	-0.192	NEU
nsp12	1	699	A	**1.164**	NEU	A	0.397	NEU
nsp12	1	700	N	**0.577**	MAX	N	-1.117	MAX
nsp12	1	701	V	**1.164**	MIN	V	1.32	MIN
nsp12	1	702	C	0.368	NEU	N	-0.543	NEU
nsp12	1	703	A	**0.873**	NEU	A	0.379	NEU
nsp12	1	704	L	**1.327**	MIN	L	1.161	MIN
nsp12	1	705	M	**1.003**	MIN	L	1.045	MIN
nsp12	1	706	S	**1.327**	NEU	S	-0.58	NEU
nsp12	1	707	C	**0.535**	MIN	T	-0.294	NEU
nsp12	1	708	N	**1.164**	NEU	D	-0.498	NEU
nsp12	1	709	G	**0.955**	NEU	G	-0.632	NEU
nsp12	1	710	N	**1.050**	NEU	N	0.13	NEU
nsp12	1	711	K	0.328	NEU	K	0.74	NEU
nsp12	1	712	I	**1.327**	MIN	I	1.814	MIN
nsp12	1	713	E	-0.133	NEU	A	-0.812	NEU
nsp12	1	714	D	0.423	NEU	D	-1.059	MAX
nsp12	1	715	L	0.237	NEU	K	-1.923	MAX
nsp12	1	716	Y	0.031	MAX	Y	-1.575	MAX
nsp12	1	717	I	**1.327**	MIN	V	1.283	MIN
nsp12	1	718	R	0.333	NEU	R	0.129	NEU
nsp12	1	719	D	**1.327**	NEU	N	-0.517	NEU
nsp12	1	720	L	**0.955**	MIN	L	1.427	MIN
nsp12	1	721	Q	0.368	MAX	Q	-0.984	NEU
nsp12	1	722	K	0.027	NEU	H	0.223	NEU
nsp12	1	723	R	0.136	NEU	R	0.303	NEU
nsp12	1	724	L	**1.327**	MIN	L	1.305	MIN
nsp12	1	725	Y	0.199	NEU	Y	0.709	NEU
nsp12	1	726	S	-0.105	NEU	E	-1.322	MAX
nsp12	1	727	N	0.058	NEU	C	2.144	MIN
nsp12	1	728	V	**1.327**	MIN	L	1.153	MIN
nsp12	1	729	Y	0.333	MAX	Y	-1.242	MAX

nsp12	1	730	R	**0.624**	NEU	R	0.496	NEU
nsp12	1	731	S	**0.796**	NEU	N	-0.396	NEU
nsp12	1	732	D	0.386	MIN	R	1.896	MIN
nsp12	1	733	T	-0.152	MIN	D	1.393	MIN
nsp12	1	734	V	0.242	MIN	V	1.141	MIN
nsp12	1	735	D	0.408	NEU	D	-0.436	NEU
nsp12	1	736	P	0.304	NEU	T	-0.352	NEU
nsp12	1	737	K	-0.183	MIN	D	0.038	NEU
nsp12	1	738	F	0.333	NEU	F	1.081	MIN
nsp12	1	739	V	**1.327**	MIN	V	1.138	MIN
nsp12	1	740	D	0.249	NEU	N	-0.342	NEU
nsp12	1	741	E	**0.677**	MAX	E	-1.132	MAX
nsp12	1	742	Y	**0.624**	NEU	F	1.098	MIN
nsp12	1	743	Y	**0.677**	NEU	Y	-0.401	NEU
nsp12	1	744	A	**0.735**	NEU	A	-0.116	NEU
nsp12	1	745	F	0.386	MIN	Y	0.209	NEU
nsp12	1	746	L	**1.327**	MIN	L	1.343	MIN
nsp12	1	747	N	**1.164**	NEU	R	-0.301	NEU
nsp12	1	748	K	**1.327**	NEU	K	0.051	NEU
nsp12	1	749	H	**1.327**	NEU	H	-0.211	NEU
nsp12	1	750	F	**1.327**	MIN	F	1.222	MIN
nsp12	1	751	S	**1.327**	NEU	S	-0.593	NEU
nsp12	1	752	M	**0.735**	MIN	M	0.779	NEU
nsp12	1	753	M	**1.050**	MIN	M	0.899	MIN
nsp12	1	754	I	**1.327**	MIN	I	1.134	MIN
nsp12	1	755	L	**1.327**	MIN	L	1.025	MIN
nsp12	1	756	S	**1.327**	NEU	S	-0.511	NEU
nsp12	1	757	D	0.434	NEU	D	-1.352	MAX
nsp12	1	758	D	**1.327**	MAX	D	-1.357	MAX
nsp12	1	759	G	**1.327**	NEU	A	0.408	NEU
nsp12	1	760	V	**1.327**	MIN	V	1.076	MIN
nsp12	1	761	V	**1.327**	MIN	V	1.229	MIN
nsp12	1	762	C	**1.327**	MIN	C	1.558	MIN
nsp12	1	763	Y	**0.735**	NEU	F	1.248	MIN
nsp12	1	764	N	**0.873**	NEU	N	-0.997	NEU
nsp12	1	765	S	**1.327**	NEU	S	-0.203	NEU
nsp12	1	766	D	0.434	MIN	T	0.117	NEU
nsp12	1	767	Y	0.128	NEU	Y	-0.625	NEU
nsp12	1	768	A	**1.164**	NEU	A	-0.239	NEU
nsp12	1	769	S	-0.074	NEU	S	-0.023	NEU
nsp12	1	770	K	**1.327**	NEU	Q	-0.873	NEU
nsp12	1	771	G	**1.327**	NEU	G	-0.669	NEU
nsp12	1	772	Y	-0.087	MIN	L	1.123	MIN
nsp12	1	773	I	**1.327**	MIN	V	1.134	MIN
nsp12	1	774	A	**1.327**	NEU	A	-0.058	NEU
nsp12	1	775	N	**1.327**	NEU	S	-0.503	NEU

nsp12	1	776	I	**1.327**	MIN	I	1.027	MIN
nsp12	1	777	Q	0.100	MIN	K	-0.227	NEU
nsp12	1	778	N	0.469	NEU	N	-0.4	NEU
nsp12	1	779	F	**1.327**	MIN	F	1.004	MIN
nsp12	1	780	K	**1.327**	NEU	K	-0.88	NEU
nsp12	1	781	E	**0.577**	MAX	S	-0.459	NEU
nsp12	1	782	V	0.497	MIN	V	1.393	MIN
nsp12	1	783	L	**1.327**	MIN	L	1.018	MIN
nsp12	1	784	Y	**1.327**	MAX	Y	-1.842	MAX
nsp12	1	785	Y	**0.677**	MIN	Y	0.985	MIN
nsp12	1	786	Q	0.341	NEU	Q	-1.149	MAX
nsp12	1	787	N	**0.577**	NEU	N	-0.965	NEU
nsp12	1	788	N	0.434	NEU	N	-0.889	NEU
nsp12	1	789	V	**1.327**	MIN	V	1.496	MIN
nsp12	1	790	F	**0.624**	NEU	F	0.269	NEU
nsp12	1	791	M	**0.955**	NEU	M	0.037	NEU
nsp12	1	792	S	**1.327**	NEU	S	-0.98	NEU
nsp12	1	793	E	**1.164**	NEU	E	-0.086	NEU
nsp12	1	794	A	**1.327**	NEU	A	-0.115	NEU
nsp12	1	795	K	**0.735**	MAX	K	-1.257	MAX
nsp12	1	796	C	**1.327**	MIN	C	1.624	MIN
nsp12	1	797	W	**1.327**	MAX	W	-1.321	MAX
nsp12	1	798	V	**0.735**	MIN	T	-0.332	NEU
nsp12	1	799	E	**1.050**	MAX	E	-1.551	MAX
nsp12	1	800	P	**0.735**	NEU	T	0.307	NEU
nsp12	1	801	D	0.365	NEU	D	0.02	NEU
nsp12	1	802	I	**1.327**	MIN	L	1.178	MIN
nsp12	1	803	T	0.318	NEU	T	0.032	NEU
nsp12	1	804	K	0.497	MIN	K	1.49	MIN
nsp12	1	805	G	**1.327**	NEU	G	-0.384	NEU
nsp12	1	806	P	**1.327**	NEU	P	-0.719	NEU
nsp12	1	807	H	**0.800**	NEU	H	0.64	NEU
nsp12	1	808	E	**1.050**	NEU	E	-0.204	NEU
nsp12	1	809	F	0.497	MIN	F	0.769	NEU
nsp12	1	810	C	**1.327**	MIN	C	1.476	MIN
nsp12	1	811	S	**1.327**	NEU	S	0.149	NEU
nsp12	1	812	Q	**1.327**	NEU	Q	-0.403	NEU
nsp12	1	813	H	**1.164**	MIN	H	1.153	MIN
nsp12	1	814	T	**1.327**	NEU	T	-0.098	NEU
nsp12	1	815	M	0.463	MIN	M	0.665	NEU
nsp12	1	816	L	0.434	MIN	L	1.193	MIN
nsp12	1	817	V	**1.327**	MIN	V	1.441	MIN
nsp12	1	818	K	0.328	MIN	K	0.491	NEU
nsp12	1	819	M	0.161	NEU	Q	-0.852	NEU
nsp12	1	820	G	0.368	MIN	G	0.709	NEU
nsp12	1	821	G	0.353	NEU	D	0.753	NEU

nsp12	1	822	D	**0.800**	NEU	D	-0.84	NEU
nsp12	1	823	Y	0.276	NEU	Y	-1.617	MAX
nsp12	1	824	V	0.497	MIN	V	1.759	MIN
nsp12	1	825	Y	-0.249	MIN	Y	0.324	NEU
nsp12	1	826	L	**1.327**	MIN	L	1.364	MIN
nsp12	1	827	P	0.408	MAX	P	-0.73	NEU
nsp12	1	828	Y	**1.327**	MIN	Y	0.75	NEU
nsp12	1	829	P	**1.327**	MAX	P	-1.842	MAX
nsp12	1	830	D	**0.535**	NEU	D	0.214	NEU
nsp12	1	831	P	**1.327**	NEU	P	-0.875	NEU
nsp12	1	832	S	**1.327**	NEU	S	-0.576	NEU
nsp12	1	833	R	**0.955**	NEU	R	-0.565	NEU
nsp12	1	834	I	**1.327**	MIN	I	1.191	MIN
nsp12	1	835	L	**1.327**	MIN	L	1.169	MIN
nsp12	1	836	G	**1.327**	NEU	G	-0.355	NEU
nsp12	1	837	A	**0.735**	NEU	A	0.521	NEU
nsp12	1	838	G	**1.164**	NEU	G	-0.415	NEU
nsp12	1	839	C	**1.327**	MIN	C	1.256	MIN
nsp12	1	840	F	**1.327**	MIN	F	1.236	MIN
nsp12	1	841	V	**1.050**	MIN	V	1.857	MIN
nsp12	1	842	D	**0.624**	NEU	D	-0.321	NEU
nsp12	1	843	D	**0.873**	NEU	D	-0.705	NEU
nsp12	1	844	L	**1.164**	NEU	I	0.011	NEU
nsp12	1	845	L	**0.735**	NEU	V	0.185	NEU
nsp12	1	846	K	0.209	NEU	K	0.96	MIN
nsp12	1	847	T	**1.327**	NEU	T	-0.253	NEU
nsp12	1	848	D	**0.677**	MAX	D	-1.071	MAX
nsp12	1	849	G	**1.327**	NEU	G	-0.823	NEU
nsp12	1	850	T	0.328	MIN	T	0.33	NEU
nsp12	1	851	L	0.333	NEU	L	-0.283	NEU
nsp12	1	852	M	**1.327**	MIN	M	1.24	MIN
nsp12	1	853	I	**1.327**	MIN	I	1.48	MIN
nsp12	1	854	E	0.327	NEU	E	-0.803	NEU
nsp12	1	855	R	**1.327**	NEU	R	-0.764	NEU
nsp12	1	856	F	**1.164**	MIN	F	1.066	MIN
nsp12	1	857	V	**1.327**	MIN	V	1.374	MIN
nsp12	1	858	S	0.434	MAX	S	-1.17	MAX
nsp12	1	859	L	**1.327**	MIN	L	1.32	MIN
nsp12	1	860	A	**0.624**	NEU	A	0.514	NEU
nsp12	1	861	I	**1.327**	MIN	I	1.538	MIN
nsp12	1	862	D	0.408	NEU	D	-0.863	NEU
nsp12	1	863	A	0.463	NEU	A	0.524	NEU
nsp12	1	864	Y	**1.327**	NEU	Y	-0.719	NEU
nsp12	1	865	P	**1.327**	NEU	P	-0.357	NEU
nsp12	1	866	L	**1.327**	MIN	L	1.565	MIN
nsp12	1	867	T	0.386	NEU	T	-0.85	NEU

nsp12	1	868	K	**0.955**	MAX	K	-1.532	MAX
nsp12	1	869	H	**1.164**	NEU	H	0.415	NEU
nsp12	1	870	E	**0.677**	NEU	P	0.506	NEU
nsp12	1	871	N	**0.577**	NEU	N	-0.403	NEU
nsp12	1	872	P	0.341	MIN	Q	-0.219	NEU
nsp12	1	873	E	**0.955**	MAX	E	-1.694	MAX
nsp12	1	874	Y	0.463	NEU	Y	-1.258	MAX
nsp12	1	875	Q	0.386	NEU	A	0.063	NEU
nsp12	1	876	N	0.353	MAX	D	-1.069	MAX
nsp12	1	877	V	**1.327**	MIN	V	1.154	MIN
nsp12	1	878	F	**0.535**	MIN	F	0.807	MIN
nsp12	1	879	W	-0.219	NEU	H	0.167	NEU
nsp12	1	880	V	**1.327**	MIN	L	1.163	MIN
nsp12	1	881	Y	0.434	MIN	Y	0.654	NEU
nsp12	1	882	L	**1.327**	MIN	L	1.368	MIN
nsp12	1	883	Q	-0.133	MAX	Q	-1.121	MAX
nsp12	1	884	Y	**1.050**	MIN	Y	0.858	MIN
nsp12	1	885	I	**1.327**	MIN	I	1.255	MIN
nsp12	1	886	K	0.341	MAX	R	0.078	NEU
nsp12	1	887	K	0.222	NEU	K	1.203	MIN
nsp12	1	888	L	**1.327**	MIN	L	1.214	MIN
nsp12	1	889	Y	**1.327**	NEU	H	-0.242	NEU
nsp12	1	890	N	0.027	MIN	D	0.923	MIN
nsp12	1	891	D	**1.327**	NEU	E	-0.729	NEU
nsp12	1	892	L	**1.327**	MIN	L	1.18	MIN
nsp12	1	893	T	**1.327**	NEU	T	0.027	NEU
nsp12	1	894	G	**0.890**	NEU	G	-0.017	NEU
nsp12	1	895	H	-0.185	MIN	H	0.278	NEU
nsp12	1	896	M	**1.050**	MIN	M	0.958	MIN
nsp12	1	897	L	**0.873**	MIN	L	0.87	MIN
nsp12	1	898	D	**1.164**	NEU	D	-0.782	NEU
nsp12	1	899	S	**1.327**	NEU	M	0.136	NEU
nsp12	1	900	Y	**0.890**	NEU	Y	0.316	NEU
nsp12	1	901	S	**1.327**	NEU	S	-0.517	NEU
nsp12	1	902	V	**1.164**	MIN	V	1.725	MIN
nsp12	1	903	M	-0.144	NEU	M	-0.617	NEU
nsp12	1	904	L	**0.624**	MIN	L	0.599	NEU
nsp12	1	905	S	0.442	NEU	T	0.255	NEU
nsp12	1	906	T	0.313	NEU	N	1.588	MIN
nsp12	1	907	D	-0.196	MIN	D	1.364	MIN
nsp12	1	908	N	0.353	NEU	N	1.414	MIN
nsp12	1	909	G	0.434	NEU	T	0.529	NEU
nsp12	1	910	Q	0.030	NEU	S	0.39	NEU
nsp12	1	911	K	0.333	MIN	R	1.466	MIN
nsp12	1	912	F	0.239	MIN	Y	-0.765	NEU
nsp12	1	913	W	0.353	MIN	W	0.712	NEU

nsp12	1	914	E	0.368	NEU	E	-1.271	MAX
nsp12	1	915	E	0.423	NEU	P	0.269	NEU
nsp12	1	916	S	-0.156	NEU	E	-1.222	MAX
nsp12	1	917	F	**0.735**	NEU	F	0.616	NEU
nsp12	1	918	Y	**1.164**	NEU	Y	-0.527	NEU
nsp12	1	919	K	0.380	NEU	E	-0.777	NEU
nsp12	1	920	N	0.075	NEU	A	0.79	MIN
nsp12	1	921	M	0.341	MIN	M	1.055	MIN
nsp12	1	922	Y	**0.890**	MIN	Y	0.854	MIN
nsp12	1	923	L	-0.072	MIN	T	0.259	NEU
nsp12	1	924	R	-0.249	MIN	P	-1.208	MAX
nsp12	1	925	S	**0.624**	NEU	H	0.031	NEU
nsp12	1	926	A	**0.873**	NEU	T	0.478	NEU
nsp13	1	1	V	**1.292**	MIN	V	1.798	MIN
nsp13	1	2	G	**1.292**	NEU	G	-0.199	NEU
nsp13	1	3	S	**1.292**	NEU	A	0.137	NEU
nsp13	1	4	C	**1.292**	MIN	C	1.724	MIN
nsp13	1	5	V	**1.292**	MIN	V	1.217	MIN
nsp13	1	6	V	**1.292**	MIN	L	1.011	MIN
nsp13	1	7	C	**1.292**	MIN	C	1.565	MIN
nsp13	1	8	N	0.307	NEU	N	-0.772	NEU
nsp13	1	9	S	**1.292**	NEU	S	-0.264	NEU
nsp13	1	10	Q	**1.292**	NEU	Q	-0.473	NEU
nsp13	1	11	T	**1.292**	NEU	T	-0.583	NEU
nsp13	1	12	S	**1.292**	NEU	S	-0.571	NEU
nsp13	1	13	L	**1.292**	MIN	L	1.004	MIN
nsp13	1	14	R	**1.292**	NEU	R	-0.678	NEU
nsp13	1	15	C	**1.292**	MIN	C	1.353	MIN
nsp13	1	16	G	**1.292**	NEU	G	-0.411	NEU
nsp13	1	17	T	**1.292**	NEU	A	-0.163	NEU
nsp13	1	18	C	**1.292**	MIN	C	1.587	MIN
nsp13	1	19	I	**1.292**	MIN	I	0.857	MIN
nsp13	1	20	R	**1.292**	NEU	R	-0.836	NEU
nsp13	1	21	R	**1.292**	MAX	R	-1.389	MAX
nsp13	1	22	P	**1.292**	NEU	P	-0.835	NEU
nsp13	1	23	F	**1.292**	MIN	F	1.376	MIN
nsp13	1	24	L	**1.292**	MIN	L	1.059	MIN
nsp13	1	25	C	**1.292**	MIN	C	1.664	MIN
nsp13	1	26	C	**1.292**	MIN	C	1.66	MIN
nsp13	1	27	K	**1.292**	MAX	K	-1.38	MAX
nsp13	1	28	C	**1.292**	MIN	C	1.619	MIN
nsp13	1	29	C	**1.292**	MIN	C	2.043	MIN
nsp13	1	30	Y	**1.292**	MIN	Y	1.085	MIN
nsp13	1	31	D	**1.292**	NEU	D	-0.778	NEU
nsp13	1	32	H	**1.292**	NEU	H	0.041	NEU
nsp13	1	33	V	**1.292**	MIN	V	1.184	MIN

nsp13	1	34	I	**0.839**	MIN	I	1.708	MIN
nsp13	1	35	S	**0.590**	NEU	S	0.283	NEU
nsp13	1	36	T	**1.292**	NEU	T	-0.459	NEU
nsp13	1	37	T	0.307	NEU	S	-0.266	NEU
nsp13	1	38	H	**1.292**	NEU	H	0.001	NEU
nsp13	1	39	K	**1.292**	MAX	K	-1.105	MAX
nsp13	1	40	L	-0.264	NEU	L	0.615	NEU
nsp13	1	41	V	**1.292**	MIN	V	1.164	MIN
nsp13	1	42	L	**1.292**	MIN	L	0.687	NEU
nsp13	1	43	S	**1.292**	NEU	S	-0.555	NEU
nsp13	1	44	V	**0.839**	MIN	V	1.952	MIN
nsp13	1	45	S	**1.292**	NEU	N	0.178	NEU
nsp13	1	46	P	**1.292**	NEU	P	-0.958	NEU
nsp13	1	47	Y	0.374	MIN	Y	0.597	NEU
nsp13	1	48	V	**1.292**	MIN	V	0.722	NEU
nsp13	1	49	C	**1.292**	MIN	C	2.079	MIN
nsp13	1	50	N	**1.016**	NEU	N	-0.675	NEU
nsp13	1	51	A	**0.743**	NEU	A	-0.322	NEU
nsp13	1	52	P	**0.701**	MIN	P	0.886	MIN
nsp13	1	53	G	**1.292**	NEU	G	-0.268	NEU
nsp13	1	54	C	**1.292**	MIN	C	1.33	MIN
nsp13	1	55	D	**0.590**	NEU	D	-0.699	NEU
nsp13	1	56	V	**1.292**	MIN	V	1.854	MIN
nsp13	1	57	A	**1.292**	NEU	T	-0.702	NEU
nsp13	1	58	D	0.374	NEU	D	-0.942	NEU
nsp13	1	59	V	**1.292**	MIN	V	1.276	MIN
nsp13	1	60	T	**1.292**	NEU	T	-0.667	NEU
nsp13	1	61	Q	0.374	MAX	Q	-0.761	NEU
nsp13	1	62	L	**1.292**	MIN	L	1.311	MIN
nsp13	1	63	Y	**0.500**	NEU	Y	0.727	NEU
nsp13	1	64	L	**1.292**	MIN	L	1.061	MIN
nsp13	1	65	G	**1.292**	NEU	G	0.158	NEU
nsp13	1	66	G	**1.292**	NEU	G	-0.298	NEU
nsp13	1	67	M	**1.292**	NEU	M	-0.09	NEU
nsp13	1	68	S	**1.292**	NEU	S	0.313	NEU
nsp13	1	69	Y	**1.292**	MIN	Y	0.706	NEU
nsp13	1	70	Y	0.071	MIN	Y	0.438	NEU
nsp13	1	71	C	**1.292**	MIN	C	2.073	MIN
nsp13	1	72	K	0.091	MAX	K	-2.009	MAX
nsp13	1	73	D	**1.016**	NEU	S	0.127	NEU
nsp13	1	74	H	**0.839**	MIN	H	1.14	MIN
nsp13	1	75	K	0.294	NEU	K	-1.219	MAX
nsp13	1	76	P	**1.292**	NEU	P	-0.755	NEU
nsp13	1	77	P	0.294	MIN	P	1.62	MIN
nsp13	1	78	I	0.307	NEU	I	0.427	NEU
nsp13	1	79	S	**1.292**	NEU	S	-0.564	NEU

nsp13	1	80	F	**1.292**	MIN	F	1.028	MIN
nsp13	1	81	P	**1.292**	NEU	P	0.232	NEU
nsp13	1	82	L	**1.292**	MIN	L	1.069	MIN
nsp13	1	83	C	**1.292**	MIN	C	1.918	MIN
nsp13	1	84	A	**1.292**	NEU	A	-0.347	NEU
nsp13	1	85	N	0.294	MIN	N	1.134	MIN
nsp13	1	86	G	**1.292**	NEU	G	-0.593	NEU
nsp13	1	87	Q	0.294	NEU	Q	0.371	NEU
nsp13	1	88	V	**1.292**	MIN	V	1.233	MIN
nsp13	1	89	F	**1.292**	MIN	F	1.282	MIN
nsp13	1	90	G	**1.292**	NEU	G	-0.249	NEU
nsp13	1	91	L	**1.292**	MIN	L	0.724	NEU
nsp13	1	92	Y	**1.292**	NEU	Y	0.185	NEU
nsp13	1	93	K	**1.292**	MAX	K	-1.471	MAX
nsp13	1	94	N	**1.292**	NEU	N	-0.93	NEU
nsp13	1	95	M	**0.500**	NEU	T	0.06	NEU
nsp13	1	96	C	**1.292**	MIN	C	2.365	MIN
nsp13	1	97	T	0.374	NEU	V	0.651	NEU
nsp13	1	98	G	**1.292**	NEU	G	-0.106	NEU
nsp13	1	99	S	**1.292**	NEU	S	-0.36	NEU
nsp13	1	100	P	0.126	NEU	D	-1.69	MAX
nsp13	1	101	S	**0.500**	NEU	N	-0.978	NEU
nsp13	1	102	V	**0.701**	MIN	V	1.146	MIN
nsp13	1	103	T	**0.743**	NEU	T	-0.189	NEU
nsp13	1	104	D	0.334	MAX	D	-1.286	MAX
nsp13	1	105	F	**1.292**	MIN	F	1.433	MIN
nsp13	1	106	N	0.374	MAX	N	-1.007	MAX
nsp13	1	107	R	**1.292**	NEU	A	0.289	NEU
nsp13	1	108	L	**1.292**	MIN	I	1.215	MIN
nsp13	1	109	A	**1.292**	NEU	A	0.012	NEU
nsp13	1	110	T	**1.292**	NEU	T	-0.215	NEU
nsp13	1	111	C	**1.292**	MIN	C	1.04	MIN
nsp13	1	112	D	**1.292**	NEU	D	-0.625	NEU
nsp13	1	113	W	**1.016**	MAX	W	-1.118	MAX
nsp13	1	114	S	**1.292**	NEU	T	0.164	NEU
nsp13	1	115	N	0.374	NEU	N	-0.392	NEU
nsp13	1	116	S	0.177	NEU	A	0.904	MIN
nsp13	1	117	G	0.307	MAX	G	-0.679	NEU
nsp13	1	118	D	**1.016**	MAX	D	-1.134	MAX
nsp13	1	119	Y	0.429	MAX	Y	-0.568	NEU
nsp13	1	120	I	0.334	NEU	I	1.621	MIN
nsp13	1	121	L	**1.292**	MIN	L	1.196	MIN
nsp13	1	122	A	**1.292**	NEU	A	0.275	NEU
nsp13	1	123	N	**0.701**	NEU	N	-1.055	MAX
nsp13	1	124	T	0.374	NEU	T	-0.228	NEU
nsp13	1	125	T	0.374	NEU	C	2.089	MIN

nsp13	1	126	T	**1.292**	NEU	T	0.265	NEU
nsp13	1	127	E	**1.292**	NEU	E	-0.688	NEU
nsp13	1	128	R	**1.016**	NEU	R	-0.347	NEU
nsp13	1	129	L	**1.292**	MIN	L	1.336	MIN
nsp13	1	130	K	0.374	NEU	K	-1.077	MAX
nsp13	1	131	L	**1.292**	MIN	L	1.577	MIN
nsp13	1	132	F	**1.292**	MIN	F	1.181	MIN
nsp13	1	133	A	**1.292**	MIN	A	0.988	MIN
nsp13	1	134	A	**1.292**	MIN	A	0.844	MIN
nsp13	1	135	E	**0.839**	NEU	E	-0.983	NEU
nsp13	1	136	T	**1.292**	NEU	T	-0.319	NEU
nsp13	1	137	L	**1.292**	MIN	L	1.621	MIN
nsp13	1	138	R	0.294	NEU	K	0.34	NEU
nsp13	1	139	A	**1.292**	MIN	A	0.818	MIN
nsp13	1	140	T	**0.569**	NEU	T	-0.267	NEU
nsp13	1	141	E	**1.292**	NEU	E	-0.382	NEU
nsp13	1	142	E	**1.292**	NEU	E	-0.683	NEU
nsp13	1	143	A	**0.500**	NEU	T	-0.299	NEU
nsp13	1	144	S	**1.292**	NEU	F	0.192	NEU
nsp13	1	145	K	0.429	NEU	K	0.588	NEU
nsp13	1	146	Q	0.177	NEU	L	1.12	MIN
nsp13	1	147	S	**1.292**	NEU	S	-0.177	NEU
nsp13	1	148	Y	0.374	MIN	Y	0.907	MIN
nsp13	1	149	A	**1.016**	NEU	G	-0.442	NEU
nsp13	1	150	I	**0.500**	MIN	I	1.317	MIN
nsp13	1	151	A	**1.292**	NEU	A	-0.319	NEU
nsp13	1	152	T	**0.590**	NEU	T	-0.28	NEU
nsp13	1	153	V	**1.292**	MIN	V	1.135	MIN
nsp13	1	154	K	0.307	NEU	R	-0.256	NEU
nsp13	1	155	E	0.294	MAX	E	-1.08	MAX
nsp13	1	156	V	**1.292**	MIN	V	1.191	MIN
nsp13	1	157	L	**0.590**	MIN	L	1.204	MIN
nsp13	1	158	S	**1.292**	NEU	S	-0.343	NEU
nsp13	1	159	D	0.307	NEU	D	0.999	MIN
nsp13	1	160	R	0.374	NEU	R	-1.038	MAX
nsp13	1	161	E	0.307	MAX	E	-1.058	MAX
nsp13	1	162	L	**1.292**	MIN	L	1.215	MIN
nsp13	1	163	I	0.429	MIN	H	-0.508	NEU
nsp13	1	164	L	**1.292**	MIN	L	1.345	MIN
nsp13	1	165	S	-0.149	MIN	S	-0.849	NEU
nsp13	1	166	W	**0.701**	NEU	W	-0.944	NEU
nsp13	1	167	E	**0.701**	NEU	E	0.387	NEU
nsp13	1	168	V	0.294	NEU	V	0.875	MIN
nsp13	1	169	G	**1.292**	NEU	G	-0.122	NEU
nsp13	1	170	K	**1.292**	NEU	K	-0.278	NEU
nsp13	1	171	P	**1.016**	NEU	P	0.155	NEU

nsp13	1	172	R	**1.292**	NEU	R	0.269	NEU
nsp13	1	173	P	**1.292**	NEU	P	-0.591	NEU
nsp13	1	174	P	**0.500**	NEU	P	-0.174	NEU
nsp13	1	175	L	**1.292**	MIN	L	1.04	MIN
nsp13	1	176	N	**1.292**	NEU	N	-0.249	NEU
nsp13	1	177	R	**1.292**	NEU	R	-0.483	NEU
nsp13	1	178	N	**1.292**	MIN	N	1.355	MIN
nsp13	1	179	Y	**1.292**	NEU	Y	-0.186	NEU
nsp13	1	180	V	**0.839**	MIN	V	1.319	MIN
nsp13	1	181	F	**1.292**	MIN	F	0.982	MIN
nsp13	1	182	T	**1.292**	NEU	T	-0.137	NEU
nsp13	1	183	G	**1.292**	NEU	G	-0.176	NEU
nsp13	1	184	Y	**1.016**	MIN	Y	0.654	NEU
nsp13	1	185	H	0.334	NEU	R	0.536	NEU
nsp13	1	186	I	**0.839**	MIN	V	1.736	MIN
nsp13	1	187	T	**0.500**	MIN	T	0.513	NEU
nsp13	1	188	K	**1.016**	NEU	K	-0.232	NEU
nsp13	1	189	N	**1.292**	NEU	N	-0.329	NEU
nsp13	1	190	S	**1.292**	NEU	S	-0.762	NEU
nsp13	1	191	K	**1.292**	NEU	K	-0.373	NEU
nsp13	1	192	V	**0.839**	MIN	V	1.655	MIN
nsp13	1	193	Q	**1.292**	NEU	Q	-0.466	NEU
nsp13	1	194	L	**1.292**	MIN	I	1.574	MIN
nsp13	1	195	G	**1.292**	NEU	G	-0.019	NEU
nsp13	1	196	E	**0.590**	NEU	E	-0.942	NEU
nsp13	1	197	Y	0.307	MIN	Y	0.639	NEU
nsp13	1	198	I	0.374	MIN	T	-0.448	NEU
nsp13	1	199	F	**0.590**	MIN	F	0.635	NEU
nsp13	1	200	E	**1.292**	NEU	E	-0.783	NEU
nsp13	1	201	K	0.429	NEU	K	-0.66	NEU
nsp13	1	202	G	0.307	NEU	G	-0.676	NEU
nsp13	1	203	D	**1.292**	MIN	D	1.023	MIN
nsp13	1	204	Y	**0.590**	MAX	Y	-2.634	MAX
nsp13	1	205	G	**1.016**	NEU	G	-0.757	NEU
nsp13	1	206	D	**1.016**	NEU	D	-0.29	NEU
nsp13	1	207	A	0.294	MIN	A	0.254	NEU
nsp13	1	208	V	**1.292**	MIN	V	1.015	MIN
nsp13	1	209	S	0.334	NEU	V	0.393	NEU
nsp13	1	210	Y	0.374	NEU	Y	0.018	NEU
nsp13	1	211	R	**0.500**	MIN	R	1.145	MIN
nsp13	1	212	S	**1.292**	NEU	G	-0.301	NEU
nsp13	1	213	S	**1.292**	NEU	T	-0.252	NEU
nsp13	1	214	T	**1.292**	NEU	T	-0.185	NEU
nsp13	1	215	T	0.429	NEU	T	0.79	MIN
nsp13	1	216	Y	0.307	MAX	Y	-2.253	MAX
nsp13	1	217	K	**1.292**	MIN	K	1.313	MIN

nsp13	1	218	L	**1.292**	MIN	L	0.988	MIN
nsp13	1	219	N	**0.701**	NEU	N	-0.347	NEU
nsp13	1	220	V	**1.292**	MIN	V	1.677	MIN
nsp13	1	221	G	**1.292**	NEU	G	-0.566	NEU
nsp13	1	222	D	0.374	MAX	D	-0.909	NEU
nsp13	1	223	Y	**1.292**	MIN	Y	0.787	MIN
nsp13	1	224	F	**1.292**	MIN	F	1.019	MIN
nsp13	1	225	V	**1.292**	MIN	V	1.351	MIN
nsp13	1	226	L	**1.292**	MIN	L	1.032	MIN
nsp13	1	227	T	**1.292**	NEU	T	-0.361	NEU
nsp13	1	228	S	**1.292**	NEU	S	-0.796	NEU
nsp13	1	229	H	**1.292**	NEU	H	0.379	NEU
nsp13	1	230	S	**1.016**	NEU	T	0.267	NEU
nsp13	1	231	V	**1.292**	MIN	V	0.852	MIN
nsp13	1	232	A	0.334	NEU	M	-0.542	NEU
nsp13	1	233	P	**0.839**	NEU	P	-0.693	NEU
nsp13	1	234	L	**1.292**	MIN	L	1.287	MIN
nsp13	1	235	S	**0.500**	NEU	S	0.236	NEU
nsp13	1	236	A	**1.292**	NEU	A	-0.113	NEU
nsp13	1	237	P	**1.292**	MAX	P	-1.542	MAX
nsp13	1	238	T	**1.292**	NEU	T	-0.518	NEU
nsp13	1	239	L	0.429	MIN	L	0.571	NEU
nsp13	1	240	V	**1.292**	MIN	V	1.235	MIN
nsp13	1	241	P	**1.016**	NEU	P	0.275	NEU
nsp13	1	242	Q	**0.701**	NEU	Q	-0.252	NEU
nsp13	1	243	E	0.374	MAX	E	-0.345	NEU
nsp13	1	244	R	0.329	NEU	H	0.043	NEU
nsp13	1	245	Y	0.334	MAX	Y	-1.827	MAX
nsp13	1	246	V	0.307	NEU	V	0.837	MIN
nsp13	1	247	K	0.307	NEU	R	0.048	NEU
nsp13	1	248	I	**1.292**	MIN	I	1.369	MIN
nsp13	1	249	T	0.374	NEU	T	0.438	NEU
nsp13	1	250	G	**1.292**	NEU	G	0.158	NEU
nsp13	1	251	L	**1.292**	MIN	L	1.229	MIN
nsp13	1	252	Y	**0.500**	MAX	Y	-1.754	MAX
nsp13	1	253	P	**1.292**	NEU	P	-0.556	NEU
nsp13	1	254	T	**1.016**	NEU	T	-0.582	NEU
nsp13	1	255	L	0.307	NEU	L	-0.641	NEU
nsp13	1	256	N	**1.292**	NEU	N	-0.338	NEU
nsp13	1	257	V	**1.292**	MIN	I	1.672	MIN
nsp13	1	258	P	0.429	NEU	S	-0.479	NEU
nsp13	1	259	E	0.307	MAX	D	-0.325	NEU
nsp13	1	260	E	**1.016**	NEU	E	-0.983	NEU
nsp13	1	261	F	**0.701**	MIN	F	0.645	NEU
nsp13	1	262	A	**0.500**	NEU	S	-0.676	NEU
nsp13	1	263	S	**1.292**	NEU	S	-0.425	NEU

nsp13	1	264	N	**0.500**	NEU	N	-0.986	NEU
nsp13	1	265	V	**1.292**	MIN	V	0.921	MIN
nsp13	1	266	A	-0.264	NEU	A	-0.355	NEU
nsp13	1	267	N	**1.016**	NEU	N	-0.476	NEU
nsp13	1	268	Y	0.307	NEU	Y	-0.217	NEU
nsp13	1	269	Q	0.307	NEU	Q	-0.999	NEU
nsp13	1	270	K	**0.500**	MAX	K	-1.359	MAX
nsp13	1	271	V	**0.500**	MIN	V	1.51	MIN
nsp13	1	272	G	**1.292**	NEU	G	-0.526	NEU
nsp13	1	273	M	0.429	MIN	M	0.964	MIN
nsp13	1	274	S	0.126	NEU	Q	-1.546	MAX
nsp13	1	275	K	0.294	NEU	K	-0.88	NEU
nsp13	1	276	Y	**0.590**	NEU	Y	0.164	NEU
nsp13	1	277	V	0.334	NEU	S	-0.516	NEU
nsp13	1	278	T	**1.292**	NEU	T	-0.494	NEU
nsp13	1	279	V	**1.292**	MIN	L	1.073	MIN
nsp13	1	280	Q	**0.500**	NEU	Q	-1.001	MAX
nsp13	1	281	G	**1.292**	NEU	G	-0.051	NEU
nsp13	1	282	P	**0.590**	MAX	P	-1.017	MAX
nsp13	1	283	P	**1.292**	NEU	P	-0.33	NEU
nsp13	1	284	G	**1.292**	NEU	G	0.245	NEU
nsp13	1	285	T	**1.292**	NEU	T	-0.251	NEU
nsp13	1	286	G	**1.292**	NEU	G	0.063	NEU
nsp13	1	287	K	**1.292**	MAX	K	-1.325	MAX
nsp13	1	288	S	**1.292**	NEU	S	-0.706	NEU
nsp13	1	289	H	0.429	NEU	H	-0.305	NEU
nsp13	1	290	F	**1.292**	MIN	F	1.308	MIN
nsp13	1	291	A	**0.590**	MIN	A	0.698	NEU
nsp13	1	292	I	**1.292**	MIN	I	1.462	MIN
nsp13	1	293	G	**1.292**	NEU	G	-0.379	NEU
nsp13	1	294	L	**1.292**	MIN	L	1.311	MIN
nsp13	1	295	A	**1.292**	NEU	A	0.508	NEU
nsp13	1	296	L	**1.292**	MIN	L	1.34	MIN
nsp13	1	297	Y	**1.292**	MIN	Y	0.948	MIN
nsp13	1	298	Y	**1.016**	NEU	Y	0.352	NEU
nsp13	1	299	P	**1.016**	NEU	P	-0.818	NEU
nsp13	1	300	S	**1.292**	NEU	S	-0.793	NEU
nsp13	1	301	A	**1.292**	MIN	A	0.798	MIN
nsp13	1	302	R	**0.500**	NEU	R	-0.275	NEU
nsp13	1	303	I	**1.292**	MIN	I	1.438	MIN
nsp13	1	304	V	**1.292**	MIN	V	1.26	MIN
nsp13	1	305	Y	0.307	NEU	Y	0.609	NEU
nsp13	1	306	T	**1.292**	NEU	T	-0.49	NEU
nsp13	1	307	A	**1.292**	NEU	A	0.093	NEU
nsp13	1	308	C	**1.292**	MIN	C	1.363	MIN
nsp13	1	309	S	**1.292**	NEU	S	-0.832	NEU

nsp13	1	310	H	**1.292**	NEU	H	-0.065	NEU
nsp13	1	311	A	**1.292**	NEU	A	-0.053	NEU
nsp13	1	312	A	**1.292**	NEU	A	-0.153	NEU
nsp13	1	313	V	**1.292**	MIN	V	1.09	MIN
nsp13	1	314	D	**1.292**	MAX	D	-1.358	MAX
nsp13	1	315	A	**1.292**	NEU	A	0.196	NEU
nsp13	1	316	L	**1.292**	MIN	L	1.187	MIN
nsp13	1	317	C	**1.292**	MIN	C	1.14	MIN
nsp13	1	318	E	**0.701**	NEU	E	-0.829	NEU
nsp13	1	319	K	**1.292**	MAX	K	-1.653	MAX
nsp13	1	320	A	0.429	NEU	A	0.569	NEU
nsp13	1	321	F	**0.701**	MIN	L	0.936	MIN
nsp13	1	322	K	**0.500**	NEU	K	-0.065	NEU
nsp13	1	323	Y	**0.590**	MAX	Y	-1.658	MAX
nsp13	1	324	L	**1.292**	MIN	L	1.521	MIN
nsp13	1	325	P	0.374	NEU	P	-1.356	MAX
nsp13	1	326	I	**1.292**	MIN	I	1.047	MIN
nsp13	1	327	A	0.429	NEU	D	0.717	NEU
nsp13	1	328	K	0.294	NEU	K	-0.748	NEU
nsp13	1	329	C	**1.292**	MIN	C	1.784	MIN
nsp13	1	330	S	**1.292**	NEU	S	-0.735	NEU
nsp13	1	331	R	**1.292**	NEU	R	-0.701	NEU
nsp13	1	332	I	**1.292**	MIN	I	1.182	MIN
nsp13	1	333	I	**1.292**	MIN	I	1.266	MIN
nsp13	1	334	P	0.334	MAX	P	-1.733	MAX
nsp13	1	335	A	0.374	MAX	A	-0.883	NEU
nsp13	1	336	K	-0.118	NEU	R	-0.309	NEU
nsp13	1	337	A	**0.701**	MIN	A	0.66	NEU
nsp13	1	338	R	**1.292**	MIN	R	1.31	MIN
nsp13	1	339	V	**1.292**	MIN	V	1.349	MIN
nsp13	1	340	E	**1.292**	NEU	E	-0.039	NEU
nsp13	1	341	C	**1.292**	MIN	C	1.175	MIN
nsp13	1	342	F	0.307	MIN	F	1.236	MIN
nsp13	1	343	D	**0.701**	MAX	D	-1.61	MAX
nsp13	1	344	K	**1.292**	NEU	K	-0.964	NEU
nsp13	1	345	F	**1.016**	MIN	F	0.612	NEU
nsp13	1	346	K	0.429	NEU	K	0.631	NEU
nsp13	1	347	V	**1.016**	MIN	V	0.615	NEU
nsp13	1	348	N	**1.016**	MAX	N	-1.085	MAX
nsp13	1	349	E	0.374	NEU	S	-0.714	NEU
nsp13	1	350	T	**0.500**	NEU	T	-0.717	NEU
nsp13	1	351	N	**0.701**	NEU	L	-0.236	NEU
nsp13	1	352	S	**1.016**	NEU	E	-0.62	NEU
nsp13	1	353	Q	**1.292**	NEU	Q	-0.117	NEU
nsp13	1	354	Y	0.429	NEU	Y	0.602	NEU
nsp13	1	355	V	**1.292**	MIN	V	1.209	MIN

nsp13	1	356	F	**1.292**	MIN	F	1.47	MIN
nsp13	1	357	S	0.429	NEU	C	1.184	MIN
nsp13	1	358	T	**1.292**	NEU	T	-0.251	NEU
nsp13	1	359	I	**1.292**	MIN	V	1.358	MIN
nsp13	1	360	N	0.429	NEU	N	-1.218	MAX
nsp13	1	361	A	0.429	NEU	A	0.558	NEU
nsp13	1	362	L	**1.292**	MIN	L	1.24	MIN
nsp13	1	363	P	**0.701**	MAX	P	-0.964	NEU
nsp13	1	364	E	0.374	NEU	E	-0.224	NEU
nsp13	1	365	T	**1.292**	NEU	T	-0.31	NEU
nsp13	1	366	T	**0.839**	NEU	T	-0.54	NEU
nsp13	1	367	A	**0.701**	MIN	A	0.894	MIN
nsp13	1	368	D	**1.292**	NEU	D	-0.662	NEU
nsp13	1	369	I	**1.292**	MIN	I	1.18	MIN
nsp13	1	370	L	**1.292**	MIN	V	1.251	MIN
nsp13	1	371	V	**1.292**	MIN	V	1.288	MIN
nsp13	1	372	V	**1.292**	MIN	F	1.306	MIN
nsp13	1	373	D	**1.292**	MAX	D	-1.309	MAX
nsp13	1	374	E	**1.292**	MAX	E	-1.246	MAX
nsp13	1	375	V	**1.292**	MIN	I	1.37	MIN
nsp13	1	376	S	**1.292**	NEU	S	-0.304	NEU
nsp13	1	377	M	**1.292**	NEU	M	0.159	NEU
nsp13	1	378	C	**1.292**	MIN	A	0.657	NEU
nsp13	1	379	T	**1.292**	NEU	T	-0.339	NEU
nsp13	1	380	N	**1.292**	NEU	N	-0.83	NEU
nsp13	1	381	Y	**0.500**	MIN	Y	0.649	NEU
nsp13	1	382	D	**1.292**	MAX	D	-1.378	MAX
nsp13	1	383	L	**1.292**	MIN	L	1.233	MIN
nsp13	1	384	S	**1.292**	NEU	S	-0.711	NEU
nsp13	1	385	I	**0.701**	MIN	V	1.369	MIN
nsp13	1	386	I	**1.292**	MIN	V	1.317	MIN
nsp13	1	387	N	**1.292**	NEU	N	-0.952	NEU
nsp13	1	388	A	**1.292**	NEU	A	-0.221	NEU
nsp13	1	389	R	**1.292**	NEU	R	-0.097	NEU
nsp13	1	390	V	**1.292**	MIN	L	1.214	MIN
nsp13	1	391	R	**1.292**	MIN	R	1.538	MIN
nsp13	1	392	A	**1.292**	NEU	A	0.512	NEU
nsp13	1	393	K	**0.500**	NEU	K	-0.761	NEU
nsp13	1	394	H	**1.292**	NEU	H	-0.413	NEU
nsp13	1	395	I	0.429	MIN	Y	-0.325	NEU
nsp13	1	396	V	**1.292**	MIN	V	1.166	MIN
nsp13	1	397	Y	**0.839**	MIN	Y	0.698	NEU
nsp13	1	398	V	**1.292**	MIN	I	1.373	MIN
nsp13	1	399	G	**1.292**	NEU	G	-0.114	NEU
nsp13	1	400	D	**1.292**	MAX	D	-1.233	MAX
nsp13	1	401	P	0.334	NEU	P	-1.215	MAX

nsp13	1	402	A	**1.292**	NEU	A	0.451	NEU
nsp13	1	403	Q	**1.292**	NEU	Q	-0.486	NEU
nsp13	1	404	L	**1.292**	MIN	L	0.606	NEU
nsp13	1	405	P	**1.292**	MAX	P	-1.297	MAX
nsp13	1	406	A	**1.292**	NEU	A	0.335	NEU
nsp13	1	407	P	**1.292**	NEU	P	-0.743	NEU
nsp13	1	408	R	**1.292**	NEU	R	-0.224	NEU
nsp13	1	409	T	**1.292**	MIN	T	0.857	MIN
nsp13	1	410	L	**0.500**	MIN	L	0.966	MIN
nsp13	1	411	L	**0.701**	MIN	L	0.896	MIN
nsp13	1	412	T	**1.292**	NEU	T	0.257	NEU
nsp13	1	413	K	0.334	NEU	K	-0.454	NEU
nsp13	1	414	G	0.307	MAX	G	-1.205	MAX
nsp13	1	415	T	**0.839**	NEU	T	0.34	NEU
nsp13	1	416	L	**0.839**	MIN	L	1.144	MIN
nsp13	1	417	E	**0.500**	MAX	E	-1.205	MAX
nsp13	1	418	P	0.294	NEU	P	-1.123	MAX
nsp13	1	419	E	**1.292**	MAX	E	-1.574	MAX
nsp13	1	420	N	-0.283	NEU	Y	0.828	MIN
nsp13	1	421	F	**1.292**	MIN	F	0.968	MIN
nsp13	1	422	N	0.307	NEU	N	-0.971	NEU
nsp13	1	423	S	**1.292**	NEU	S	-0.513	NEU
nsp13	1	424	V	**1.292**	MIN	V	1.147	MIN
nsp13	1	425	C	0.307	MIN	C	2.583	MIN
nsp13	1	426	R	0.294	MAX	R	-0.99	NEU
nsp13	1	427	L	**1.016**	MIN	L	1.074	MIN
nsp13	1	428	M	0.429	NEU	M	0.728	NEU
nsp13	1	429	C	0.374	MIN	K	-1.891	MAX
nsp13	1	430	N	-0.064	NEU	T	0.033	NEU
nsp13	1	431	L	**1.292**	MIN	I	1.53	MIN
nsp13	1	432	G	0.374	NEU	G	0.576	NEU
nsp13	1	433	P	0.374	NEU	P	-0.883	NEU
nsp13	1	434	D	**0.839**	NEU	D	-0.734	NEU
nsp13	1	435	I	**0.500**	MIN	M	0.383	NEU
nsp13	1	436	F	**1.292**	MIN	F	0.91	MIN
nsp13	1	437	L	**1.292**	MIN	L	1.117	MIN
nsp13	1	438	G	**0.500**	NEU	G	-0.274	NEU
nsp13	1	439	T	0.334	NEU	T	-0.188	NEU
nsp13	1	440	C	**1.292**	MIN	C	1.786	MIN
nsp13	1	441	Y	-0.253	MIN	R	-0.058	NEU
nsp13	1	442	R	0.307	NEU	R	-0.965	NEU
nsp13	1	443	C	**1.292**	MIN	C	1.453	MIN
nsp13	1	444	P	**1.292**	MAX	P	-1.336	MAX
nsp13	1	445	K	0.334	MAX	A	0.496	NEU
nsp13	1	446	E	**1.292**	MAX	E	-1.202	MAX
nsp13	1	447	I	**1.292**	MIN	I	1.063	MIN

nsp13	1	448	V	**1.292**	MIN	V	1.124	MIN
nsp13	1	449	D	**0.839**	NEU	D	-1.026	MAX
nsp13	1	450	T	**1.292**	NEU	T	-0.284	NEU
nsp13	1	451	V	**1.292**	MIN	V	1.353	MIN
nsp13	1	452	S	**1.292**	NEU	S	-0.368	NEU
nsp13	1	453	A	**1.292**	NEU	A	-0.253	NEU
nsp13	1	454	L	**1.292**	MIN	L	1.033	MIN
nsp13	1	455	V	**1.292**	MIN	V	1.135	MIN
nsp13	1	456	Y	**1.016**	NEU	Y	0.443	NEU
nsp13	1	457	D	**1.292**	NEU	D	-0.445	NEU
nsp13	1	458	N	**1.292**	NEU	N	-0.624	NEU
nsp13	1	459	K	0.307	NEU	K	-0.467	NEU
nsp13	1	460	L	**1.292**	MIN	L	0.977	MIN
nsp13	1	461	K	0.334	NEU	K	0.043	NEU
nsp13	1	462	A	**1.292**	NEU	A	-0.505	NEU
nsp13	1	463	K	0.334	MAX	H	0.47	NEU
nsp13	1	464	K	**1.292**	MAX	K	-1.426	MAX
nsp13	1	465	E	-0.227	MIN	D	-1.087	MAX
nsp13	1	466	K	-0.020	MAX	K	-1.154	MAX
nsp13	1	467	S	**1.292**	NEU	S	-0.492	NEU
nsp13	1	468	G	**1.292**	NEU	A	-0.862	NEU
nsp13	1	469	Q	**1.292**	NEU	Q	-0.775	NEU
nsp13	1	470	C	**1.292**	MIN	C	1.259	MIN
nsp13	1	471	F	**0.701**	MIN	F	1.327	MIN
nsp13	1	472	K	**1.292**	MAX	K	-1.244	MAX
nsp13	1	473	I	**1.292**	MIN	M	0.899	MIN
nsp13	1	474	L	**0.500**	MIN	F	1.025	MIN
nsp13	1	475	Y	**1.292**	MIN	Y	0.874	MIN
nsp13	1	476	K	**1.016**	NEU	K	-0.101	NEU
nsp13	1	477	G	**1.016**	NEU	G	-0.459	NEU
nsp13	1	478	S	0.429	NEU	V	0.044	NEU
nsp13	1	479	V	**0.500**	MIN	I	0.925	MIN
nsp13	1	480	T	**1.292**	NEU	T	-0.223	NEU
nsp13	1	481	H	0.429	NEU	H	-0.835	NEU
nsp13	1	482	D	0.429	NEU	D	-0.796	NEU
nsp13	1	483	A	0.294	NEU	V	-0.847	NEU
nsp13	1	484	S	**1.292**	NEU	S	-0.371	NEU
nsp13	1	485	S	**1.292**	NEU	S	-0.375	NEU
nsp13	1	486	A	**0.839**	MIN	A	0.674	NEU
nsp13	1	487	I	**1.292**	MIN	I	1.084	MIN
nsp13	1	488	N	**0.500**	NEU	N	-1.013	MAX
nsp13	1	489	R	**1.016**	NEU	R	-0.606	NEU
nsp13	1	490	P	**1.016**	MAX	P	-1.294	MAX
nsp13	1	491	Q	**1.292**	NEU	Q	-0.847	NEU
nsp13	1	492	L	**1.292**	MIN	I	1.443	MIN
nsp13	1	493	G	**1.292**	NEU	G	-0.607	NEU

nsp13	1	494	F	**1.292**	MIN	V	1.11	MIN
nsp13	1	495	V	**1.292**	MIN	V	1.238	MIN
nsp13	1	496	K	0.334	MAX	R	-0.717	NEU
nsp13	1	497	E	**0.590**	NEU	E	-0.658	NEU
nsp13	1	498	F	**1.292**	MIN	F	1.366	MIN
nsp13	1	499	L	**1.292**	MIN	L	1.645	MIN
nsp13	1	500	T	**0.839**	NEU	T	0.275	NEU
nsp13	1	501	A	**1.292**	NEU	R	0.413	NEU
nsp13	1	502	N	**0.590**	NEU	N	-0.878	NEU
nsp13	1	503	P	**0.701**	MAX	P	-1.496	MAX
nsp13	1	504	A	-0.201	NEU	A	-0.912	NEU
nsp13	1	505	W	**1.292**	NEU	W	0.222	NEU
nsp13	1	506	S	**0.701**	NEU	R	-0.602	NEU
nsp13	1	507	K	0.374	NEU	K	-0.787	NEU
nsp13	1	508	A	**1.292**	NEU	A	0.402	NEU
nsp13	1	509	V	**1.292**	MIN	V	1.146	MIN
nsp13	1	510	F	**1.292**	MIN	F	1.223	MIN
nsp13	1	511	I	**1.292**	MIN	I	1.284	MIN
nsp13	1	512	S	**1.292**	NEU	S	-0.473	NEU
nsp13	1	513	P	**1.292**	MAX	P	-1.335	MAX
nsp13	1	514	Y	0.294	MAX	Y	0.199	NEU
nsp13	1	515	N	**1.292**	MIN	N	0.926	MIN
nsp13	1	516	S	**1.016**	NEU	S	-0.986	NEU
nsp13	1	517	Q	**1.292**	NEU	Q	-0.771	NEU
nsp13	1	518	N	**1.292**	NEU	N	-0.237	NEU
nsp13	1	519	A	**1.016**	NEU	A	-0.794	NEU
nsp13	1	520	V	**1.292**	MIN	V	1.247	MIN
nsp13	1	521	A	**1.016**	NEU	A	0.516	NEU
nsp13	1	522	R	**1.292**	NEU	S	-0.269	NEU
nsp13	1	523	S	**1.292**	NEU	K	-0.718	NEU
nsp13	1	524	M	**0.701**	MIN	I	1.668	MIN
nsp13	1	525	L	**1.292**	MIN	L	1.352	MIN
nsp13	1	526	G	**1.292**	NEU	G	0.022	NEU
nsp13	1	527	L	**1.292**	MIN	L	1.366	MIN
nsp13	1	528	P	**1.292**	NEU	P	0.069	NEU
nsp13	1	529	T	**1.292**	NEU	T	-0.247	NEU
nsp13	1	530	Q	**1.292**	NEU	Q	-0.764	NEU
nsp13	1	531	T	**1.292**	NEU	T	0.236	NEU
nsp13	1	532	V	**1.292**	MIN	V	1.272	MIN
nsp13	1	533	D	**1.292**	NEU	D	-0.799	NEU
nsp13	1	534	S	**1.292**	NEU	S	-0.262	NEU
nsp13	1	535	S	**1.292**	NEU	S	-0.77	NEU
nsp13	1	536	Q	**1.292**	NEU	Q	-0.8	NEU
nsp13	1	537	G	**1.292**	NEU	G	-0.697	NEU
nsp13	1	538	S	**1.292**	NEU	S	-0.124	NEU
nsp13	1	539	E	**1.292**	NEU	E	0.178	NEU

nsp13	1	540	Y	**0.500**	NEU	Y	-0.422	NEU
nsp13	1	541	D	-0.283	NEU	D	-0.552	NEU
nsp13	1	542	Y	0.374	MIN	Y	0.566	NEU
nsp13	1	543	V	**1.292**	MIN	V	1.264	MIN
nsp13	1	544	I	**1.292**	MIN	I	1.574	MIN
nsp13	1	545	F	**1.292**	MIN	F	1.215	MIN
nsp13	1	546	C	0.374	MIN	T	-0.389	NEU
nsp13	1	547	Q	**1.016**	NEU	Q	-0.972	NEU
nsp13	1	548	T	**1.292**	NEU	T	-0.064	NEU
nsp13	1	549	A	**1.292**	NEU	T	-0.038	NEU
nsp13	1	550	D	**1.292**	NEU	E	-0.333	NEU
nsp13	1	551	T	**1.292**	NEU	T	0.083	NEU
nsp13	1	552	A	0.334	MAX	A	-0.865	NEU
nsp13	1	553	H	**1.292**	NEU	H	0.518	NEU
nsp13	1	554	A	**1.292**	NEU	S	-0.342	NEU
nsp13	1	555	N	0.374	NEU	C	2.092	MIN
nsp13	1	556	N	**1.292**	NEU	N	-0.345	NEU
nsp13	1	557	V	**1.292**	MIN	V	1.225	MIN
nsp13	1	558	N	**1.292**	NEU	N	-0.669	NEU
nsp13	1	559	R	**1.292**	NEU	R	-0.318	NEU
nsp13	1	560	F	**1.292**	MIN	F	1.148	MIN
nsp13	1	561	N	**1.292**	MAX	N	-1.114	MAX
nsp13	1	562	V	**1.292**	MIN	V	1.177	MIN
nsp13	1	563	A	0.429	MIN	A	0.631	NEU
nsp13	1	564	I	**1.292**	MIN	I	1.386	MIN
nsp13	1	565	T	**1.292**	NEU	T	-0.592	NEU
nsp13	1	566	R	**1.292**	NEU	R	-0.624	NEU
nsp13	1	567	A	**1.292**	NEU	A	0.263	NEU
nsp13	1	568	K	0.307	MAX	K	-1.284	MAX
nsp13	1	569	K	-0.253	MAX	V	1.407	MIN
nsp13	1	570	G	**1.292**	NEU	G	-0.39	NEU
nsp13	1	571	I	**1.292**	MIN	I	1.185	MIN
nsp13	1	572	L	**1.292**	MIN	L	1.257	MIN
nsp13	1	573	C	**1.292**	MIN	C	1.113	MIN
nsp13	1	574	V	**1.292**	MIN	I	1.39	MIN
nsp13	1	575	M	**0.701**	MIN	M	0.536	NEU
nsp13	1	576	S	**1.292**	NEU	S	-0.648	NEU
nsp13	1	577	D	0.307	MAX	D	-1.805	MAX
nsp13	1	578	Q	-0.227	MAX	R	1.21	MIN
nsp13	1	579	A	**0.701**	NEU	D	-0.065	NEU
nsp13	1	580	L	**1.016**	MIN	L	0.946	MIN
nsp13	1	581	F	0.294	MIN	Y	-0.171	NEU
nsp13	1	582	D	**0.500**	NEU	D	0.278	NEU
nsp13	1	583	S	0.086	NEU	K	-1.032	MAX
nsp13	1	584	L	**1.292**	MIN	L	1.378	MIN
nsp13	1	585	Q	0.334	NEU	Q	-0.913	NEU

nsp13	1	586	F	**1.292**	MIN	F	1.349	MIN
nsp13	1	587	T	0.435	NEU	T	-0.33	NEU
nsp13	1	588	E	**0.590**	NEU	S	-0.452	NEU
nsp13	1	589	L	**1.292**	MIN	L	1.524	MIN
nsp13	1	590	S	0.143	NEU	E	-0.57	NEU
nsp13	1	591	V	**0.590**	MIN	I	1.501	MIN
nsp13	1	592	N	0.091	MIN	P	-0.108	NEU
nsp14	1	1	E	**0.754**	NEU	E	-0.954	NEU
nsp14	1	2	N	0.012	MIN	N	0.577	NEU
nsp14	1	3	V	0.190	NEU	V	0.528	NEU
nsp14	1	4	T	0.279	NEU	T	-0.293	NEU
nsp14	1	5	G	**1.276**	NEU	G	0.243	NEU
nsp14	1	6	L	**1.276**	MIN	L	0.642	NEU
nsp14	1	7	F	**0.604**	MIN	F	1.049	MIN
nsp14	1	8	K	**0.604**	MAX	K	-1.044	MAX
nsp14	1	9	D	0.298	NEU	D	-1.017	MAX
nsp14	1	10	C	**1.276**	MIN	C	1.65	MIN
nsp14	1	11	S	**0.604**	NEU	S	-0.718	NEU
nsp14	1	12	K	-0.208	NEU	K	-0.911	NEU
nsp14	1	13	A	-0.107	NEU	V	0.801	MIN
nsp14	1	14	E	0.299	NEU	I	-0.527	NEU
nsp14	1	15	T	**0.604**	NEU	T	0.048	NEU
nsp14	1	16	G	**1.201**	NEU	G	-0.172	NEU
nsp14	1	17	L	0.339	MIN	L	0.332	NEU
nsp14	1	18	H	**0.953**	NEU	H	0.22	NEU
nsp14	1	19	P	**1.276**	NEU	P	-0.85	NEU
nsp14	1	20	A	**0.754**	NEU	T	-0.699	NEU
nsp14	1	21	Y	0.279	NEU	Q	-0.661	NEU
nsp14	1	22	A	0.489	NEU	A	-1.473	MAX
nsp14	1	23	P	0.442	NEU	P	0.244	NEU
nsp14	1	24	T	**1.276**	NEU	T	-0.375	NEU
nsp14	1	25	F	0.190	NEU	H	0.12	NEU
nsp14	1	26	L	0.339	MIN	L	0.479	NEU
nsp14	1	27	S	**0.636**	NEU	S	-1.124	MAX
nsp14	1	28	V	**1.276**	MIN	V	1.274	MIN
nsp14	1	29	D	**1.276**	NEU	D	-0.224	NEU
nsp14	1	30	D	0.299	MIN	T	0.328	NEU
nsp14	1	31	K	-0.208	NEU	K	0.74	NEU
nsp14	1	32	F	**0.953**	MIN	F	0.557	NEU
nsp14	1	33	K	**1.276**	NEU	K	-0.131	NEU
nsp14	1	34	L	0.279	MIN	T	-0.37	NEU
nsp14	1	35	N	0.299	NEU	E	2.093	MIN
nsp14	1	36	G	0.023	NEU	G	-0.381	NEU
nsp14	1	37	L	0.489	NEU	L	0.359	NEU
nsp14	1	38	C	**0.953**	MIN	C	0.653	NEU
nsp14	1	39	V	**0.953**	MIN	V	1.334	MIN

nsp14	1	40	H	**0.953**	NEU	D	-0.042	NEU
nsp14	1	41	F	0.489	MIN	I	1.165	MIN
nsp14	1	42	D	-0.103	NEU	P	0.471	NEU
nsp14	1	43	T	**0.636**	NEU	G	-0.085	NEU
nsp14	1	44	I	0.489	MIN	I	1.535	MIN
nsp14	1	45	E	**0.953**	NEU	P	0.327	NEU
nsp14	1	46	K	0.489	MIN	K	1.152	MIN
nsp14	1	47	Q	0.012	NEU	D	-0.271	NEU
nsp14	1	48	M	0.110	NEU	M	-0.278	NEU
nsp14	1	49	P	0.402	NEU	T	-0.011	NEU
nsp14	1	50	Y	0.012	MIN	Y	-0.698	NEU
nsp14	1	51	R	-0.246	NEU	R	-0.384	NEU
nsp14	1	52	R	0.299	NEU	R	0.268	NEU
nsp14	1	53	L	**1.276**	MIN	L	0.916	MIN
nsp14	1	54	I	**1.276**	MIN	I	1.117	MIN
nsp14	1	55	S	**1.276**	NEU	S	-0.616	NEU
nsp14	1	56	M	0.299	MIN	M	1.165	MIN
nsp14	1	57	M	**0.604**	MIN	M	0.224	NEU
nsp14	1	58	G	**0.754**	NEU	G	-0.069	NEU
nsp14	1	59	F	**1.276**	MIN	F	1.111	MIN
nsp14	1	60	K	-0.246	MAX	K	-1.767	MAX
nsp14	1	61	F	0.279	NEU	M	-0.2	NEU
nsp14	1	62	D	**0.953**	MIN	N	0.82	MIN
nsp14	1	63	Y	-0.126	MIN	Y	-1.639	MAX
nsp14	1	64	Q	**0.953**	NEU	Q	0.451	NEU
nsp14	1	65	V	**0.953**	MIN	V	1.253	MIN
nsp14	1	66	P	0.299	NEU	N	1.03	MIN
nsp14	1	67	G	**0.953**	NEU	G	-0.607	NEU
nsp14	1	68	Y	-0.107	MAX	Y	1.367	MIN
nsp14	1	69	P	**1.276**	NEU	P	-0.652	NEU
nsp14	1	70	K	0.299	NEU	N	-0.981	NEU
nsp14	1	71	L	0.279	MIN	M	-0.008	NEU
nsp14	1	72	F	**1.276**	MIN	F	1.27	MIN
nsp14	1	73	I	**1.276**	MIN	I	2.129	MIN
nsp14	1	74	T	0.279	NEU	T	0.623	NEU
nsp14	1	75	R	**1.276**	NEU	R	-0.013	NEU
nsp14	1	76	E	**1.276**	NEU	E	-0.563	NEU
nsp14	1	77	E	**0.754**	NEU	E	-0.147	NEU
nsp14	1	78	A	**1.276**	NEU	A	0.343	NEU
nsp14	1	79	I	**1.276**	MIN	I	1.378	MIN
nsp14	1	80	K	0.279	MIN	R	0.509	NEU
nsp14	1	81	E	0.279	NEU	H	-0.253	NEU
nsp14	1	82	V	**1.276**	MIN	V	1.27	MIN
nsp14	1	83	R	**1.276**	NEU	R	-0.842	NEU
nsp14	1	84	G	**1.276**	NEU	A	0.416	NEU
nsp14	1	85	W	**1.276**	NEU	W	0.541	NEU

nsp14	1	86	I	**1.276**	MIN	I	1.246	MIN
nsp14	1	87	G	**1.276**	NEU	G	-0.53	NEU
nsp14	1	88	F	**1.276**	MIN	F	1.203	MIN
nsp14	1	89	D	**1.276**	MAX	D	-1.343	MAX
nsp14	1	90	V	**1.276**	MIN	V	1.15	MIN
nsp14	1	91	E	**1.276**	MAX	E	-1.312	MAX
nsp14	1	92	G	**0.953**	NEU	G	-0.249	NEU
nsp14	1	93	A	0.299	NEU	C	1.792	MIN
nsp14	1	94	H	**1.276**	NEU	H	-0.424	NEU
nsp14	1	95	A	0.489	MIN	A	0.991	MIN
nsp14	1	96	C	0.279	NEU	T	-0.403	NEU
nsp14	1	97	G	**0.953**	NEU	R	-0.648	NEU
nsp14	1	98	P	0.023	NEU	E	-0.365	NEU
nsp14	1	99	A	**0.754**	NEU	A	0.386	NEU
nsp14	1	100	V	**0.754**	MIN	V	1.147	MIN
nsp14	1	101	G	0.299	NEU	G	-0.65	NEU
nsp14	1	102	T	**1.276**	NEU	T	-0.257	NEU
nsp14	1	103	N	**1.276**	NEU	N	-0.702	NEU
nsp14	1	104	L	**1.276**	MIN	L	1.178	MIN
nsp14	1	105	P	**1.276**	NEU	P	-0.903	NEU
nsp14	1	106	L	**1.276**	MIN	L	1.069	MIN
nsp14	1	107	Q	**0.953**	NEU	Q	-0.901	NEU
nsp14	1	108	I	**1.276**	MIN	L	1.221	MIN
nsp14	1	109	G	**1.276**	NEU	G	-0.815	NEU
nsp14	1	110	F	**1.276**	MIN	F	1.228	MIN
nsp14	1	111	S	**1.276**	NEU	S	-0.679	NEU
nsp14	1	112	T	**0.953**	NEU	T	-0.227	NEU
nsp14	1	113	G	0.339	NEU	G	-0.986	NEU
nsp14	1	114	V	**0.953**	MIN	V	1.41	MIN
nsp14	1	115	N	**0.953**	NEU	N	-0.808	NEU
nsp14	1	116	F	**0.953**	MIN	L	0.894	MIN
nsp14	1	117	V	**1.276**	MIN	V	1.129	MIN
nsp14	1	118	V	0.279	MIN	A	0.479	NEU
nsp14	1	119	T	0.299	NEU	V	1.189	MIN
nsp14	1	120	P	**0.754**	NEU	P	-0.855	NEU
nsp14	1	121	T	**0.953**	NEU	T	-0.6	NEU
nsp14	1	122	G	**1.276**	NEU	G	-0.449	NEU
nsp14	1	123	Y	-0.107	MAX	Y	-1.225	MAX
nsp14	1	124	V	**1.276**	MIN	V	0.96	MIN
nsp14	1	125	D	**0.636**	NEU	D	-0.883	NEU
nsp14	1	126	T	**0.953**	NEU	T	0.227	NEU
nsp14	1	127	E	0.023	MAX	P	0.377	NEU
nsp14	1	128	S	0.190	NEU	N	-0.045	NEU
nsp14	1	129	G	0.442	NEU	N	0.689	NEU
nsp14	1	130	T	**0.953**	NEU	T	0.083	NEU
nsp14	1	131	E	0.402	NEU	D	-0.129	NEU

nsp14	1	132	F	0.299	MIN	F	0.2	NEU
nsp14	1	133	A	**0.754**	NEU	S	-0.311	NEU
nsp14	1	134	R	**0.953**	NEU	R	-0.691	NEU
nsp14	1	135	V	**1.276**	MIN	V	1.234	MIN
nsp14	1	136	V	**0.636**	NEU	S	-0.596	NEU
nsp14	1	137	A	0.279	MIN	A	0.604	NEU
nsp14	1	138	K	0.299	NEU	K	-1.56	MAX
nsp14	1	139	P	**0.754**	NEU	P	-0.611	NEU
nsp14	1	140	P	0.299	NEU	P	0.798	MIN
nsp14	1	141	P	**0.953**	MIN	P	0.992	MIN
nsp14	1	142	G	**1.276**	NEU	G	0.033	NEU
nsp14	1	143	D	**0.953**	MIN	D	2.192	MIN
nsp14	1	144	Q	**0.953**	NEU	Q	-0.632	NEU
nsp14	1	145	F	**0.953**	MIN	F	1.13	MIN
nsp14	1	146	K	0.299	MIN	K	0.722	NEU
nsp14	1	147	H	0.442	MIN	H	0.82	MIN
nsp14	1	148	L	**1.276**	MIN	L	0.924	MIN
nsp14	1	149	I	**0.953**	MIN	I	1.723	MIN
nsp14	1	150	P	0.279	NEU	P	0.645	NEU
nsp14	1	151	L	**1.276**	MIN	L	0.884	MIN
nsp14	1	152	M	**0.604**	MIN	M	0.718	NEU
nsp14	1	153	R	0.442	NEU	Y	0.249	NEU
nsp14	1	154	K	0.279	NEU	K	-1.259	MAX
nsp14	1	155	G	**1.276**	NEU	G	-0.47	NEU
nsp14	1	156	E	0.012	MIN	L	1.338	MIN
nsp14	1	157	P	**0.754**	MAX	P	-1.148	MAX
nsp14	1	158	W	0.279	MIN	W	0.522	NEU
nsp14	1	159	N	**0.953**	NEU	N	-0.029	NEU
nsp14	1	160	V	**0.754**	MIN	V	1.363	MIN
nsp14	1	161	V	**1.276**	MIN	V	1.372	MIN
nsp14	1	162	R	**1.276**	NEU	R	-0.622	NEU
nsp14	1	163	K	0.023	NEU	I	1.679	MIN
nsp14	1	164	R	0.299	NEU	K	-1.471	MAX
nsp14	1	165	I	**1.276**	MIN	I	1.458	MIN
nsp14	1	166	V	**1.276**	MIN	V	1.137	MIN
nsp14	1	167	E	0.299	NEU	Q	-1.366	MAX
nsp14	1	168	M	**0.754**	MIN	M	0.561	NEU
nsp14	1	169	L	**0.754**	MIN	L	1.334	MIN
nsp14	1	170	C	**0.604**	NEU	S	-0.8	NEU
nsp14	1	171	D	0.299	NEU	D	-0.205	NEU
nsp14	1	172	T	**1.276**	NEU	T	-0.271	NEU
nsp14	1	173	L	**1.276**	MIN	L	1.539	MIN
nsp14	1	174	D	**0.953**	NEU	K	0.34	NEU
nsp14	1	175	G	**1.276**	NEU	N	-0.403	NEU
nsp14	1	176	V	**1.276**	MIN	L	1.219	MIN
nsp14	1	177	S	**1.276**	NEU	S	-0.51	NEU

nsp14	1	178	D	0.299	MIN	D	0.355	NEU
nsp14	1	179	R	**0.953**	NEU	R	-0.863	NEU
nsp14	1	180	V	**1.276**	MIN	V	1.103	MIN
nsp14	1	181	V	0.279	MIN	V	1.143	MIN
nsp14	1	182	F	**1.276**	MIN	F	1.442	MIN
nsp14	1	183	V	**1.276**	MIN	V	1.257	MIN
nsp14	1	184	T	0.299	NEU	L	1.055	MIN
nsp14	1	185	W	**1.276**	MIN	W	0.94	MIN
nsp14	1	186	A	**1.276**	NEU	A	-0.783	NEU
nsp14	1	187	H	**1.276**	NEU	H	-0.201	NEU
nsp14	1	188	G	**1.276**	NEU	G	-0.691	NEU
nsp14	1	189	F	**0.953**	NEU	F	0.08	NEU
nsp14	1	190	E	**1.276**	MAX	E	-1.544	MAX
nsp14	1	191	L	**1.276**	MIN	L	1.405	MIN
nsp14	1	192	T	**1.276**	NEU	T	-0.163	NEU
nsp14	1	193	T	**1.276**	NEU	S	-0.78	NEU
nsp14	1	194	M	0.299	MIN	M	0.414	NEU
nsp14	1	195	H	0.279	NEU	K	-1.295	MAX
nsp14	1	196	Y	**0.953**	NEU	Y	-0.63	NEU
nsp14	1	197	F	**1.276**	MIN	F	1.343	MIN
nsp14	1	198	V	0.339	MIN	V	1.305	MIN
nsp14	1	199	K	0.489	MAX	K	-1.171	MAX
nsp14	1	200	I	**0.754**	MIN	I	1.92	MIN
nsp14	1	201	G	**1.276**	NEU	G	0.023	NEU
nsp14	1	202	P	0.254	NEU	P	-0.258	NEU
nsp14	1	203	E	0.339	NEU	E	0.148	NEU
nsp14	1	204	R	0.279	NEU	R	0.913	MIN
nsp14	1	205	T	**0.953**	NEU	T	-0.946	NEU
nsp14	1	206	C	**1.276**	MIN	C	1.677	MIN
nsp14	1	207	C	**0.953**	MIN	C	1.271	MIN
nsp14	1	208	L	-0.220	MIN	L	0.72	NEU
nsp14	1	209	C	**1.276**	MIN	C	0.821	MIN
nsp14	1	210	D	0.279	MIN	D	0.936	MIN
nsp14	1	211	K	**0.953**	NEU	R	-0.39	NEU
nsp14	1	212	R	**0.953**	MIN	R	2.259	MIN
nsp14	1	213	A	**1.276**	NEU	A	-0.079	NEU
nsp14	1	214	T	0.190	NEU	T	-0.941	NEU
nsp14	1	215	C	**0.953**	MIN	C	1.435	MIN
nsp14	1	216	F	0.402	MIN	F	1.338	MIN
nsp14	1	217	S	**0.953**	NEU	S	-0.626	NEU
nsp14	1	218	S	**1.276**	NEU	T	0.222	NEU
nsp14	1	219	V	0.023	NEU	A	-1.002	MAX
nsp14	1	220	Y	**1.276**	NEU	S	-0.224	NEU
nsp14	1	221	D	0.279	NEU	D	-1.126	MAX
nsp14	1	222	A	**1.276**	NEU	T	-0.077	NEU
nsp14	1	223	Y	-0.246	NEU	Y	0.753	NEU

nsp14	1	224	A	**1.276**	NEU	A	-0.066	NEU
nsp14	1	225	C	**1.276**	MIN	C	2.325	MIN
nsp14	1	226	W	**0.953**	NEU	W	-0.107	NEU
nsp14	1	227	S	**0.953**	NEU	H	-0.825	NEU
nsp14	1	228	H	**1.276**	NEU	H	-0.209	NEU
nsp14	1	229	H	**0.953**	NEU	S	-0.673	NEU
nsp14	1	230	G	0.279	MIN	I	2.356	MIN
nsp14	1	231	G	**0.953**	NEU	G	-0.916	NEU
nsp14	1	232	A	**0.604**	NEU	F	0.582	NEU
nsp14	1	233	D	0.279	MAX	D	-1.172	MAX
nsp14	1	234	Y	0.279	MIN	Y	0.487	NEU
nsp14	1	235	V	**1.276**	MIN	V	1.088	MIN
nsp14	1	236	Y	**1.276**	MIN	Y	0.73	NEU
nsp14	1	237	N	0.299	NEU	N	-1.038	MAX
nsp14	1	238	P	0.299	NEU	P	-0.951	NEU
nsp14	1	239	F	**1.276**	MIN	F	1.458	MIN
nsp14	1	240	L	**1.276**	MIN	M	0.743	NEU
nsp14	1	241	V	**1.276**	MIN	I	1.226	MIN
nsp14	1	242	D	0.402	MAX	D	-1.159	MAX
nsp14	1	243	V	**1.276**	MIN	V	1.266	MIN
nsp14	1	244	Q	**1.276**	NEU	Q	-0.804	NEU
nsp14	1	245	Q	**0.754**	NEU	Q	-0.88	NEU
nsp14	1	246	W	**1.276**	NEU	W	0.398	NEU
nsp14	1	247	G	**1.276**	NEU	G	-0.525	NEU
nsp14	1	248	Y	-0.220	MIN	F	1.308	MIN
nsp14	1	249	V	**1.276**	NEU	T	0.446	NEU
nsp14	1	250	G	**1.276**	NEU	G	-0.29	NEU
nsp14	1	251	N	0.279	NEU	N	0.714	NEU
nsp14	1	252	L	**1.276**	MIN	L	1.025	MIN
nsp14	1	253	Q	**1.276**	NEU	Q	-0.058	NEU
nsp14	1	254	S	0.489	NEU	S	0.389	NEU
nsp14	1	255	N	0.402	NEU	N	-1.088	MAX
nsp14	1	256	H	0.402	NEU	H	0.284	NEU
nsp14	1	257	D	**0.604**	NEU	D	0.134	NEU
nsp14	1	258	N	-0.057	NEU	L	0.286	NEU
nsp14	1	259	V	0.055	MIN	Y	-2.137	MAX
nsp14	1	260	C	**1.276**	MIN	C	1.241	MIN
nsp14	1	261	D	0.279	MIN	Q	-0.378	NEU
nsp14	1	262	V	**0.953**	MIN	V	1.25	MIN
nsp14	1	263	H	0.489	MIN	H	0.864	MIN
nsp14	1	264	G	0.279	NEU	G	-0.518	NEU
nsp14	1	265	G	0.299	NEU	N	-1.06	MAX
nsp14	1	266	A	**1.276**	MAX	A	-1.24	MAX
nsp14	1	267	H	**0.953**	MIN	H	0.709	NEU
nsp14	1	268	V	**0.953**	NEU	V	-0.079	NEU
nsp14	1	269	A	0.279	MIN	A	0.497	NEU

nsp14	1	270	S	**1.276**	NEU	S	-0.752	NEU
nsp14	1	271	C	-0.103	MIN	C	0.864	MIN
nsp14	1	272	D	**0.953**	MAX	D	-1.062	MAX
nsp14	1	273	A	**1.276**	NEU	A	0.198	NEU
nsp14	1	274	I	**0.953**	MIN	I	1.135	MIN
nsp14	1	275	M	**1.276**	NEU	M	-0.122	NEU
nsp14	1	276	T	**1.276**	NEU	T	-0.187	NEU
nsp14	1	277	R	**1.276**	NEU	R	-0.789	NEU
nsp14	1	278	C	**1.276**	MIN	C	1.657	MIN
nsp14	1	279	L	**1.276**	MIN	L	1.03	MIN
nsp14	1	280	A	**1.276**	NEU	A	0.046	NEU
nsp14	1	281	I	**1.276**	MIN	V	1.234	MIN
nsp14	1	282	H	**1.276**	NEU	H	-0.033	NEU
nsp14	1	283	D	**1.276**	NEU	E	-0.532	NEU
nsp14	1	284	C	**1.276**	MIN	C	1.267	MIN
nsp14	1	285	F	**1.276**	MIN	F	1.341	MIN
nsp14	1	286	V	**1.276**	MIN	V	1.528	MIN
nsp14	1	287	K	0.489	NEU	K	-0.047	NEU
nsp14	1	288	E	-0.126	MIN	R	1.616	MIN
nsp14	1	289	V	**1.276**	MIN	V	1.533	MIN
nsp14	1	290	N	0.442	NEU	D	-0.354	NEU
nsp14	1	291	W	0.254	NEU	W	-1.862	MAX
nsp14	1	292	D	0.299	MIN	T	0.38	NEU
nsp14	1	293	V	**1.276**	MIN	I	1.803	MIN
nsp14	1	294	E	0.299	MIN	E	1.182	MIN
nsp14	1	295	Y	0.117	NEU	Y	-1.454	MAX
nsp14	1	296	P	0.489	MIN	P	0.529	NEU
nsp14	1	297	I	**0.754**	MIN	I	1.467	MIN
nsp14	1	298	I	**1.276**	MIN	I	1.417	MIN
nsp14	1	299	A	0.489	NEU	G	-1.091	MAX
nsp14	1	300	D	**0.754**	NEU	D	-0.664	NEU
nsp14	1	301	E	0.489	MAX	E	-1.102	MAX
nsp14	1	302	L	0.442	MIN	L	1.099	MIN
nsp14	1	303	A	**0.754**	NEU	K	-0.198	NEU
nsp14	1	304	I	**1.276**	MIN	I	1.418	MIN
nsp14	1	305	N	**1.276**	NEU	N	-0.694	NEU
nsp14	1	306	K	-0.126	NEU	A	0.508	NEU
nsp14	1	307	A	0.489	NEU	A	0.462	NEU
nsp14	1	308	C	**1.276**	MIN	C	1.188	MIN
nsp14	1	309	R	**1.276**	NEU	R	-0.562	NEU
nsp14	1	310	K	0.298	NEU	K	-1.079	MAX
nsp14	1	311	V	**1.276**	MIN	V	1.186	MIN
nsp14	1	312	Q	0.339	MAX	Q	-1.079	MAX
nsp14	1	313	R	0.489	NEU	H	-0.244	NEU
nsp14	1	314	M	0.339	MIN	M	0.485	NEU
nsp14	1	315	V	**0.754**	MIN	V	1.5	MIN

nsp14	1	316	L	**1.276**	MIN	V	1.281	MIN
nsp14	1	317	K	**0.754**	MAX	K	-1.273	MAX
nsp14	1	318	A	**0.953**	NEU	A	0.551	NEU
nsp14	1	319	A	**0.953**	MIN	A	0.555	NEU
nsp14	1	320	L	**0.953**	MIN	L	1.587	MIN
nsp14	1	321	L	-0.171	MIN	L	1.533	MIN
nsp14	1	322	A	0.402	NEU	A	0.608	NEU
nsp14	1	323	D	-0.126	MIN	D	-1.174	MAX
nsp14	1	324	K	0.012	MIN	K	0.429	NEU
nsp14	1	325	F	**0.953**	MIN	F	0.772	NEU
nsp14	1	326	P	0.279	NEU	P	-1.177	MAX
nsp14	1	327	T	0.299	NEU	V	1.092	MIN
nsp14	1	328	I	**1.276**	MIN	L	1.182	MIN
nsp14	1	329	H	0.489	NEU	H	0.066	NEU
nsp14	1	330	D	0.339	MAX	D	-1.458	MAX
nsp14	1	331	I	**1.276**	MIN	I	1.107	MIN
nsp14	1	332	G	**1.276**	NEU	G	-0.141	NEU
nsp14	1	333	N	**1.276**	NEU	N	-0.696	NEU
nsp14	1	334	P	0.279	NEU	P	-0.914	NEU
nsp14	1	335	K	**1.276**	MAX	K	-1.576	MAX
nsp14	1	336	A	0.339	MIN	A	-0.322	NEU
nsp14	1	337	I	0.489	NEU	I	0.48	NEU
nsp14	1	338	K	0.339	NEU	K	-1.034	MAX
nsp14	1	339	C	**0.953**	MIN	C	1.509	MIN
nsp14	1	340	V	-0.246	MIN	V	1.136	MIN
nsp14	1	341	G	**0.934**	NEU	P	-0.944	NEU
nsp14	1	342	V	0.316	MIN	Q	-0.031	NEU
nsp14	1	343	A	**0.636**	NEU	A	0.338	NEU
nsp14	1	344	V	**0.754**	MIN	D	0.87	MIN
nsp14	1	345	V	0.279	NEU	V	1.228	MIN
nsp14	1	346	N	0.190	NEU	E	-0.892	NEU
nsp14	1	347	W	**0.953**	NEU	W	-0.003	NEU
nsp14	1	348	K	**0.604**	NEU	K	-0.674	NEU
nsp14	1	349	F	0.489	NEU	F	-0.012	NEU
nsp14	1	350	Y	-0.107	MAX	Y	0.753	NEU
nsp14	1	351	D	**0.754**	NEU	D	-1.269	MAX
nsp14	1	352	A	**0.754**	NEU	A	0.141	NEU
nsp14	1	353	K	0.190	NEU	Q	-0.65	NEU
nsp14	1	354	P	**1.276**	NEU	P	0.513	NEU
nsp14	1	355	V	**1.276**	MIN	C	0.936	MIN
nsp14	1	356	V	0.279	NEU	S	0.394	NEU
nsp14	1	357	D	0.110	NEU	D	-0.962	NEU
nsp14	1	358	-	**0.637**	MAX	K	-1.476	MAX
nsp14	1	359	-	**0.637**	MAX	A	-1.08	MAX
nsp14	1	360	-	**0.637**	MAX	Y	-2.014	MAX
nsp14	1	361	K	0.299	NEU	K	1.081	MIN

nsp14	1	362	V	**0.953**	MIN	I	1.053	MIN
nsp14	1	363	E	**0.953**	NEU	E	0.161	NEU
nsp14	1	364	E	**0.953**	NEU	E	-0.767	NEU
nsp14	1	365	L	**0.953**	MIN	L	0.926	MIN
nsp14	1	366	H	0.110	NEU	F	-0.731	NEU
nsp14	1	367	Y	**0.953**	NEU	Y	-0.33	NEU
nsp14	1	368	S	**0.754**	NEU	S	0.013	NEU
nsp14	1	369	Y	0.489	MAX	Y	-2.88	MAX
nsp14	1	370	A	-0.290	MAX	A	-1.071	MAX
nsp14	1	371	T	**0.754**	NEU	T	-0.224	NEU
nsp14	1	372	H	**1.276**	NEU	H	-0.333	NEU
nsp14	1	373	K	**0.754**	NEU	S	-0.586	NEU
nsp14	1	374	D	**0.604**	MIN	D	0.628	NEU
nsp14	1	375	Q	-0.304	MIN	K	0.395	NEU
nsp14	1	376	F	0.279	NEU	F	0.869	MIN
nsp14	1	377	K	0.023	MIN	T	0.193	NEU
nsp14	1	378	D	0.015	NEU	D	-0.877	NEU
nsp14	1	379	G	**1.276**	NEU	G	-0.393	NEU
nsp14	1	380	L	**1.276**	MIN	V	1.21	MIN
nsp14	1	381	C	0.279	NEU	C	1.468	MIN
nsp14	1	382	L	**1.276**	MIN	L	0.938	MIN
nsp14	1	383	F	**1.276**	MIN	F	1.279	MIN
nsp14	1	384	W	0.279	NEU	W	0.663	NEU
nsp14	1	385	N	**1.276**	NEU	N	-0.58	NEU
nsp14	1	386	C	**1.276**	MIN	C	0.992	MIN
nsp14	1	387	N	0.279	NEU	N	-0.963	NEU
nsp14	1	388	V	**1.276**	MIN	V	1.23	MIN
nsp14	1	389	D	**0.754**	MAX	D	-1.218	MAX
nsp14	1	390	C	0.279	MIN	R	0.236	NEU
nsp14	1	391	Y	0.402	MIN	Y	0.56	NEU
nsp14	1	392	P	0.299	MAX	P	-1.014	MAX
nsp14	1	393	A	-0.304	MIN	A	-1.164	MAX
nsp14	1	394	N	**0.604**	NEU	N	-1.002	MAX
nsp14	1	395	A	**1.276**	NEU	S	-0.693	NEU
nsp14	1	396	L	**1.276**	MIN	I	1.36	MIN
nsp14	1	397	V	**1.276**	MIN	V	1.307	MIN
nsp14	1	398	C	**1.276**	MIN	C	1.141	MIN
nsp14	1	399	R	**1.276**	NEU	R	-0.355	NEU
nsp14	1	400	F	**1.276**	MIN	F	1.21	MIN
nsp14	1	401	D	0.298	NEU	D	0.037	NEU
nsp14	1	402	T	**1.276**	NEU	T	-0.367	NEU
nsp14	1	403	R	**1.276**	MIN	R	2.185	MIN
nsp14	1	404	V	**1.276**	NEU	V	-0.109	NEU
nsp14	1	405	L	**0.953**	NEU	L	-0.529	NEU
nsp14	1	406	S	**1.276**	NEU	S	-0.572	NEU
nsp14	1	407	K	-0.103	MAX	N	0.93	MIN

nsp14	1	408	L	**1.276**	MIN	L	0.774	NEU
nsp14	1	409	N	**1.276**	NEU	N	-0.873	NEU
nsp14	1	410	L	**1.276**	MIN	L	0.976	MIN
nsp14	1	411	P	0.339	MIN	P	0.932	MIN
nsp14	1	412	G	**1.276**	NEU	G	-0.191	NEU
nsp14	1	413	C	**1.276**	MIN	C	1.593	MIN
nsp14	1	414	N	0.279	MAX	D	-1.486	MAX
nsp14	1	415	G	**1.276**	NEU	G	0.129	NEU
nsp14	1	416	G	**1.276**	NEU	G	-0.244	NEU
nsp14	1	417	S	**1.276**	NEU	S	-0.01	NEU
nsp14	1	418	L	**1.276**	MIN	L	0.898	MIN
nsp14	1	419	Y	**0.953**	NEU	Y	-0.653	NEU
nsp14	1	420	V	**1.276**	MIN	V	1.166	MIN
nsp14	1	421	N	**1.276**	NEU	N	-0.823	NEU
nsp14	1	422	K	**0.754**	NEU	K	-0.306	NEU
nsp14	1	423	H	**1.276**	NEU	H	-0.018	NEU
nsp14	1	424	A	**1.276**	NEU	A	0.499	NEU
nsp14	1	425	F	**1.276**	MIN	F	1.158	MIN
nsp14	1	426	H	**1.276**	NEU	H	-0.392	NEU
nsp14	1	427	T	**1.276**	NEU	T	-0.368	NEU
nsp14	1	428	P	**0.754**	NEU	P	0.035	NEU
nsp14	1	429	A	**0.604**	NEU	A	0.157	NEU
nsp14	1	430	F	0.254	NEU	F	-0.233	NEU
nsp14	1	431	D	**1.276**	NEU	D	0.167	NEU
nsp14	1	432	K	**0.953**	MAX	K	-1.534	MAX
nsp14	1	433	S	0.489	NEU	S	-0.26	NEU
nsp14	1	434	A	**1.276**	NEU	A	-0.217	NEU
nsp14	1	435	F	**1.276**	NEU	F	-0.441	NEU
nsp14	1	436	V	0.023	NEU	V	1.041	MIN
nsp14	1	437	N	**0.604**	NEU	N	0.131	NEU
nsp14	1	438	L	**1.276**	MIN	L	1.127	MIN
nsp14	1	439	K	**0.953**	MAX	K	-2.131	MAX
nsp14	1	440	P	0.299	NEU	Q	-1.008	MAX
nsp14	1	441	L	**1.276**	MIN	L	1.407	MIN
nsp14	1	442	P	**1.276**	NEU	P	-0.601	NEU
nsp14	1	443	F	**0.604**	NEU	F	0.209	NEU
nsp14	1	444	F	**1.276**	MIN	F	0.932	MIN
nsp14	1	445	Y	**0.953**	MIN	Y	0.612	NEU
nsp14	1	446	Y	**0.953**	MIN	Y	0.544	NEU
nsp14	1	447	S	**0.953**	NEU	S	-0.117	NEU
nsp14	1	448	D	**0.754**	NEU	D	0.511	NEU
nsp14	1	449	T	**0.953**	NEU	S	0.006	NEU
nsp14	1	450	P	0.279	MIN	P	0.981	MIN
nsp14	1	451	C	**1.276**	MIN	C	2.492	MIN
nsp14	1	452	E	0.254	NEU	E	0.745	NEU
nsp14	1	453	S	-0.148	MIN	S	0.061	NEU

nsp14	1	454	A	0.023	NEU	H	1.089	MIN
nsp14	1	455	G	0.023	NEU	G	0.024	NEU
nsp14	1	456	G	0.316	NEU	K	-0.03	NEU
nsp14	1	457	Q	-0.246	MAX	Q	-0.505	NEU
nsp14	1	458	V	0.190	NEU	V	-0.209	NEU
nsp14	1	459	V	0.299	MIN	V	0.75	NEU
nsp14	1	460	S	0.254	NEU	S	-0.741	NEU
nsp14	1	461	D	0.299	MAX	D	-0.403	NEU
nsp14	1	462	V	**1.276**	MIN	I	2.018	MIN
nsp14	1	463	D	0.110	NEU	D	-0.327	NEU
nsp14	1	464	Y	0.442	NEU	Y	0.125	NEU
nsp14	1	465	V	**1.276**	MIN	V	1.278	MIN
nsp14	1	466	P	**1.276**	NEU	P	-0.718	NEU
nsp14	1	467	L	**1.276**	MIN	L	1.066	MIN
nsp14	1	468	K	0.190	NEU	K	-0.091	NEU
nsp14	1	469	S	**1.276**	NEU	S	-0.465	NEU
nsp14	1	470	N	**0.953**	NEU	A	-1.108	MAX
nsp14	1	471	V	0.023	MIN	T	-0.564	NEU
nsp14	1	472	C	**1.276**	MIN	C	1.857	MIN
nsp14	1	473	I	**1.276**	MIN	I	1.155	MIN
nsp14	1	474	T	**1.276**	NEU	T	-0.764	NEU
nsp14	1	475	R	**0.604**	NEU	R	1.205	MIN
nsp14	1	476	C	**1.276**	MIN	C	1.493	MIN
nsp14	1	477	N	0.489	MAX	N	-1.028	MAX
nsp14	1	478	L	**1.276**	MIN	L	1.15	MIN
nsp14	1	479	G	**0.953**	NEU	G	-1.204	MAX
nsp14	1	480	G	**1.276**	NEU	G	-0.496	NEU
nsp14	1	481	A	**0.604**	NEU	A	0.511	NEU
nsp14	1	482	V	**1.276**	MIN	V	1.511	MIN
nsp14	1	483	C	**1.276**	MIN	C	2.177	MIN
nsp14	1	484	K	**0.754**	NEU	R	-0.186	NEU
nsp14	1	485	K	0.110	NEU	H	0.905	MIN
nsp14	1	486	H	**0.754**	NEU	H	0.007	NEU
nsp14	1	487	A	**0.953**	NEU	A	0.127	NEU
nsp14	1	488	D	-0.126	NEU	N	-0.939	NEU
nsp14	1	489	E	**0.754**	MAX	E	-1.311	MAX
nsp14	1	490	Y	**1.276**	NEU	Y	-0.171	NEU
nsp14	1	491	R	0.279	NEU	R	0.298	NEU
nsp14	1	492	Q	-0.057	NEU	L	0.175	NEU
nsp14	1	493	F	0.279	MIN	Y	-0.204	NEU
nsp14	1	494	L	**1.276**	MIN	L	1.109	MIN
nsp14	1	495	E	**0.953**	NEU	D	-0.456	NEU
nsp14	1	496	A	0.442	NEU	A	0.421	NEU
nsp14	1	497	Y	**1.276**	NEU	Y	0.124	NEU
nsp14	1	498	N	0.299	MAX	N	-0.832	NEU
nsp14	1	499	T	**0.754**	NEU	M	0.064	NEU

nsp14	1	500	M	**0.754**	MIN	M	0.803	MIN
nsp14	1	501	I	**0.953**	MIN	I	1.565	MIN
nsp14	1	502	S	**0.953**	NEU	S	-0.469	NEU
nsp14	1	503	A	**1.276**	NEU	A	0.329	NEU
nsp14	1	504	G	**1.276**	NEU	G	-0.395	NEU
nsp14	1	505	F	**1.276**	MIN	F	1.193	MIN
nsp14	1	506	T	**1.276**	NEU	S	-0.029	NEU
nsp14	1	507	L	**1.276**	MIN	L	1.149	MIN
nsp14	1	508	W	**0.604**	MIN	W	0.589	NEU
nsp14	1	509	V	**1.276**	MIN	V	1.294	MIN
nsp14	1	510	Y	-0.171	NEU	Y	0.564	NEU
nsp14	1	511	K	**0.604**	MAX	K	-1.55	MAX
nsp14	1	512	Q	0.442	NEU	Q	-0.475	NEU
nsp14	1	513	F	0.489	MIN	F	0.596	NEU
nsp14	1	514	D	**0.754**	MAX	D	-1.348	MAX
nsp14	1	515	T	0.279	MIN	T	-0.377	NEU
nsp14	1	516	Y	0.012	MIN	Y	-0.573	NEU
nsp14	1	517	N	0.279	MIN	N	0.718	NEU
nsp14	1	518	L	**1.276**	MIN	L	1.187	MIN
nsp14	1	519	W	0.190	NEU	W	-1.113	MAX
nsp14	1	520	S	**0.953**	NEU	N	-0.315	NEU
nsp14	1	521	T	**0.953**	NEU	T	-0.342	NEU
nsp14	1	522	F	**1.276**	MIN	F	1.219	MIN
nsp15	1	1	S	**1.301**	NEU	S	-0.345	NEU
nsp15	1	2	L	**1.301**	MIN	L	0.961	MIN
nsp15	1	3	E	**0.563**	MAX	E	-1.091	MAX
nsp15	1	4	N	**1.301**	NEU	N	-0.382	NEU
nsp15	1	5	V	**1.301**	MIN	V	1.468	MIN
nsp15	1	6	A	**1.301**	MIN	A	0.86	MIN
nsp15	1	7	Y	0.260	MAX	F	0.956	MIN
nsp15	1	8	N	**1.301**	NEU	N	-0.499	NEU
nsp15	1	9	V	**1.301**	MIN	V	1.32	MIN
nsp15	1	10	V	**1.301**	MIN	V	1.557	MIN
nsp15	1	11	N	**1.301**	NEU	N	-0.638	NEU
nsp15	1	12	K	0.321	MAX	K	-1.016	MAX
nsp15	1	13	G	**1.301**	NEU	G	-0.701	NEU
nsp15	1	14	H	**1.301**	NEU	H	-0.87	NEU
nsp15	1	15	F	**1.051**	NEU	F	0.216	NEU
nsp15	1	16	D	**1.301**	MIN	D	2.041	MIN
nsp15	1	17	G	**0.651**	NEU	G	-1.142	MAX
nsp15	1	18	Q	**1.301**	NEU	Q	0.108	NEU
nsp15	1	19	Q	0.126	NEU	Q	-0.104	NEU
nsp15	1	20	G	**0.651**	NEU	G	-1.157	MAX
nsp15	1	21	E	**1.301**	MAX	E	-1.433	MAX
nsp15	1	22	A	0.490	NEU	V	1.32	MIN
nsp15	1	23	P	0.321	MAX	P	-1.385	MAX

nsp15	1	24	V	**1.301**	MIN	V	1.29	MIN
nsp15	1	25	S	**0.651**	MAX	S	-1.087	MAX
nsp15	1	26	I	**1.301**	MIN	I	1.62	MIN
nsp15	1	27	I	**1.301**	MIN	I	1.642	MIN
nsp15	1	28	N	**1.301**	NEU	N	-0.483	NEU
nsp15	1	29	N	0.490	NEU	N	-0.987	NEU
nsp15	1	30	A	**1.301**	NEU	T	0.083	NEU
nsp15	1	31	V	**1.301**	MIN	V	1.549	MIN
nsp15	1	32	Y	**1.301**	NEU	Y	-0.493	NEU
nsp15	1	33	T	**1.301**	NEU	T	-0.224	NEU
nsp15	1	34	K	**1.301**	NEU	K	-0.801	NEU
nsp15	1	35	V	**1.301**	MIN	V	1.415	MIN
nsp15	1	36	D	**0.757**	NEU	D	-0.702	NEU
nsp15	1	37	G	**1.301**	NEU	G	-0.29	NEU
nsp15	1	38	V	0.412	MIN	V	1.087	MIN
nsp15	1	39	D	**1.301**	NEU	D	-0.771	NEU
nsp15	1	40	V	**1.301**	MIN	V	1.602	MIN
nsp15	1	41	E	0.430	MAX	E	-0.803	NEU
nsp15	1	42	I	**1.301**	MIN	L	1.167	MIN
nsp15	1	43	F	**1.301**	MIN	F	1.041	MIN
nsp15	1	44	E	**1.301**	NEU	E	-0.247	NEU
nsp15	1	45	N	**1.301**	NEU	N	-0.267	NEU
nsp15	1	46	K	**1.301**	MIN	K	2.021	MIN
nsp15	1	47	T	**1.301**	NEU	T	-0.677	NEU
nsp15	1	48	T	**1.051**	NEU	T	-0.391	NEU
nsp15	1	49	L	**1.301**	MIN	L	1.077	MIN
nsp15	1	50	P	**1.301**	NEU	P	-0.44	NEU
nsp15	1	51	V	**1.301**	MIN	V	1.085	MIN
nsp15	1	52	N	**1.301**	MAX	N	-1.096	MAX
nsp15	1	53	V	**1.301**	MIN	V	1.228	MIN
nsp15	1	54	A	**1.301**	NEU	A	-0.023	NEU
nsp15	1	55	F	**1.301**	MIN	F	0.714	NEU
nsp15	1	56	E	**1.301**	NEU	E	-0.883	NEU
nsp15	1	57	L	**1.301**	MIN	L	1.327	MIN
nsp15	1	58	W	**1.301**	NEU	W	0.245	NEU
nsp15	1	59	A	**1.301**	NEU	A	0.471	NEU
nsp15	1	60	K	**1.301**	MAX	K	-1.201	MAX
nsp15	1	61	R	**1.301**	NEU	R	-0.743	NEU
nsp15	1	62	N	**0.804**	MAX	N	-1.145	MAX
nsp15	1	63	I	0.490	NEU	I	0.311	NEU
nsp15	1	64	K	0.301	MIN	K	0.593	NEU
nsp15	1	65	P	0.490	MAX	P	-1.198	MAX
nsp15	1	66	V	**1.301**	MIN	V	1.147	MIN
nsp15	1	67	P	**1.301**	NEU	P	-0.848	NEU
nsp15	1	68	E	**0.887**	MAX	E	-1.069	MAX
nsp15	1	69	I	**1.301**	MIN	V	1.617	MIN

nsp15	1	70	K	**1.301**	NEU	K	-0.546	NEU
nsp15	1	71	I	**1.301**	MIN	I	1.4	MIN
nsp15	1	72	L	**1.301**	MIN	L	1.144	MIN
nsp15	1	73	N	**0.757**	NEU	N	-0.948	NEU
nsp15	1	74	N	**0.563**	NEU	N	-0.982	NEU
nsp15	1	75	L	**1.301**	MIN	L	1.141	MIN
nsp15	1	76	G	**1.301**	NEU	G	-0.647	NEU
nsp15	1	77	V	**1.301**	MIN	V	1.364	MIN
nsp15	1	78	D	**1.301**	NEU	D	-0.398	NEU
nsp15	1	79	I	**1.301**	MIN	I	1.084	MIN
nsp15	1	80	A	**1.301**	NEU	A	0.043	NEU
nsp15	1	81	A	0.430	MIN	A	0.618	NEU
nsp15	1	82	N	0.321	NEU	N	-1.059	MAX
nsp15	1	83	T	**1.301**	NEU	T	-0.141	NEU
nsp15	1	84	V	**1.301**	MIN	V	1.169	MIN
nsp15	1	85	I	**1.301**	MIN	I	1.923	MIN
nsp15	1	86	W	**1.301**	NEU	W	-0.177	NEU
nsp15	1	87	D	**0.887**	NEU	D	-0.744	NEU
nsp15	1	88	Y	**0.757**	MAX	Y	-2.957	MAX
nsp15	1	89	K	**0.887**	MIN	K	1.55	MIN
nsp15	1	90	R	**1.301**	NEU	R	-0.211	NEU
nsp15	1	91	E	**1.301**	NEU	D	0.288	NEU
nsp15	1	92	A	**1.051**	NEU	A	-0.563	NEU
nsp15	1	93	P	**0.651**	NEU	P	-1.051	MAX
nsp15	1	94	A	**1.051**	NEU	A	0.028	NEU
nsp15	1	95	H	**1.301**	NEU	H	-0.164	NEU
nsp15	1	96	V	**1.301**	MIN	I	1.552	MIN
nsp15	1	97	S	**1.301**	NEU	S	-0.703	NEU
nsp15	1	98	T	**1.301**	NEU	T	-0.287	NEU
nsp15	1	99	I	**1.301**	MIN	I	1.949	MIN
nsp15	1	100	G	**1.301**	NEU	G	-0.312	NEU
nsp15	1	101	V	**1.301**	MIN	V	1.067	MIN
nsp15	1	102	C	**1.051**	MIN	C	0.631	NEU
nsp15	1	103	T	**1.051**	NEU	S	0.189	NEU
nsp15	1	104	M	**1.301**	NEU	M	0.03	NEU
nsp15	1	105	T	**1.301**	NEU	T	-0.531	NEU
nsp15	1	106	D	**1.301**	MAX	D	-1.161	MAX
nsp15	1	107	I	**1.301**	MIN	I	2.076	MIN
nsp15	1	108	A	**1.301**	NEU	A	0.3	NEU
nsp15	1	109	K	**1.051**	MIN	K	1.528	MIN
nsp15	1	110	K	**1.301**	NEU	K	-0.569	NEU
nsp15	1	111	P	**1.301**	NEU	P	-0.59	NEU
nsp15	1	112	T	**0.563**	NEU	T	-0.569	NEU
nsp15	1	113	E	**1.051**	NEU	E	-0.177	NEU
nsp15	1	114	S	**0.887**	NEU	T	-0.736	NEU
nsp15	1	115	A	**0.563**	NEU	I	1.363	MIN

nsp15	1	116	C	**1.301**	MIN	C	1.805	MIN
nsp15	1	117	S	**0.516**	NEU	A	-0.864	NEU
nsp15	1	118	S	**1.051**	NEU	P	-0.255	NEU
nsp15	1	119	L	**1.301**	MIN	L	0.975	MIN
nsp15	1	120	T	**1.051**	NEU	T	-0.2	NEU
nsp15	1	121	V	**1.301**	MIN	V	1.22	MIN
nsp15	1	122	L	**1.301**	MIN	F	1.267	MIN
nsp15	1	123	F	**1.051**	MIN	F	0.883	MIN
nsp15	1	124	D	**1.301**	NEU	D	-0.772	NEU
nsp15	1	125	G	**1.301**	NEU	G	-0.503	NEU
nsp15	1	126	R	**1.301**	MIN	R	1.843	MIN
nsp15	1	127	V	**0.757**	MIN	V	0.8	MIN
nsp15	1	128	E	**1.051**	MIN	D	1.009	MIN
nsp15	1	129	G	**1.301**	NEU	G	-0.16	NEU
nsp15	1	130	Q	**1.301**	NEU	Q	-0.744	NEU
nsp15	1	131	V	**1.051**	MIN	V	1.349	MIN
nsp15	1	132	D	**1.301**	MIN	D	1.476	MIN
nsp15	1	133	L	**1.051**	MIN	L	0.913	MIN
nsp15	1	134	F	**1.301**	MIN	F	0.886	MIN
nsp15	1	135	R	**1.051**	NEU	R	-0.507	NEU
nsp15	1	136	N	**0.804**	NEU	N	-0.376	NEU
nsp15	1	137	A	**0.651**	NEU	A	0.497	NEU
nsp15	1	138	R	**1.301**	NEU	R	-0.344	NEU
nsp15	1	139	N	**0.887**	MAX	N	-1.021	MAX
nsp15	1	140	G	**1.301**	NEU	G	-0.549	NEU
nsp15	1	141	V	**1.301**	MIN	V	1.259	MIN
nsp15	1	142	L	**1.051**	MIN	L	0.654	NEU
nsp15	1	143	I	**1.301**	MIN	I	1.23	MIN
nsp15	1	144	T	**1.301**	NEU	T	-0.422	NEU
nsp15	1	145	E	**1.051**	MIN	E	1.8	MIN
nsp15	1	146	G	0.143	NEU	G	0.772	NEU
nsp15	1	147	S	**0.516**	NEU	S	0.577	NEU
nsp15	1	148	V	**1.301**	MIN	V	1.466	MIN
nsp15	1	149	K	**1.301**	NEU	K	-0.689	NEU
nsp15	1	150	G	**1.051**	NEU	G	-0.714	NEU
nsp15	1	151	L	**1.301**	MIN	L	1.027	MIN
nsp15	1	152	T	-0.025	NEU	Q	-1.192	MAX
nsp15	1	153	P	0.490	NEU	P	-0.71	NEU
nsp15	1	154	S	**1.301**	NEU	S	0.182	NEU
nsp15	1	155	K	0.260	MAX	V	-0.004	NEU
nsp15	1	156	G	**1.301**	NEU	G	-0.334	NEU
nsp15	1	157	P	**1.301**	NEU	P	-0.084	NEU
nsp15	1	158	A	-0.084	NEU	K	-1.228	MAX
nsp15	1	159	Q	**1.301**	NEU	Q	-0.401	NEU
nsp15	1	160	A	**1.301**	NEU	A	-0.023	NEU
nsp15	1	161	S	**1.301**	NEU	S	-0.045	NEU

nsp15	1	162	V	**1.301**	MIN	L	1.084	MIN
nsp15	1	163	N	0.301	NEU	N	-0.909	NEU
nsp15	1	164	G	**1.301**	NEU	G	0.222	NEU
nsp15	1	165	V	**1.301**	MIN	V	1.217	MIN
nsp15	1	166	T	0.321	NEU	T	0.72	NEU
nsp15	1	167	L	**1.301**	MIN	L	1.257	MIN
nsp15	1	168	I	**0.887**	NEU	I	0.031	NEU
nsp15	1	169	G	**1.301**	NEU	G	-0.898	NEU
nsp15	1	170	E	0.301	MIN	E	1.801	MIN
nsp15	1	171	S	0.306	MIN	A	-0.496	NEU
nsp15	1	172	V	**1.051**	MIN	V	1.395	MIN
nsp15	1	173	K	**1.051**	MIN	K	1.504	MIN
nsp15	1	174	T	**1.301**	NEU	T	-0.099	NEU
nsp15	1	175	Q	**1.051**	NEU	Q	0.188	NEU
nsp15	1	176	F	**0.651**	MIN	F	0.065	NEU
nsp15	1	177	N	**0.887**	NEU	N	-0.942	NEU
nsp15	1	178	Y	**1.051**	MIN	Y	0.649	NEU
nsp15	1	179	F	**0.563**	MIN	Y	-0.606	NEU
nsp15	1	180	K	**1.301**	MAX	K	-1.394	MAX
nsp15	1	181	K	**1.301**	MAX	K	-1.376	MAX
nsp15	1	182	V	**1.301**	MIN	V	1.476	MIN
nsp15	1	183	D	**1.051**	NEU	D	-0.364	NEU
nsp15	1	184	G	**1.301**	NEU	G	-0.238	NEU
nsp15	1	185	I	**0.757**	MIN	V	0.535	NEU
nsp15	1	186	I	**1.301**	MIN	V	1.13	MIN
nsp15	1	187	Q	**0.651**	NEU	Q	-1.055	MAX
nsp15	1	188	Q	**1.301**	NEU	Q	0.461	NEU
nsp15	1	189	L	**1.301**	MIN	L	1.218	MIN
nsp15	1	190	P	**1.301**	NEU	P	0.174	NEU
nsp15	1	191	E	**1.301**	NEU	E	-0.749	NEU
nsp15	1	192	T	**1.301**	NEU	T	-0.248	NEU
nsp15	1	193	Y	**1.301**	NEU	Y	0.351	NEU
nsp15	1	194	F	**1.051**	NEU	F	-0.471	NEU
nsp15	1	195	T	**1.301**	NEU	T	-0.107	NEU
nsp15	1	196	Q	**1.301**	NEU	Q	-0.716	NEU
nsp15	1	197	S	**1.301**	NEU	S	-0.12	NEU
nsp15	1	198	R	**0.887**	MIN	R	0.972	MIN
nsp15	1	199	D	0.360	NEU	N	0.111	NEU
nsp15	1	200	L	**1.301**	MIN	L	1.241	MIN
nsp15	1	201	E	**0.887**	NEU	Q	-0.514	NEU
nsp15	1	202	D	**0.651**	MIN	E	0.958	MIN
nsp15	1	203	F	**1.301**	NEU	F	0.283	NEU
nsp15	1	204	K	**1.301**	MIN	K	1.702	MIN
nsp15	1	205	P	**1.301**	NEU	P	-0.451	NEU
nsp15	1	206	R	**0.563**	MIN	R	0.555	NEU
nsp15	1	207	S	**1.301**	NEU	S	-0.487	NEU

nsp15	1	208	Q	0.430	MIN	Q	0.169	NEU
nsp15	1	209	M	**1.301**	MIN	M	1.065	MIN
nsp15	1	210	E	**1.301**	NEU	E	-0.801	NEU
nsp15	1	211	T	**0.887**	MIN	I	1.486	MIN
nsp15	1	212	D	**1.301**	MAX	D	-1.466	MAX
nsp15	1	213	F	**1.301**	MIN	F	0.744	NEU
nsp15	1	214	L	**1.301**	MIN	L	1.337	MIN
nsp15	1	215	E	**0.887**	MAX	E	-1.613	MAX
nsp15	1	216	L	**1.301**	NEU	L	-0.125	NEU
nsp15	1	217	A	**1.301**	NEU	A	-0.581	NEU
nsp15	1	218	M	**1.301**	MIN	M	0.856	MIN
nsp15	1	219	D	**1.301**	MAX	D	-1.365	MAX
nsp15	1	220	E	**1.301**	NEU	E	-0.832	NEU
nsp15	1	221	F	**1.301**	MIN	F	0.701	NEU
nsp15	1	222	I	**1.301**	MIN	I	1.573	MIN
nsp15	1	223	Q	**0.563**	NEU	E	-0.968	NEU
nsp15	1	224	R	**0.757**	NEU	R	0.558	NEU
nsp15	1	225	Y	**1.301**	NEU	Y	-0.401	NEU
nsp15	1	226	K	**0.887**	MIN	K	1.034	MIN
nsp15	1	227	L	**1.301**	MIN	L	0.801	MIN
nsp15	1	228	E	**1.301**	NEU	E	-0.218	NEU
nsp15	1	229	G	**0.887**	NEU	G	-0.87	NEU
nsp15	1	230	Y	**0.887**	NEU	Y	-0.967	NEU
nsp15	1	231	A	**1.301**	NEU	A	0.47	NEU
nsp15	1	232	F	**1.301**	MIN	F	0.886	MIN
nsp15	1	233	E	**1.301**	MAX	E	-1.147	MAX
nsp15	1	234	H	**1.051**	NEU	H	-0.914	NEU
nsp15	1	235	I	**1.051**	MIN	I	1.236	MIN
nsp15	1	236	V	**1.301**	MIN	V	1.258	MIN
nsp15	1	237	Y	**1.301**	MAX	Y	-1.748	MAX
nsp15	1	238	G	**1.301**	NEU	G	-0.499	NEU
nsp15	1	239	D	**1.301**	NEU	D	-0.554	NEU
nsp15	1	240	F	**1.301**	NEU	F	-0.526	NEU
nsp15	1	241	S	**1.051**	MIN	S	0.705	NEU
nsp15	1	242	H	**1.301**	NEU	H	-0.069	NEU
nsp15	1	243	G	**1.301**	NEU	S	-0.389	NEU
nsp15	1	244	Q	**1.301**	NEU	Q	0.362	NEU
nsp15	1	245	L	**1.301**	MIN	L	0.822	MIN
nsp15	1	246	G	**1.301**	NEU	G	-0.653	NEU
nsp15	1	247	G	**1.301**	NEU	G	-0.517	NEU
nsp15	1	248	L	**1.301**	MIN	L	1.22	MIN
nsp15	1	249	H	**1.301**	NEU	H	-0.313	NEU
nsp15	1	250	L	**1.301**	MIN	L	1.328	MIN
nsp15	1	251	M	**1.301**	MIN	L	1.389	MIN
nsp15	1	252	I	**1.301**	MIN	I	1.459	MIN
nsp15	1	253	G	**1.301**	NEU	G	-0.465	NEU

nsp15	1	254	L	**1.301**	MIN	L	1.321	MIN
nsp15	1	255	A	**1.301**	NEU	A	0.484	NEU
nsp15	1	256	K	**1.301**	MAX	K	-1.465	MAX
nsp15	1	257	R	**1.051**	NEU	R	-0.676	NEU
nsp15	1	258	S	**1.051**	NEU	F	0.872	MIN
nsp15	1	259	Q	**1.301**	NEU	K	-0.525	NEU
nsp15	1	260	D	**1.301**	MIN	E	1.703	MIN
nsp15	1	261	S	**1.301**	NEU	S	-0.007	NEU
nsp15	1	262	P	**0.651**	MIN	P	0.966	MIN
nsp15	1	263	L	**1.301**	MIN	F	0.915	MIN
nsp15	1	264	K	**0.757**	NEU	E	-1.196	MAX
nsp15	1	265	L	**1.301**	MIN	L	1.209	MIN
nsp15	1	266	E	0.430	NEU	E	-1.455	MAX
nsp15	1	267	D	**0.887**	NEU	D	-0.754	NEU
nsp15	1	268	F	0.064	NEU	F	0.906	MIN
nsp15	1	269	I	**0.757**	MIN	I	0.8	MIN
nsp15	1	270	P	0.490	NEU	P	-0.958	NEU
nsp15	1	271	M	**0.757**	NEU	M	-0.698	NEU
nsp15	1	272	D	**1.051**	NEU	D	-0.648	NEU
nsp15	1	273	S	**1.301**	NEU	S	-0.855	NEU
nsp15	1	274	T	**1.301**	NEU	T	-0.096	NEU
nsp15	1	275	V	**1.301**	MIN	V	1.031	MIN
nsp15	1	276	K	**0.563**	NEU	K	-0.956	NEU
nsp15	1	277	N	**1.301**	NEU	N	-0.894	NEU
nsp15	1	278	Y	**0.651**	MIN	Y	0.631	NEU
nsp15	1	279	F	**1.051**	NEU	F	0.572	NEU
nsp15	1	280	I	**1.301**	MIN	I	1.14	MIN
nsp15	1	281	T	**1.301**	NEU	T	-0.096	NEU
nsp15	1	282	D	**0.757**	MAX	D	-1.134	MAX
nsp15	1	283	A	**1.301**	MAX	A	-1.171	MAX
nsp15	1	284	Q	**0.757**	NEU	Q	0.466	NEU
nsp15	1	285	T	**1.301**	NEU	T	0.336	NEU
nsp15	1	286	G	**1.301**	NEU	G	-0.079	NEU
nsp15	1	287	S	**1.051**	NEU	S	0.222	NEU
nsp15	1	288	S	**1.301**	NEU	S	-0.713	NEU
nsp15	1	289	K	**1.301**	MAX	K	-1.484	MAX
nsp15	1	290	C	**1.301**	MIN	C	1.513	MIN
nsp15	1	291	V	**1.301**	MIN	V	0.727	NEU
nsp15	1	292	C	**1.301**	MIN	C	1.15	MIN
nsp15	1	293	S	**1.301**	NEU	S	-0.518	NEU
nsp15	1	294	V	**1.301**	MIN	V	1.176	MIN
nsp15	1	295	I	**1.301**	MIN	I	1.305	MIN
nsp15	1	296	D	**1.301**	NEU	D	-0.81	NEU
nsp15	1	297	L	**1.301**	MIN	L	1.315	MIN
nsp15	1	298	L	**1.301**	MIN	L	0.927	MIN
nsp15	1	299	L	**1.301**	MIN	L	1.128	MIN

nsp15	1	300	D	**1.301**	MAX	D	-1.315	MAX
nsp15	1	301	D	**1.301**	MAX	D	-1.504	MAX
nsp15	1	302	F	**1.301**	MIN	F	1.124	MIN
nsp15	1	303	V	**1.301**	MIN	V	1.48	MIN
nsp15	1	304	E	**1.301**	MAX	E	-1.352	MAX
nsp15	1	305	I	**1.301**	MIN	I	1.515	MIN
nsp15	1	306	I	**1.301**	MIN	I	1.402	MIN
nsp15	1	307	K	**1.301**	NEU	K	-0.672	NEU
nsp15	1	308	S	**1.301**	NEU	S	-0.841	NEU
nsp15	1	309	Q	**1.301**	NEU	Q	-0.942	NEU
nsp15	1	310	D	0.430	MAX	D	-1.093	MAX
nsp15	1	311	L	**1.301**	MIN	L	1.352	MIN
nsp15	1	312	S	**1.301**	MIN	S	1.002	MIN
nsp15	1	313	V	**1.301**	MIN	V	1.534	MIN
nsp15	1	314	V	0.430	MIN	V	0.676	NEU
nsp15	1	315	S	**1.301**	NEU	S	-0.726	NEU
nsp15	1	316	K	**1.301**	MAX	K	-1.19	MAX
nsp15	1	317	V	**0.887**	NEU	V	0.291	NEU
nsp15	1	318	V	**1.301**	MIN	V	1.386	MIN
nsp15	1	319	K	**0.804**	NEU	K	-0.72	NEU
nsp15	1	320	V	**1.301**	MIN	V	1.186	MIN
nsp15	1	321	T	0.306	MIN	T	0.844	MIN
nsp15	1	322	I	**1.301**	MIN	I	1.369	MIN
nsp15	1	323	D	**1.301**	NEU	D	-0.854	NEU
nsp15	1	324	Y	**1.301**	NEU	Y	0.436	NEU
nsp15	1	325	A	**1.051**	NEU	T	0.065	NEU
nsp15	1	326	E	**1.301**	NEU	E	0.339	NEU
nsp15	1	327	I	**1.301**	MIN	I	1.158	MIN
nsp15	1	328	S	**1.301**	NEU	S	-0.913	NEU
nsp15	1	329	F	**1.301**	MIN	F	0.766	NEU
nsp15	1	330	M	**1.301**	NEU	M	-0.232	NEU
nsp15	1	331	L	**1.301**	MIN	L	1.197	MIN
nsp15	1	332	W	**1.301**	MAX	W	-1.348	MAX
nsp15	1	333	C	**1.301**	MIN	C	1.943	MIN
nsp15	1	334	K	**1.301**	NEU	K	-0.884	NEU
nsp15	1	335	D	**0.804**	NEU	D	-0.667	NEU
nsp15	1	336	G	**1.301**	NEU	G	-0.535	NEU
nsp15	1	337	H	**1.051**	MIN	H	0.962	MIN
nsp15	1	338	V	**1.301**	MIN	V	1.091	MIN
nsp15	1	339	E	**1.301**	NEU	E	-0.833	NEU
nsp15	1	340	T	**1.301**	NEU	T	-0.449	NEU
nsp15	1	341	F	**1.301**	MIN	F	0.959	MIN
nsp15	1	342	Y	**1.301**	NEU	Y	-0.088	NEU
nsp15	1	343	P	**1.301**	NEU	P	-0.939	NEU
nsp15	1	344	K	**1.301**	MAX	K	-1.309	MAX
nsp15	1	345	L	**1.301**	MIN	L	0.855	MIN

nsp16	1	1	S	0.462	NEU	S	-0.214	NEU	
nsp16	1	2	Q	**1.175**	NEU	Q	-0.7	NEU	
nsp16	1	3	D	0.052	NEU	A	-0.86	NEU	
nsp16	1	4	W	0.346	MAX	W	-1.723	MAX	
nsp16	1	5	K	**0.613**	NEU	Q	-0.865	NEU	
nsp16	1	6	P	0.372	NEU	P	-1.204	MAX	
nsp16	1	7	G	**1.329**	NEU	G	-0.494	NEU	
nsp16	1	8	V	0.132	MIN	V	1.311	MIN	
nsp16	1	9	A	**1.329**	NEU	A	-0.032	NEU	
nsp16	1	10	M	**1.175**	MIN	M	0.708	NEU	
nsp16	1	11	P	**0.607**	NEU	P	-0.667	NEU	
nsp16	1	12	N	**1.175**	NEU	N	-0.692	NEU	
nsp16	1	13	L	**1.329**	MIN	L	1.409	MIN	
nsp16	1	14	Y	**0.557**	NEU	Y	-0.963	NEU	
nsp16	1	15	K	0.332	NEU	K	-0.652	NEU	
nsp16	1	16	V	0.434	NEU	M	-0.096	NEU	
nsp16	1	17	Q	0.329	MAX	Q	-1.09	MAX	
nsp16	1	18	N	0.236	NEU	R	0.149	NEU	
nsp16	1	19	M	-0.077	NEU	M	-0.773	NEU	
nsp16	1	20	L	0.251	MIN	L	0.8	MIN	
nsp16	1	21	L	0.332	NEU	L	0.831	MIN	
nsp16	1	22	E	**1.067**	MAX	E	-1.485	MAX	
nsp16	1	23	P	0.329	MIN	K	1.811	MIN	
nsp16	1	24	C	**1.329**	MIN	C	1.414	MIN	
nsp16	1	25	D	0.004	NEU	D	1.274	MIN	
nsp16	1	26	L	**1.175**	NEU	L	0.383	NEU	
nsp16	1	27	H	**1.022**	NEU	Q	-0.364	NEU	
nsp16	1	28	N	0.462	NEU	N	-0.951	NEU	
nsp16	1	29	Y	**1.329**	MAX	Y	-2.597	MAX	
nsp16	1	30	G	**1.329**	NEU	G	0.244	NEU	
nsp16	1	31	Q	0.492	MAX	D	-1.198	MAX	
nsp16	1	32	S	0.346	NEU	S	-0.662	NEU	
nsp16	1	33	A	0.221	MIN	A	0.379	NEU	
nsp16	1	34	R	0.434	NEU	T	-0.796	NEU	
nsp16	1	35	L	0.411	MIN	L	1.326	MIN	
nsp16	1	36	P	**1.175**	NEU	P	-0.739	NEU	
nsp16	1	37	K	**0.676**	NEU	K	-0.964	NEU	
nsp16	1	38	G	**1.329**	NEU	G	-0.07	NEU	
nsp16	1	39	I	0.462	MIN	I	1.741	MIN	
nsp16	1	40	M	0.411	NEU	M	-0.564	NEU	
nsp16	1	41	M	**1.329**	NEU	M	0.141	NEU	
nsp16	1	42	N	**0.976**	NEU	N	-0.716	NEU	
nsp16	1	43	V	**1.329**	MIN	V	1.404	MIN	
nsp16	1	44	A	0.462	MIN	A	0.819	MIN	
nsp16	1	45	K	0.462	MAX	K	-1.633	MAX	
nsp16	1	46	Y	**0.896**	NEU	Y	-0.45	NEU	

nsp16	1	47	T	**1.329**	NEU	T	-0.286	NEU
nsp16	1	48	Q	0.332	NEU	Q	-0.859	NEU
nsp16	1	49	L	**1.329**	MIN	L	1.303	MIN
nsp16	1	50	C	**0.826**	MIN	C	1.415	MIN
nsp16	1	51	Q	**1.067**	NEU	Q	-0.525	NEU
nsp16	1	52	Y	0.234	NEU	Y	0.621	NEU
nsp16	1	53	L	**1.329**	MIN	L	1.372	MIN
nsp16	1	54	N	**1.329**	NEU	N	-0.094	NEU
nsp16	1	55	T	**1.175**	NEU	T	-0.811	NEU
nsp16	1	56	C	**1.175**	MIN	L	1.433	MIN
nsp16	1	57	T	**1.329**	NEU	T	-0.537	NEU
nsp16	1	58	L	**1.329**	MIN	L	1.205	MIN
nsp16	1	59	A	**1.329**	NEU	A	0.241	NEU
nsp16	1	60	V	**1.175**	MIN	V	1.081	MIN
nsp16	1	61	P	**1.329**	MAX	P	-1.368	MAX
nsp16	1	62	A	**1.175**	NEU	Y	-0.234	NEU
nsp16	1	63	N	**1.329**	NEU	N	-0.586	NEU
nsp16	1	64	M	**1.175**	MIN	M	0.92	MIN
nsp16	1	65	R	**1.329**	NEU	R	-0.552	NEU
nsp16	1	66	V	**1.329**	MIN	V	1.157	MIN
nsp16	1	67	I	**1.329**	MIN	I	1.62	MIN
nsp16	1	68	H	**1.329**	NEU	H	-0.374	NEU
nsp16	1	69	F	**1.329**	MIN	F	1.454	MIN
nsp16	1	70	G	**1.329**	NEU	G	-0.067	NEU
nsp16	1	71	A	**1.329**	NEU	A	-0.121	NEU
nsp16	1	72	G	**1.175**	NEU	G	0.046	NEU
nsp16	1	73	S	**1.067**	NEU	S	-0.569	NEU
nsp16	1	74	D	**0.527**	MIN	D	0.745	NEU
nsp16	1	75	K	0.492	MAX	K	-1.283	MAX
nsp16	1	76	G	**1.329**	NEU	G	0.236	NEU
nsp16	1	77	V	**1.329**	MIN	V	1.411	MIN
nsp16	1	78	A	-0.033	NEU	A	-0.777	NEU
nsp16	1	79	P	**0.762**	NEU	P	-0.916	NEU
nsp16	1	80	G	**1.329**	NEU	G	-0.813	NEU
nsp16	1	81	T	**1.329**	NEU	T	-0.449	NEU
nsp16	1	82	A	**0.896**	NEU	A	-0.286	NEU
nsp16	1	83	V	**1.329**	MIN	V	1.177	MIN
nsp16	1	84	L	**1.329**	MIN	L	1.34	MIN
nsp16	1	85	R	0.492	NEU	R	-0.18	NEU
nsp16	1	86	Q	**0.896**	NEU	Q	-0.737	NEU
nsp16	1	87	W	0.358	NEU	W	0.76	NEU
nsp16	1	88	L	**1.329**	MIN	L	1.286	MIN
nsp16	1	89	P	0.411	MAX	P	-0.989	NEU
nsp16	1	90	T	0.042	NEU	T	-0.379	NEU
nsp16	1	91	D	**0.896**	NEU	G	-0.309	NEU
nsp16	1	92	A	**1.329**	NEU	T	-0.254	NEU

nsp16	1	93	L	**1.175**	MIN	L	1.231	MIN
nsp16	1	94	L	**1.329**	MIN	L	1.284	MIN
nsp16	1	95	V	**1.329**	MIN	V	1.166	MIN
nsp16	1	96	D	**1.067**	MAX	D	-1.075	MAX
nsp16	1	97	N	0.372	MAX	S	-0.558	NEU
nsp16	1	98	D	**1.329**	MAX	D	-1.518	MAX
nsp16	1	99	L	**0.654**	NEU	L	-0.267	NEU
nsp16	1	100	N	0.349	NEU	N	-0.214	NEU
nsp16	1	101	D	0.234	NEU	D	-0.713	NEU
nsp16	1	102	F	-0.054	NEU	F	-0.224	NEU
nsp16	1	103	V	0.332	MIN	V	0.506	NEU
nsp16	1	104	S	**1.329**	NEU	S	-0.693	NEU
nsp16	1	105	D	**1.175**	NEU	D	-0.617	NEU
nsp16	1	106	A	0.372	NEU	A	0.396	NEU
nsp16	1	107	D	**1.329**	MAX	D	-1.413	MAX
nsp16	1	108	S	0.329	MIN	S	-0.749	NEU
nsp16	1	109	T	**1.175**	NEU	T	-0.408	NEU
nsp16	1	110	L	**0.896**	MIN	L	1.197	MIN
nsp16	1	111	I	-0.137	NEU	I	1.631	MIN
nsp16	1	112	G	**1.329**	NEU	G	-0.627	NEU
nsp16	1	113	D	**0.826**	MAX	D	-1.22	MAX
nsp16	1	114	C	**1.329**	MIN	C	1.617	MIN
nsp16	1	115	A	-0.241	NEU	A	-1.268	MAX
nsp16	1	116	T	0.372	NEU	T	0.501	NEU
nsp16	1	117	V	**1.329**	MIN	V	1.441	MIN
nsp16	1	118	H	0.404	NEU	H	0.293	NEU
nsp16	1	119	T	0.411	NEU	T	-0.457	NEU
nsp16	1	120	A	0.404	NEU	A	-0.556	NEU
nsp16	1	121	N	**0.745**	NEU	N	-0.738	NEU
nsp16	1	122	K	0.492	NEU	K	-0.542	NEU
nsp16	1	123	W	**1.329**	MIN	W	0.742	NEU
nsp16	1	124	D	**1.175**	MAX	D	-1.072	MAX
nsp16	1	125	L	**1.329**	MIN	L	1.165	MIN
nsp16	1	126	I	**1.329**	MIN	I	1.352	MIN
nsp16	1	127	I	**1.329**	MIN	I	1.574	MIN
nsp16	1	128	S	**1.329**	NEU	S	-0.738	NEU
nsp16	1	129	D	**1.329**	NEU	D	-0.831	NEU
nsp16	1	130	M	**1.329**	MIN	M	0.978	MIN
nsp16	1	131	Y	**0.826**	MAX	Y	-3.116	MAX
nsp16	1	132	D	0.118	MIN	D	1.883	MIN
nsp16	1	133	P	0.319	NEU	P	-1.023	MAX
nsp16	1	134	K	**0.826**	MIN	K	1.262	MIN
nsp16	1	135	T	**1.175**	NEU	T	0.144	NEU
nsp16	1	136	K	0.349	NEU	K	0.429	NEU
nsp16	1	137	N	**0.527**	MIN	N	0.593	NEU
nsp16	1	138	V	**0.826**	NEU	V	-0.281	NEU

nsp16	1	139	T	0.236	NEU	T	0.112	NEU
nsp16	1	140	G	**0.506**	NEU	K	0.77	NEU
nsp16	1	141	E	-0.254	MIN	E	-1.857	MAX
nsp16	1	142	N	0.372	MAX	N	-1.026	MAX
nsp16	1	143	D	0.276	NEU	D	0.159	NEU
nsp16	1	144	S	**1.329**	NEU	S	0.015	NEU
nsp16	1	145	K	**1.067**	MAX	K	-1.575	MAX
nsp16	1	146	E	0.411	MAX	E	-1.294	MAX
nsp16	1	147	G	0.411	NEU	G	-0.277	NEU
nsp16	1	148	F	**1.329**	MIN	F	0.835	MIN
nsp16	1	149	F	**1.175**	MIN	F	0.891	MIN
nsp16	1	150	T	0.492	NEU	T	-0.108	NEU
nsp16	1	151	Y	**1.175**	NEU	Y	-0.025	NEU
nsp16	1	152	L	**1.329**	MIN	I	1.551	MIN
nsp16	1	153	C	**0.527**	MIN	C	1.26	MIN
nsp16	1	154	G	**1.175**	NEU	G	-0.389	NEU
nsp16	1	155	F	**1.329**	MIN	F	1.444	MIN
nsp16	1	156	I	**1.329**	MIN	I	1.219	MIN
nsp16	1	157	K	0.434	MAX	Q	-1.451	MAX
nsp16	1	158	Q	**0.565**	NEU	Q	-0.996	NEU
nsp16	1	159	K	**1.329**	MAX	K	-1.367	MAX
nsp16	1	160	L	**1.329**	MIN	L	1.249	MIN
nsp16	1	161	A	0.462	MIN	A	0.743	NEU
nsp16	1	162	L	**1.329**	MIN	L	1.475	MIN
nsp16	1	163	G	**1.329**	NEU	G	-0.484	NEU
nsp16	1	164	G	**1.329**	NEU	G	-0.566	NEU
nsp16	1	165	S	**1.329**	NEU	S	-0.609	NEU
nsp16	1	166	V	**1.175**	MIN	V	1.329	MIN
nsp16	1	167	A	**1.329**	NEU	A	0.459	NEU
nsp16	1	168	I	**1.329**	MIN	I	1.378	MIN
nsp16	1	169	K	**1.175**	MAX	K	-1.151	MAX
nsp16	1	170	I	**1.329**	MIN	I	1.801	MIN
nsp16	1	171	T	**0.705**	NEU	T	0.341	NEU
nsp16	1	172	E	**1.329**	NEU	E	-0.347	NEU
nsp16	1	173	H	0.211	NEU	H	0.732	NEU
nsp16	1	174	S	**1.329**	NEU	S	-0.592	NEU
nsp16	1	175	W	**0.762**	MAX	W	-1.558	MAX
nsp16	1	176	S	**1.329**	NEU	N	-0.481	NEU
nsp16	1	177	A	**0.976**	NEU	A	-0.58	NEU
nsp16	1	178	D	0.372	NEU	D	-0.8	NEU
nsp16	1	179	L	**1.329**	MIN	L	1.347	MIN
nsp16	1	180	Y	0.170	NEU	Y	-0.613	NEU
nsp16	1	181	K	0.293	NEU	K	-0.568	NEU
nsp16	1	182	L	**1.067**	MIN	L	1.423	MIN
nsp16	1	183	M	0.338	NEU	M	0.782	MIN
nsp16	1	184	G	**1.329**	NEU	G	-0.266	NEU

nsp16	1	185	H	0.042	NEU	H	-0.307	NEU
nsp16	1	186	F	**1.329**	MIN	F	1.031	MIN
nsp16	1	187	A	**0.509**	NEU	A	0.243	NEU
nsp16	1	188	W	**1.175**	NEU	W	0.172	NEU
nsp16	1	189	W	**1.175**	NEU	W	0.126	NEU
nsp16	1	190	T	**1.329**	NEU	T	-0.381	NEU
nsp16	1	191	A	0.372	MIN	A	0.175	NEU
nsp16	1	192	F	**1.329**	MIN	F	1.126	MIN
nsp16	1	193	C	**1.329**	MIN	V	1.275	MIN
nsp16	1	194	T	**1.329**	NEU	T	-0.474	NEU
nsp16	1	195	N	**1.329**	NEU	N	-0.842	NEU
nsp16	1	196	V	0.390	MIN	V	0.964	MIN
nsp16	1	197	N	**1.329**	NEU	N	-0.736	NEU
nsp16	1	198	A	**1.175**	NEU	A	0.091	NEU
nsp16	1	199	S	**1.329**	NEU	S	-0.634	NEU
nsp16	1	200	S	0.434	NEU	S	0.148	NEU
nsp16	1	201	S	**1.329**	NEU	S	-0.192	NEU
nsp16	1	202	E	**1.067**	NEU	E	-0.939	NEU
nsp16	1	203	A	**1.329**	NEU	A	0.377	NEU
nsp16	1	204	F	**1.329**	MIN	F	1.315	MIN
nsp16	1	205	L	**1.329**	MIN	L	1.281	MIN
nsp16	1	206	I	**1.329**	MIN	I	1.369	MIN
nsp16	1	207	G	**1.329**	NEU	G	-0.531	NEU
nsp16	1	208	V	**1.329**	MIN	C	1.573	MIN
nsp16	1	209	N	**0.705**	NEU	N	-0.886	NEU
nsp16	1	210	Y	**0.826**	MIN	Y	0.738	NEU
nsp16	1	211	L	**1.175**	MIN	L	1.117	MIN
nsp16	1	212	G	**1.175**	NEU	G	-0.802	NEU
nsp16	1	213	K	-0.011	NEU	K	-1.447	MAX
nsp16	1	214	-	0.319	MIN	P	-0.905	NEU
nsp16	1	215	K	0.222	NEU	R	-0.504	NEU
nsp16	1	216	E	0.409	NEU	E	-0.521	NEU
nsp16	1	217	Q	0.251	MIN	Q	0.564	NEU
nsp16	1	218	I	**1.329**	MIN	I	1.277	MIN
nsp16	1	219	D	0.434	NEU	D	-0.222	NEU
nsp16	1	220	G	**1.329**	NEU	G	-0.447	NEU
nsp16	1	221	Y	0.199	MAX	Y	-1.08	MAX
nsp16	1	222	A	-0.128	NEU	V	1.453	MIN
nsp16	1	223	M	**0.571**	NEU	M	-0.517	NEU
nsp16	1	224	H	**1.329**	NEU	H	-0.393	NEU
nsp16	1	225	A	0.462	MIN	A	0.673	NEU
nsp16	1	226	N	**0.762**	MAX	N	-1.129	MAX
nsp16	1	227	Y	**1.329**	NEU	Y	-0.564	NEU
nsp16	1	228	I	**1.329**	MIN	I	0.869	MIN
nsp16	1	229	F	**1.329**	NEU	F	-0.672	NEU
nsp16	1	230	W	0.462	MAX	W	-1.828	MAX

nsp16	1	231	R	**1.329**	NEU	R	-0.699	NEU
nsp16	1	232	N	0.358	NEU	N	-0.386	NEU
nsp16	1	233	S	**1.175**	NEU	T	-0.855	NEU
nsp16	1	234	T	**1.329**	NEU	N	-0.853	NEU
nsp16	1	235	P	0.293	NEU	P	-0.119	NEU
nsp16	1	236	M	0.372	NEU	I	1.474	MIN
nsp16	1	237	Q	-0.071	NEU	Q	-0.791	NEU
nsp16	1	238	L	**0.976**	MIN	L	0.853	MIN
nsp16	1	239	S	**1.329**	NEU	S	-0.176	NEU
nsp16	1	240	S	**0.914**	NEU	S	-0.719	NEU
nsp16	1	241	Y	0.329	MAX	Y	-0.677	NEU
nsp16	1	242	S	**1.329**	NEU	S	-0.615	NEU
nsp16	1	243	L	**1.329**	MIN	L	0.929	MIN
nsp16	1	244	F	0.349	MIN	F	1.131	MIN
nsp16	1	245	D	**0.762**	NEU	D	0.459	NEU
nsp16	1	246	L	0.358	NEU	M	-0.436	NEU
nsp16	1	247	S	0.462	MIN	S	1.197	MIN
nsp16	1	248	K	**0.896**	MIN	K	1.129	MIN
nsp16	1	249	F	**1.329**	MIN	F	0.947	MIN
nsp16	1	250	P	0.390	NEU	P	0.636	NEU
nsp16	1	251	L	**1.329**	MIN	L	1.431	MIN
nsp16	1	252	K	**0.527**	NEU	K	0.44	NEU
nsp16	1	253	L	**0.607**	MIN	L	1.197	MIN
nsp16	1	254	K	0.390	MAX	R	-0.718	NEU
nsp16	1	255	G	**1.329**	NEU	G	-0.386	NEU
nsp16	1	256	T	**1.329**	NEU	T	-0.337	NEU
nsp16	1	257	P	0.346	NEU	A	0.308	NEU
nsp16	1	258	V	**1.329**	MIN	V	1.125	MIN
nsp16	1	259	M	**1.329**	MIN	M	0.912	MIN
nsp16	1	260	S	**0.824**	NEU	S	0.369	NEU
nsp16	1	261	L	**1.329**	MIN	L	1.273	MIN
nsp16	1	262	K	-0.147	NEU	K	-1.4	MAX
nsp16	1	263	E	**0.762**	NEU	E	-0.548	NEU
nsp16	1	264	S	0.332	MIN	G	-0.481	NEU
nsp16	1	265	Q	**1.175**	NEU	Q	-0.312	NEU
nsp16	1	266	I	**1.329**	MIN	I	1.967	MIN
nsp16	1	267	N	**0.565**	MAX	N	-1.343	MAX
nsp16	1	268	E	0.332	MAX	D	-0.949	NEU
nsp16	1	269	L	**1.175**	NEU	M	-0.135	NEU
nsp16	1	270	V	**1.329**	MIN	I	1.613	MIN
nsp16	1	271	L	0.174	MIN	L	1.302	MIN
nsp16	1	272	S	**0.896**	NEU	S	-0.261	NEU
nsp16	1	273	L	**1.329**	MIN	L	1.306	MIN
nsp16	1	274	L	**1.329**	MIN	L	1.182	MIN
nsp16	1	275	S	**0.976**	NEU	S	-0.181	NEU
nsp16	1	276	K	0.157	NEU	K	-1.818	MAX

nsp16	1	277	G	**1.329**	NEU	G	-0.323	NEU
nsp16	1	278	R	**1.175**	NEU	R	-0.659	NEU
nsp16	1	279	L	**1.329**	MIN	L	1.359	MIN
nsp16	1	280	L	**1.329**	MIN	I	1.422	MIN
nsp16	1	281	I	**1.329**	MIN	I	1.586	MIN
nsp16	1	282	R	**1.329**	NEU	R	-0.209	NEU
nsp16	1	283	D	0.329	MAX	E	-1	MAX
nsp16	1	284	N	0.372	NEU	N	0.491	NEU
nsp16	1	285	N	**0.754**	NEU	N	0.106	NEU
nsp16	1	286	-	0.083	NEU	R	1.762	MIN
nsp16	1	287	L	**1.316**	MIN	V	1.098	MIN
nsp16	1	288	V	**0.620**	NEU	V	-0.221	NEU
nsp16	1	289	V	**1.316**	MIN	I	1.778	MIN
nsp16	1	290	S	**1.316**	NEU	S	-0.61	NEU
nsp16	1	291	S	**0.927**	NEU	S	-0.702	NEU
nsp16	1	292	D	0.494	NEU	D	0.34	NEU
nsp16	1	293	V	**1.172**	MIN	V	1.195	MIN
nsp16	1	294	L	0.204	NEU	L	1.454	MIN
nsp16	1	295	V	0.383	MIN	V	1.503	MIN
nsp16	1	296	N	-0.011	NEU	N	-0.989	NEU
nsp16	1	297	N	0.382	NEU	N	-0.676	NEU
S_protein	4	1	Q	**0.818**	NEU	Q	-0.567	NEU
S_protein	4	2	C	-0.267	NEU	C	3.138	MIN
S_protein	4	3	G	0.329	NEU	V	1.23	MIN
S_protein	4	4	T	**0.543**	NEU	N	1.506	MIN
S_protein	4	5	F	**1.311**	MIN	L	1.409	MIN
S_protein	4	6	S	**0.736**	NEU	T	0.28	NEU
S_protein	4	7	D	0.186	NEU	T	0.134	NEU
S_protein	4	8	K	-0.180	MIN	R	1.704	MIN
S_protein	4	9	P	**0.621**	NEU	T	0.989	MIN
S_protein	4	10	Q	-0.175	NEU	Q	0.634	NEU
S_protein	4	11	P	**0.831**	NEU	L	-0.829	NEU
S_protein	4	12	K	0.133	NEU	P	0.887	MIN
S_protein	4	13	L	0.312	NEU	P	0.043	NEU
S_protein	4	14	T	0.306	NEU	A	-1.001	MAX
S_protein	4	15	Q	**0.524**	NEU	Y	-1.029	MAX
S_protein	4	16	V	0.220	MIN	T	-0.436	NEU
S_protein	4	17	S	**0.736**	NEU	N	-0.653	NEU
S_protein	4	18	S	**1.311**	NEU	S	-0.773	NEU
S_protein	4	19	S	**1.095**	NEU	F	1.595	MIN
S_protein	4	20	R	0.276	NEU	T	-0.486	NEU
S_protein	4	21	R	**1.095**	NEU	R	-0.586	NEU
S_protein	4	22	G	**1.311**	NEU	G	-0.511	NEU
S_protein	4	23	V	**1.311**	MIN	V	1.36	MIN
S_protein	4	24	Y	0.312	MAX	Y	-1.893	MAX
S_protein	4	25	Y	**0.732**	NEU	Y	0.729	NEU

S_protein	4	26	P	0.133	NEU	P	-0.643	NEU
S_protein	4	27	D	**1.095**	NEU	D	-0.21	NEU
S_protein	4	28	D	**1.095**	NEU	K	-0.679	NEU
S_protein	4	29	I	**0.648**	MIN	V	-0.138	NEU
S_protein	4	30	F	-0.105	MIN	F	-1.085	MAX
S_protein	4	31	R	**1.311**	NEU	R	-0.566	NEU
S_protein	4	32	S	**1.311**	NEU	S	-0.442	NEU
S_protein	4	33	D	0.220	NEU	S	-0.216	NEU
S_protein	4	34	V	**0.514**	MIN	V	1.114	MIN
S_protein	4	35	L	**0.576**	MIN	L	0.867	MIN
S_protein	4	36	H	**0.576**	NEU	H	0.162	NEU
S_protein	4	37	L	**1.095**	MIN	S	-0.479	NEU
S_protein	4	38	T	**0.576**	NEU	T	-0.16	NEU
S_protein	4	39	Q	**1.311**	NEU	Q	0.109	NEU
S_protein	4	40	D	**0.831**	NEU	D	-0.997	NEU
S_protein	4	41	Y	0.386	MIN	L	0.911	MIN
S_protein	4	42	F	**1.311**	MIN	F	1.22	MIN
S_protein	4	43	L	**1.311**	MIN	L	0.987	MIN
S_protein	4	44	P	**0.949**	MAX	P	-1.73	MAX
S_protein	4	45	F	**1.311**	MIN	F	1.224	MIN
S_protein	4	46	N	0.064	NEU	F	0.944	MIN
S_protein	4	47	S	**1.311**	NEU	S	-0.389	NEU
S_protein	4	48	N	0.481	NEU	N	-0.44	NEU
S_protein	4	49	V	**1.311**	MIN	V	1.455	MIN
S_protein	4	50	T	**1.095**	NEU	T	-0.548	NEU
S_protein	4	51	Q	**0.732**	NEU	W	0.625	NEU
S_protein	4	52	Y	0.333	NEU	F	1.423	MIN
S_protein	4	53	F	0.019	MIN	H	-0.74	NEU
S_protein	4	54	S	**1.311**	NEU	A	0.548	NEU
S_protein	4	55	L	**0.524**	MIN	I	0.965	MIN
S_protein	4	56	N	0.441	NEU	H	-0.68	NEU
S_protein	4	57	V	0.064	NEU	V	0.333	NEU
S_protein	4	58	D	0.026	NEU	S	0.29	NEU
S_protein	4	60	-	**0.971**	NEU	T	0.183	NEU
S_protein	4	61	-	-0.178	NEU	N	-0.477	NEU
S_protein	4	62	-	0.048	NEU	G	0.159	NEU
S_protein	4	63	-	-0.250	NEU	T	0.816	MIN
S_protein	4	64	-	-0.071	MIN	K	-1.149	MAX
S_protein	4	65	Y	0.428	MAX	R	-0.353	NEU
S_protein	4	66	F	**0.736**	MIN	F	1.283	MIN
S_protein	4	67	D	0.418	NEU	D	-0.736	NEU
S_protein	4	68	N	**0.514**	MAX	N	-1	MAX
S_protein	4	69	P	0.418	NEU	P	-1.089	MAX
S_protein	4	70	I	0.386	MIN	V	1.125	MIN
S_protein	4	71	L	**1.311**	MIN	L	1.062	MIN
S_protein	4	72	P	**0.648**	MAX	P	-1.858	MAX

S_protein	4	73	F	**1.311**	MIN	F	1.223	MIN
S_protein	4	74	G	0.312	NEU	N	-0.782	NEU
S_protein	4	75	D	0.333	NEU	D	-1.102	MAX
S_protein	4	76	G	**1.311**	NEU	G	-0.389	NEU
S_protein	4	77	V	**1.311**	MIN	V	1.259	MIN
S_protein	4	78	Y	**0.831**	MIN	Y	0.617	NEU
S_protein	4	79	F	**1.311**	MIN	F	1.332	MIN
S_protein	4	80	A	**0.949**	NEU	A	0.534	NEU
S_protein	4	81	A	**1.095**	NEU	S	-0.67	NEU
S_protein	4	82	T	**0.949**	NEU	T	-0.359	NEU
S_protein	4	83	E	**0.514**	NEU	E	-0.768	NEU
S_protein	4	84	K	**0.514**	MAX	K	-0.212	NEU
S_protein	4	85	S	**1.311**	NEU	S	-0.643	NEU
S_protein	4	86	N	0.382	NEU	N	-0.233	NEU
S_protein	4	87	V	**1.095**	MIN	I	1.261	MIN
S_protein	4	88	I	**1.311**	MIN	I	1.206	MIN
S_protein	4	89	R	**1.311**	NEU	R	-0.8	NEU
S_protein	4	90	G	**1.311**	NEU	G	-0.438	NEU
S_protein	4	91	W	**0.831**	MIN	W	0.661	NEU
S_protein	4	92	I	**1.311**	MIN	I	1.328	MIN
S_protein	4	93	F	**1.311**	MIN	F	1.416	MIN
S_protein	4	94	G	**1.311**	NEU	G	-0.694	NEU
S_protein	4	95	S	**1.311**	NEU	T	-0.004	NEU
S_protein	4	96	T	**0.949**	MIN	T	1.286	MIN
S_protein	4	97	M	**1.311**	MIN	L	1.009	MIN
S_protein	4	98	D	**0.576**	MAX	D	0.003	NEU
S_protein	4	99	N	**0.648**	NEU	S	-0.322	NEU
S_protein	4	100	T	0.078	MIN	K	0.481	NEU
S_protein	4	101	T	**1.311**	NEU	T	0.341	NEU
S_protein	4	102	Q	**1.311**	NEU	Q	-0.857	NEU
S_protein	4	103	S	**1.311**	NEU	S	-0.821	NEU
S_protein	4	104	A	0.354	NEU	L	1.279	MIN
S_protein	4	105	I	**1.311**	MIN	L	1.091	MIN
S_protein	4	106	I	**1.311**	MIN	I	1.894	MIN
S_protein	4	107	V	**1.095**	MIN	V	1.214	MIN
S_protein	4	108	N	0.461	NEU	N	-0.862	NEU
S_protein	4	109	N	0.319	MAX	N	-1.193	MAX
S_protein	4	110	S	**1.095**	NEU	A	0.186	NEU
S_protein	4	111	T	0.461	NEU	T	0.064	NEU
S_protein	4	112	H	**1.311**	NEU	N	-0.831	NEU
S_protein	4	113	I	**0.831**	MIN	V	1.657	MIN
S_protein	4	114	V	**0.949**	MIN	V	1.363	MIN
S_protein	4	115	I	**1.311**	MIN	I	1.75	MIN
S_protein	4	116	R	0.418	NEU	K	-1.185	MAX
S_protein	4	117	V	0.461	MIN	V	1.27	MIN
S_protein	4	118	C	**1.311**	MIN	C	1.764	MIN

S_protein	4	119	N	0.306	NEU	E	-1.512	MAX
S_protein	4	120	F	**1.311**	MIN	F	1.371	MIN
S_protein	4	121	N	-0.030	NEU	Q	0.108	NEU
S_protein	4	122	L	**1.311**	MIN	F	1.396	MIN
S_protein	4	123	C	**1.311**	MIN	C	2.119	MIN
S_protein	4	124	K	0.418	MAX	N	0.033	NEU
S_protein	4	125	E	0.371	MAX	D	-0.796	NEU
S_protein	4	126	P	**0.732**	NEU	P	-0.93	NEU
S_protein	4	127	M	**1.095**	MIN	F	1.518	MIN
S_protein	4	128	F	0.461	MIN	L	1.223	MIN
S_protein	4	129	T	0.371	NEU	G	-0.635	NEU
S_protein	4	130	V	**1.095**	MIN	V	1.231	MIN
S_protein	4	131	S	0.198	NEU	Y	-1.839	MAX
S_protein	4	132	K	-0.180	NEU	Y	-2.29	MAX
S_protein	4	133	G	0.421	NEU	H	0.582	NEU
S_protein	4	134	T	**0.880**	NEU	K	-0.609	NEU
S_protein	4	135	Q	**0.594**	NEU	N	1.085	MIN
S_protein	4	136	T	-0.047	NEU	N	-0.638	NEU
S_protein	4	137	K	**1.095**	NEU	K	-0.221	NEU
S_protein	4	138	S	**1.095**	NEU	S	-0.342	NEU
S_protein	4	139	W	-0.050	NEU	W	-2.036	MAX
S_protein	4	146	V	**1.311**	MIN	V	1.243	MIN
S_protein	4	147	Y	**0.576**	MIN	Y	0.496	NEU
S_protein	4	148	Q	0.026	NEU	S	-0.164	NEU
S_protein	4	149	N	**1.095**	NEU	S	-0.315	NEU
S_protein	4	150	A	**0.736**	NEU	A	-0.222	NEU
S_protein	4	151	F	0.187	MIN	N	-0.427	NEU
S_protein	4	152	N	0.461	NEU	N	-0.83	NEU
S_protein	4	153	C	**1.311**	MIN	C	1.963	MIN
S_protein	4	154	T	**1.311**	NEU	T	-0.082	NEU
S_protein	4	155	Y	-0.061	NEU	F	0.99	MIN
S_protein	4	156	D	0.094	NEU	E	-0.774	NEU
S_protein	4	157	R	-0.061	NEU	Y	-0.598	NEU
S_protein	4	158	V	0.461	MIN	V	0.696	NEU
S_protein	4	159	S	0.465	NEU	S	0.621	NEU
S_protein	4	160	K	0.312	MAX	Q	-1.315	MAX
S_protein	4	161	S	0.276	NEU	P	-1.423	MAX
S_protein	4	162	F	-0.127	NEU	F	0.053	NEU
S_protein	4	163	Q	0.333	NEU	L	1.006	MIN
S_protein	4	164	L	0.354	NEU	M	-0.204	NEU
S_protein	4	165	D	0.312	NEU	D	0.496	NEU
S_protein	4	166	T	0.162	MIN	L	1.185	MIN
S_protein	4	167	A	0.371	NEU	E	-0.54	NEU
S_protein	4	168	E	-0.030	NEU	G	-0.251	NEU
S_protein	4	169	K	**0.576**	MIN	K	1.777	MIN
S_protein	4	170	T	0.133	NEU	Q	-0.817	NEU

S_protein	4	171	G	**0.621**	NEU	G	-0.647	NEU
S_protein	4	172	N	-0.030	NEU	N	-0.987	NEU
S_protein	4	173	F	**1.095**	MIN	F	0.868	MIN
S_protein	4	174	K	0.333	NEU	K	0.307	NEU
S_protein	4	175	H	**0.949**	NEU	N	0.257	NEU
S_protein	4	176	L	**1.311**	MIN	L	1.098	MIN
S_protein	4	177	R	**0.732**	NEU	R	-0.141	NEU
S_protein	4	178	E	**0.732**	MAX	E	-0.896	NEU
S_protein	4	179	F	0.382	MIN	F	1.21	MIN
S_protein	4	180	V	**1.311**	MIN	V	1.168	MIN
S_protein	4	181	F	**1.311**	MIN	F	1.438	MIN
S_protein	4	182	K	**0.576**	MAX	K	-1.173	MAX
S_protein	4	183	N	**0.732**	NEU	N	-0.917	NEU
S_protein	4	184	K	0.026	NEU	I	1.767	MIN
S_protein	4	185	D	**0.831**	MAX	D	-1.397	MAX
S_protein	4	186	G	**1.311**	NEU	G	-0.55	NEU
S_protein	4	187	F	0.461	MIN	Y	-0.776	NEU
S_protein	4	188	L	**1.311**	MIN	F	1.019	MIN
S_protein	4	189	Y	**0.514**	NEU	K	-1.119	MAX
S_protein	4	190	V	**1.311**	MIN	I	1.725	MIN
S_protein	4	191	Y	0.220	NEU	Y	0.627	NEU
S_protein	4	192	S	**0.648**	NEU	S	-0.692	NEU
S_protein	4	193	G	0.369	NEU	K	-1.112	MAX
S_protein	4	194	Y	0.319	NEU	H	-0.505	NEU
S_protein	4	195	T	**1.095**	NEU	T	-0.135	NEU
S_protein	4	196	P	0.333	NEU	P	1.211	MIN
S_protein	4	197	I	**0.949**	MIN	I	1.314	MIN
S_protein	4	198	N	**0.648**	MIN	N	1.119	MIN
S_protein	4	199	L	**1.095**	MIN	L	1.315	MIN
S_protein	4	200	V	0.276	NEU	V	-0.168	NEU
S_protein	4	201	R	0.441	NEU	R	-1.085	MAX
S_protein	4	202	G	**0.648**	NEU	D	-0.92	NEU
S_protein	4	203	L	**1.311**	MIN	L	1.277	MIN
S_protein	4	204	P	**1.095**	NEU	P	-1.002	MAX
S_protein	4	205	S	0.481	NEU	Q	-0.797	NEU
S_protein	4	206	G	**1.095**	NEU	G	-0.214	NEU
S_protein	4	207	F	0.354	NEU	F	0.386	NEU
S_protein	4	208	S	**0.949**	NEU	S	-0.611	NEU
S_protein	4	209	V	0.461	MIN	A	0.382	NEU
S_protein	4	210	L	**1.311**	MIN	L	1.105	MIN
S_protein	4	211	K	0.418	MAX	E	-0.808	NEU
S_protein	4	212	P	**1.311**	NEU	P	0.069	NEU
S_protein	4	213	I	**1.311**	MIN	L	1.413	MIN
S_protein	4	214	L	**0.732**	MIN	V	1.536	MIN
S_protein	4	215	K	0.312	NEU	D	-0.055	NEU
S_protein	4	216	L	**1.311**	MIN	L	1.286	MIN

S_protein	4	217	P	0.382	NEU	P	0.999	MIN
S_protein	4	218	L	**1.311**	MIN	I	1.316	MIN
S_protein	4	219	G	**1.311**	NEU	G	-0.113	NEU
S_protein	4	220	I	**1.311**	MIN	I	1.446	MIN
S_protein	4	221	N	0.333	MIN	N	0.154	NEU
S_protein	4	222	I	**1.311**	MIN	I	1.305	MIN
S_protein	4	223	T	**1.311**	NEU	T	0.042	NEU
S_protein	4	224	S	0.064	NEU	R	-0.81	NEU
S_protein	4	225	F	**0.831**	MIN	F	1.351	MIN
S_protein	4	226	R	**0.514**	NEU	Q	-0.801	NEU
S_protein	4	227	V	0.461	MIN	T	-0.476	NEU
S_protein	4	228	V	**1.311**	MIN	L	1.49	MIN
S_protein	4	229	M	**0.949**	MIN	L	1.207	MIN
S_protein	4	230	T	**0.514**	NEU	A	0.542	NEU
S_protein	4	231	M	0.461	MIN	L	1.327	MIN
S_protein	4	232	F	**0.514**	MIN	H	-0.015	NEU
S_protein	4	233	S	**0.524**	NEU	R	-0.324	NEU
S_protein	4	240	P	-0.171	NEU	D	0.348	NEU
S_protein	4	241	T	0.133	NEU	S	0.244	NEU
S_protein	4	242	T	0.153	NEU	S	1.144	MIN
S_protein	4	243	S	**0.804**	NEU	S	0.16	NEU
S_protein	4	244	N	0.332	NEU	G	0.054	NEU
S_protein	4	245	F	0.153	MIN	W	0.58	NEU
S_protein	4	246	L	0.144	NEU	T	-0.168	NEU
S_protein	4	247	T	0.033	NEU	A	0.44	NEU
S_protein	4	248	E	0.129	NEU	G	-0.451	NEU
S_protein	4	249	A	**0.880**	NEU	A	-0.864	NEU
S_protein	4	250	A	**1.095**	NEU	A	0.127	NEU
S_protein	4	251	A	**0.949**	NEU	A	-0.047	NEU
S_protein	4	252	Y	0.319	MIN	Y	0.716	NEU
S_protein	4	253	F	**0.949**	MIN	Y	0.416	NEU
S_protein	4	254	V	**1.311**	MIN	V	1.401	MIN
S_protein	4	255	G	**1.311**	NEU	G	-0.395	NEU
S_protein	4	256	Y	**1.311**	NEU	Y	-0.824	NEU
S_protein	4	257	L	**1.311**	MIN	L	0.913	MIN
S_protein	4	258	K	**0.648**	MAX	Q	-0.893	NEU
S_protein	4	259	P	0.418	NEU	P	-0.182	NEU
S_protein	4	260	T	**0.648**	NEU	R	-0.502	NEU
S_protein	4	261	T	**1.095**	NEU	T	-0.33	NEU
S_protein	4	262	F	**1.311**	MIN	F	1.416	MIN
S_protein	4	263	M	**0.576**	MIN	L	0.981	MIN
S_protein	4	264	L	**1.311**	MIN	L	1.278	MIN
S_protein	4	265	K	**0.949**	NEU	K	-1.083	MAX
S_protein	4	266	F	**1.311**	MIN	Y	0.839	MIN
S_protein	4	267	N	0.261	NEU	N	0.466	NEU
S_protein	4	268	E	0.198	MAX	E	-0.813	NEU

S_protein	4	269	N	0.312	NEU	N	1.309	MIN
S_protein	4	270	G	**1.311**	NEU	G	-0.195	NEU
S_protein	4	271	T	**1.311**	NEU	T	-0.36	NEU
S_protein	4	272	I	**0.949**	MIN	I	1.665	MIN
S_protein	4	273	T	0.354	NEU	T	0.091	NEU
S_protein	4	274	D	**0.831**	NEU	D	-0.988	NEU
S_protein	4	275	A	**0.576**	MIN	A	0.865	MIN
S_protein	4	276	V	**1.311**	MIN	V	1.112	MIN
S_protein	4	277	D	**0.514**	MAX	D	-1.053	MAX
S_protein	4	278	C	**1.095**	MIN	C	2.363	MIN
S_protein	4	279	S	**1.095**	NEU	A	0.421	NEU
S_protein	4	280	Q	**0.831**	NEU	L	1.06	MIN
S_protein	4	281	N	**0.949**	MAX	D	-1.499	MAX
S_protein	4	282	P	**1.311**	NEU	P	-0.97	NEU
S_protein	4	283	L	**1.311**	MIN	L	1.316	MIN
S_protein	4	284	A	**1.095**	NEU	S	-0.189	NEU
S_protein	4	285	E	**1.311**	NEU	E	-0.809	NEU
S_protein	4	286	L	**0.831**	MIN	T	-0.154	NEU
S_protein	4	287	K	0.319	MAX	K	-1.141	MAX
S_protein	4	288	C	**1.311**	MIN	C	1.854	MIN
S_protein	4	289	T	**1.311**	NEU	T	0.28	NEU
S_protein	4	290	L	**0.648**	MIN	L	1.157	MIN
S_protein	4	291	K	0.354	NEU	K	-0.695	NEU
S_protein	4	292	S	**1.095**	NEU	S	-0.979	NEU
S_protein	4	293	F	0.418	NEU	F	0.214	NEU
S_protein	4	294	N	**1.095**	MIN	T	0.833	MIN
S_protein	4	295	V	**1.311**	MIN	V	1.395	MIN
S_protein	4	296	D	0.306	NEU	E	-1.524	MAX
S_protein	4	297	K	**0.732**	NEU	K	-0.813	NEU
S_protein	4	298	G	**1.311**	NEU	G	-0.439	NEU
S_protein	4	299	I	**1.311**	MIN	I	1.321	MIN
S_protein	4	300	Y	**1.311**	NEU	Y	-0.612	NEU
S_protein	4	301	Q	**1.311**	NEU	Q	-0.428	NEU
S_protein	4	302	T	**1.311**	NEU	T	0.029	NEU
S_protein	4	303	S	**1.311**	NEU	S	-0.606	NEU
S_protein	4	304	N	**1.311**	NEU	N	-0.772	NEU
S_protein	4	305	F	**1.311**	MIN	F	1.305	MIN
S_protein	4	306	R	**0.831**	NEU	R	0.43	NEU
S_protein	4	307	V	**1.311**	MIN	V	1.21	MIN
S_protein	4	308	S	-0.005	NEU	Q	-0.052	NEU
S_protein	4	309	P	**0.514**	NEU	P	-0.977	NEU
S_protein	4	310	T	**0.949**	NEU	T	-0.669	NEU
S_protein	4	311	Q	0.481	NEU	E	-0.861	NEU
S_protein	4	312	E	**0.732**	MAX	S	-0.648	NEU
S_protein	4	313	V	**1.311**	MIN	I	1.267	MIN
S_protein	4	314	V	**1.095**	MIN	V	1.089	MIN

S_protein	4	315	R	**1.311**	NEU	R	-0.532	NEU
S_protein	4	316	F	**1.311**	MIN	F	1.386	MIN
S_protein	4	317	P	**1.095**	NEU	P	-0.148	NEU
S_protein	4	318	N	**1.311**	NEU	N	-0.047	NEU
S_protein	4	319	I	**1.311**	MIN	I	0.918	MIN
S_protein	4	320	T	**1.311**	NEU	T	-0.486	NEU
S_protein	4	321	N	**0.949**	NEU	N	-0.817	NEU
S_protein	4	322	L	0.354	MIN	L	1.408	MIN
S_protein	4	323	C	**1.311**	MIN	C	1.537	MIN
S_protein	4	324	P	**0.648**	NEU	P	-0.877	NEU
S_protein	4	325	F	**1.311**	MIN	F	1.177	MIN
S_protein	4	326	D	**0.648**	NEU	G	-0.41	NEU
S_protein	4	327	E	0.319	NEU	E	-0.776	NEU
S_protein	4	328	V	**1.311**	MIN	V	1.141	MIN
S_protein	4	329	F	**1.311**	MIN	F	1.261	MIN
S_protein	4	330	N	0.312	MIN	N	-0.52	NEU
S_protein	4	331	A	**0.831**	NEU	A	0.493	NEU
S_protein	4	332	T	**1.311**	NEU	T	-0.125	NEU
S_protein	4	333	R	0.319	MIN	R	0.325	NEU
S_protein	4	334	F	**1.311**	MIN	F	1.258	MIN
S_protein	4	335	P	0.382	NEU	A	0.47	NEU
S_protein	4	336	S	**1.311**	NEU	S	-0.678	NEU
S_protein	4	337	V	**1.311**	MIN	V	1.292	MIN
S_protein	4	338	Y	**0.949**	NEU	Y	0.099	NEU
S_protein	4	339	A	**1.311**	NEU	A	0.368	NEU
S_protein	4	340	W	0.418	MIN	W	0.546	NEU
S_protein	4	341	E	**0.648**	NEU	N	-0.737	NEU
S_protein	4	342	R	**1.311**	MIN	R	1.304	MIN
S_protein	4	343	T	**0.648**	NEU	K	-0.598	NEU
S_protein	4	344	K	0.354	NEU	R	1.626	MIN
S_protein	4	345	I	**1.311**	MIN	I	1.143	MIN
S_protein	4	346	S	**1.311**	NEU	S	-0.206	NEU
S_protein	4	347	D	0.319	MAX	N	-0.794	NEU
S_protein	4	348	C	**1.311**	MIN	C	1.556	MIN
S_protein	4	349	V	**1.311**	MIN	V	1.113	MIN
S_protein	4	350	A	**1.311**	NEU	A	0.18	NEU
S_protein	4	351	D	**1.311**	MAX	D	-1.057	MAX
S_protein	4	352	Y	**1.311**	NEU	Y	-0.428	NEU
S_protein	4	353	T	0.354	NEU	S	1.405	MIN
S_protein	4	354	V	0.382	MIN	V	0.594	NEU
S_protein	4	355	L	**1.311**	MIN	L	1.209	MIN
S_protein	4	356	Y	**1.311**	NEU	Y	0.504	NEU
S_protein	4	357	N	**0.648**	NEU	N	-0.669	NEU
S_protein	4	358	S	**1.311**	NEU	S	-0.532	NEU
S_protein	4	359	T	**1.311**	NEU	A	-0.341	NEU
S_protein	4	360	S	**0.831**	NEU	S	-0.32	NEU

S_protein	4	361	F	**1.311**	MIN	F	1.184	MIN
S_protein	4	362	S	**1.311**	NEU	S	-0.574	NEU
S_protein	4	363	T	**1.311**	NEU	T	-0.378	NEU
S_protein	4	364	F	**1.311**	MIN	F	1.679	MIN
S_protein	4	365	K	**0.949**	MAX	K	-1.243	MAX
S_protein	4	366	C	**1.311**	MIN	C	1.553	MIN
S_protein	4	367	Y	**1.311**	MIN	Y	1.015	MIN
S_protein	4	368	G	**1.311**	NEU	G	-0.7	NEU
S_protein	4	369	V	**1.311**	MIN	V	1.322	MIN
S_protein	4	370	S	**1.311**	NEU	S	-0.292	NEU
S_protein	4	371	P	**0.648**	NEU	P	-0.56	NEU
S_protein	4	372	S	**1.095**	NEU	T	-0.68	NEU
S_protein	4	373	K	**0.732**	NEU	K	-0.92	NEU
S_protein	4	374	L	**1.311**	MIN	L	1.241	MIN
S_protein	4	375	I	0.319	MIN	N	-0.582	NEU
S_protein	4	376	D	**1.095**	MIN	D	0.528	NEU
S_protein	4	377	L	**1.311**	MIN	L	0.992	MIN
S_protein	4	378	C	**1.311**	MIN	C	2.304	MIN
S_protein	4	379	F	**1.311**	MIN	F	1.231	MIN
S_protein	4	380	T	**1.311**	NEU	T	-0.19	NEU
S_protein	4	381	S	**1.095**	NEU	N	-0.998	NEU
S_protein	4	382	V	**1.311**	MIN	V	1.204	MIN
S_protein	4	383	Y	**0.576**	NEU	Y	-0.907	NEU
S_protein	4	384	A	**1.311**	MIN	A	0.781	MIN
S_protein	4	385	D	**0.576**	MAX	D	-0.898	NEU
S_protein	4	386	T	**0.831**	NEU	S	-0.643	NEU
S_protein	4	387	F	**1.311**	MIN	F	1.189	MIN
S_protein	4	388	L	**1.311**	MIN	V	1.359	MIN
S_protein	4	389	I	**1.311**	MIN	I	1.087	MIN
S_protein	4	390	R	0.354	NEU	R	-0.149	NEU
S_protein	4	391	G	**0.514**	NEU	G	-0.403	NEU
S_protein	4	392	S	**0.831**	NEU	D	0.334	NEU
S_protein	4	393	E	**1.311**	NEU	E	-0.834	NEU
S_protein	4	394	V	**1.311**	MIN	V	1.391	MIN
S_protein	4	395	R	**1.311**	MIN	R	1.395	MIN
S_protein	4	396	Q	0.312	MAX	Q	-0.943	NEU
S_protein	4	397	V	**1.311**	MIN	I	1.692	MIN
S_protein	4	398	A	**1.311**	NEU	A	-0.264	NEU
S_protein	4	399	P	**0.831**	MAX	P	-1.034	MAX
S_protein	4	400	G	**1.095**	NEU	G	0.078	NEU
S_protein	4	401	Q	0.312	NEU	Q	-0.936	NEU
S_protein	4	402	T	**1.311**	MIN	T	0.727	NEU
S_protein	4	403	G	**1.311**	NEU	G	-0.48	NEU
S_protein	4	404	V	0.461	NEU	K	0.441	NEU
S_protein	4	405	I	**1.311**	MIN	I	1.509	MIN
S_protein	4	406	A	**1.311**	NEU	A	-0.354	NEU

S_protein	4	407	D	**0.514**	NEU	D	0.589	NEU
S_protein	4	408	Y	0.319	MAX	Y	-1.229	MAX
S_protein	4	409	N	0.333	MAX	N	-1.062	MAX
S_protein	4	410	Y	**1.311**	NEU	Y	-0.779	NEU
S_protein	4	411	K	0.312	MAX	K	-0.644	NEU
S_protein	4	412	L	**1.311**	MIN	L	0.97	MIN
S_protein	4	413	P	0.312	NEU	P	0.764	NEU
S_protein	4	414	D	**0.949**	NEU	D	-0.701	NEU
S_protein	4	415	D	**1.311**	NEU	D	-0.246	NEU
S_protein	4	416	F	**1.311**	MIN	F	1.362	MIN
S_protein	4	417	T	**0.514**	NEU	T	-0.297	NEU
S_protein	4	418	G	**1.311**	NEU	G	-0.324	NEU
S_protein	4	419	C	**1.311**	MIN	C	1.049	MIN
S_protein	4	420	V	**1.311**	MIN	V	1.329	MIN
S_protein	4	421	I	**1.311**	MIN	I	1.218	MIN
S_protein	4	422	A	0.418	NEU	A	0.391	NEU
S_protein	4	423	W	**0.514**	MIN	W	0.561	NEU
S_protein	4	424	N	**1.311**	NEU	N	-0.272	NEU
S_protein	4	425	T	**1.311**	NEU	S	-0.702	NEU
S_protein	4	426	A	**0.736**	NEU	N	0.007	NEU
S_protein	4	427	K	0.129	MIN	N	0.366	NEU
S_protein	4	428	Q	**0.880**	NEU	L	-0.127	NEU
S_protein	4	429	D	**0.514**	NEU	D	-0.491	NEU
S_protein	4	430	A	**1.095**	NEU	S	-0.271	NEU
S_protein	4	431	G	**0.880**	NEU	K	-0.813	NEU
S_protein	4	432	-	-0.079	NEU	V	-0.135	NEU
S_protein	4	433	-	**0.790**	NEU	G	0.187	NEU
S_protein	4	434	-	**1.217**	NEU	G	0.199	NEU
S_protein	4	435	-	**1.217**	NEU	N	-0.208	NEU
S_protein	4	436	-	0.254	NEU	Y	-0.495	NEU
S_protein	4	437	N	**0.648**	NEU	N	-0.157	NEU
S_protein	4	438	Y	0.319	NEU	Y	0.576	NEU
S_protein	4	439	Y	-0.227	MAX	L	0.869	MIN
S_protein	4	440	Y	0.354	NEU	Y	0.534	NEU
S_protein	4	441	R	**1.311**	NEU	R	-0.862	NEU
S_protein	4	442	S	0.386	NEU	L	1.208	MIN
S_protein	4	443	H	0.220	NEU	F	-0.341	NEU
S_protein	4	444	R	**0.831**	MIN	R	1.072	MIN
S_protein	4	445	K	-0.216	MIN	K	0.531	NEU
S_protein	4	446	T	**0.949**	NEU	S	0.067	NEU
S_protein	4	447	K	**0.949**	MIN	N	0.42	NEU
S_protein	4	448	L	**0.831**	NEU	L	-0.036	NEU
S_protein	4	449	K	0.386	NEU	K	-0.011	NEU
S_protein	4	450	P	**1.311**	NEU	P	-0.476	NEU
S_protein	4	451	F	**1.311**	MAX	F	-1.291	MAX
S_protein	4	452	E	**0.732**	MIN	E	0.171	NEU

S_protein	4	453	R	**1.311**	NEU	R	-0.385	NEU
S_protein	4	454	D	0.461	NEU	D	-0.967	NEU
S_protein	4	455	L	**1.311**	MIN	I	1.385	MIN
S_protein	4	456	S	0.354	MIN	S	0.805	MIN
S_protein	4	457	S	**0.949**	NEU	T	-0.292	NEU
S_protein	4	458	D	0.033	NEU	E	0.075	NEU
S_protein	4	459	E	0.418	MIN	I	1.559	MIN
S_protein	4	460	-	-0.210	MIN	Y	-1.945	MAX
S_protein	4	461	-	0.052	MIN	Q	-1.177	MAX
S_protein	4	462	-	**0.859**	NEU	A	0.166	NEU
S_protein	4	463	-	0.254	MIN	G	-0.754	NEU
S_protein	4	464	-	0.471	NEU	S	0.678	NEU
S_protein	4	465	-	**0.859**	NEU	T	-0.73	NEU
S_protein	4	466	-	0.289	MIN	P	0.792	MIN
S_protein	4	467	-	**0.859**	MIN	C	-0.214	NEU
S_protein	4	468	-	0.011	MAX	N	-1.032	MAX
S_protein	4	469	-	0.471	NEU	G	-0.603	NEU
S_protein	4	471	-	-0.064	NEU	E	-1.541	MAX
S_protein	4	472	-	0.254	NEU	G	-1.293	MAX
S_protein	4	473	-	0.279	NEU	F	0.976	MIN
S_protein	4	474	N	0.354	NEU	N	-0.538	NEU
S_protein	4	475	G	0.333	MIN	C	0.72	NEU
S_protein	4	476	V	0.144	NEU	Y	0.084	NEU
S_protein	4	477	R	0.094	MIN	F	0.967	MIN
S_protein	4	478	T	0.319	NEU	P	-1.055	MAX
S_protein	4	479	L	**0.648**	MIN	L	1.219	MIN
S_protein	4	480	S	**0.949**	NEU	Q	-1.128	MAX
S_protein	4	481	T	**1.311**	NEU	S	-0.596	NEU
S_protein	4	482	Y	**0.648**	NEU	Y	-1.035	MAX
S_protein	4	483	D	0.037	NEU	G	0.799	MIN
S_protein	4	484	F	0.319	MIN	F	0.532	NEU
S_protein	4	485	Y	-0.061	MAX	Q	-0.641	NEU
S_protein	4	486	P	0.064	NEU	P	-1.385	MAX
S_protein	4	487	T	0.064	NEU	T	0.867	MIN
S_protein	4	488	V	0.129	MIN	N	-0.585	NEU
S_protein	4	489	P	0.461	NEU	G	0.291	NEU
S_protein	4	490	V	0.333	MIN	V	0.05	NEU
S_protein	4	491	G	-0.163	MIN	G	0.245	NEU
S_protein	4	492	Y	0.382	NEU	Y	-0.492	NEU
S_protein	4	493	Q	**0.732**	NEU	Q	-0.701	NEU
S_protein	4	494	A	0.019	MIN	P	-1.326	MAX
S_protein	4	495	T	0.333	NEU	Y	0.78	MIN
S_protein	4	496	R	**1.311**	NEU	R	-0.707	NEU
S_protein	4	497	V	**1.311**	MIN	V	1.304	MIN
S_protein	4	498	V	**1.311**	MIN	V	1.321	MIN
S_protein	4	499	V	**1.311**	MIN	V	1.429	MIN

S_protein	4	500	L	**1.311**	MIN	L	1.394	MIN
S_protein	4	501	S	**1.311**	NEU	S	-0.599	NEU
S_protein	4	502	F	**1.311**	MIN	F	1.345	MIN
S_protein	4	503	E	**1.095**	MAX	E	-0.932	NEU
S_protein	4	504	L	**1.311**	MIN	L	1.024	MIN
S_protein	4	505	L	**1.311**	MIN	L	1.098	MIN
S_protein	4	506	N	0.198	NEU	H	0.319	NEU
S_protein	4	507	A	0.333	MAX	A	-1.017	MAX
S_protein	4	508	P	**0.736**	NEU	P	0.613	NEU
S_protein	4	509	A	**1.311**	NEU	A	0.234	NEU
S_protein	4	510	T	**1.311**	NEU	T	-0.333	NEU
S_protein	4	511	V	**1.311**	MIN	V	1.298	MIN
S_protein	4	512	C	**1.311**	MIN	C	1.575	MIN
S_protein	4	513	G	**1.311**	NEU	G	-0.466	NEU
S_protein	4	514	P	**0.831**	MAX	P	-0.949	NEU
S_protein	4	515	K	0.312	MAX	K	-1.153	MAX
S_protein	4	516	L	**0.576**	MIN	K	-0.311	NEU
S_protein	4	517	S	**1.311**	NEU	S	-0.683	NEU
S_protein	4	518	T	**1.311**	NEU	T	-0.028	NEU
S_protein	4	519	D	**0.514**	NEU	N	-1.344	MAX
S_protein	4	520	L	**1.311**	MIN	L	1.422	MIN
S_protein	4	521	V	**1.311**	MIN	V	0.985	MIN
S_protein	4	522	K	**0.514**	MAX	K	-1.033	MAX
S_protein	4	523	N	**0.576**	MAX	N	-1.255	MAX
S_protein	4	524	Q	**0.648**	NEU	K	-1.262	MAX
S_protein	4	525	C	**1.311**	MIN	C	2.825	MIN
S_protein	4	526	V	**1.311**	MIN	V	1.106	MIN
S_protein	4	527	N	**0.831**	NEU	N	-0.658	NEU
S_protein	4	528	F	**1.311**	MIN	F	0.917	MIN
S_protein	4	529	N	**0.949**	NEU	N	-0.899	NEU
S_protein	4	530	F	**1.311**	MIN	F	1.507	MIN
S_protein	4	531	N	**0.831**	MAX	N	-1.051	MAX
S_protein	4	532	G	**1.311**	NEU	G	-0.416	NEU
S_protein	4	533	L	**1.311**	MIN	L	1.137	MIN
S_protein	4	534	K	**1.095**	NEU	T	-0.029	NEU
S_protein	4	535	G	**1.311**	NEU	G	-0.47	NEU
S_protein	4	536	T	**1.095**	NEU	T	-0.815	NEU
S_protein	4	537	G	**1.311**	NEU	G	-0.157	NEU
S_protein	4	538	V	**1.311**	MIN	V	1.249	MIN
S_protein	4	539	L	**1.311**	MIN	L	1.222	MIN
S_protein	4	540	T	**1.311**	NEU	T	0.024	NEU
S_protein	4	541	P	**1.095**	NEU	E	-0.234	NEU
S_protein	4	542	S	**1.311**	NEU	S	-0.89	NEU
S_protein	4	543	S	**1.311**	NEU	N	0.189	NEU
S_protein	4	544	K	**1.311**	NEU	K	-0.508	NEU
S_protein	4	545	R	0.382	MIN	K	0.365	NEU

S_protein	4	546	F	**1.311**	MIN	F	1.179	MIN
S_protein	4	547	Q	**0.949**	NEU	L	1.292	MIN
S_protein	4	548	S	0.354	MIN	P	1.296	MIN
S_protein	4	549	F	0.461	NEU	F	0.889	MIN
S_protein	4	550	Q	**1.311**	NEU	Q	-0.367	NEU
S_protein	4	551	Q	**1.095**	MAX	Q	-1.219	MAX
S_protein	4	552	F	**1.311**	MIN	F	0.898	MIN
S_protein	4	553	G	**1.311**	NEU	G	-0.347	NEU
S_protein	4	554	R	**1.095**	MIN	R	1.145	MIN
S_protein	4	555	D	**1.095**	MAX	D	-0.988	NEU
S_protein	4	556	V	**0.648**	NEU	I	-0.152	NEU
S_protein	4	557	S	**1.311**	NEU	A	-0.544	NEU
S_protein	4	558	D	**1.311**	MIN	D	1.591	MIN
S_protein	4	559	F	**1.311**	NEU	T	-0.108	NEU
S_protein	4	560	T	**1.095**	NEU	T	-0.257	NEU
S_protein	4	561	D	**1.095**	NEU	D	-0.348	NEU
S_protein	4	562	S	**1.311**	NEU	A	0.285	NEU
S_protein	4	563	V	**1.311**	MIN	V	1.279	MIN
S_protein	4	564	R	**1.311**	NEU	R	-0.166	NEU
S_protein	4	565	D	0.382	NEU	D	-1.019	MAX
S_protein	4	566	P	**1.311**	MAX	P	-1.059	MAX
S_protein	4	567	Q	**1.095**	NEU	Q	0.202	NEU
S_protein	4	568	T	**1.095**	MIN	T	0.733	NEU
S_protein	4	569	L	**1.095**	NEU	L	-0.518	NEU
S_protein	4	570	E	0.418	NEU	E	0.087	NEU
S_protein	4	571	I	0.461	MIN	I	1.929	MIN
S_protein	4	572	L	**1.311**	MIN	L	1.182	MIN
S_protein	4	573	D	**1.311**	NEU	D	-0.711	NEU
S_protein	4	574	I	**1.311**	MIN	I	1.468	MIN
S_protein	4	575	S	**1.311**	NEU	T	0.067	NEU
S_protein	4	576	P	**0.949**	NEU	P	-1.052	MAX
S_protein	4	577	C	**1.311**	MIN	C	2.004	MIN
S_protein	4	578	S	**1.311**	NEU	S	-0.579	NEU
S_protein	4	579	F	**0.831**	MIN	F	1.881	MIN
S_protein	4	580	G	**1.311**	NEU	G	-0.329	NEU
S_protein	4	581	G	**1.311**	NEU	G	-0.222	NEU
S_protein	4	582	V	**1.311**	MIN	V	1.414	MIN
S_protein	4	583	S	**1.311**	NEU	S	-0.88	NEU
S_protein	4	584	V	**1.311**	MIN	V	1.419	MIN
S_protein	4	585	I	**1.311**	MIN	I	1.292	MIN
S_protein	4	586	T	**1.311**	NEU	T	-0.306	NEU
S_protein	4	587	P	**1.311**	NEU	P	-0.485	NEU
S_protein	4	588	G	**1.311**	NEU	G	-0.856	NEU
S_protein	4	589	T	**1.095**	NEU	T	0.459	NEU
S_protein	4	590	N	0.333	MIN	N	0.526	NEU
S_protein	4	591	A	0.162	MAX	T	0.473	NEU

S_protein	4	592	S	**1.311**	NEU	S	-0.67	NEU
S_protein	4	593	S	**0.621**	NEU	N	-1.049	MAX
S_protein	4	594	E	**0.949**	NEU	Q	-1.005	MAX
S_protein	4	595	V	**1.311**	MIN	V	1.3	MIN
S_protein	4	596	A	**1.311**	NEU	A	-0.295	NEU
S_protein	4	597	V	**1.311**	MIN	V	1.31	MIN
S_protein	4	598	L	**1.311**	MIN	L	1.213	MIN
S_protein	4	599	Y	**1.311**	NEU	Y	-0.003	NEU
S_protein	4	600	Q	**1.311**	NEU	Q	-0.797	NEU
S_protein	4	601	D	**1.311**	NEU	D	0.194	NEU
S_protein	4	602	V	**1.311**	MIN	V	1.264	MIN
S_protein	4	603	N	**1.095**	NEU	N	-1.012	MAX
S_protein	4	604	C	**1.311**	MIN	C	1.679	MIN
S_protein	4	605	T	0.312	NEU	T	-1.362	MAX
S_protein	4	606	D	0.312	NEU	E	-1.47	MAX
S_protein	4	607	V	**1.311**	MIN	V	1.476	MIN
S_protein	4	608	P	**0.514**	MAX	P	-1.098	MAX
S_protein	4	609	T	**0.514**	NEU	V	1.814	MIN
S_protein	4	610	A	**0.576**	MIN	A	0.539	NEU
S_protein	4	611	I	**1.311**	MIN	I	1.863	MIN
S_protein	4	612	H	0.333	MIN	H	0.42	NEU
S_protein	4	613	A	**0.648**	NEU	A	0.523	NEU
S_protein	4	614	D	**0.621**	NEU	D	0.389	NEU
S_protein	4	615	Q	**0.949**	NEU	Q	-0.512	NEU
S_protein	4	616	L	**1.311**	MIN	L	1.225	MIN
S_protein	4	617	T	**1.311**	NEU	T	0.394	NEU
S_protein	4	618	P	**0.514**	MAX	P	-1.09	MAX
S_protein	4	619	A	0.198	NEU	T	0.189	NEU
S_protein	4	620	W	**1.095**	MIN	W	0.946	MIN
S_protein	4	621	R	**0.732**	NEU	R	-0.817	NEU
S_protein	4	622	V	**0.949**	MIN	V	1.119	MIN
S_protein	4	623	Y	**0.621**	NEU	Y	-0.655	NEU
S_protein	4	624	S	**0.949**	NEU	S	-0.337	NEU
S_protein	4	625	T	**0.831**	NEU	T	-0.415	NEU
S_protein	4	626	G	**0.621**	NEU	G	0.385	NEU
S_protein	4	627	V	-0.175	NEU	S	-0.54	NEU
S_protein	4	628	N	0.133	NEU	N	-0.94	NEU
S_protein	4	629	V	**1.095**	MIN	V	1.454	MIN
S_protein	4	630	F	**1.095**	MIN	F	1.59	MIN
S_protein	4	631	Q	**1.311**	MAX	Q	-1.7	MAX
S_protein	4	632	T	**1.311**	NEU	T	-0.441	NEU
S_protein	4	633	Q	**0.949**	NEU	R	1.383	MIN
S_protein	4	634	A	**1.311**	MIN	A	0.59	NEU
S_protein	4	635	G	**1.311**	NEU	G	-0.432	NEU
S_protein	4	636	C	**1.311**	MIN	C	0.839	MIN
S_protein	4	637	L	**1.311**	MIN	L	1.27	MIN

S_protein	4	638	I	**1.311**	MIN	I	1.982	MIN
S_protein	4	639	G	**1.311**	NEU	G	-0.596	NEU
S_protein	4	640	A	**1.311**	NEU	A	0.388	NEU
S_protein	4	641	E	**0.831**	MAX	E	-1.22	MAX
S_protein	4	642	H	**1.095**	NEU	H	-0.286	NEU
S_protein	4	643	V	**0.736**	MIN	V	0.944	MIN
S_protein	4	644	N	**0.514**	NEU	N	0.218	NEU
S_protein	4	645	A	**1.095**	NEU	N	-0.814	NEU
S_protein	4	646	S	**1.311**	NEU	S	-0.7	NEU
S_protein	4	647	Y	**1.311**	MIN	Y	0.611	NEU
S_protein	4	648	E	0.461	MAX	E	-1.183	MAX
S_protein	4	649	C	**1.311**	MIN	C	1.339	MIN
S_protein	4	650	D	0.312	NEU	D	0.22	NEU
S_protein	4	651	I	**1.311**	MIN	I	1.153	MIN
S_protein	4	652	P	**1.311**	NEU	P	-0.471	NEU
S_protein	4	653	I	**1.311**	MIN	I	1.555	MIN
S_protein	4	654	G	**1.311**	NEU	G	-0.352	NEU
S_protein	4	655	A	**1.311**	MIN	A	0.953	MIN
S_protein	4	656	G	**1.311**	NEU	G	-0.276	NEU
S_protein	4	657	I	**1.311**	MIN	I	1.509	MIN
S_protein	4	658	C	**1.311**	MIN	C	0.807	MIN
S_protein	4	659	A	**0.949**	MIN	A	0.636	NEU
S_protein	4	660	S	**1.311**	NEU	S	-0.912	NEU
S_protein	4	661	Y	0.133	MAX	Y	0.298	NEU
S_protein	4	662	H	**1.095**	NEU	Q	-0.768	NEU
S_protein	4	663	T	**1.087**	NEU	T	0.231	NEU
S_protein	4	664	A	0.498	NEU	Q	-0.18	NEU
S_protein	4	665	S	**1.095**	NEU	T	0.641	NEU
S_protein	4	666	T	0.261	NEU	N	1.115	MIN
S_protein	4	671	L	0.133	NEU	A	-1.273	MAX
S_protein	4	672	R	0.261	NEU	R	-1.229	MAX
S_protein	4	673	S	0.319	MIN	S	0.375	NEU
S_protein	4	674	T	**0.648**	NEU	V	-0.553	NEU
S_protein	4	675	G	0.481	NEU	A	-0.916	NEU
S_protein	4	676	Q	**0.880**	NEU	S	-0.167	NEU
S_protein	4	677	K	**1.095**	NEU	Q	-0.091	NEU
S_protein	4	678	S	**1.311**	NEU	S	-0.115	NEU
S_protein	4	679	I	**1.311**	MIN	I	1.305	MIN
S_protein	4	680	V	**1.311**	MIN	I	0.645	NEU
S_protein	4	681	A	**1.095**	NEU	A	0.521	NEU
S_protein	4	682	Y	**1.095**	MIN	Y	0.926	MIN
S_protein	4	683	T	**1.311**	NEU	T	-0.735	NEU
S_protein	4	684	M	**1.095**	MIN	M	0.708	NEU
S_protein	4	685	S	**1.311**	NEU	S	-0.521	NEU
S_protein	4	686	L	**1.311**	MIN	L	1.063	MIN
S_protein	4	687	G	**1.311**	NEU	G	-0.172	NEU

S_protein	4	688	A	**0.949**	NEU	A	-0.45	NEU
S_protein	4	689	E	0.382	MAX	E	-1.099	MAX
S_protein	4	690	N	0.418	MAX	N	-1.202	MAX
S_protein	4	691	S	**0.514**	NEU	S	0.334	NEU
S_protein	4	692	I	0.382	MIN	V	1.001	MIN
S_protein	4	693	A	0.333	NEU	A	-1.364	MAX
S_protein	4	694	Y	**1.311**	MAX	Y	-2.493	MAX
S_protein	4	695	A	0.064	NEU	S	0.642	NEU
S_protein	4	696	N	**1.311**	NEU	N	-0.646	NEU
S_protein	4	697	N	0.319	MAX	N	-0.676	NEU
S_protein	4	698	S	**1.095**	NEU	S	0.175	NEU
S_protein	4	699	I	**1.311**	MIN	I	1.791	MIN
S_protein	4	700	A	**0.732**	NEU	A	-0.599	NEU
S_protein	4	701	I	**1.311**	MIN	I	1.107	MIN
S_protein	4	702	P	**1.311**	MAX	P	-1.261	MAX
S_protein	4	703	T	**0.949**	NEU	T	-0.281	NEU
S_protein	4	704	N	**1.311**	NEU	N	-0.623	NEU
S_protein	4	705	F	**1.311**	MIN	F	1.436	MIN
S_protein	4	706	S	**1.311**	NEU	T	0.072	NEU
S_protein	4	707	I	**1.311**	MIN	I	1.344	MIN
S_protein	4	708	S	**1.311**	NEU	S	-0.638	NEU
S_protein	4	709	V	**1.311**	MIN	V	1.182	MIN
S_protein	4	710	T	**1.311**	NEU	T	-0.069	NEU
S_protein	4	711	T	**1.311**	NEU	T	-0.312	NEU
S_protein	4	712	E	**1.311**	MAX	E	-1.301	MAX
S_protein	4	713	V	**1.311**	MIN	I	1.372	MIN
S_protein	4	714	M	**1.311**	MIN	L	1.231	MIN
S_protein	4	715	P	**1.311**	MAX	P	-1.297	MAX
S_protein	4	716	V	**1.311**	MIN	V	1.213	MIN
S_protein	4	717	S	**1.311**	NEU	S	-0.424	NEU
S_protein	4	718	M	**1.095**	MIN	M	0.643	NEU
S_protein	4	719	A	**1.311**	NEU	T	-0.255	NEU
S_protein	4	720	K	**1.095**	NEU	K	0.269	NEU
S_protein	4	721	T	**1.311**	NEU	T	-0.147	NEU
S_protein	4	722	S	**1.311**	NEU	S	-0.778	NEU
S_protein	4	723	V	**1.311**	MIN	V	1.067	MIN
S_protein	4	724	D	**1.311**	NEU	D	-0.222	NEU
S_protein	4	725	C	**1.311**	MIN	C	1.261	MIN
S_protein	4	726	T	**1.311**	NEU	T	-0.785	NEU
S_protein	4	727	M	**1.311**	MAX	M	-1.245	MAX
S_protein	4	728	Y	**0.514**	NEU	Y	0.388	NEU
S_protein	4	729	I	**1.311**	MIN	I	1.948	MIN
S_protein	4	730	C	**1.311**	MIN	C	1.025	MIN
S_protein	4	731	G	**1.311**	NEU	G	-0.284	NEU
S_protein	4	732	D	**0.831**	NEU	D	0.408	NEU
S_protein	4	733	S	**1.311**	NEU	S	-0.075	NEU

S_protein	4	734	T	-0.204	NEU	T	-0.898	NEU
S_protein	4	735	E	**1.311**	MAX	E	-1.242	MAX
S_protein	4	736	C	**1.311**	MIN	C	1.076	MIN
S_protein	4	737	S	**1.311**	NEU	S	-0.46	NEU
S_protein	4	738	N	0.418	NEU	N	-0.944	NEU
S_protein	4	739	L	**1.311**	MIN	L	1.049	MIN
S_protein	4	740	L	**1.311**	MIN	L	1.362	MIN
S_protein	4	741	L	**1.311**	MIN	L	1.684	MIN
S_protein	4	742	Q	**1.311**	NEU	Q	-0.822	NEU
S_protein	4	743	Y	**1.311**	NEU	Y	0.267	NEU
S_protein	4	744	G	**1.095**	MAX	G	-1.031	MAX
S_protein	4	745	S	**1.311**	NEU	S	-0.059	NEU
S_protein	4	746	F	**1.311**	MIN	F	0.992	MIN
S_protein	4	747	C	**1.311**	MIN	C	1.943	MIN
S_protein	4	748	T	**1.311**	NEU	T	-0.342	NEU
S_protein	4	749	Q	**1.311**	NEU	Q	-0.099	NEU
S_protein	4	750	L	**1.311**	MIN	L	1.405	MIN
S_protein	4	751	N	0.312	NEU	N	-1.041	MAX
S_protein	4	752	R	**1.311**	NEU	R	-0.49	NEU
S_protein	4	753	A	0.461	NEU	A	0.43	NEU
S_protein	4	754	L	**1.311**	MIN	L	1.336	MIN
S_protein	4	755	S	**1.311**	NEU	T	-0.043	NEU
S_protein	4	756	G	**1.311**	NEU	G	-0.743	NEU
S_protein	4	757	I	**1.311**	MIN	I	1.531	MIN
S_protein	4	758	A	0.461	NEU	A	0.548	NEU
S_protein	4	759	V	0.418	NEU	V	0.2	NEU
S_protein	4	760	E	**0.514**	NEU	E	-0.47	NEU
S_protein	4	761	Q	**0.831**	NEU	Q	-0.783	NEU
S_protein	4	762	D	**0.831**	NEU	D	-0.635	NEU
S_protein	4	763	K	**0.514**	NEU	K	0.459	NEU
S_protein	4	764	N	**0.648**	MAX	N	-1.044	MAX
S_protein	4	765	T	**1.311**	NEU	T	-0.153	NEU
S_protein	4	766	Q	**0.831**	MAX	Q	-1.131	MAX
S_protein	4	767	E	**1.311**	NEU	E	-0.467	NEU
S_protein	4	768	V	**1.311**	MIN	V	1.444	MIN
S_protein	4	769	F	**1.311**	MIN	F	0.957	MIN
S_protein	4	770	A	**1.311**	NEU	A	0.136	NEU
S_protein	4	771	Q	0.354	MAX	Q	-0.928	NEU
S_protein	4	772	V	**0.831**	MIN	V	1.506	MIN
S_protein	4	773	K	**1.311**	NEU	K	-0.369	NEU
S_protein	4	774	Q	**0.831**	MAX	Q	-1.147	MAX
S_protein	4	775	M	**0.648**	NEU	I	1.817	MIN
S_protein	4	776	Y	**0.732**	NEU	Y	-1.14	MAX
S_protein	4	777	K	0.312	MIN	K	0.786	MIN
S_protein	4	778	T	**1.311**	NEU	T	-0.129	NEU
S_protein	4	779	P	**1.311**	NEU	P	-0.679	NEU

S_protein	4	780	A	**0.732**	NEU	P	0.739	NEU
S_protein	4	781	I	**0.949**	NEU	I	0.195	NEU
S_protein	4	782	K	**1.311**	NEU	K	-0.748	NEU
S_protein	4	783	D	**0.949**	MAX	D	-1.195	MAX
S_protein	4	784	F	**1.311**	MIN	F	1.346	MIN
S_protein	4	785	G	**1.311**	NEU	G	-0.616	NEU
S_protein	4	786	G	**1.311**	NEU	G	-0.09	NEU
S_protein	4	787	F	**1.311**	MIN	F	1.197	MIN
S_protein	4	788	N	**0.732**	MIN	N	0.566	NEU
S_protein	4	789	F	**1.311**	MIN	F	1.248	MIN
S_protein	4	790	S	**1.311**	NEU	S	-0.706	NEU
S_protein	4	791	Q	**1.311**	NEU	Q	-0.86	NEU
S_protein	4	792	I	**1.311**	MIN	I	1.386	MIN
S_protein	4	793	L	**1.311**	MIN	L	0.874	MIN
S_protein	4	794	P	**1.311**	MAX	P	-1.605	MAX
S_protein	4	795	D	0.333	MIN	D	1.101	MIN
S_protein	4	796	P	**0.949**	NEU	P	0.376	NEU
S_protein	4	797	S	0.064	MIN	S	1.292	MIN
S_protein	4	798	K	**1.095**	MIN	K	0.642	NEU
S_protein	4	799	P	**1.095**	MIN	P	1.163	MIN
S_protein	4	800	T	**1.311**	NEU	S	0.17	NEU
S_protein	4	801	K	**0.831**	MIN	K	1.127	MIN
S_protein	4	802	R	**1.311**	MIN	R	1.123	MIN
S_protein	4	803	S	**1.311**	NEU	S	-0.928	NEU
S_protein	4	804	F	**1.311**	NEU	F	-0.945	NEU
S_protein	4	805	I	**1.311**	MIN	I	1.344	MIN
S_protein	4	806	E	**1.311**	MAX	E	-1.225	MAX
S_protein	4	807	D	**1.311**	NEU	D	-0.414	NEU
S_protein	4	808	L	**1.311**	MIN	L	0.579	NEU
S_protein	4	809	L	**1.311**	MIN	L	1.357	MIN
S_protein	4	810	F	**0.576**	NEU	F	-0.458	NEU
S_protein	4	811	N	**1.311**	NEU	N	-0.623	NEU
S_protein	4	812	K	**0.949**	MAX	K	-1.057	MAX
S_protein	4	813	V	**1.311**	MIN	V	1.302	MIN
S_protein	4	814	T	**1.311**	NEU	T	0.323	NEU
S_protein	4	815	L	**1.311**	MIN	L	1.104	MIN
S_protein	4	816	A	0.133	NEU	A	0.288	NEU
S_protein	4	817	D	-0.240	MIN	D	-1.273	MAX
S_protein	4	818	A	0.418	NEU	A	0.412	NEU
S_protein	4	819	G	**1.311**	NEU	G	-0.321	NEU
S_protein	4	820	F	**1.095**	MIN	F	0.717	NEU
S_protein	4	821	M	0.382	NEU	I	1.78	MIN
S_protein	4	822	K	**0.831**	NEU	K	-0.992	NEU
S_protein	4	823	Q	**0.831**	MAX	Q	-1.149	MAX
S_protein	4	824	Y	0.261	MIN	Y	-1.804	MAX
S_protein	4	825	G	**0.949**	NEU	G	-0.383	NEU

S_protein	4	826	E	0.276	NEU	D	-0.158	NEU
S_protein	4	827	C	**1.311**	MIN	C	3.029	MIN
S_protein	4	828	L	**0.576**	MIN	L	0.371	NEU
S_protein	4	829	G	**1.095**	NEU	G	-0.981	NEU
S_protein	4	830	D	-0.047	MIN	D	-1.064	MAX
S_protein	4	831	I	**0.648**	MIN	I	0.53	NEU
S_protein	4	832	N	**0.524**	MIN	A	-1.027	MAX
S_protein	4	833	A	**1.311**	NEU	A	-0.654	NEU
S_protein	4	834	R	**1.095**	NEU	R	-0.373	NEU
S_protein	4	835	D	**0.949**	NEU	D	-0.915	NEU
S_protein	4	836	L	**1.095**	MIN	L	0.412	NEU
S_protein	4	837	I	**1.311**	MIN	I	1.676	MIN
S_protein	4	838	C	**1.311**	MIN	C	3.017	MIN
S_protein	4	839	A	**1.095**	NEU	A	0.307	NEU
S_protein	4	840	Q	**1.311**	NEU	Q	-0.869	NEU
S_protein	4	841	K	**1.311**	MAX	K	-1.257	MAX
S_protein	4	842	F	**1.311**	MIN	F	1.29	MIN
S_protein	4	843	N	**1.311**	MAX	N	-1.107	MAX
S_protein	4	844	G	**0.648**	NEU	G	-0.809	NEU
S_protein	4	845	L	**1.311**	MIN	L	1.228	MIN
S_protein	4	846	T	**1.311**	NEU	T	0.074	NEU
S_protein	4	847	V	**1.311**	MIN	V	1.288	MIN
S_protein	4	848	L	**1.311**	MIN	L	1.253	MIN
S_protein	4	849	P	**0.732**	NEU	P	-0.448	NEU
S_protein	4	850	P	**0.732**	NEU	P	-0.82	NEU
S_protein	4	851	L	**1.311**	NEU	L	-0.107	NEU
S_protein	4	852	L	**1.311**	MIN	L	1.137	MIN
S_protein	4	853	T	**1.311**	NEU	T	-0.085	NEU
S_protein	4	854	D	**1.311**	NEU	D	-0.767	NEU
S_protein	4	855	E	**0.736**	NEU	E	1.367	MIN
S_protein	4	856	M	**1.311**	NEU	M	0.39	NEU
S_protein	4	857	I	**1.311**	MIN	I	1.472	MIN
S_protein	4	858	A	**0.949**	MIN	A	0.429	NEU
S_protein	4	859	A	**1.311**	NEU	Q	-0.808	NEU
S_protein	4	860	Y	**1.311**	NEU	Y	0.204	NEU
S_protein	4	861	T	**1.311**	NEU	T	-0.361	NEU
S_protein	4	862	A	**0.949**	MIN	S	-0.679	NEU
S_protein	4	863	A	**0.732**	NEU	A	0.39	NEU
S_protein	4	864	L	**1.311**	MIN	L	1.159	MIN
S_protein	4	865	V	**1.311**	MIN	L	1.106	MIN
S_protein	4	866	S	**1.311**	NEU	A	0.385	NEU
S_protein	4	867	G	**1.311**	NEU	G	-0.543	NEU
S_protein	4	868	T	**1.311**	NEU	T	-0.165	NEU
S_protein	4	869	A	**0.949**	NEU	I	1.426	MIN
S_protein	4	870	T	**1.311**	NEU	T	-0.033	NEU
S_protein	4	871	A	**1.095**	NEU	S	-0.446	NEU

S_protein	4	872	G	**1.311**	NEU	G	-0.635	NEU
S_protein	4	873	W	0.354	MAX	W	-1.423	MAX
S_protein	4	874	T	**1.311**	NEU	T	0.152	NEU
S_protein	4	875	F	**1.311**	MIN	F	0.999	MIN
S_protein	4	876	G	**1.311**	NEU	G	-0.483	NEU
S_protein	4	877	A	**1.311**	NEU	A	0.186	NEU
S_protein	4	878	G	0.461	NEU	G	0.661	NEU
S_protein	4	879	A	0.371	NEU	A	0.558	NEU
S_protein	4	880	A	**0.732**	NEU	A	-0.604	NEU
S_protein	4	881	L	**1.311**	MIN	L	1.352	MIN
S_protein	4	882	Q	**1.311**	NEU	Q	0.321	NEU
S_protein	4	883	I	**1.311**	MIN	I	1.127	MIN
S_protein	4	884	P	**1.311**	NEU	P	-0.459	NEU
S_protein	4	885	F	**1.311**	MIN	F	1.308	MIN
S_protein	4	886	A	**1.311**	NEU	A	0.111	NEU
S_protein	4	887	M	**1.311**	NEU	M	-0.166	NEU
S_protein	4	888	Q	**0.831**	NEU	Q	-0.748	NEU
S_protein	4	889	M	**1.311**	NEU	M	0.176	NEU
S_protein	4	890	A	**1.311**	NEU	A	0.359	NEU
S_protein	4	891	Y	**1.311**	MAX	Y	-2.849	MAX
S_protein	4	892	R	**1.311**	NEU	R	-0.463	NEU
S_protein	4	893	F	**1.311**	MIN	F	0.996	MIN
S_protein	4	894	N	**1.311**	NEU	N	-0.795	NEU
S_protein	4	895	G	**1.311**	NEU	G	0.371	NEU
S_protein	4	896	I	**1.311**	MIN	I	2.014	MIN
S_protein	4	897	G	**1.095**	MIN	G	0.612	NEU
S_protein	4	898	V	**1.311**	MIN	V	1.34	MIN
S_protein	4	899	T	**1.095**	NEU	T	0.49	NEU
S_protein	4	900	Q	**1.311**	MAX	Q	-1.191	MAX
S_protein	4	901	N	**1.311**	NEU	N	-0.878	NEU
S_protein	4	902	V	**1.311**	MIN	V	1.414	MIN
S_protein	4	903	L	**1.311**	MIN	L	1.37	MIN
S_protein	4	904	Y	**1.311**	MAX	Y	-2.111	MAX
S_protein	4	905	E	**0.576**	MIN	E	-0.335	NEU
S_protein	4	906	N	**1.311**	MAX	N	-1.126	MAX
S_protein	4	907	Q	**1.311**	NEU	Q	-0.86	NEU
S_protein	4	908	K	**1.311**	NEU	K	-0.019	NEU
S_protein	4	909	Q	**0.514**	NEU	L	1.079	MIN
S_protein	4	910	I	**1.311**	MIN	I	1.601	MIN
S_protein	4	911	A	**1.311**	NEU	A	-0.144	NEU
S_protein	4	912	N	**1.095**	NEU	N	-0.531	NEU
S_protein	4	913	Q	**1.311**	NEU	Q	-0.755	NEU
S_protein	4	914	F	**1.311**	MIN	F	1.214	MIN
S_protein	4	915	N	**1.311**	NEU	N	-0.372	NEU
S_protein	4	916	K	**0.648**	MAX	S	-0.055	NEU
S_protein	4	917	A	**1.311**	MIN	A	0.602	NEU

S_protein	4	918	I	**1.311**	MIN	I	1.562	MIN
S_protein	4	919	S	**0.732**	NEU	G	-0.496	NEU
S_protein	4	920	Q	**0.732**	NEU	K	-1.131	MAX
S_protein	4	921	I	**1.311**	MIN	I	1.455	MIN
S_protein	4	922	Q	**0.648**	NEU	Q	-1.037	MAX
S_protein	4	923	E	**0.732**	NEU	D	1.023	MIN
S_protein	4	924	S	**1.311**	NEU	S	-0.582	NEU
S_protein	4	925	L	**1.311**	MIN	L	1.591	MIN
S_protein	4	926	T	**0.648**	MIN	S	0.099	NEU
S_protein	4	927	T	**1.311**	MIN	S	1.212	MIN
S_protein	4	928	T	0.333	MIN	T	0.427	NEU
S_protein	4	929	S	**1.311**	NEU	A	0.382	NEU
S_protein	4	930	T	**1.095**	MIN	S	0.949	MIN
S_protein	4	931	A	**1.311**	NEU	A	-0.058	NEU
S_protein	4	932	L	**1.311**	MIN	L	1.486	MIN
S_protein	4	933	G	**1.311**	NEU	G	-0.701	NEU
S_protein	4	934	K	**1.311**	NEU	K	-0.867	NEU
S_protein	4	935	L	**1.311**	MIN	L	1.201	MIN
S_protein	4	936	Q	**1.311**	NEU	Q	-0.559	NEU
S_protein	4	937	D	**1.311**	NEU	D	0.446	NEU
S_protein	4	938	V	**1.311**	MIN	V	1.118	MIN
S_protein	4	939	V	**1.311**	MIN	V	1.152	MIN
S_protein	4	940	N	**1.311**	NEU	N	0.265	NEU
S_protein	4	941	Q	**1.311**	MAX	Q	-1.1	MAX
S_protein	4	942	N	**0.831**	NEU	N	-0.907	NEU
S_protein	4	943	A	**1.311**	MIN	A	0.954	MIN
S_protein	4	944	Q	0.481	NEU	Q	0.501	NEU
S_protein	4	945	A	**1.311**	NEU	A	-0.719	NEU
S_protein	4	946	L	**1.311**	MIN	L	1.483	MIN
S_protein	4	947	N	**0.648**	NEU	N	-0.816	NEU
S_protein	4	948	T	**1.311**	NEU	T	0.438	NEU
S_protein	4	949	L	**1.311**	MIN	L	1.548	MIN
S_protein	4	950	V	**1.311**	MIN	V	1.09	MIN
S_protein	4	951	K	**1.311**	MAX	K	-2.084	MAX
S_protein	4	952	Q	**0.732**	NEU	Q	-0.855	NEU
S_protein	4	953	L	**1.311**	MIN	L	0.923	MIN
S_protein	4	954	S	**1.311**	NEU	S	-0.069	NEU
S_protein	4	955	S	**1.311**	NEU	S	-0.153	NEU
S_protein	4	956	N	**1.311**	NEU	N	0.166	NEU
S_protein	4	957	F	**1.311**	NEU	F	0.437	NEU
S_protein	4	958	G	**1.311**	NEU	G	-0.96	NEU
S_protein	4	959	A	**1.311**	NEU	A	0.144	NEU
S_protein	4	960	I	**1.311**	MIN	I	1.032	MIN
S_protein	4	961	S	**1.311**	NEU	S	-0.573	NEU
S_protein	4	962	S	**1.311**	NEU	S	0.281	NEU
S_protein	4	963	V	0.319	MIN	V	0.736	NEU

S_protein	4	964	L	**1.311**	MIN	L	1.395	MIN
S_protein	4	965	N	**1.095**	NEU	N	-0.925	NEU
S_protein	4	966	D	**1.311**	MIN	D	0.748	NEU
S_protein	4	967	I	**1.311**	MIN	I	1.878	MIN
S_protein	4	968	L	**1.311**	MIN	L	1.183	MIN
S_protein	4	969	S	**1.311**	MIN	S	1.028	MIN
S_protein	4	970	R	**1.311**	MIN	R	1.982	MIN
S_protein	4	971	L	**1.311**	MIN	L	1.241	MIN
S_protein	4	972	D	**1.311**	MAX	D	-1.569	MAX
S_protein	4	973	K	**1.095**	MIN	K	1.859	MIN
S_protein	4	974	V	**1.095**	MIN	V	1.172	MIN
S_protein	4	975	E	**1.095**	NEU	E	0.53	NEU
S_protein	4	976	A	**1.095**	MIN	A	0.698	NEU
S_protein	4	977	E	**0.949**	NEU	E	-0.946	NEU
S_protein	4	978	V	**1.311**	MIN	V	1.495	MIN
S_protein	4	979	Q	**1.311**	NEU	Q	-0.763	NEU
S_protein	4	980	I	**1.311**	MIN	I	1.335	MIN
S_protein	4	981	D	**1.311**	MAX	D	-1.441	MAX
S_protein	4	982	R	**1.311**	NEU	R	-0.563	NEU
S_protein	4	983	L	**1.311**	MIN	L	1.143	MIN
S_protein	4	984	I	**1.311**	MIN	I	1.147	MIN
S_protein	4	985	T	**1.311**	NEU	T	0.236	NEU
S_protein	4	986	G	0.333	NEU	G	-0.995	NEU
S_protein	4	987	R	**1.311**	NEU	R	-0.399	NEU
S_protein	4	988	L	**1.311**	MIN	L	1.208	MIN
S_protein	4	989	Q	**1.311**	NEU	Q	-0.072	NEU
S_protein	4	990	S	**1.311**	NEU	S	0.124	NEU
S_protein	4	991	L	**1.311**	MIN	L	1.082	MIN
S_protein	4	992	Q	**1.311**	NEU	Q	-0.936	NEU
S_protein	4	993	T	**1.311**	MIN	T	1.346	MIN
S_protein	4	994	Y	**1.311**	NEU	Y	-0.751	NEU
S_protein	4	995	V	**1.311**	MIN	V	1.252	MIN
S_protein	4	996	T	**1.311**	NEU	T	0.048	NEU
S_protein	4	997	Q	**1.311**	NEU	Q	-0.658	NEU
S_protein	4	998	Q	**1.311**	NEU	Q	-0.851	NEU
S_protein	4	999	L	**1.311**	MIN	L	1.507	MIN
S_protein	4	1000	I	**1.311**	MIN	I	1.164	MIN
S_protein	4	1001	R	**1.311**	NEU	R	-0.073	NEU
S_protein	4	1002	A	**1.311**	MIN	A	0.765	NEU
S_protein	4	1003	A	**1.311**	NEU	A	0.177	NEU
S_protein	4	1004	E	**1.095**	NEU	E	-0.26	NEU
S_protein	4	1005	I	**1.311**	MIN	I	1.211	MIN
S_protein	4	1006	R	0.382	MIN	R	0.659	NEU
S_protein	4	1007	A	**1.311**	NEU	A	0.196	NEU
S_protein	4	1008	S	**1.311**	NEU	S	-0.851	NEU
S_protein	4	1009	A	**1.311**	MIN	A	0.637	NEU

S_protein	4	1010	N	**1.095**	NEU	N	-0.888	NEU
S_protein	4	1011	L	**1.311**	MIN	L	1.294	MIN
S_protein	4	1012	A	**1.311**	MIN	A	1.051	MIN
S_protein	4	1013	A	**1.311**	MIN	A	0.947	MIN
S_protein	4	1014	T	**1.311**	NEU	T	-0.021	NEU
S_protein	4	1015	K	**1.095**	NEU	K	-0.831	NEU
S_protein	4	1016	M	**0.648**	MIN	M	0.777	NEU
S_protein	4	1017	S	**1.311**	NEU	S	-0.717	NEU
S_protein	4	1018	E	**0.648**	NEU	E	0.242	NEU
S_protein	4	1019	C	**1.311**	MIN	C	1.857	MIN
S_protein	4	1020	V	**1.311**	MIN	V	1.232	MIN
S_protein	4	1021	L	**1.311**	MIN	L	1.35	MIN
S_protein	4	1022	G	**1.311**	NEU	G	-0.883	NEU
S_protein	4	1023	Q	**1.311**	NEU	Q	-0.476	NEU
S_protein	4	1024	S	**1.311**	NEU	S	0.037	NEU
S_protein	4	1025	K	**1.311**	NEU	K	-0.443	NEU
S_protein	4	1026	R	**1.311**	MIN	R	2.032	MIN
S_protein	4	1027	V	**1.311**	NEU	V	-0.505	NEU
S_protein	4	1028	D	**1.311**	MIN	D	2.962	MIN
S_protein	4	1029	F	**1.311**	NEU	F	-0.816	NEU
S_protein	4	1030	C	**1.311**	MIN	C	1.539	MIN
S_protein	4	1031	G	**1.311**	NEU	G	-0.379	NEU
S_protein	4	1032	K	**0.732**	MAX	K	-1.45	MAX
S_protein	4	1033	G	**1.311**	NEU	G	-0.696	NEU
S_protein	4	1034	Y	**0.648**	MAX	Y	-1.149	MAX
S_protein	4	1035	H	**0.831**	NEU	H	0.253	NEU
S_protein	4	1036	L	**1.311**	MIN	L	1.203	MIN
S_protein	4	1037	M	**1.311**	MAX	M	-1.511	MAX
S_protein	4	1038	S	**1.311**	NEU	S	-0.571	NEU
S_protein	4	1039	F	**1.311**	MIN	F	1.573	MIN
S_protein	4	1040	P	0.354	MAX	P	-0.99	NEU
S_protein	4	1041	Q	**1.311**	NEU	Q	-0.838	NEU
S_protein	4	1042	A	**0.831**	NEU	S	-0.601	NEU
S_protein	4	1043	A	0.319	MIN	A	0.595	NEU
S_protein	4	1044	P	**1.095**	NEU	P	-0.919	NEU
S_protein	4	1045	H	**1.311**	NEU	H	-0.264	NEU
S_protein	4	1046	G	**1.311**	NEU	G	-0.467	NEU
S_protein	4	1047	V	**1.311**	MIN	V	1.167	MIN
S_protein	4	1048	V	**1.311**	MIN	V	1.213	MIN
S_protein	4	1049	F	**1.311**	MIN	F	1.865	MIN
S_protein	4	1050	L	**1.311**	MIN	L	1.304	MIN
S_protein	4	1051	H	**1.311**	NEU	H	0.044	NEU
S_protein	4	1052	V	**1.311**	MIN	V	1.192	MIN
S_protein	4	1053	T	**1.311**	NEU	T	-0.259	NEU
S_protein	4	1054	Y	**1.311**	MIN	Y	0.818	MIN
S_protein	4	1055	V	**1.311**	MIN	V	1.385	MIN

S_protein	4	1056	P	**1.311**	NEU	P	-0.555	NEU
S_protein	4	1057	S	**0.831**	NEU	A	0.895	MIN
S_protein	4	1058	Q	**1.311**	NEU	Q	-0.366	NEU
S_protein	4	1059	E	**0.576**	NEU	E	-1.022	MAX
S_protein	4	1060	R	0.382	NEU	K	-1.358	MAX
S_protein	4	1061	N	**1.311**	NEU	N	-0.47	NEU
S_protein	4	1062	F	**1.311**	MIN	F	1.269	MIN
S_protein	4	1063	T	**1.311**	NEU	T	-0.255	NEU
S_protein	4	1064	T	**1.311**	NEU	T	-0.287	NEU
S_protein	4	1065	A	**1.311**	NEU	A	-0.101	NEU
S_protein	4	1066	P	**0.949**	NEU	P	-0.863	NEU
S_protein	4	1067	A	**1.095**	NEU	A	0.544	NEU
S_protein	4	1068	I	**1.311**	MIN	I	1.048	MIN
S_protein	4	1069	C	**1.311**	MIN	C	1.613	MIN
S_protein	4	1070	H	**1.311**	NEU	H	-0.771	NEU
S_protein	4	1071	E	**0.621**	NEU	D	1.014	MIN
S_protein	4	1072	G	**1.311**	NEU	G	0.221	NEU
S_protein	4	1073	K	**0.732**	NEU	K	-0.692	NEU
S_protein	4	1074	A	**1.311**	MIN	A	0.719	NEU
S_protein	4	1075	Y	**1.311**	NEU	H	-0.477	NEU
S_protein	4	1076	F	**1.311**	MIN	F	1.298	MIN
S_protein	4	1077	P	**0.949**	MAX	P	-1	MAX
S_protein	4	1078	R	**1.311**	NEU	R	-0.761	NEU
S_protein	4	1079	E	0.418	NEU	E	0.784	MIN
S_protein	4	1080	G	**1.311**	NEU	G	-0.521	NEU
S_protein	4	1081	V	**1.311**	MIN	V	1.382	MIN
S_protein	4	1082	F	**1.311**	MIN	F	1.36	MIN
S_protein	4	1083	V	**1.311**	MIN	V	1.735	MIN
S_protein	4	1084	S	0.418	NEU	S	-0.578	NEU
S_protein	4	1085	N	**1.095**	NEU	N	-0.815	NEU
S_protein	4	1086	G	**1.311**	NEU	G	-0.359	NEU
S_protein	4	1087	T	**0.949**	NEU	T	-0.238	NEU
S_protein	4	1088	S	**0.949**	NEU	H	0.29	NEU
S_protein	4	1089	W	0.319	MIN	W	0.966	MIN
S_protein	4	1090	F	**1.311**	MIN	F	1.05	MIN
S_protein	4	1091	I	**1.311**	MIN	V	1.431	MIN
S_protein	4	1092	T	**1.311**	NEU	T	-0.17	NEU
S_protein	4	1093	Q	**0.949**	NEU	Q	-1.055	MAX
S_protein	4	1094	R	**1.311**	NEU	R	-0.293	NEU
S_protein	4	1095	N	**1.095**	NEU	N	-0.929	NEU
S_protein	4	1096	F	**1.311**	MIN	F	1.18	MIN
S_protein	4	1097	Y	0.418	MAX	Y	-3.363	MAX
S_protein	4	1098	S	**1.311**	NEU	E	-0.825	NEU
S_protein	4	1099	P	**0.949**	NEU	P	-0.473	NEU
S_protein	4	1100	Q	**1.311**	NEU	Q	-0.126	NEU
S_protein	4	1101	I	**0.732**	MIN	I	2.249	MIN

S_protein	4	1102	I	**1.311**	MIN	I	1.24	MIN
S_protein	4	1103	T	**1.311**	NEU	T	-0.735	NEU
S_protein	4	1104	T	**1.311**	NEU	T	0.064	NEU
S_protein	4	1105	D	**1.095**	NEU	D	0.411	NEU
S_protein	4	1106	N	**0.831**	NEU	N	-0.928	NEU
S_protein	4	1107	T	**1.311**	NEU	T	-0.38	NEU
S_protein	4	1108	F	**0.949**	MIN	F	0.607	NEU
S_protein	4	1109	V	**1.095**	MIN	V	1.329	MIN
S_protein	4	1110	S	0.382	NEU	S	-0.853	NEU
S_protein	4	1111	G	**0.831**	NEU	G	-0.882	NEU
S_protein	4	1112	N	**1.311**	NEU	N	-0.593	NEU
S_protein	4	1113	C	0.461	MIN	C	0.694	NEU
S_protein	4	1114	D	**1.095**	NEU	D	0.223	NEU
S_protein	4	1115	V	**1.311**	MIN	V	0.772	NEU
S_protein	4	1116	V	**1.311**	MIN	V	1.327	MIN
S_protein	4	1117	I	**1.311**	MIN	I	2.454	MIN
S_protein	4	1118	G	**1.311**	NEU	G	-0.815	NEU
S_protein	4	1119	I	**1.311**	MIN	I	1.64	MIN
S_protein	4	1120	I	**1.311**	MIN	V	0.726	NEU
S_protein	4	1121	N	**1.311**	MAX	N	-1.039	MAX
S_protein	4	1122	N	**1.311**	MAX	N	-1.357	MAX
S_protein	4	1123	T	**1.311**	NEU	T	-0.432	NEU
S_protein	4	1124	V	**1.311**	MIN	V	1.222	MIN
S_protein	4	1125	Y	**0.831**	NEU	Y	0.635	NEU
S_protein	4	1126	D	**1.311**	MAX	D	-1.457	MAX
S_protein	4	1127	P	**1.311**	NEU	P	0.338	NEU
S_protein	4	1128	L	**1.311**	NEU	L	-0.671	NEU
S_protein	4	1129	Q	**0.949**	NEU	Q	0.274	NEU
S_protein	4	1130	P	-0.142	NEU	P	-0.06	NEU
S_protein	4	1131	E	0.319	NEU	E	-1.006	MAX
S_protein	4	1132	L	0.461	NEU	L	-0.305	NEU
S_protein	4	1133	D	0.319	NEU	D	0.575	NEU
S_protein	4	1134	S	0.461	MIN	S	0.695	NEU
S_protein	4	1135	F	**1.311**	NEU	F	-0.74	NEU
S_protein	4	1136	K	**0.514**	MIN	K	1.622	MIN
S_protein	4	1137	E	**1.095**	NEU	E	-0.272	NEU
S_protein	4	1138	E	**1.311**	NEU	E	0.247	NEU
S_protein	4	1139	L	**1.311**	MIN	L	1.32	MIN
S_protein	4	1140	D	**1.311**	NEU	D	-0.531	NEU
S_protein	4	1141	K	**0.949**	NEU	K	0.162	NEU
S_protein	4	1142	Y	**0.576**	MAX	Y	-1.438	MAX
S_protein	4	1143	F	**1.311**	NEU	F	-0.118	NEU
S_protein	4	1144	K	**1.311**	MIN	K	0.93	MIN
S_protein	4	1145	N	**1.311**	NEU	N	-0.377	NEU
S_protein	4	1146	H	**1.311**	MIN	H	0.741	NEU
S_protein	4	1147	T	**1.311**	NEU	T	-0.206	NEU

S_protein	4	1148	S	**1.311**	NEU	S	0.099	NEU
S_protein	4	1149	P	**0.949**	NEU	P	-0.43	NEU
E	1	1	E	**1.079**	MAX	E	-1.954	MAX
E	1	2	T	**0.582**	MAX	T	-1.045	MAX
E	1	3	G	**1.308**	MAX	G	-1.753	MAX
E	1	4	T	**1.308**	NEU	T	0.367	NEU
E	1	5	L	**1.079**	NEU	L	-0.785	NEU
E	1	6	I	**1.308**	MIN	I	0.773	NEU
E	1	7	V	**1.308**	MIN	V	0.758	NEU
E	1	8	N	**1.308**	MAX	N	-2.152	MAX
E	1	9	S	**0.853**	MAX	S	-1.71	MAX
E	1	10	V	**1.308**	MIN	V	0.652	NEU
E	1	11	L	**0.927**	MIN	L	0.728	NEU
E	1	12	L	**0.582**	NEU	L	0.511	NEU
E	1	13	F	**0.804**	NEU	F	0.463	NEU
E	1	14	L	**0.927**	MIN	L	0.616	NEU
E	1	15	A	**0.927**	NEU	A	0.425	NEU
E	1	16	F	**0.927**	NEU	F	0.385	NEU
E	1	17	V	**0.702**	MIN	V	0.584	NEU
E	1	18	V	**0.927**	MIN	V	0.781	MIN
E	1	19	F	**1.308**	NEU	F	0.134	NEU
E	1	20	L	0.431	NEU	L	0.528	NEU
E	1	21	L	**1.079**	MIN	L	0.717	NEU
E	1	22	V	**1.079**	MIN	V	0.804	MIN
E	1	23	T	0.436	MAX	T	-1.193	MAX
E	1	24	L	**0.927**	NEU	L	0.507	NEU
E	1	25	A	**1.308**	NEU	A	0.374	NEU
E	1	26	I	**0.853**	MIN	I	0.743	NEU
E	1	27	L	**1.308**	MIN	L	0.673	NEU
E	1	28	T	**0.927**	NEU	T	-0.994	NEU
E	1	29	A	**1.308**	NEU	A	0.307	NEU
E	1	30	L	**1.079**	MIN	L	0.684	NEU
E	1	31	R	**1.308**	MAX	R	-2.162	MAX
N_Nterm	1	1	N	-0.050	NEU	N	1.141	MIN
N_Nterm	1	2	N	**0.713**	NEU	T	-0.6	NEU
N_Nterm	1	3	V	**0.553**	MIN	A	-0.641	NEU
N_Nterm	1	4	S	**1.338**	NEU	S	-0.722	NEU
N_Nterm	1	5	W	**0.565**	NEU	W	-0.014	NEU
N_Nterm	1	6	F	**1.222**	MIN	F	1.447	MIN
N_Nterm	1	7	T	**0.795**	NEU	T	-0.086	NEU
N_Nterm	1	8	P	**0.840**	NEU	A	0.331	NEU
N_Nterm	1	9	L	**1.338**	MIN	L	1.654	MIN
N_Nterm	1	10	T	0.216	NEU	T	-0.008	NEU
N_Nterm	1	11	Q	0.442	NEU	Q	-1.225	MAX
N_Nterm	1	12	H	0.396	MIN	H	0.959	MIN
N_Nterm	1	13	G	0.345	MIN	G	1.098	MIN

N_Nterm	1	14	K	**1.138**	MIN	K	1.569	MIN
N_Nterm	1	15	Q	0.195	NEU	E	0.313	NEU
N_Nterm	1	16	P	0.345	MIN	D	2.43	MIN
N_Nterm	1	17	L	**1.138**	MIN	L	1.59	MIN
N_Nterm	1	18	R	-0.026	MIN	K	1.694	MIN
N_Nterm	1	19	F	**1.338**	MIN	F	0.93	MIN
N_Nterm	1	20	P	0.125	NEU	P	-0.251	NEU
N_Nterm	1	21	R	0.394	MIN	R	1.116	MIN
N_Nterm	1	22	G	**1.338**	NEU	G	-0.258	NEU
N_Nterm	1	23	Q	**0.879**	NEU	Q	-0.416	NEU
N_Nterm	1	24	G	**0.795**	NEU	G	-0.354	NEU
N_Nterm	1	25	V	**1.338**	MIN	V	2.03	MIN
N_Nterm	1	26	P	**1.338**	NEU	P	-0.857	NEU
N_Nterm	1	27	I	**1.222**	MIN	I	2.63	MIN
N_Nterm	1	28	N	**1.138**	NEU	N	-0.592	NEU
N_Nterm	1	29	A	0.238	MAX	T	-0.302	NEU
N_Nterm	1	30	N	0.339	MIN	N	-0.024	NEU
N_Nterm	1	31	S	0.461	MIN	S	-0.101	NEU
N_Nterm	1	32	T	0.170	NEU	S	1.064	MIN
N_Nterm	1	33	P	0.101	NEU	P	0.448	NEU
N_Nterm	1	34	D	0.290	NEU	D	-0.455	NEU
N_Nterm	1	35	Q	0.394	NEU	D	-0.455	NEU
N_Nterm	1	36	N	**0.623**	NEU	Q	-0.474	NEU
N_Nterm	1	37	H	**0.527**	NEU	I	1.895	MIN
N_Nterm	1	38	G	**1.338**	NEU	G	-0.257	NEU
N_Nterm	1	39	Y	**1.338**	MIN	Y	0.892	MIN
N_Nterm	1	40	W	**1.338**	MIN	Y	0.884	MIN
N_Nterm	1	41	R	0.396	NEU	R	-0.428	NEU
N_Nterm	1	42	R	**1.107**	NEU	R	-0.014	NEU
N_Nterm	1	43	Q	**1.138**	NEU	A	-1.489	MAX
N_Nterm	1	44	D	0.279	NEU	T	0.244	NEU
N_Nterm	1	45	R	0.091	NEU	R	1.005	MIN
N_Nterm	1	46	K	-0.182	NEU	R	1.717	MIN
N_Nterm	1	47	F	0.435	MIN	I	2.296	MIN
N_Nterm	1	48	N	0.020	NEU	R	1.512	MIN
N_Nterm	1	49	K	-0.201	NEU	G	0.746	NEU
N_Nterm	1	50	G	0.364	NEU	G	0.416	NEU
N_Nterm	1	51	-	0.336	NEU	D	2.387	MIN
N_Nterm	1	52	G	0.356	MIN	G	1.361	MIN
N_Nterm	1	53	K	0.347	MAX	K	0.378	NEU
N_Nterm	1	54	M	0.205	NEU	M	0.843	MIN
N_Nterm	1	55	K	-0.186	MIN	K	-0.585	NEU
N_Nterm	1	56	Q	0.234	NEU	D	1.865	MIN
N_Nterm	1	57	L	0.345	NEU	L	0.149	NEU
N_Nterm	1	58	A	0.240	NEU	S	0.597	NEU
N_Nterm	1	59	P	0.461	MAX	P	-0.127	NEU

N_Nterm	1	60	R	**1.138**	NEU	R	-0.078	NEU
N_Nterm	1	61	W	**1.338**	MIN	W	1.015	MIN
N_Nterm	1	62	Y	**0.553**	MIN	Y	0.614	NEU
N_Nterm	1	63	F	**1.338**	MIN	F	1.844	MIN
N_Nterm	1	64	Y	**1.222**	MIN	Y	0.778	NEU
N_Nterm	1	65	Y	-0.050	MAX	Y	-1.303	MAX
N_Nterm	1	66	T	**0.527**	NEU	L	1.539	MIN
N_Nterm	1	67	G	**1.338**	NEU	G	0.071	NEU
N_Nterm	1	68	T	**1.338**	NEU	T	-0.176	NEU
N_Nterm	1	69	G	**1.338**	NEU	G	-0.673	NEU
N_Nterm	1	70	P	0.085	MAX	P	-1.325	MAX
N_Nterm	1	71	E	0.061	NEU	E	-1.022	MAX
N_Nterm	1	72	A	**1.138**	NEU	A	-0.63	NEU
N_Nterm	1	73	D	0.023	NEU	G	0.161	NEU
N_Nterm	1	74	L	**0.840**	MIN	L	1.781	MIN
N_Nterm	1	75	P	0.425	MIN	P	-0.079	NEU
N_Nterm	1	76	F	0.353	MIN	Y	0.735	NEU
N_Nterm	1	77	G	**0.503**	NEU	G	-0.516	NEU
N_Nterm	1	78	T	**0.753**	NEU	A	-0.013	NEU
N_Nterm	1	79	V	0.238	NEU	N	-0.343	NEU
N_Nterm	1	80	K	0.216	NEU	K	-0.875	NEU
N_Nterm	1	81	D	**0.943**	MIN	D	1.299	MIN
N_Nterm	1	82	G	0.324	NEU	G	-0.536	NEU
N_Nterm	1	83	I	**1.338**	MIN	I	1.582	MIN
N_Nterm	1	84	V	**0.733**	MIN	I	2.112	MIN
N_Nterm	1	85	W	0.339	NEU	W	0.064	NEU
N_Nterm	1	86	V	**1.338**	MIN	V	1.676	MIN
N_Nterm	1	87	G	0.130	NEU	A	-0.544	NEU
N_Nterm	1	88	E	0.181	MAX	T	-0.325	NEU
N_Nterm	1	89	E	0.425	MIN	E	0.976	MIN
N_Nterm	1	90	G	**1.001**	NEU	G	-0.315	NEU
N_Nterm	1	91	A	-0.060	NEU	A	-0.103	NEU
N_Nterm	1	92	N	0.338	MIN	L	0.803	MIN
N_Nterm	1	93	N	0.425	MIN	N	-0.532	NEU
N_Nterm	1	94	A	-0.197	NEU	T	-0.331	NEU
N_Nterm	1	95	P	**0.503**	NEU	P	-0.385	NEU
N_Nterm	1	96	-	0.249	MIN	K	-1.358	MAX
N_Nterm	1	97	S	0.451	NEU	D	-1.114	MAX
N_Nterm	1	98	D	0.410	NEU	H	1.446	MIN
N_Nterm	1	99	F	0.364	MIN	I	1.12	MIN
N_Nterm	1	100	G	**1.338**	NEU	G	-0.052	NEU
N_Nterm	1	101	T	**1.338**	NEU	T	-0.069	NEU
N_Nterm	1	102	R	**1.138**	NEU	R	-0.454	NEU
N_Nterm	1	103	N	**0.890**	MIN	N	0.896	MIN
N_Nterm	1	104	P	**0.875**	NEU	P	-0.231	NEU
N_Nterm	1	105	N	0.341	MIN	A	-0.547	NEU

N_Nterm	1	106	N	**1.338**	MIN	N	1.635	MIN
N_Nterm	1	107	D	**0.567**	MIN	N	0.47	NEU
N_Nterm	1	108	A	-0.105	MAX	A	-1.204	MAX
N_Nterm	1	109	A	**1.338**	NEU	A	-0.264	NEU
N_Nterm	1	110	I	**1.219**	MIN	I	2.2	MIN
N_Nterm	1	111	V	0.108	MIN	V	2.256	MIN
N_Nterm	1	112	T	0.375	MIN	L	1.615	MIN
N_Nterm	1	113	Q	**1.132**	NEU	Q	-0.523	NEU
N_Nterm	1	114	L	**0.783**	MIN	L	1.109	MIN
N_Nterm	1	115	A	**0.629**	NEU	P	0.113	NEU
N_Nterm	1	116	P	**0.829**	NEU	Q	-0.459	NEU
N_Nterm	1	117	G	**1.100**	NEU	G	0.102	NEU
N_Nterm	1	118	T	**0.614**	NEU	T	0.524	NEU
N_Nterm	1	119	-	-0.021	NEU	T	0.761	NEU
N_Nterm	1	120	L	**1.222**	MIN	L	1.728	MIN
N_Nterm	1	121	P	-0.176	NEU	P	-0.926	NEU
N_Nterm	1	122	K	-0.213	MIN	K	1.477	MIN
N_Nterm	1	123	G	-0.102	NEU	G	1.188	MIN
N_Nterm	1	124	F	**1.222**	MIN	F	1.316	MIN
N_Nterm	1	125	Y	0.037	NEU	Y	-1.231	MAX
N_Cterm	1	1	T	0.350	NEU	T	0.42	NEU
N_Cterm	1	2	K	**0.803**	NEU	K	-0.908	NEU
N_Cterm	1	3	K	0.111	NEU	K	-0.425	NEU
N_Cterm	1	4	D	**0.829**	NEU	S	-0.116	NEU
N_Cterm	1	5	A	0.408	NEU	A	0.422	NEU
N_Cterm	1	6	A	**1.065**	NEU	A	0.232	NEU
N_Cterm	1	7	A	0.205	MIN	E	2.542	MIN
N_Cterm	1	8	A	**0.642**	NEU	A	0.064	NEU
N_Cterm	1	9	A	**0.796**	NEU	S	-0.218	NEU
N_Cterm	1	10	K	0.356	MIN	K	2.012	MIN
N_Cterm	1	11	K	0.452	NEU	K	0.338	NEU
N_Cterm	1	12	L	**0.890**	NEU	P	-0.311	NEU
N_Cterm	1	13	R	**0.803**	NEU	R	0.005	NEU
N_Cterm	1	14	H	**1.138**	NEU	Q	-0.305	NEU
N_Cterm	1	15	K	0.442	NEU	K	-0.045	NEU
N_Cterm	1	16	R	**0.676**	NEU	R	-0.698	NEU
N_Cterm	1	17	T	**0.943**	MIN	T	0.877	MIN
N_Cterm	1	18	A	**1.001**	NEU	A	-0.683	NEU
N_Cterm	1	19	T	**1.065**	NEU	T	0.112	NEU
N_Cterm	1	20	K	-0.115	NEU	K	-1.116	MAX
N_Cterm	1	21	G	**0.867**	NEU	A	-0.36	NEU
N_Cterm	1	22	Y	0.026	NEU	Y	-2.427	MAX
N_Cterm	1	23	N	0.499	NEU	N	-0.675	NEU
N_Cterm	1	24	V	0.443	MIN	V	1.632	MIN
N_Cterm	1	25	T	**0.890**	NEU	T	0.141	NEU
N_Cterm	1	26	Q	-0.026	MAX	Q	-1.397	MAX

N_Cterm	1	27	A	0.102	NEU	A	-0.457	NEU
N_Cterm	1	28	F	0.125	NEU	F	-0.691	NEU
N_Cterm	1	29	G	**0.943**	NEU	G	-0.612	NEU
N_Cterm	1	30	R	0.481	NEU	R	-0.063	NEU
N_Cterm	1	31	R	0.481	MIN	R	0.701	NEU
N_Cterm	1	32	G	**0.753**	MIN	G	0.621	NEU
N_Cterm	1	33	P	**0.581**	MIN	P	0.946	MIN
N_Cterm	1	34	G	0.119	NEU	E	1.044	MIN
N_Cterm	1	35	D	0.176	NEU	Q	0.514	NEU
N_Cterm	1	36	L	0.139	MIN	T	0.838	MIN
N_Cterm	1	37	Q	**1.058**	NEU	Q	-0.065	NEU
N_Cterm	1	38	G	**1.338**	NEU	G	-0.056	NEU
N_Cterm	1	39	N	**0.601**	NEU	N	0.641	NEU
N_Cterm	1	40	F	**0.527**	MIN	F	0.698	NEU
N_Cterm	1	41	G	**0.713**	NEU	G	-0.5	NEU
N_Cterm	1	42	D	0.373	NEU	D	-0.07	NEU
N_Cterm	1	43	L	0.172	NEU	Q	-0.861	NEU
N_Cterm	1	44	E	0.394	NEU	E	0.084	NEU
N_Cterm	1	45	L	0.111	MIN	L	0.689	NEU
N_Cterm	1	46	L	**0.503**	MIN	I	1.115	MIN
N_Cterm	1	47	K	**1.001**	MIN	R	1.757	MIN
N_Cterm	1	48	L	0.339	NEU	Q	-0.977	NEU
N_Cterm	1	49	G	**1.338**	NEU	G	-0.102	NEU
N_Cterm	1	50	T	0.443	NEU	T	-0.031	NEU
N_Cterm	1	51	D	0.335	MIN	D	1.112	MIN
N_Cterm	1	52	D	-0.214	MIN	Y	-2	MAX
N_Cterm	1	53	P	0.338	MIN	K	1.1	MIN
N_Cterm	1	54	R	**0.527**	MIN	H	-0.116	NEU
N_Cterm	1	55	W	-0.236	MIN	W	-1.111	MAX
N_Cterm	1	56	P	**1.338**	NEU	P	0.008	NEU
N_Cterm	1	57	Q	**0.829**	NEU	Q	-0.848	NEU
N_Cterm	1	58	I	0.345	MIN	I	1.693	MIN
N_Cterm	1	59	A	**0.943**	MIN	A	0.766	NEU
N_Cterm	1	60	Q	**1.065**	NEU	Q	0.031	NEU
N_Cterm	1	61	L	**1.065**	MIN	F	1.281	MIN
N_Cterm	1	62	A	**1.222**	NEU	A	0.102	NEU
N_Cterm	1	63	P	0.425	NEU	P	1.013	MIN
N_Cterm	1	64	S	**0.713**	NEU	S	0.005	NEU
N_Cterm	1	65	A	0.318	MIN	A	0.647	NEU
N_Cterm	1	66	S	**0.581**	NEU	S	0.3	NEU
N_Cterm	1	67	A	**1.138**	NEU	A	0.365	NEU
N_Cterm	1	68	F	**1.065**	MIN	F	1.307	MIN
N_Cterm	1	69	L	0.325	MIN	F	0.926	MIN
N_Cterm	1	70	G	0.384	NEU	G	0.108	NEU
N_Cterm	1	71	M	0.279	MIN	M	1.095	MIN
N_Cterm	1	72	S	**1.338**	NEU	S	-0.301	NEU

N_Cterm	1	73	H	**0.693**	NEU	R	-0.039	NEU
N_Cterm	1	74	F	**0.753**	MIN	I	2.276	MIN
N_Cterm	1	75	K	0.061	NEU	G	0.161	NEU
N_Cterm	1	76	L	0.461	MIN	M	0.695	NEU
N_Cterm	1	77	R	0.338	MIN	E	0.688	NEU
N_Cterm	1	78	H	-0.065	NEU	V	2.228	MIN
N_Cterm	1	79	E	0.040	NEU	T	1.012	MIN
N_Cterm	1	80	S	0.481	NEU	P	0.87	MIN
N_Cterm	1	81	D	0.198	NEU	S	0.248	NEU
N_Cterm	1	82	A	0.365	NEU	G	0.14	NEU
N_Cterm	1	83	T	**0.585**	NEU	T	0.345	NEU
N_Cterm	1	84	W	-0.188	MAX	W	-2.128	MAX
N_Cterm	1	85	L	**1.338**	MIN	L	1.503	MIN
N_Cterm	1	86	R	0.364	NEU	T	0.628	NEU
N_Cterm	1	87	Y	0.461	NEU	Y	-0.547	NEU
N_Cterm	1	88	S	**0.713**	NEU	T	0.781	MIN
N_Cterm	1	89	G	**0.713**	NEU	G	0.495	NEU
N_Cterm	1	90	A	0.470	NEU	A	-0.936	NEU
N_Cterm	1	91	I	**1.138**	MIN	I	2.163	MIN
N_Cterm	1	92	K	-0.045	MIN	K	1.776	MIN
N_Cterm	1	93	L	**1.065**	MIN	L	0.881	MIN
N_Cterm	1	94	D	0.020	MIN	D	1.571	MIN
N_Cterm	1	95	K	0.187	MIN	D	-0.546	NEU
N_Cterm	1	96	K	0.396	MIN	K	0.867	MIN
N_Cterm	1	97	D	0.350	NEU	D	0.748	NEU
N_Cterm	1	98	P	**0.890**	MIN	P	1.034	MIN
N_Cterm	1	99	N	0.339	NEU	N	0.948	MIN
N_Cterm	1	100	Y	0.112	MAX	F	-0.891	NEU
N_Cterm	1	101	K	**0.527**	NEU	K	0.351	NEU
N_Cterm	1	102	K	-0.006	NEU	D	-1.142	MAX
N_Cterm	1	103	W	**0.713**	NEU	Q	-0.867	NEU
N_Cterm	1	104	L	**0.503**	MIN	V	1.585	MIN
N_Cterm	1	105	E	-0.040	NEU	I	-1.229	MAX
N_Cterm	1	106	L	0.481	MIN	L	-0.143	NEU
N_Cterm	1	107	L	**1.107**	MIN	L	1.278	MIN
N_Cterm	1	108	E	0.180	MAX	N	-1.232	MAX
N_Cterm	1	109	E	0.124	NEU	K	-0.58	NEU
N_Cterm	1	110	N	**1.065**	NEU	H	0.099	NEU
N_Cterm	1	111	I	**1.338**	MIN	I	1.599	MIN
N_Cterm	1	112	D	**1.065**	MIN	D	1.895	MIN
N_Cterm	1	113	A	**0.950**	NEU	A	-0.708	NEU
N_Cterm	1	114	Y	**0.803**	MAX	Y	-3.488	MAX
N_Cterm	1	115	K	0.461	MIN	K	0.974	MIN
N_Cterm	1	116	T	**1.001**	NEU	T	-0.514	NEU
N_Cterm	1	117	F	**0.542**	NEU	F	-0.771	NEU
N_Cterm	1	118	P	0.127	MIN	P	0.309	NEU

Supplementary Table 5A: Amino acid positions that are frustrationally conserved in the SARS-CoV-2 containing subfamilies (FrustIC>0.5 and their most informative frustration state is the highly frustrated one). For full table, check Supplementary Table 4.

Protein	SARS2 containing cluster	Protein position	MSA consensus AA	FrustIC	Frustraevo state	SARS2 AA	SRFI	Frustratometer state
N_Nterm	1	43	Q	1.138	NEU	A	-1.489	MAX
S_protein	4	15	Q	0.524	NEU	Y	-1.029	MAX
S_protein	4	189	Y	0.514	NEU	K	-1.119	MAX
S_protein	4	204	P	1.095	NEU	P	-1.002	MAX
S_protein	4	265	K	0.949	NEU	K	-1.083	MAX
S_protein	4	480	S	0.949	NEU	Q	-1.128	MAX
S_protein	4	482	Y	0.648	NEU	Y	-1.035	MAX
S_protein	4	519	D	0.514	NEU	N	-1.344	MAX
S_protein	4	524	Q	0.648	NEU	K	-1.262	MAX
S_protein	4	576	P	0.949	NEU	P	-1.052	MAX
S_protein	4	593	S	0.621	NEU	N	-1.049	MAX
S_protein	4	594	E	0.949	NEU	Q	-1.005	MAX
S_protein	4	603	N	1.095	NEU	N	-1.012	MAX
S_protein	4	776	Y	0.732	NEU	Y	-1.14	MAX
S_protein	4	832	N	0.524	MIN	A	-1.027	MAX
S_protein	4	920	Q	0.732	NEU	K	-1.131	MAX
S_protein	4	922	Q	0.648	NEU	Q	-1.037	MAX
S_protein	4	1059	E	0.576	NEU	E	-1.022	MAX
S_protein	4	1093	Q	0.949	NEU	Q	-1.055	MAX
nsp10	1	72	V	0.556	NEU	F	-1.308	MAX
nsp12	1	88	K	0.552	NEU	K	-1.192	MAX
nsp12	1	185	N	0.577	NEU	K	-1.317	MAX
nsp12	1	366	K	0.577	NEU	K	-1.319	MAX
nsp12	1	506	F	0.677	MIN	W	-2.508	MAX
nsp13	1	123	N	0.701	NEU	N	-1.055	MAX
nsp13	1	280	Q	0.500	NEU	Q	-1.001	MAX
nsp13	1	449	D	0.839	NEU	D	-1.026	MAX
nsp13	1	488	N	0.500	NEU	N	-1.013	MAX
nsp14	1	27	S	0.636	NEU	S	-1.124	MAX
nsp14	1	351	D	0.754	NEU	D	-1.269	MAX
nsp14	1	394	N	0.604	NEU	N	-1.002	MAX
nsp14	1	470	N	0.953	NEU	A	-1.108	MAX
nsp14	1	479	G	0.953	NEU	G	-1.204	MAX
nsp15	1	17	G	0.651	NEU	G	-1.142	MAX
nsp15	1	20	G	0.651	NEU	G	-1.157	MAX
nsp15	1	93	P	0.651	NEU	P	-1.051	MAX
nsp15	1	187	Q	0.651	NEU	Q	-1.055	MAX
nsp15	1	264	K	0.757	NEU	E	-1.196	MAX
nsp2	1	35	Q	0.663	NEU	Q	-1.009	MAX
nsp2	1	71	P	0.529	NEU	P	-1.029	MAX
nsp2	1	162	G	0.960	NEU	G	-1.13	MAX
nsp2	1	186	P	0.591	NEU	Y	-3.026	MAX
nsp2	1	203	D	0.529	NEU	E	-1.559	MAX
nsp2	1	324	G	0.960	NEU	G	-1.004	MAX
nsp2	1	341	G	0.591	NEU	G	-1.163	MAX
nsp2	1	374	H	0.960	NEU	N	-1.199	MAX
nsp2	1	411	S	0.529	NEU	N	-1.104	MAX
nsp3_MacroX	1	19	C	0.544	MIN	N	-1.012	MAX
nsp3_NAB	1	75	K	0.632	NEU	K	-1.03	MAX
nsp3_PLPro	1	95	G	0.544	NEU	N	-1.074	MAX
nsp3_PLPro	1	228	Q	0.905	NEU	K	-1.325	MAX
nsp3_PLPro	1	253	T	0.606	NEU	T	-1.152	MAX
nsp3_SUD_Nt	1	72	P	0.677	NEU	P	-1.067	MAX
nsp3_SUD_Nt	1	102	Q	1.109	NEU	Q	-1.126	MAX
nsp3_Ubl1	1	47	G	0.760	NEU	G	-1.128	MAX
nsp3_Y3	1	90	Q	0.544	NEU	Q	-1.122	MAX
nsp5	1	7	A	0.781	NEU	A	-1.104	MAX
nsp5	1	102	T	0.558	NEU	K	-1.425	MAX
nsp5	1	154	G	0.558	NEU	Y	-1.047	MAX
nsp5	1	236	K	0.505	NEU	K	-1.19	MAX
nsp5	1	240	T	0.620	NEU	E	-1.061	MAX
nsp5	1	244	G	0.558	NEU	Q	-1.059	MAX

Supplementary Table 5B: Frequency of frustrationally conserved positions per protein.

Protein	Count	Protein length	Norm (Count/Protein length)
nsp5	6	306	0.02
nsp15	5	346	0.014
nsp2	9	638	0.014
S_protein	18	1273	0.014
nsp3_PLPro	3	308	0.01
nsp3_Y3	1	101	0.01
nsp14	5	527	0.009
nsp3_NAB	1	112	0.009
nsp3_Ubl1	1	110	0.009
N_Nterm	1	124	0.008
nsp3_SUD_Nterr	2	263	0.008
nsp10	1	139	0.007
nsp13	4	601	0.007
nsp3_MacroX	1	171	0.006
nsp12	4	932	0.004

Supplementary Table 6. Non-redundant dataset of the 21 mammalian hemoglobins used in section 3.

PDB ID	Organism	Cell Type	Bound/Unbound state
1fsx	Bos taurus (Bovine)	Mammalia	Deoxy
3d4x	Felis catus (Cat) (Felis silvestris catus)	Mammalia	Deoxy
2dn1	Homo sapiens (Human)	Mammalia	Deoxy
3vre	Mammuthus primigenius (Siberian woolly mammoth)	Mammalia	Deoxy
3d1a	Capra hircus (Goat)	Mammalia	Methemoglobin
2dhb	Equus caballus (Horse)	Mammalia	Not clear
3fh9	Pteropus giganteus (Indian flying fox)	Mammalia	Not clear
3gou	Canis lupus familiaris (Dog)	Mammalia	Oxy
3cy5	Bubalus bubalis (Domestic water buffalo)	Mammalia	Oxy
3gdj	Camelus dromedarius (Dromedary) (Arabian camel)	Mammalia	Oxy
3a0g	Cavia porcellus (Guinea pig)	Mammalia	Oxy
2b7h	Cerdocyon thous	Mammalia	Oxy
1fhj	Chrysocyon brachyurus (Maned wolf)	Mammalia	Oxy
1s0h	Equus asinus (Donkey) (Equus africanus asinus)	Mammalia	Oxy
4yu3	Helogale parvula	Mammalia	Oxy
3lqd	Lepus europaeus (European hare)	Mammalia	Oxy
1hds	Odocoileus virginianus	Mammalia	Oxy
2rao	Oryctolagus cuniculus (Rabbit)	Mammalia	Oxy
2qu0	Ovis aries (Sheep)	Mammalia	Oxy
3dht	Rattus norvegicus (Rat)	Mammalia	Oxy
1qpw	Sus scrofa (Pig)	Mammalia	Oxy

Supplementary References

1. Rojas, A. M., Fuentes, G., Rausell, A. & Valencia, A. The Ras protein superfamily: evolutionary tree and role of conserved amino acids. *J. Cell Biol.* **196**, 189–201 (2012).
2. Cox, A. D. & Der, C. J. Ras history: The saga continues. *Small GTPases* **1**, 2–27 (2010).
3. Pylayeva-Gupta, Y., Grabocka, E. & Bar-Sagi, D. RAS oncogenes: weaving a tumorigenic web. *Nat. Rev. Cancer* **11**, 761–774 (2011).
4. Stephen, A. G., Esposito, D., Bagni, R. K. & McCormick, F. Dragging ras back in the ring. *Cancer Cell* **25**, 272–281 (2014).
5. McCormick, F. KRAS as a Therapeutic Target. *Clin. Cancer Res.* **21**, 1797–1801 (2015).
6. Cox, A. D., Fesik, S. W., Kimmelman, A. C., Luo, J. & Der, C. J. Drugging the undruggable RAS: Mission possible? *Nat. Rev. Drug Discov.* **13**, 828–851 (2014).
7. Wittinghofer, A. *Ras Superfamily Small G Proteins: Biology and Mechanisms 1+2*. (Springer, 2014).
8. Macara, I. G. The ras superfamily of molecular switches. *Cell. Signal.* **3**, 179–187 (1991).
9. Spoerner, M., Herrmann, C., Vetter, I. R., Kalbitzer, H. R. & Wittinghofer, A. Dynamic properties of the Ras switch I region and its importance for binding to effectors. *Proc. Natl. Acad. Sci. U. S. A.* **98**, 4944–4949 (2001).
10. Faidon Brotzakis, Z., Zhang, S. & Vendruscolo, M. AlphaFold Prediction of Structural Ensembles of Disordered Proteins. *bioRxiv* 2023.01.19.524720 (2023) doi:10.1101/2023.01.19.524720.
11. Piovesan, D., Monzon, A. M. & Tosatto, S. C. E. Intrinsic protein disorder and conditional folding in AlphaFoldDB. *Protein Sci.* **31**, e4466 (2022).
