# Supplementary material for: Local energetic frustration conservation in protein families and superfamilies
Source: Nat Commun. 2023 Dec 16;14:8379. doi: 10.1038/s41467-023-43801-2 (PMC10725452; doi:10.1038/s41467-023-43801-2)
Supplement: Supplementary file 3 — Reporting Summary [file 41467_2023_43801_MOESM3_ESM.pdf]

nature portfolio

Corresponding author(s): Rodrigo Gonzalo Parra

Last updated by author(s): 2023/09/07

Reporting Summary

Nature Portfolio wishes to improve the reproducibility of the work that we publish. This form provides structure for consistency and transparency in reporting. For further information on Nature Portfolio policies, see our Editorial Policies and the Editorial Policy Checklist.

Statistics

For all statistical analyses, confirm that the following items are present in the figure legend, table legend, main text, or Methods section.

n/a	Confirmed	
☐	☒	The exact sample size (n) for each experimental group/condition, given as a discrete number and unit of measurement
☒	☐	A statement on whether measurements were taken from distinct samples or whether the same sample was measured repeatedly
☐	☒	The statistical test(s) used AND whether they are one- or two-sided<br>*Only common tests should be described solely by name; describe more complex techniques in the Methods section.*
☒	☐	A description of all covariates tested
☒	☐	A description of any assumptions or corrections, such as tests of normality and adjustment for multiple comparisons
☐	☒	A full description of the statistical parameters including central tendency (e.g. means) or other basic estimates (e.g. regression coefficient) AND variation (e.g. standard deviation) or associated estimates of uncertainty (e.g. confidence intervals)
☐	☒	For null hypothesis testing, the test statistic (e.g. F, t, r) with confidence intervals, effect sizes, degrees of freedom and P value noted<br>*Give P values as exact values whenever suitable.*
☒	☐	For Bayesian analysis, information on the choice of priors and Markov chain Monte Carlo settings
☒	☐	For hierarchical and complex designs, identification of the appropriate level for tests and full reporting of outcomes
☒	☐	Estimates of effect sizes (e.g. Cohen's d, Pearson's r), indicating how they were calculated

Our web collection on statistics for biologists contains articles on many of the points above.

Software and code

Policy information about availability of computer code

Data collection

Provide a description of all commercial, open source and custom code used to collect the data in this study, specifying the version used OR state that no software was used.

Data analysis

Local energetic frustration was calculated with FrustratometeR (R Package) https://doi.org/10.1093/bioinformatics/btab176
Local energetic frustration conservation was calculated with FrustraEvo, developed for this work and available at: https://github.com/proteinphysiologylab/FrustraEvo/
In addition, FrustraEvo can be used from its docker container: https://hub.docker.com/r/proteinphysiologylab/frustraevo
A webserver (beta version and unpublished) is available as well to use FrustraEvo: http://frustraevo.qb.fcen.uba.ar/

CD-Hit was used to perform sequence redundancy reduction
MAFFT v7.453 was used to produce automatic Multiple Sequence Alignments
HMMer was used to perform alignments for the KRAS family

For manuscripts utilizing custom algorithms or software that are central to the research but not yet described in published literature, software must be made available to editors and reviewers. We strongly encourage code deposition in a community repository (e.g. GitHub). See the Nature Portfolio guidelines for submitting code & software for further information.

Data

Policy information about availability of data

All manuscripts must include a data availability statement. This statement should provide the following information, where applicable:

- Accession codes, unique identifiers, or web links for publicly available datasets
- A description of any restrictions on data availability
- For clinical datasets or third party data, please ensure that the statement adheres to our policy

- experimental protein structures were retrieved from the Protein Data Bank
-protein sequences were retrieved from the NCBI protein database (April 2021)
- alphafold-v2.1.0 for structure models + scripts in https://github.com/proteinphysiologylab/FrustraEvo/tree/master/Data/scripts
- sequence analysis: BLAST 2.11.0, MAFFT v7.453, S3Det + scripts in https://github.com/proteinphysiologylab/FrustraEvo/tree/master/Data/scripts

Experimental ddPCA scores were retrieved from:
SH3 and PDZ: 10.1038/s41586-022-04586-4
KRAS: https://doi.org/10.1101/2022.12.06.519122

DATA AVAILABILITY:
All input data needed to reproduce the main results of this article as well as the intermediate outputs are available at this ZENODO repository https://zenodo.org/records/10093060, DOI 10.5281/zenodo.10093060).
Source data are provided with this article for Figs 2A, 2B, 2C, 2D, 2E, 2F, 2G, 3A, 3B, 3C, 4A, 4B, 5A, 5B, 5C, 6A, 6B

CODE AVAILABILITY:
FrustraEvo code, written in Python 3 and R 4.1.2 programming languages is available at: https://github.com/proteinphysiologylab/FrustraEvo. A Docker container is also available at (https://hub.docker.com/r/proteinphysiologylab/frustraevo).

Research involving human participants, their data, or biological material

Policy information about studies with human participants or human data. See also policy information about sex, gender (identity/presentation), and sexual orientation and race, ethnicity and racism.

Reporting on sex and gender	Not applicable. The terms sex and gender were not used in this study
Reporting on race, ethnicity, or other socially relevant groupings	Not applicable. No social analysis is done in this study.
Population characteristics	N/A
Recruitment	N/A
Ethics oversight	N/A

Note that full information on the approval of the study protocol must also be provided in the manuscript.

Field-specific reporting

Please select the one below that is the best fit for your research. If you are not sure, read the appropriate sections before making your selection.

☒ Life sciences ☐ Behavioural & social sciences ☐ Ecological, evolutionary & environmental sciences

For a reference copy of the document with all sections, see nature.com/documents/nr-reporting-summary-flat.pdf

Life sciences study design

All studies must disclose on these points even when the disclosure is negative.

Sample size	No sample size calculation was performed.<br>Sample size were defined as a result of data retrieval. Most protein families that are analysed have more than 30 family members after redundancy reduction and therefore, results are representative for the entire families.<br>As stated in the manuscript some analysis were made on purely experimental data from protein structures available in the Protein Data Bank (Hemoglobins). Other analysis were made in datasets that were built by retrieving protein sequences from public databases and their structures were predicted by using AlphaFold2.<br><br>Scripts for retrieving homologous protein sequences from public databases are available at:<br>https://github.com/proteinphysiologylab/FrustraEvo/tree/master/Data

Data exclusions	No data was excluded after retrieval using the code and public databases mentioned in the previous section
Replication	There are no replications in our study as we are using computational methods that are deterministic. The "replicates" word is present in the text as part of external datasets that were used in our analysis.
Randomization	No randomization was used. All data after redundancy reduction at the protein sequence level (using CD-HIT) were used.
Blinding	Not applicable. Blinding was not used as we are not comparing across groups.

Reporting for specific materials, systems and methods

We require information from authors about some types of materials, experimental systems and methods used in many studies. Here, indicate whether each material, system or method listed is relevant to your study. If you are not sure if a list item applies to your research, read the appropriate section before selecting a response.

Materials & experimental systems

n/a	Involved in the study
☒	☐ Antibodies
☒	☐ Eukaryotic cell lines
☒	☐ Palaeontology and archaeology
☒	☐ Animals and other organisms
☒	☐ Clinical data
☒	☐ Dual use research of concern
☒	☐ Plants

Methods

n/a	Involved in the study
☒	☐ ChIP-seq
☒	☐ Flow cytometry
☒	☐ MRI-based neuroimaging
